# Evaluating the Performance of Low-Cost Air Quality Monitors in Dallas, Texas

**DOI:** 10.3390/ijerph19031647

**Published:** 2022-01-31

**Authors:** Haneen Khreis, Jeremy Johnson, Katherine Jack, Bahar Dadashova, Eun Sug Park

**Affiliations:** 1 Texas A&M Transportation Institute (TTI), Texas A&M University System, Bryan, TX 77807, USA; j-johnson@tti.tamu.edu (J.J.); b-dadashova@tti.tamu.edu (B.D.); e-park@tti.tamu.edu (E.S.P.); 2 Center for Advancing Research in Transportation Emissions, Energy, and Health (CARTEEH), Texas A&M University System, Bryan, TX 77807, USA; 3 The Nature Conservancy, Texas Chapter, San Antonio, TX 78215, USA; kathy.jack@tnc.org

**Keywords:** low-cost sensors, air pollution, criteria air pollutants, co-location, meteorological factors, air quality index

## Abstract

The emergence of low-cost air quality sensors may improve our ability to capture variations in urban air pollution and provide actionable information for public health. Despite the increasing popularity of low-cost sensors, there remain some gaps in the understanding of their performance under real-world conditions, as well as compared to regulatory monitors with high accuracy, but also high cost and maintenance requirements. In this paper, we report on the performance and the linear calibration of readings from 12 commercial low-cost sensors co-located at a regulatory air quality monitoring site in Dallas, Texas, for 18 continuous measurement months. Commercial AQY1 sensors were used, and their reported readings of O_3_, NO_2_, PM_2.5_, and PM_10_ were assessed against a regulatory monitor. We assessed how well the raw and calibrated AQY1 readings matched the regulatory monitor and whether meteorology impacted performance. We found that each sensor’s response was different. Overall, the sensors performed best for O_3_ (R^2^ = 0.36–0.97) and worst for NO_2_ (0.00–0.58), showing a potential impact of meteorological factors, with an effect of temperature on O_3_ and relative humidity on PM. Calibration seemed to improve the accuracy, but not in all cases or for all performance metrics (e.g., precision versus bias), and it was limited to a linear calibration in this study. Our data showed that it is critical for users to regularly calibrate low-cost sensors and monitor data once they are installed, as sensors may not be operating properly, which may result in the loss of large amounts of data. We also recommend that co-location should be as exact as possible, minimizing the distance between sensors and regulatory monitors, and that the sampling orientation is similar. There were important deviations between the AQY1 and regulatory monitors’ readings, which in small part depended on meteorology, hindering the ability of the low-costs sensors to present air quality accurately. However, categorizing air pollution levels, using for example the Air Quality Index framework, rather than reporting absolute readings, may be a more suitable approach. In addition, more sophisticated calibration methods, including accounting for individual sensor performance, may further improve performance. This work adds to the literature by assessing the performance of low-cost sensors over one of the longest durations reported to date.

## 1. Introduction

Ambient air pollution is a major environmental stressor, posing a huge but modifiable health burden, particularly in urban environments. In a recent study, particulate matter with a diameter less than 2.5 μm (PM_2.5_) was estimated to result in 8.9 million (95% confidence interval (CI): 7.5–10.3) premature deaths globally; more than the number of deaths from cigarette smoking [1]. These deaths were categorized into five cause categories, which have been convincingly associated with air pollution: ischemic heart disease (IHD), stroke, chronic obstructive pulmonary disease (COPD), lung cancer, and lower respiratory infections (LRIs). Other research has also associated elemental carbon (EC), nitrogen dioxide (NO_2_), and Ozone (O_3_), amongst other pollutants, with premature mortality and a wide spectrum of diseases, including adverse birth outcomes, respiratory outcomes in children and adults, and cardiometabolic outcomes [2]. The health burden of air pollution is not fully elucidated and could increase as more evidence emerges on the adverse effects of various pollutants on new health outcomes such as autism, cognitive decline, neurodegenerative diseases such as dementia, Alzheimer’s, and Parkinson’s disease, diabetes, and obesity [3]. Higher air pollution exposures and the associated adverse health effects are unequally distributed, with lower socioeconomic classes and ethnic minorities suffering the most overall [4,5].

To study the myriad of health effects of air pollution and devise adequate air quality guidelines, standards, and exposure mitigation strategies, human exposure to air pollution must be first assessed. The assessment of ambient air pollution, and subsequently the assignment of human exposures, can be done using a wide variety of methods, which are broadly classified as measurement, modeling, or the use of air pollution exposure surrogates, such as proximity to major roadways or traffic density within certain distances from a residence [2]. Air pollution measurements from fixed-site reference-grade and regulatory monitors may be considered as the gold standard, as they offer direct observations (rather than estimations) of pollutant concentrations and subsequent exposures and, importantly, undergo stringent quality assessment and control. These regulatory monitors can measure multiple pollutants with a high degree of accuracy and high temporal resolution. However, due to their high costs and maintenance requirements, they are only present in limited quantities and have a low spatial coverage [6]. In addition, their locations are selected based on regulatory purposes, rather than scientific ones [7] (e.g., siting based on random selection may be considered the gold standard for data collection and analysis). This hinders the ability to characterize the profile of urban air pollution (exposures) and its well-established spatial variability, which can vary by five times within a single city block [8,9]. Moreover, due to the lack of an established study design when installing these devices, the measurements collected from these sources may not be adequate for estimating exposures and scaling them to larger geographies. Capturing the large variability of air pollution within urban areas is critical, as it underpins the ability of epidemiological studies to pinpoint adverse health effects and risk assessment studies to identify hotspots and the distribution of pollutants and attributable health burdens by, for example, socioeconomic class or ethnicity. Despite these limitations, numerous studies have had to rely on insufficient spatial data from fixed-site and regulatory monitors over the years, due to lack of feasible alternative methods.

In recent years, an important advancement in air pollution measurement technology has occurred: the development and deployment of low-cost and portable air quality sensors. Low-cost air quality sensors are flooding the market, and sensors are being used more often for numerous applications, which continue to expand [10] and can somewhat mimic application of regulatory and reference-grade monitors. The emergence of low-cost sensors has generated great interest among researchers and community members hoping to better understand the intersection of local air quality and health through broader geographic deployment. Low-cost sensors were also identified as an integral aspect of a ‘changing paradigm of air pollution monitoring’, consisting of a shift from reliance on government and regulatory monitoring to the use of low-cost, portable, easy-to-operate sensors; made possible by advances in microfabrication techniques, microelectromechanical systems, and energy efficient radios and sensor circuits [11]. Low-cost sensors can result in spatially denser monitoring and resolved pollution measurements, which can advance research, policy, and practice, and more effectively direct programs and resources to address local pollution, health, and environmental justice. Other potential applications include air pollution warnings, epidemiological studies, and model validation. Some studies investigated data fusion methods by combining low-cost sensor measurements with well-established air quality models and evaluated the resulting air pollution maps [12,13]. Low-cost sensors also offer data at fine temporal resolution, and such data may be used to study the correlations between air pollution concentration and acute or short-term health effects. Despite the increasing popularity of these sensors, only a few studies have examined their performance (i.e., accuracy, precision, reliability, and reproducibility) under real-world conditions and against regulatory air quality monitors. This is a crucial and under-researched field; given the proliferation of low-cost sensors and their data, and the associated interpretation challenges [14]. The validity and uncertainty of low-cost sensor measurements over a range of different meteorological and aerosol loading environments need to be better quantified, and this was flagged as a research gap [15].

To understand the state of the research in this field, we conducted a literature review including twenty-five recent studies, as summarized in Table S1. As shown in Table S1, low-cost sensors, and the corresponding reference monitors used for comparison, measured either one or multiple gaseous and particulate pollutants, most commonly nitrogen oxide (NO), NO_2_, O_3_, carbon monoxide (CO), carbon dioxide (CO_2_), sulfur dioxide (SO_2_), PM_2.5_, particulate matter with a diameter of less than 10 μm (PM_10_), and particulate matter with a diameter of less than 1 μm (PM_1_). Many studies also measured meteorological parameters that may impact the performance of the low-cost sensors, such as relative humidity (RH), temperature (Temp), dew point (DP), atmospheric pressure (P), wind speed (WS), and wind direction (WD). One study measured ambient light (AL) [16]. Most studies evaluated low-cost sensor performance through co-location with a regulatory or a refence-grade monitor, without applying additional calibration methods. Some studies suggested that RH can hinder the accuracy of optical particle sensors, because of the detection of water droplets in addition to particulate matter [17], and cautioned against using low-cost sensors for measuring particulate matter in high RH conditions [10,18,19,20]. Many sensors failed in periods of high and sustained RH [21]. Sensor performance seemed to be negatively affected at higher air pollution concentrations [19], including during sand and dust storms, with a trend toward underestimating at those levels and when RH was >75%. Moreover, low-cost air quality sensors can suffer from a degraded response over time, leading to the drift of measured concentrations; while gaseous sensors of a specific pollutant can suffer from cross-sensitivities to other pollutants, generating false sensor responses [22,23]. Research also suggests that the r^2^ values for low-cost sensors have a wide range, when evaluated against field reference monitors; for example, between 0.4–0.8 [22]. These variabilities, uncertainties, and unknowns can result in a lack of confidence in data quality and in users not knowing if, and which, low-cost sensors may fit their intended applications. Quantifying the performance of sensors in real-world conditions is critical to ensure sensors will be used in a manner commensurate with their data quality [15].

In the face of their limitations and remaining knowledge gaps, low-cost sensors present new opportunities for ubiquitous monitoring and hold a lot of promise, due to their small size, low-cost, and ease of use. Their deployment can provide a more complete assessment of the spatiotemporal variability of urban pollution when, compared to traditional monitoring, and identify hotspots and concentrations affecting personal and community exposures. They may also allow communicating the state of air quality, through for example, established air quality categories and indices with health implications for the public [24,25,26], such as the Air Quality Index (AQI); used by the U.S. Environmental Protection Agency (USEPA) to allow information about air quality and its impacts on health to be relayed to the public, so they can avoid harmful situations [27].

In this work, we explore the performance and calibration of 12 commercial low-cost sensors co-located at a regulatory (reference) air quality monitoring site in Dallas, Texas, for 18 continuous months; the longest assessment duration, to the best of the authors’ knowledge. AQY version 1 (AQY1) sensors, manufactured by Aeroqual, New Zealand, were selected for this study. We assessed how well the raw and calibrated low-cost air quality sensors’ readings matched readings from the reference monitor, and whether meteorological factors impacted the sensors’ performance. This work adds to a growing, but limited, body of literature assessing the performance of low-cost sensors in real-world environments, with one of the longest assessments. As shown in Table S1, only a handful of studies have assessed the performance of AQY sensors, and particulate matter was the pollutant most studied. In this study, our data spanned a realistic range of concentrations and meteorological variables, captured by 12 sensors operated at the same location. We used the well-established co-location method and inspected precision, bias, and mean error for four criteria pollutants with adverse health effects: O_3_, NO_2_, PM_2.5_, and PM_10_. We also systematically investigated the impact of the meteorological factors, Temp, RH, WS, and WD on accuracy parameters. Finally, we compared the low-cost sensor readings with the reference monitor’s readings using AQI categories; as such, investigating this potential option for utilizing and communicating data from low-cost sensors.

## 2. Materials and Methods

### 2.1. Low-Cost Sensors and Pollutants Evaluated

We evaluated 12 low-cost air quality sensors of the same type: AQY1. These units were first released in June 2018 [28], and at the time of purchase (August 2018), they were the latest version on the market. The research team looked at a few different ‘low-cost’ air quality sensors, and the Aeroqual AQY1 devices were selected for use in the project based on multiple criteria. First, the cost of the sensors, approximately USD 4000/device, was in the mid-range of costs for low-cost sensor options, with some options costing as little as a few hundred dollars and other over USD 10,000. The units also monitored multiple pollutants, including PM and gases, which was a positive factor that led to the final selection of the AQY1 units.

The AQY1 units report the minute-by-minute concentrations of four criteria pollutants: NO_2_ and O_3_, both measured in parts per billion (ppb), and PM_2.5_ and PM_10_, both measured in microgram per cubic meter (ug/m^3^). The AQY1 units contain two separate sensor boards to measure pollutants: one for measuring gases, and the other for measuring particles. The units also collect information on RH and Temp at the same resolution as the pollutant data. The AQY1 units come fully assembled out of the box and are ready to be plugged into a power source and used.

To calibrate and evaluate the performance of the low-cost sensors, the 12 units were all co-located at the same reference site in Hinton, Dallas, where a high-cost regulatory air quality monitor was operating continuously. The Hinton monitor is operated by the City of Dallas for the Texas Commission on Environmental Quality (TCEQ) [29] and records all pollutants measured by the AQY1 units, in addition to Temp, RH, and wind data (WS and WD) [30], which we used to investigate the impact of meteorological variables on the low-cost sensor performance. All data from the Hinton, Dallas reference site is available from the TCEQ’s website at https://www.tceq.texas.gov/cgi-bin/compliance/monops/daily_summary.pl?cams=401, accessed on 14 September 2019. We obtained and used the RH, Temp, WS, and WD to investigate whether the performance of the low-cost sensors was affected by meteorology. This co-location procedure follows co-location calibration protocols recommended by the USEPA, and the AQY1 user guide [28]. By locating the low-cost air quality sensors at the same site as the reference (regulatory) monitor, the data from the two could be compared under real-world conditions, to assess the performance of the low-cost sensors. In addition, calibration factors can be calculated for the low-cost sensors to increase the accuracy of their data and achieve a better fit with the data measured by the regulatory air quality monitor. Furthermore, the AQY1 user guide recommends that at the regulatory co-location site there should ideally be some hourly values for O_3_ > 60 ppb, NO_2_ > 40 ppb, and PM_2.5_ > 50 ug/m^3^. These conditions were also met in this study.

Except as noted below, both the low-cost sensors and the reference air quality monitor operated continuously for a period of 18 months, and their measurements were timestamped and later matched using the timestamp to conduct the analyses. As the reference air quality monitor only reported hourly averages for the four pollutants, the minute-by-minute readings of the AQY1 monitors were converted into hourly averages. This approach allowed managing the noise in the data, by averaging the minute-by-minute readings, as reported by the AQY1 monitors, to hourly averages, comparable to the regulatory monitor. No further assessment or correction for noise was undertaken in this study.

Measurements for this study were taken continuously between 11 February 2019 and 31 August 2020, which we used as the start and end dates for all our analyses. Each AQY1 unit had both Wi-Fi and cellular capabilities, to allow for connection and periodic data transfer to a proprietary Aeroqual Cloud system.

There are essentially three options for secure data retrieval by users. First, the user can login into the Cloud system, choose a date range for download, and instantly download data from single or multiple AQY1 units over the selected time frame. This may be considered a manual user process. Second, the user can choose to have the Cloud auto-generate daily/weekly/or monthly reporting emails for each AQY1 unit. This is considered an automated process. Finally, is the option we used: the user can use an application program interface (API). Through the API, the user can send a request to the Cloud including a beginning and end date (selected time frame) and the device IDs the user is interested in. The response to this request will be the requested data, which can then be reformatted as needed for storage and analysis. We did not use the option of auto-generating periodic reports by e-mail, and although we were downloading data periodically, we did not conduct periodical checks or analysis of the data. We recommend that users utilize this automatic retrieval option and have a plan in place to check the data, which may have helped in better managing data quality control and assurance. Each AQY1 unit also has an onboard computer, which includes a memory card where the data is automatically stored if connection to the Aeroqual Cloud system is lost. Once a connection has been re-established, the unit will upload all saved data to the Aeroqual Cloud system.

Beyond the cellular capabilities mentioned above, the AQY1 units have Wi-Fi capabilities, which is only relevant if the user is in close proximity to the unit. Through the Wi-Fi capability, the data from the AQY1 unit can be retrieved directly from the device itself via the device’s internal web server. Any Wi-Fi enabled device can connect directly to the AQY unit and download the data. For this project, all data were collected using the Cloud system’s API feature and a source code developed in Python to download the data to a local database for analysis.

The Cloud system also includes a function to calibrate each unit, which in our case was done through co-location at the reference monitor site (Hinton). This function allows a user to upload data from the reference monitor, for comparison with the AQY1 unit’s data, and automatically calculates new calibration factors (as discussed in Section 2.3). The user then needs to manually apply the new calibration factors to the AQY1 unit’s data in the cloud system. From that point onwards (after manually applying the new calibration factors), these factors are implemented in the cloud system, until the next time new reference monitor data is uploaded or new calibration factors are manually entered by the user.

We recommend that future users utilize the automated daily/weekly/or monthly reporting emails for each AQY1 unit, periodically check the quality of the data, and replace sensors or recalibrate as needed. In addition, there is no clear guidance on how much data are needed for calibration, and it took us some time to decide on the 1-month of data for calibration, as will be discussed next. The process described above, in addition to needing 1-month of data for each calibration led to relatively large amounts of missing data in the calibrated dataset.

Over the course of data collection for this study, a total of 11 sensor boards required replacement, due to failure and their limited lifetime. Data collected during these events (between the time the sensor was reported as faulty until it was replaced) was excluded from the comparison to the reference air quality monitor. Additionally, some data were missing due to power loss at the site, which sometimes required an in-person power re-set and did not happen immediately (data collection was in Dallas, Texas, while the research team was in College Station, Texas, ≈ 180 miles away). The percentage of data lost due to power losses, in addition to data collected between the time the sensor was reported as faulty until it was replaced and calibrated (which was sometimes not immediate, as described above), was approximately 20% of the overall data and was not included in the analysis. When conducting data analysis, both the unavailable data, due to faulty sensor replacement and need for a new calibration, and missing data due to power loss, were treated as ‘Not Available’ (N/A).

### 2.2. Site Set-Up and Instrumentation

**Figure 1:**
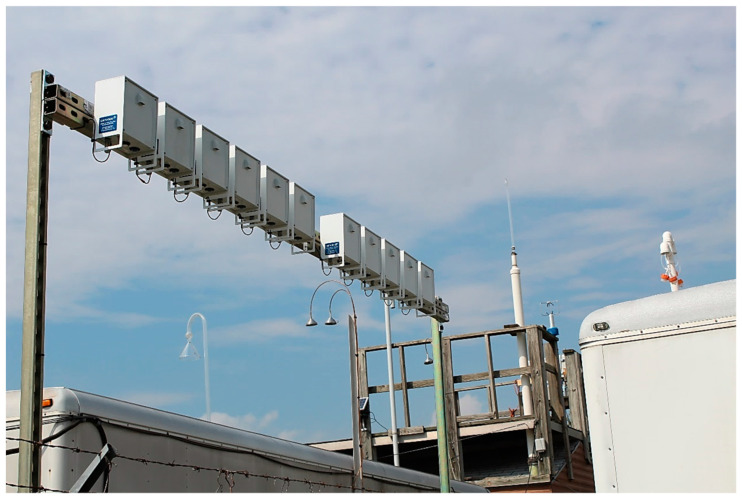
AQY1 Units Installed at the Reference Site at Hinton, Dallas, Source: Own Photo.

The reference air quality monitor had the following parameters: USEPA Site Number was 481130069, located at 1415 Hinton Street, 75235, with the following site coordinates: latitude: 32°49′12″ North (32.8200660°), longitude: −96°51′36″ West (−96.8601230°). The low-cost air quality sensors were placed approximately 7 inches apart, sensor to sensor, near the regulatory monitoring station’s inlet for gases, which is shown circled in red in Figure 1. The location of each sensor’s inlet is shown in Figure S1. The distance from the regulatory monitoring station’s inlet for gases to the AQY1 monitors was between 15 and 25 feet, and both inlets were at approximately the same height of 10 feet. However, the regulatory monitoring station’s instruments for PM_2.5_ and PM_10_ were mounted on a ground level cement pad, approximately 29 feet from the AQY1 monitors, and at a height difference of 7 feet 5 inches (Figure S2). This was the only possible installation, due to the site’s set-up and space availability.

Table 1 shows the details of the instruments used in the AQY1 units and the reference monitor at Hinton, highlighting the different gases and particle measurement methods, which contributed to the difference in readings.

### 2.3. Linear Calibration

Each of the 12 units had a unique ID and was investigated separately. We expected that each sensor would have a different performance, and as such, we planned on conducting the calibration and performance assessment separately for each sensor (i.e., the calibration factors were calculated separately for each sensor and not for all sensors together). The device IDs for the 12 units were: AQY1-BA-479A; AQY1-BA-480A; AQY1-WilburSpare-07; AQY1-WilburSpare-08; AQY1-WilburSpare-09; AQY1-WilburSpare-10; AQY-BA-353; AQY-BA-431; AQY-BA-432; AQY-BA-464; AQY-BA-480; and AQY-BA-481.

The raw and calibrated data from the low-cost sensors were compared against the reference monitor’s data, separately for each pollutant and each AQY1 unit, at 1-h intervals. The data calibration was conducted as follows. The two data sets (the low-cost sensors versus the reference monitor’s data) were plotted against each other in a scatter plot. The slope and offset of the linear least squares fit line of the data was then calculated, and these parameters were used to calculate the new gain and offset calibration factors for each pollutant and each AQY1 unit (as distinct from the default gain (1) and offset (0)). The formulas used to calculate the new gain and offset for the AQY1 units were

(1) $$\mathrm{New}\;\mathrm{Gain}=\frac{\mathrm{Current}\;\mathrm{Gain}}{\mathrm{Slope}}$$

(2) $$\mathrm{New}\;\mathrm{Offset}=\mathrm{Current}\;\mathrm{Offset}+(\frac{\mathrm{Intercept}}{\mathrm{Current}\;\mathrm{Gain}})$$

These factors were then entered into the Aeroqual Cloud system [31], which applies the calibration factors to the raw values using the equation below.

(3) $$\mathrm{Calibrated}\;\mathrm{Value}=\mathrm{Gain}*\left(\mathrm{Raw}\;\mathrm{Value}-\mathrm{Offset}\right)$$

As the equation outlines, the offset represents a shift in each raw data point, either positive or negative, and the gain was a multiplier for the value, after the shift from the offset. When first installed, a new unit has default calibration values of 1 (gain) and 0 (offset). Applying the calibration equation with these default values does not alter the reported data, which are therefore treated as raw data in our analyses.

We calibrated each unit after it had collected a minimum of 1 months’ worth of data, with the first calibration occurring in February 2019. In February 2019, all units had been at the Hinton site and had collected data for one month. The literature does not establish firm recommendations as to the amount of data needed before a calibration can be conducted or between maintenance calibration(s); however, more data may provide for a better calibration. The manufacturer (Aeroqual) suggests a minimum of 3 days, when using hourly data [31]. After the calibration interval is complete, the new calibration values (both gain and offset) were calculated using the data from the AQY1 unit and the reference monitor at the same location using slopes and intercepts from this data. After they were calculated, the new calibration values (both gain and offset) were entered into the Aeroqual Cloud system and applied, and the data from that point forward were considered to be calibrated data. In September 2019, the units were calibrated again using the same methodology as above, as we had originally planned to move them to different sites across Dallas for another field study, but this plan was halted due to delays in the field study (not discussed here, but part of the bigger project). As such, every unit was calibrated twice, in February and September 2019, and again if a sensor had to be replaced due to its limited lifetime. Units AQY-BA-480 and AQY-BA-481 were only calibrated in September 2019, as they were purchased later than all other units, in June 2019, as back-up units for the other field study planned.

Due to the nature of the study, and the question that was being asked, we did not repeat calibrations of the low-cost sensors on a set schedule, and did not specifically conduct maintenance calibrations (i.e., regularly re-calibrating on a set schedule, for example, every three months). Therefore, in this study, we did not address the drift or change in low-cost sensor performance over time and do not make recommendations as to how calibration should be conducted or maintained, as this was not the objective of our work. Researchers interested in accessing the data for further analysis can reach out with their request to HK or KJ on the research team.

### 2.4. Data Analysis

To assess the performance of the AQY1 units against the refence monitor, we conducted an exploratory data analysis, regression analysis, and analysis of covariance (ANCOVA). The reference air quality monitor’s data were considered the ‘True Values’ for pollutants, and free from error. Data obtained from AQY1 units were labeled as ‘Raw Data’, while calibrated data were labeled as ‘Calibrated Data’. We briefly describe these next. All analyses were conducted using R and JMP (SAS product).

The exploratory data analysis was conducted using multiple summary statistics and graphics, where we used both the raw and the calibrated data for each pollutant and each AQY1 unit separately, to assess the performance of the low-cost sensors. We calculated and present the descriptive (summary) statistics for the whole datasets using the raw data from all low-cost sensors, the calibrated data from all low-cost sensors, the reference monitor’s readings, and the differences between the low-cost sensors and the reference monitor’s readings. We also plotted the time series to visually elucidate the trends over time. Finally, we compared and assessed the differences between the readings from the low-cost sensors and the reference monitor using the mean average percentage error (MAPE). We selected this metric because it is easy to interpret. Its calculation is shown below:

(4) $$\mathrm{MAPE}=\frac{1}{N}\overset{N}{\underset{i=1}{\sum }}\left|\frac{y_{i}-{\hat{y}}_{i}}{y_{i}}\right|\times 100\%$$

where N is the number of observations, $y_{i}$ is the reference monitor’s reading, and ${\hat{y}}_{i}$ is the raw or calibrated reading from the low-cost sensor.

Measurements from the reference air quality monitor (assumed to be free from measurement error) were plotted on the x-axis, and measurements from the low-cost sensors (which are subject to measurement error) were plotted on the y-axis in scatter plots. As the performance of each AQY1 unit was expected to be different, the accuracy (regression) analysis was carried out separately for each sensor and each pollutant. We assessed systematic bias by inspecting the slope of the estimated regression line and the intercept. A deviation from the slope of 1 indicates a proportional discrepancy between the reference monitor and a low-cost monitor and indicates that a low-cost monitor is subject to a proportional systematic error. A non-zero intercept represents an absolute discrepancy or an absolute systematic error. We also calculated the root mean square error (RMSE) for the regression line, as follows:

(5) $$\mathrm{RMSE}=\sqrt{\frac{1}{\left(N-2\right)}\overset{N}{\underset{i=1}{\sum }}{\left(y_{i}-{\hat{y}}_{i}\right)}^{2}}$$

where N is the number of observations, $y_{i}$ is the reference monitor’s reading, and ${\hat{y}}_{i}$ is the raw or calibrated reading from the low-cost sensor.

We also obtained meteorological data from the Hinton site and investigated how the performance of each unit was affected depending on meteorological conditions: Temp, RH, WD, and WS. To analyze the potential impact of meteorological conditions on the performance of the low-cost sensors, the differences between the low-cost sensor data and the reference monitor’s data were analyzed using the analysis of covariance (ANCOVA) model, with the Device ID as a categorical factor and meteorological variables as covariates (continuous variables), to assess the effects of meteorological variables on measurement errors in the low-cost sensor data.

### 2.5. Comparison with the United States Environmental Protection Agency’s Air Quality Index Categories

In addition to the above analysis, which relied on comparing the absolute air pollutant readings from the low-cost sensors and the reference air quality monitor, we also conducted an analysis of the performance of the low-cost sensors using the AQI categories put forward by the USEPA [27]. The AQI is an index value, which runs from 0 to 500, and is calculated using the concentration measurements of the pollutant of interest. It is applicable to the four pollutants measured by the low-cost sensors: NO_2_, O_3_, PM_2.5_, and PM_10_. The AQI is split into six categories, each with a different level of health concerns, ranging from ‘Good’, which corresponds to little or no health-related risk, to ‘Hazardous’, which corresponds to an emergency level health concern.

The equation used to calculate the AQI uses a time-averaged value of the measured concentration of each pollutant. The time used to calculate the average concentration in the AQI equation varies by pollutant. For NO_2_, a 1-h average is used, for O_3_, either a 1-h or an 8-h average is used, and for both PM_2.5_ and PM_10_, a 24-h average is used [32]. The equation used to calculate the AQI is shown below.

(6) $$\mathrm{AQI}=\frac{\left(\mathrm{AQI}\_\mathrm{hi}\right)-\left(\mathrm{AQI}\_\mathrm{lo}\right)}{\left(\mathrm{CONC}\_\mathrm{hi}\right)-\left(\mathrm{CONC}\_\mathrm{lo}\right)}*\left({\mathrm{CONC}}_{i}-\mathrm{CONC}\_\mathrm{lo}\right)+\left(\mathrm{AQI}\_\mathrm{lo}\right)$$

where CONC_i = the average value of the pollutant over the corresponding period of time as above; CONC_lo = the concentration value at the low end for the given AQI level; CONC_hi = the concentration value at the high end for the given AQI level; AQI_hi = the maximum AQI index value for the given CONC_i; AQI_lo = the minimum AQI index value for the given CONC_i (United States Environmental Protection Agency, 2020b).

The AQI_hi, AQI_lo, CONC_hi, and CONC_lo values required in the above equation were provided by the USEPA and are presented in Table S2. We used the equation above, and values shown in Table S2, to calculate the AQI levels using readings from both the low-cost air quality sensors and the reference monitor and compared the two.

## 3. Results

### 3.1. Descriptive Summary Statistics and Comparison between the AQY1 Monitors and the Reference Monitor

Table 2 shows the descriptive (summary) statistics for both the raw and the calibrated data from all the AQY1 monitors against the reference monitor at the Hinton site, for the four pollutants studied. Note that, as we show later, the performance varied considerably by device, but Table 2 is meant to give a general overview of the combined data. Results by Device ID are considered to be more meaningful.

The reference monitor reported 46 negative readings for O_3_, 185 negative readings for NO_2_, 579 negative readings for PM_2.5_, and 359 negative readings for PM_10_. As there cannot be real negative pollutant concentrations in ambient air, and as our underlying assumption is that the reference monitor’s readings were the true values of the pollutants and free from error, we replaced these negative values with zeros in all our analyses. Since the objective of this work was not to assess the performance of the reference monitor at the Hinton site, this practice was considered acceptable. Next, we overview the differences between the reference and the AQY1 monitors, by pollutant.

#### 3.1.1. Ozone

The difference between the reference and the AQY1 monitors for the overall dataset was reduced after the calibration for all summary statistics, except for the maximum value; indicating that overall, the calibration seemed to achieve its intended purpose. The general trend in the data was that the AQY1 monitors tended to overestimate O_3_ values across all summary statistics. Therefore, it seems that the AQY1 monitors may have over-reported the true O_3_ concentrations, with the potential for specifically overreporting at the highest O_3_ concentrations by up to 63% (calibrated data), as compared to the reference monitor (Table 2).

#### 3.1.2. Nitrogen Dioxide

The difference between the reference and the AQY1 monitors for the overall dataset was reduced after the calibration for all summary statistics, except for the 3rd quartile value, indicating that overall, the calibration seemed to achieve its intended purpose. The general trend in the data was that the AQY1 monitors tended to underestimate NO_2_ values across the minimum, 1st quartile, median, mean, and 3rd quartile, but overestimate the maximum NO_2_ value, with a large deviation in the raw dataset (356%), which was reduced, but remained high, in the calibrated dataset (143%). Therefore, it seems that the AQY1 monitors may underreport the true NO_2_ concentrations with the potential for specifically overreporting at the highest NO_2_ concentrations by up to 143% (calibrated data), as compared to the reference monitor. The AQY1 monitors recorded many negative NO_2_ readings in the raw dataset, which were corrected after applying the calibration factors (Table 2).

#### 3.1.3. Particulate Matter with a Diameter Less Than 2.5 μm

The difference between the reference and the AQY1 monitors for the overall dataset was reduced after the calibration for all summary statistics, indicating that overall, the calibration seemed to achieve its intended purpose. The general trend in the data was that the AQY1 monitors tended to underestimate PM_2.5_ values across the 1st quartile and median (i.e., lower ends of the air pollution range), and overestimate across the mean, 3rd quartile, and maximum calibrated values (i.e., higher ends of the air pollution range). Therefore, it seems that the AQY1 monitors may have underreported the true PM_2.5_ concentrations but with the potential for overreporting at the highest concentrations by up to 103% (calibrated data), as compared to the reference (Table 2), but this also varied by device (data not shown).

#### 3.1.4. Particulate Matter with a Diameter Less Than 10 μm

The difference between the reference and the AQY1 monitors for the overall dataset was reduced after the calibration for all summary statistics, except the maximum value, which only slightly increased (≈1%), indicating that overall, the calibration seemed to achieve its intended purpose. The general trend in the data was that the AQY1 monitors tended to underestimate PM_10_ values across the 1st quartile and median (i.e., lower ends of the air pollution range), and overestimate across the mean, 3rd quartile, and maximum calibrated values (i.e., higher ends of the air pollution range). Therefore, it seems that the AQY1 monitors may have underreported the true PM_10_ concentrations, but with the potential for overreporting at the highest PM_10_ concentrations by up to 35% (calibrated data), as compared to the reference monitor (Table 2), but this also varied by device (data not shown).

#### 3.1.5. Mean Average Percentage Error

We also assessed the differences between readings from the reference monitor and the AQY1 monitors using MAPE. The detailed results are shown in Table S3 in the Supplementary Material.

For O_3_, the MAPE (%) ranged from 20% (AQY-BA-353) to 84% (AQY-BA-432) in the raw data, and from 5% (AQY-BA-353) to 45% (AQY-BA-480) in the calibrated data. For NO_2_, the MAPE (%) ranged from 145% (AQY1-WilburSpare-09) to 361% (AQY-BA-431) in the raw data, and from 26% (AQY-BA-480 and AQY-BA-481) to 106% (AQY-BA-464) in the calibrated data. For PM_2.5_, the MAPE (%) ranged from 41% (AQY-BA-464) to 68% (AQY1-BA-479A) in the raw data, and from 29% (AQY1-WilburSpare-07) to 132% (AQY1-BA-480A) in the calibrated data. For most AQY1 monitors, the MAPE increased in the calibrated PM_2.5_ data, which is problematic. For PM_10_, the MAPE (%) ranged from 36% (AQY-BA-464) to 63% (AQY-BA-353 and AQY1-WilburSpare-09) in the raw data, and from 20% (AQY1-WilburSpare-07) to 77% in the calibrated data (AQY1-BA-479A and AQY1-BA-480A). As such, these results show that there were high variations between the AQY1 monitors, and according to the pollutants examined, and that the performance based on the MAPE deteriorated in the PM_2.5_ dataset.

#### 3.1.6. Time Series Plots

We also plotted the time series to elucidate the pollutant trends over time, comparing between the AQY1 monitors and the reference monitor. While there were differences between the AQY1 monitors, the general trend was that the time series patterns were consistent across the AQY1 and the reference monitors, where the high and low readings generally coincided. Select examples are shown in Figures S3–S6 in the Supplementary Material.

### 3.2. Regression Analysis

Table 3 contains a summary of regression analyses of for all the data, while the regression lines for the calibrated data, separately by AQY1 monitor, are shown in full in the Supplementary Material (in the ‘Underlying Regression Analysis Results’ section). For O_3_, it can be observed from Table 3 that the performance (bias and precision) of the low-cost monitors varied considerably by monitor. In general, calibration seemed to improve accuracy significantly, except for AQY1-WilburSpare-07, AQY-BA-480, and AQY-BA-481 (for which the bias in the calibrated data seems to be larger or about the same). There seemed to be a non-negligible absolute systematic error (non-zero intercept that is significantly different from 0) and/or non-negligible proportional systematic error (slope that is significantly different from 1) in the data from some monitors (e.g., AQY1-WilburSpare-07, AQY-BA-464, AQY-BA-480, and AQY-BA-481), even after calibration. The RMSE values in Table 3 are precision estimates for the measurements from the monitors. It appears that calibration improved the precision in general (except for AQY1-WilburSpare-07, AQY-BA-464, AQY-BA-480, and AQY-BA-481). For the calibrated data, R^2^ ranged from 0.36 to 0.97, with an average of 0.84. All AQY1 monitors, expect AQY1-WilburSpare-07 and AQY-BA-464 had an R^2^ of 0.86 or above.

There were many negative values in the raw NO_2_ data obtained by the AQY1 monitors, which is problematic. Those negative values were corrected by calibration. In general, calibration seemed to improve accuracy (except for AQY1-WilburSpare-07 and AQY-BA-464). There seemed to be a non-negligible absolute systematic error and/or non-negligible proportional systematic error in the NO_2_ data from several monitors (AQY1-WilburSpare-07, AQY1-WilburSpare-08, AQY-BA-353, AQY-BA-431, AQY-BA-432, AQY-BA-464, and AQY-BA-481), however, even after calibration. The RMSE values were large in general, which indicates that the precision of the monitors was low. It appears that calibration improved the precision in general. The scatterplots of the reference NO_2_ data and the AQY1 monitors NO_2_ data, showed a much wider spread around the regression compared to those for O_3_ (data not shown). For the calibrated data, R^2^ ranged from 0.00 to 0.58, with an average of 0.35 and a median value of 0.37.

For PM_2.5_, the performance (bias and precision) of the AQY1 monitors varied by monitor. In general, calibration seemed to decrease the bias in PM_2.5_ data obtained from the AQY1 monitors. The RMSE values, however, were larger for the calibrated data, which indicates that the precision of PM_2.5_ data from the low-cost monitors may have deteriorated after calibration, except for AQY-BA-481. For the calibrated data, R^2^ ranged from 0.20 to 0.39, with an average of 0.32 and a median value of 0.33.

For PM_10_, the performance (bias and precision) of the AQY1 monitors varied by monitor. In general, calibration seemed to improve accuracy. Like PM_2.5_, but unlike O_3_ or NO_2_, the RMSE values for the calibrated PM_10_ data were larger (the precision is lower) than those for raw PM_10_ data, except for AQY-BA-481. For the calibrated data, R^2^ ranged from 0.36 to 0.54, with an average of 0.47 and a median value of 0.49. One monitor reported no PM_10_ data for the whole project duration: AQY-BA-480. This was due to the PM_10_ sensor in the device being deactivated in the Cloud system by the manufacturer.

### 3.3. Analysis of Covariance

We analyzed the potential impact of meteorological conditions on the performance of the AQY1 monitors using the ANCOVA model with measurement errors, defined by the difference between the low-cost monitor measurements and the reference monitor (referred to as Hinton) measurements as a dependent variable, Device ID as a categorical factor, and meteorological variables as covariates. Detailed ANCOVA results are shown in the Supplementary Material in the ‘Underlying ANCOVA Results’ section.

#### 3.3.1. Ozone

**Figure 2:**
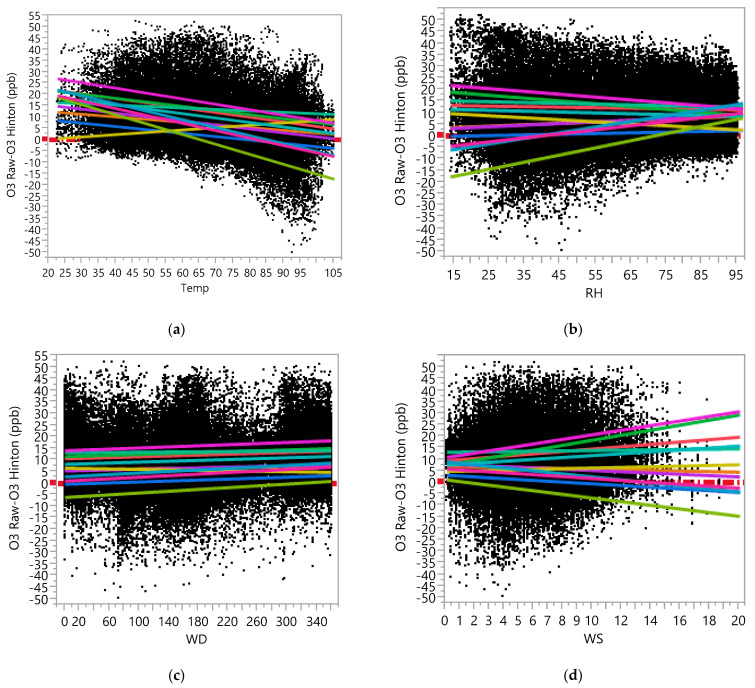

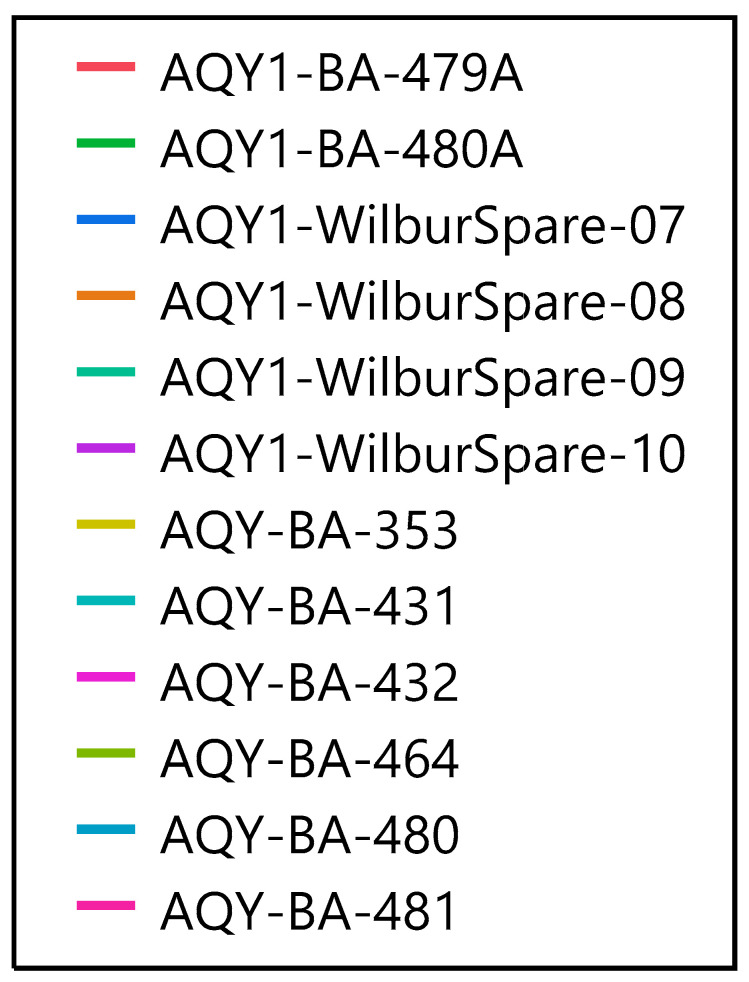
Regression plot between O_3_ Raw-O_3_ Hinton Data versus (**a**) Temperature, (**b**) RH, (**c**) Wind Direction, and (**d**) Wind Speed, as measured at the Hinton Reference Site.

In the raw dataset, the ANCOVA model with measurement error (computed by O_3_ Raw—O_3_ Hinton) as a dependent variable, Device ID, Temp, RH, WD, and WS as main effects, and two-way interaction effects among them indicated that the effects of the meteorological variables on the measurement errors in the raw data of the AQY1 monitors varied by sensor. Table S4 contains the results of the ANCOVA for the dependent variable O_3_ Raw—O_3_ Hinton, which shows that there were statistically significant interaction effects between the Device ID and each of Temp, RH, WD, and WS. While all four meteorological variables were statistically significant, due to the large sample sizes, not all those effects seemed to be practically significant. Only the effects of Temp, RH, and WS were deemed to be practically significant (effects sizes of Temp, RH, and WS are larger compared to those of WD). Figure 2 presents the regression plots based on the ANCOVA analysis results, including each of Temp, RH, WD, and WS in the model along with Device ID and the interaction effect between them (see Supplementary Material for the underlying ANCOVA results for Figure 2). From Figure 2, it can be observed that the effect of Temp on the differences between the raw and the Hinton’s O_3_ data varied by Device ID, although the effect was negative in general (except for AQY-BA-353). That is, the AQY1 monitors tended to overestimate at lower temperatures and underestimate at higher temperatures (except for AQY-BA-353). The effect of RH on the differences also seemed to be negative in general (except for three devices). The effect of WD on the differences seemed to be negligible. The effect of WS on the differences seemed to be different for each sensor (see regression plot in Figure 2), so it may not be meaningful to assess the effects of those variables without referring to the Device ID. For the AQY1 monitors such as AQY-BA-432, AQY-BA-479A, and AQY-BA-480A, the low-cost monitor readings overestimated more as the WS increased, but for some other monitors (e.g., AQY-BA-464, AQY-BA-480, AQY-BA-481, and AQY1-WilburSpare-07), the low-cost monitors underestimated more as the WS increased.

We also conducted the same analysis using the calibrated dataset but note that we consider the raw data analysis the main analysis, as it is representative of the AQY1 monitors’ performance before being altered by the calibration, and as the raw data set was larger and more complete. The results from the calibrated data set are in the Supplementary Material and showed that, overall, the effects of meteorological variables on measurement errors were found to be smaller compared to those for the raw O_3_ data, but less consistent. In the case of Temp, there was evidence that the differences (O_3_ Calibrated-O_3_ Hinton) did not significantly vary with Device ID, except for AQ1-WilburSpare-07. For AQ1-WilburSpare-07, the monitor tended to overestimate at lower temperatures and underestimate at higher temperatures. For RH, WD, and WS it appeared that the calibration deteriorated the performance of AQY-BA-480 and AQY-BA-481, while it generally improved the performance of the other monitors.

#### 3.3.2. Nitrogen Dioxide

In the raw dataset, the ANCOVA model indicates that the effects of the meteorological variables on the measurement errors in the raw data of the AQY1 monitors varied by sensor. Table S5 contains the results of the ANCOVA for the dependent variable NO_2_ Raw—NO_2_ Hinton, which show that there are statistically significant interaction effects, resulting from the large sample sizes, between Device ID and each of Temp, RH, WD, and WS, although not all of them were deemed to be practically significant.

**Figure 3:**
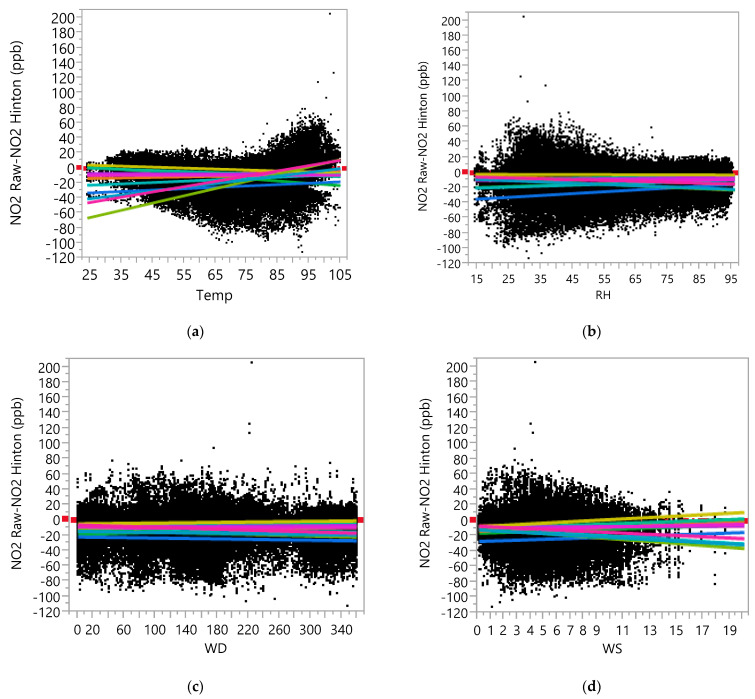

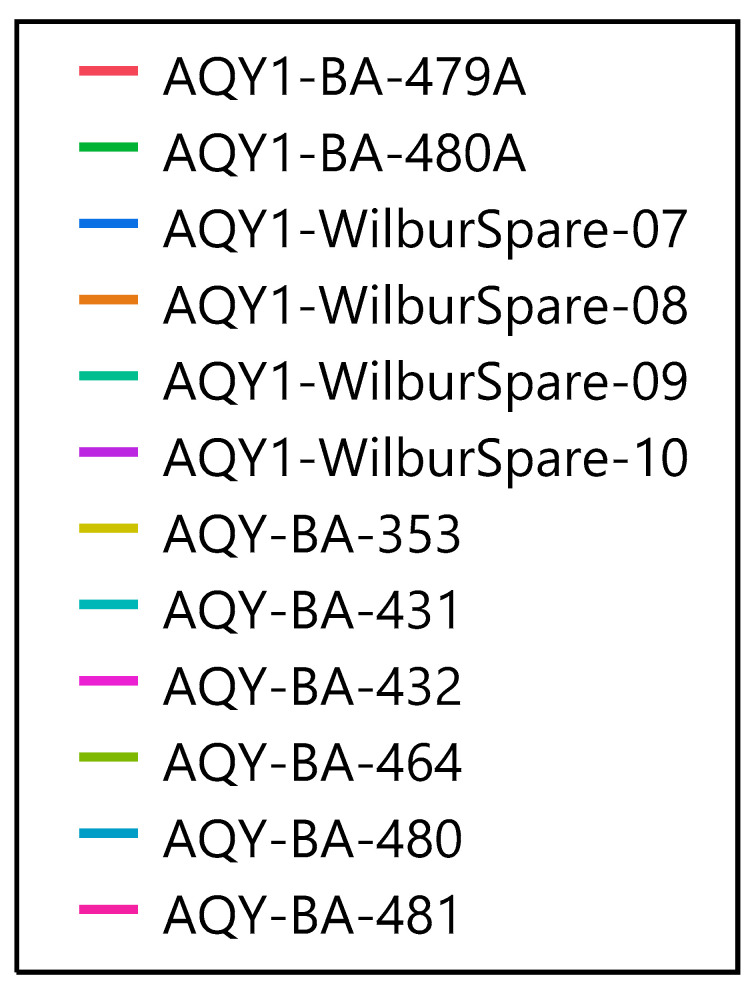
Regression Plot between NO_2_ Raw-NO_2_ Hinton Data versus (**a**) Temperature (**b**) RH, (**c**) Wind Direction, and (**d**) Wind Speed, as measured at the Hinton Reference Site.

Figure 3 presents regression plots based on the ANCOVA analysis results (provided in Supplementary Material), including each of Temp, RH, WD, and WD in the model, along with Device ID and the interaction between them. From Figure 3, it can be observed that the effect of Temp on the differences between the raw and the true NO_2_ data varies with Device ID. No consistent trend for over- or underestimating can be observed across the monitors (see Regression Plot in Figure 3a). The effect of RH on the differences appeared to be either negative (i.e., low-cost sensors underestimated NO_2_ concentrations as RH increases) or negligible (i.e., the slopes are close to zero) in general. The effect of WD on the differences seemed to be negligible (in terms of both the main effect of WD and the interaction effect between WD and Device ID, i.e., the slopes are close to zero). The effects of WS on the differences seemed to be different for each sensor (see Regression Plot in Figure 3d). As in the case of O_3_, the effects of meteorological variables (especially, the effects of Temp and WS) on the measurement errors of the AQY1 monitors were deemed to be different for the different sensors, so it may not be meaningful to assess effects of these variables without referring to Device ID.

We also conducted the same analysis using the calibrated dataset. The results from the calibrated data set are in the Supplementary Material and showed that, overall, the effects of the meteorological variables on the measurement errors in the calibrated NO_2_ data varied with Device ID. There was a suggestion that the measurement errors increased for some monitors as the WS increased. The results from the calibrated data set showed that overall, the effects of Temp, RH, WD, and WS on measurement errors were found to be smaller, in general, compared to those for the raw NO_2_ data. In the case of RH, there was evidence that the differences (NO_2_ Calibrated- NO_2_ Hinton) did not significantly vary with Device ID, except for AQY-BA-464. For AQY-BA-464, the monitor tended to overestimate at lower RH values and underestimate at higher RH values.

#### 3.3.3. Particulate Matter with a Diameter Less Than 2.5 μm

**Figure 4:**
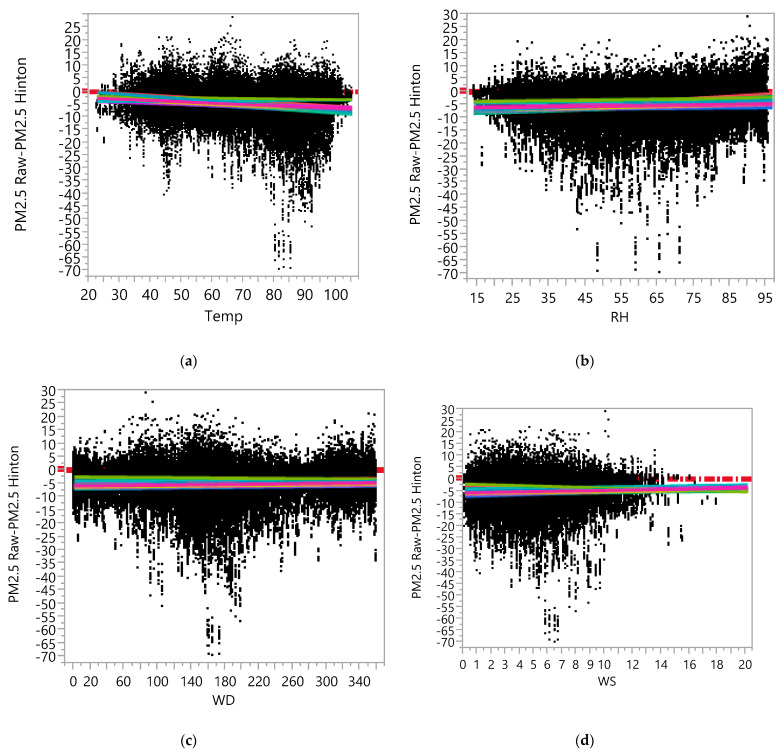

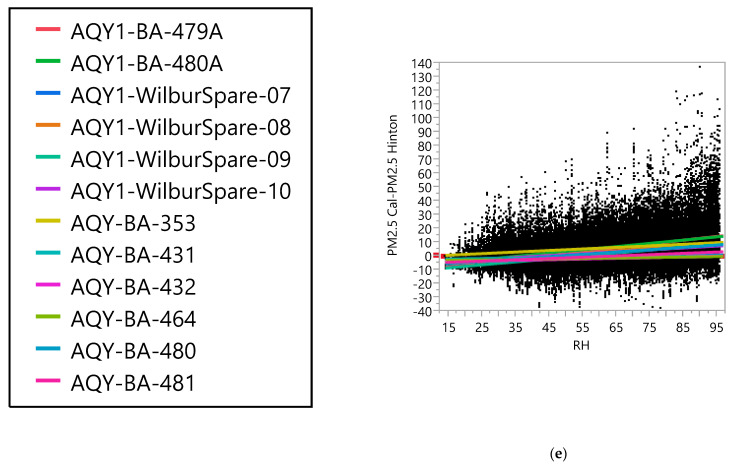
Regression Plot between PM_2.5_ Raw-PM_2.5_ Hinton Data versus the (**a**) Temperature (**b**) RH, (**c**) Wind Direction, (**d**) Wind Speed, (**e**) between PM_2.5_ Calibrated-PM_2.5_ Hinton Data versus the RH, as measured at the Hinton Reference Site.

In the raw dataset, the ANCOVA model, with measurement error (computed by PM_2.5_ Raw—PM_2.5_ Hinton) as a dependent variable, Device ID, Temp, RH, WD, and WS as the main effects and two-way interaction effects among them indicated that the effects of meteorological variables on the measurement errors in the raw data of the AQY1 monitors varied by sensor. Table S6 contains the results of the ANCOVA for the dependent variable PM_2.5_ Raw—PM_2.5_ Hinton, which show that there were statistically significant interaction effects (due to the large sample sizes) between Device ID and each of Temp, RH, WD, and WS, although none of those effects were deemed to be practically significant. Figure 4 contains regression plots based on the ANCOVA analysis results, including each of Temp, RH, WD, and WD in the model, along with the Device ID and the interaction between them, after removing 31 extreme outliers from the data (see Supplementary Material for underlying ANCOVA results for Figure 4). The results including the 31 outliers are not materially different, but are harder to see in an illustration. In general, the low-cost monitors seemed to underestimate PM_2.5_ concentrations on average (i.e., there seemed to be a negative bias). The bias did not seem to vary significantly over the range of meteorological variables. The variability (spread) of differences appeared to increase as the values of Temp or RH increased (i.e., the precision of the low-cost monitors seemed to decrease as Temp or RH increased).

The results from the calibrated data set (see Supplementary Material) showed that the bias of the low-cost monitors decreased (the regression lines are closer to the zero line) compared to the raw data, but that the precision decreased as well (the spread of PM_2.5_ Cal—PM_2.5_ Hinton increased). Figure 4e shows the regression plot based on the result of fitting the ANCOVA model with RH, Device ID, and an interaction effect between them based on the calibrated data. It can be observed that the spread in differences (PM_2.5_ Cal—PM_2.5_ Hinton) increased as the RH increased (i.e., precision of low-cost monitors seemed to decrease as the RH increased).

#### 3.3.4. Particulate Matter with a Diameter Less Than 10 μm

In the raw dataset, the ANCOVA model with measurement error (computed by PM_10_ Raw—PM_10_ Hinton) as a dependent variable, Device ID, Temp, RH, WS, and WD as main effects, and two-way interaction effects among them indicated that the effects of the meteorological variables (Temp, RH, and WS) on the measurement errors in the raw data of the AQY1 monitors varied by sensor. Table S6 contains the results of the ANCOVA for the dependent variable PM_10_ Raw—PM_10_ Hinton, which show that there were statistically significant (although not practically significant) interaction effects between Device ID and each of Temp, RH, and WS.

**Figure 5:**
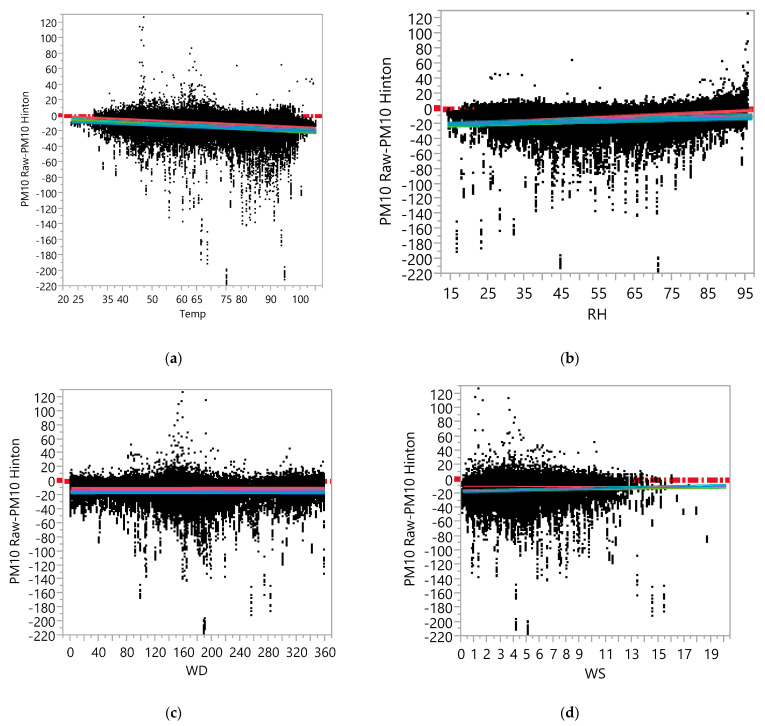

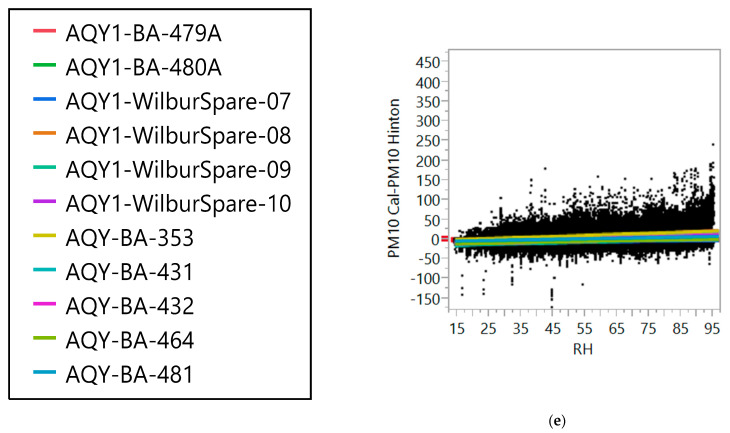
Regression Plot between PM_10_ Raw-PM_10_ Hinton Data versus (**a**) Temperature, (**b**) RH, (**c**) Wind Direction, (**d**) Wind Speed, (**e**) between PM_10_ Calibrated-PM_10_ Hinton Data versus the RH, as measured at the Hinton Reference Site.

Figure 5 contains regression plots based on the ANCOVA analysis results, including each of Temp, RH, and WS in the model along with Device ID, and an interaction between them after removing 69 extreme outliers. There were 31 extremely large outliers (with values greater than 210) and 38 extremely small outliers (with values less than −237). In general, the low-cost monitors seemed to underestimate PM_10_ concentrations on average (i.e., there seemed to be a negative bias), as in the case of PM_2.5_. The bias did not seem to vary significantly over the range of meteorological variables. It can be observed from Figure 5a,b that the spread of differences somewhat increased as the Temp or RH increased (i.e., precision of the low-cost monitors seemed to decrease as the Temp or RH increased).

The results from the calibrated data set (see Supplementary Material) showed that the bias of the low-cost monitors decreased (the regression lines are closer to the zero line) compared to the raw data, but that the precision decreased as well (the spread of PM_10_ Cal—PM_10_ Hinton increased), as in the case of PM_2.5_. A regression plot based on the result of fitting the ANCOVA model with RH, Device ID, and an interaction effect between them is shown in Figure 5e, and this demonstrates the increasing spread in PM_10_ Cal—PM_10_ Hinton (i.e., lower precision of lower-cost monitors) as RH increases more clearly than in the raw dataset.

### 3.4. Comparison Based on the Air Quality Index Categories

Table S8 shows a comparison of the AQI levels calculated using the calibrated AQY1 monitor data and the reference monitor data, separately for each pollutant. As shown in Table S8, all pollutant AQI categories, except for PM_2.5_, were correct over 95% of the time, when the reference monitor’s AQI level was ‘Good’. The PM_2.5_ data categorization matched 75.6% of the time when the reference monitor’s AQI level was ‘Good’ and 62.3% of the time when the reference monitor’s AQI level was ‘Moderate’. The O_3_ data also showed lower correlations in the ‘Moderate’ and ‘Unhealthy for Sensitive Groups’ AQI categories, but the number of observations in these categories was much lower than in the ‘Good’ AQI level, and as such more data are needed to confirm this observation.

## 4. Discussions

The need for low-cost air quality monitors arose in response to the fixed nature, high calibration requirements, and purchase and maintenance costs of traditional air pollution monitors, as well as the high spatial variability of urban air pollution, which is not captured by reference monitors. Despite the proliferation of low-cost sensors and their data, there is still a lack of clarity and inconsistency about how these perform in comparison to regulatory monitors, and how their performance might be affected by meteorological factors. The literature calls for further research in this area, in real-world conditions, as opposed to in laboratories, and over long periods of time that include realistic ranges of air pollutant concentrations and meteorological conditions. This study responded to this call and adds to a growing body of evidence assessing the performance of low-cost air quality sensors.

In this study, our collected data spanned a realistic range of pollution concentrations and meteorological variables; over a period of 18 months, captured by 12 sensors from the same manufacturer and of the same type (model), and operated at the same location. The assessment of the performance of the AQY1 low-cost sensors used here is an important factor, as researchers and practitioners determine their overall usefulness in specific research projects and for specific applications. Overall, our findings showed that the performance of the AQY1 monitors varied greatly by device and pollutant, and to a minor extent, was affected by temperature, relative humidity, and wind speed, as will be discussed next. The AQY1 sensors seemed to perform best when measuring O_3_ (e.g., R^2^ from 0.36 to 0.97, and this generally improved with calibration), followed by PM_10_ (e.g., R^2^ from 0.36 to 0.54, with mixed results after the calibration), while they performed poorly when measuring NO_2_ (e.g., R^2^ from 0.00 to 0.58, with mixed results after the calibration) and PM_2.5_ (e.g., R^2^ from 0.20 to 0.39, with mixed results after the calibration, and generally deteriorating performance). Studies that specifically investigated the sensors manufactured by Aeroqual (AQY) in the past also suggested that the best performance was for O_3_, followed by PM_2.5_ and PM_10_, and lastly NO_2_ [33,34,35], in line with our findings. We can only comment on this specific low-cost sensor type and expect different performances for different sensors, as shown in Table S1.

The wide range of R^2^ and varying performance in our study is also shown in the literature. As shown in Table S1, the degree of accuracy against reference-grade or regulatory monitor readings was highly variable (large ranges for e.g., R^2^) and is not directly comparable from study to study, due to the different pollutants, concentration ranges, sensor types, field location, and context-specific factors, such as meteorological conditions and calibration methods. The performance even varied from unit to unit of the same make, and this high heterogeneity is problematic when interpreting and comparing findings across the body of evidence. This finding was replicated in our study and in more recent studies, which suggested that the calibration models improve when individual sensor performance is accounted for.

In this study, we used a simple linear regression calibration method, which improved the performance of the low-costs sensors across certain parameters but not others. We explored the performance difference before and after calibration by inspecting the slope of the estimated regression line, the intercept, the coefficient of variation, and the RMSE. Overall, the reviewed studies found that data calibration improved the performance of low-cost sensors [19], but sometimes only certain performance parameters were inspected and reported and not others. Our inspection of different parameters suggests that the picture is mixed, and for PM, general deteriorations in performance were seen, which may be partly because of the set-up of the site (discussed next). Our calibration method, however, was also basic. Some novel calibration methods, such as the artificial neural network (AAN) calibration used in Spinelle, Gerboles, Villani, Aleixandre, and Bonavitacola [36] and Spinelle, Gerboles, Villani, Aleixandre, and Bonavitacola [37] further lowered the bias and seemed to help solve cross-sensitivity issues from which a major part of sensors suffer, such as when measuring O_3_ and NO_2_. While beyond the scope of our study, future research could investigate the difference in performance when calibration is conducted using linear regression versus more complex methods, such as multi-variate linear regression (MLR), machine learning and artificial neural network techniques, and other novel methods such as segmented model and residual treatment calibration (SMART) (see Table S1).

A particular issue was with the many negative raw NO_2_ values which the sensors reported (76,046 records or 46%), which were corrected in the calibrated datasets but poorly impacted on the calibrated values and the agreement between the calibrated and the reference monitors’ data. It is, however, important to note that the AQY1 monitors do not directly measure NO_2_. Instead, NO_2_ data is calculated based on the difference between the O_3_ and O_x_ sensors in the monitors, using the equation NO_2_ = O_x_ − 1.1 × O_3_, as per the manufacturer [31]. These results highlight the importance of recording and assessing both the raw and calibrated low-cost sensor measurements, as the added value of the calibration is complex and varies by pollutant and device. Owing to the logistical limitations at the Hinton site, the reference monitor’s instruments for PM_2.5_ and PM_10_ were mounted on a ground level cement pad, approximately 29 feet from the AQY1 monitors, and with a height difference of 7 feet 5 inches. We also think this difference in sampling location may have contributed to the worse performance in the PM assessment and perhaps the worse calibration results. We recommend that co-location is as exact as possible in future studies, but were not able to achieve this in the current study.

In ancillary analyses, some results showed that a good performance of a low-cost sensor in the laboratory is not indicative of a good performance in the real-world, and some authors suggested that it is necessary to perform a field calibration of each individual sensor and to do so periodically (at least once ~3 months) [6,13,38]. Some studies showed a gradual drift in the sensor readings as time passed. An example would be a drift to a lower PM concentration, which may be attributed to dust accumulating on a fan, reducing flow rate [15], and a drift towards higher O_3_ concentrations, which varied in magnitude depending on calibration [37]. We did not investigate these issues in the present study, as our focus was to assess and better understand the performance of the AQY1 sensors for a later application, but these are important issues, and resulted in weaknesses in our work.

The effects of meteorological data on performance were not uniform and the results again varied across different monitors. Measuring O_3_ seemed to be affected by temperature and RH, with a negative trend, except for a handful of sensors, while the effect of WS was more mixed. There was also an indication of an effect of Temp on the NO_2_ errors, which was more prominent in the calibrated data analysis (note, as above, that the calibration removed the many negative NO_2_ values). While the effect was mixed, for some monitors the error was higher (after calibration) at lower temperatures. The observation that the errors follow these trends of meteorological parameters is problematic, as NO_2_ is expected to be higher at lower temperatures, which could be associated with restricted atmospheric dispersion and/or to changes in traffic exhaust emission characteristics and emission source strength at low temperatures [39]. On the other hand, we expected O_3_ to be higher at higher temperatures, as it requires sunlight intensity and solar radiation to form, and higher temperatures may be indicative of more sunlight. As for PM, we observed a negative bias in general (i.e., the low-cost monitors seemed to underestimate PM_2.5_ concentrations on average).

Although the bias did not seem to vary significantly over the range of meteorological variables, the precision of the low-cost monitors seemed to decrease as the Temp or RH increased. There are no climate-controls on the low-cost sensors, while the reference monitor is climate-controlled (e.g., humidity control), and as such direct comparisons are challenging. As the AQY1 monitors measure PM using an optical particle counter and a light scattering method, humid conditions might have impacted the measurements. According to the AQY1 user guide, ‘light scattering is susceptible to humidity artefacts which over-report particulate levels due to ‘fogging’ where the particles are encapsulated by moisture and appear larger to the sensor than they actually are’. The AQY1 user guide mentioned that this effect is corrected for by way of a humidity correction algorithm; however, we still observed that the precision of the low-cost sensors decreased as RH increased; an effect which was more prominent in the calibrated datasets, and which may have been larger had there been no control for it by the manufacturer. Other studies [40] suggested an overestimation of particle concentrations when RH is high, potentially explained by the operational nature of optical particle counters and the detection and interpretation of water droplets as PM [17,20,41,42]. There are, however, studies showing negligible effects of meteorological variables on PM readings [10,16], and that the biases to RH and Temp varied across each sensor model and node; demonstrating that each sensor response is unique [10], as we found.

Overall, the time series patterns of pollutant concentrations measured by the AQY1 monitors followed the time series trends from the traditional reference monitor, although no formal analysis was conducted, and this deduction was based on a simple visual inspection of time series plots, such as those shown in Figures S3–S6, and others not shown here. Similarly to our deduction, other studies in air quality sensor performance evaluation [34], air quality sensor performance evaluation [35], and air quality sensor performance evaluation [33] showed that low-cost sensors seem to track diurnal variations well. As in the general performance of the AQY1 monitors, the time series from the low-cost sensors seemed to best follow the reference monitor’s data in the case of O_3_, NO_2_, and PMs. As such, although the absolute air pollution readings from the AQY1 monitors deviated from the reference monitors’ readings, the AQY1 monitor readings better tracked the reference monitors’ air-quality trends over time. Future research should formally assess this by conducting Granger causality and cointegration tests, and dynamic regression analyses. Other potential comparison analyses could be conducted using relative differences (instead of absolute differences) or machine learning tools, but this was outside the scope and the resources available for this study.

The strengths of our study are the long assessment duration, as compared to the literature (see Table S1); the assessment of 12 identical sensors, which elucidated the uniqueness of each of their responses; the assessment of four pollutant criteria, across both raw and calibrated data using an established and commonly employed co-location and calibration methodology; and the systematic investigation of the effects of meteorological variables on performance, inspecting both bias and precision to determine accuracy, and reporting these parameters.

The weaknesses of our study are the amount of missing NO_2_ data from the reference monitor and the negative value recordings, which are not practically sensible. Another limitation was the missing data from the AQY1 monitors, which was mainly due to sensor failure and the need to replace those sensors and reinstall the units in the field, and then manually upload the reference monitor’s data, recalculating the calibration factors and applying them. After a sensor had been replaced, the calibration factors were reset, and the co-location calibration had to be conducted again for the new sensor. There were two logistical and planning issues which increased the amount of missing data from the AQY 1 monitors: first, new sensors were shipped from overseas, which introduced some delay in the old sensors being replaced. The project researchers also needed to travel 180 miles to the measurement site for the replacement, which was complicated by COVID-19 protocols. These issues occurred frequently, and we learned the importance of having back-up sensors and a local, on the ground, contact for maintenance, where possible. We are applying these lessons in our next study phase. Our data also show that it is important for users to properly calibrate the low-cost sensors and continuously monitor the data once they are installed. Monitoring can lead to early detection of low-performing or faulty monitors, which can be replaced for better performance. While we did not investigate sensor drift in this particular study, research suggests that calibration must be conducted periodically, because the sensitivity of sensors changes over time [15,19,36,37]. This is a limitation of our study, which calibrated at the outset when sensors had collected a full month of data, at another intermediate point (when we expected to move the sensors in the field for another study), and when calibration was needed again, for example due to sensor failure and replacement.

Future studies should evaluate drift over time, and the frequency of re-calibration that is needed for optimal results, depending on that drift. We also recommend that calibration is done in real-world conditions, as laboratory calibrations may not be transferable to real-world applications [6,38], and on a set schedule, to be determined based on the drift. A good guide to thinking through issues of drift and regular re-calibration can be found in Williams, Kilaru, Snyder, Kaufman, Dye, Rutter, Russell, and Hafner [43]. Our study is limited in this regard, partly due to time and financial constraints, but we welcome collaboration and data exchange with other researchers to investigate these issues.

In terms of application, the deviations between the low-cost sensors’ readings and the reference monitor’s reading may be deemed as important, suggesting that there is still much work and developments needed to improve this emerging technology. However, the general tracking of diurnal patterns is promising. In addition, the low-costs sensors seemed to perform better if air pollution levels were binned in the AQI categories, rather than presented as absolute continuous numbers. Again, even when using categories, the performance was best for O_3_, and worst for PM_2.5_. More data are needed, especially at the higher AQI bins, which can be obtained by sampling for longer periods of times or in different locations, in order to better understand the agreement and disagreement in the different bin categories. Since the AQI is a calculated index value, which uses averaged concentrations over a specific period, the AQI comparison may provide an alternative framework for interpreting data from the low-cost sensors and improving their utility. The absolute low-cost sensor readings were not expected to match the reference monitor readings with the current state of technology. Therefore, using values such as the AQI levels for comparison allowed us to assess how the AQY1 monitors performed in categorizing air pollution levels in well-established categories, which have been used for public and stakeholder communication. This can be done after installing and calibrating the low-cost sensors and depending on the amount of data deemed appropriate before conducting a calibration, in addition to maintenance calibrations.

Overall, there seemed to be important deviations between the air pollution concentrations from the AQY1 low-cost sensors versus the reference monitor, which should be considered carefully in low-cost sensor applications. We also noted that the performance seemed to vary by device, indicating that no overall conclusion can be made. Based on our results, we do not recommend using the AQY1 monitors to report on absolute air pollution concentrations, or for comparing these measurements directly with measurements from a reference monitor, air quality guidelines (such as the World Health Organization’s), or to ascertain if air quality standards are being met (such as the U.S. Environmental Protection Agency’s), as the state of the technology has not developed sufficiently to accurately support this application. Low-cost sensors, however, allow broad deployment and the tracking of air quality trends, to compare air pollution levels, ideally binned into categories.

## Figures and Tables

**Table 1:** AQY1 and reference monitor instrumentation, pollutant ranges, and lower detectable limits.

AQY1 Units’ Instrumentation ^1^	Range	Lower Detectable Limit
PM_2.5_ (Optical Particle Counter using Laser Scattering)—includes a pump for active sampling	0–1000 µg/m^3^	1 µg/m^3^
PM_10_ (Optical Particle Counter using Laser Scattering)—includes a pump for active sampling	0–1000 µg/m^3^	1 µg/m^3^
O_3_ (Gas Sensitive Semiconductor)	0–200 ppb	1 ppb
NO_2_ (NO_2_ is reported as the difference between the O_x_ and O_3_ sensors according to the equation [NO_2_] = [O_x_] − 1.1 × [O_3_]. The O_x_ sensor is a Gas Sensitive Electrochemical sensor)	0–500 ppb	2 ppb
Reference Monitor Instrumentation	Range	Lower Detectable Limit
PM_2.5_ and PM_10_ (Beta Attenuation Mass Monitor 1020 ^2^)—active sampling	0–10,000 μg/m^3^	Less than 1.0 μg/m^3^
O_3_ (API Teledyne T400, UV Absorption O_3_ Analyzer ^3^)—active sampling	Min: 0–100 ppb full scale Max: 0–10,000 ppb full scale (selectable, dual-range supported)	<0.4 ppb
NO_2_ (API Teledyne T200E ^4^ Chemiluminescence NO/NO_2_/NO_x_ Analyzer)—active sampling	Min: 0–50 ppb full scale Max: 0–20,000 ppb full scale (selectable, dual-range supported)	<0.2 ppb

^1^ Information extracted from correspondence between the manufacturer and research team member JJ and confirmed via https://dozuki-prod-us-east-1-documents.s3.amazonaws.com/SPAHibXIfEj2AY4H.pdf#pdfjs.action=download (accessed on 29 November 2021); ^2^ https://metone.com/wp-content/uploads/2020/10/BAM-1020-N.pdf (accessed on 29 November 2021); ^3^ https://www.teledyne-api.com/products/oxygen-compound-instruments/t400 (accessed on 29 November 2021); ^4^ https://www.teledyne-api.com/products/nitrogen-compound-instruments/t200 (accessed on 29 January 2022).

**Table 2:** Descriptive (Summary) Statistics Comparison Between the AQY1 Monitors and the Reference (Hinton) Monitor. Results by Device ID may be more meaningful.

Ozone							
Data Set	O_3_ Raw AQY1 Data (ppb)	O_3_ Calibrated AQY1 Data (ppb)	O_3_ Reference Monitor (Hinton) Data (ppb)	O_3_ Reference Monitor (Hinton) Data—O_3_ Raw AQY1 Data (Absolute Difference)	O_3_ Reference Monitor (Hinton) Data—O_3_ Raw AQY1 Data (Difference in %)	O_3_ Reference Monitor (Hinton) Data—O_3_ Calibrated AQY1 Data (Absolute Difference)	O_3_ Reference Monitor (Hinton) Data—O_3_ Calibrated AQY1 Data (Difference in %)
Number of records	163,584	163,584	136,632	Not Applicable	Not Applicable	Not Applicable	Not Applicable
Missing records (%)	31,382 (19.2%)	74,322 (45%)	955 (7%)	Not Applicable	Not Applicable	Not Applicable	Not Applicable
Minimum	0	0	0	0	Not Applicable	0	Not Applicable
1st Quartile	23.5	19.6	16	−7.5	−47%	−3.4	−21%
Median	33.1	31.3	27	−6.1	−23%	−4.3	−16%
Mean	35	32.7	27.2	−7.8	−29%	−5.2	−19%
3rd Quartile	44.6	43.9	38	−6.6	−17%	−5.6	−15%
Maximum	121	138.7	85	−36	−42%	−53.7	−63%
**Nitrogen Dioxide**							
**Data Set**	**NO_2_ Raw AQY1 Data (ppb)**	**NO_2_ Calibrated AQY1 Data (ppb)**	**NO_2_ Reference Monitor (Hinton) Data (ppb)**	**NO_2_ Reference Monitor (Hinton) Data—NO_2_ Raw AQY1 Data (Absolute Difference)**	**NO_2_ Reference Monitor (Hinton) Data—NO_2_ Raw AQY1 Data (Difference in %)**	**NO_2_ Reference Monitor (Hinton) Data—NO_2_ Calibrated AQY1 Data (Absolute Difference)**	**NO_2_ Reference Monitor (Hinton) Data—NO_2_ Calibrated AQY1 Data (Difference in %)**
Number of records	163,584	163,584	136,632	Not Applicable	Not Applicable	Not Applicable	Not Applicable
Missing records (%)	31,382 (19.2%)	67,772 (41%)	3026 (22%)	Not Applicable	Not Applicable	Not Applicable	Not Applicable
Minimum	−109.0	0.0	0.0	109	Not Applicable	0.0	0%
1st Quartile	−11.0	0.0	2.8	13.8	493%	2.8	100%
Median	−2.4	0.0	4.8	7.2	150%	4.8	100%
Mean	−3.4	5.6	7.3	10.7	147%	1.7	23%
3rd Quartile	6.6	5.5	8.8	2.2	25%	3.3	38%
Maximum	208.5	110.9	45.7	−162.8	−356%	−65.2	−143%
**Particulate Matter with a Diameter Less than 2.5 μm**							
**Data Set**	**PM_2.5_ Raw AQY1 Data (ug/m^3^)**	**PM_2.5_ Calibrated AQY1 Data (ug/m^3^)**	**PM_2.5_ Reference Monitor (Hinton) Data (ug/m^3^)**	**PM_2.5_ Reference Monitor (Hinton) Data—NO_2_ Raw AQY1 Data (Absolute Difference)**	**PM_2.5_ Reference Monitor (Hinton) Data—NO_2_ Raw AQY1 Data (Difference in %)**	**PM_2.5_ Reference Monitor (Hinton) Data—NO_2_ Calibrated AQY1 Data (Absolute Difference)**	**PM_2.5_ Reference Monitor (Hinton) Data—NO_2_ Calibrated AQY1 Data (Difference in %)**
Number of records	163,584	163,584	136,632	Not Applicable	Not Applicable	Not Applicable	Not Applicable
Missing records (%)	24,677 (15%)	62,269 (38%)	241 (0.18%)	Not Applicable	Not Applicable	Not Applicable	Not Applicable
Minimum	0	0	0	0	Not Applicable	0	Not Applicable
1st Quartile	1.8	2.7	4.2	2.4	57%	1.5	36%
Median	3.1	7.7	8	4.9	61%	0.3	4%
Mean	4.3	11.2	9	4.7	52%	−2.2	−24%
3rd Quartile	5.3	15.6	12.2	6.9	57%	−3.4	−28%
Maximum	866.7	156.2	77	−789.7	−1026%	−79.2	−103%
**Particulate Matter with a Diameter Less than 10 μm**							
**Data Set**	**PM_10_ Raw AQY1 Data (ug/m^3^)**	**PM_10_ Calibrated AQY1 Data (ug/m^3^)**	**PM_10_ Reference Monitor (Hinton) Data (ug/m^3^)**	**PM_10_ Reference Monitor (Hinton) Data—NO_2_ Raw AQY1 Data (Absolute Difference)**	**PM_10_ Reference Monitor (Hinton) Data—NO_2_ Raw AQY1 Data (Difference in %)**	**PM_10_ Reference Monitor (Hinton) Data—NO_2_ Calibrated AQY1 Data (Absolute Difference)**	**PM_10_ Reference Monitor (Hinton) Data—NO_2_ Calibrated AQY1 Data (Difference in %)**
Number of records	163,584	163,584	136,632	Not Applicable	Not Applicable	Not Applicable	Not Applicable
Missing records (%)	34,883 (21%)	68,098 (42%)	321 (2.4%)	Not Applicable	Not Applicable	Not Applicable	Not Applicable
Minimum	0	0	0	0	Not Applicable	0	0%
1st Quartile	3.5	7	11	7.5	68%	4	36%
Median	5.6	17.5	18	12.4	69%	0.5	3%
Mean	7.24	23.14	20.83	13.59	65%	−2.31	−11%
3rd Quartile	8.7	31.9	27	18.3	68%	−4.8	−18%
Maximum	968.7	971.7	721	−247.7	−34%	−250.7	−35%

**Table 3:** Summary of Regression Analysis for O_3_, NO_2_, PM_2.5_, and PM_10_ Data from the AQY1 Monitors. Results are shown by device.

	y: Raw O_3_ Data					y: Calibrated O_3_ Data				
Device ID	b_0_	b_1_	R^2^	RMSE	n	b_0_	b_1_	R^2^	RMSE	n
AQY1-BA-479A	12.03	1.00	0.82	7.18	11,653	1.89	1.05	0.92	4.67	9561
AQY1-BA-480A	9.63	1.14	0.69	11.49	10,910	4.97	1.03	0.91	4.93	8819
AQY1-WilburSpare-07	6.31	0.81	0.73	7.40	9159	11.86	0.69	0.56	9.45	4341
AQY1-WilburSpare-08	11.96	0.80	0.83	5.54	10,854	2.96	0.91	0.93	3.98	5433
AQY1-WilburSpare-09	14.03	0.98	0.96	2.90	11,284	0.76	0.94	0.97	2.67	9914
AQY1-WilburSpare-10	12.85	0.77	0.87	4.54	11,563	1.81	1.09	0.93	4.45	9471
AQY-BA-353	2.49	1.11	0.93	4.42	9928	−0.58	1.08	0.94	4.02	4868
AQY-BA-431	9.98	0.99	0.77	8.31	11,485	5.67	1.04	0.87	6.35	9393
AQY-BA-432	12.57	1.13	0.64	12.92	11,312	5.78	1.00	0.89	5.35	9578
AQY-BA-464	13.29	0.40	0.59	5.05	6591	13.62	0.92	0.36	18.64	6041
AQY-BA-480	18.55	0.49	0.73	4.46	8835	7.71	1.57	0.86	9.13	5646
AQY-BA-481	14.10	0.60	0.77	4.90	9559	7.15	1.54	0.89	8.07	5646
	**y: Raw NO_2_ Data**					**y: Calibrated NO_2_ Data**				
**Device ID**	**b_0_**	**b_1_**	**R^2^**	**RMSE**	**n**	**b_0_**	**b_1_**	**R^2^**	**RMSE**	**n**
AQY1-BA-479A	−4.72	0.76	0.25	9.71	9912	−0.83	1.00	0.40	7.00	7821
AQY1-BA-480A	−12.96	0.95	0.18	15.38	8770	−0.99	0.72	0.43	6.80	6680
AQY1-WilburSpare-07	−19.69	0.45	0.02	20.30	7916	−0.29	0.05	0.14	1.01	4525
AQY1-WilburSpare-08	−7.58	0.57	0.19	7.59	8850	−0.92	0.76	0.29	8.82	4891
AQY1-WilburSpare-09	−4.60	0.72	0.29	8.47	9161	0.73	1.08	0.35	11.49	7817
AQY1-WilburSpare-10	−6.26	0.80	0.22	11.02	9912	−2.52	1.08	0.46	9.10	7821
AQY-BA-353	0.01	0.56	0.08	11.20	7953	−0.49	0.54	0.24	6.39	4295
AQY-BA-431	−14.64	1.02	0.19	15.80	9545	−1.14	0.75	0.30	9.09	7454
AQY-BA-432	−7.31	0.69	0.18	11.04	9172	−1.38	0.68	0.44	6.20	7401
AQY-BA-464	−14.28	0.66	0.02	27.48	5727	8.30	−0.05	0.00	12.84	5190
AQY-BA-480	−15.12	0.81	0.06	23.48	7462	−4.52	0.90	0.58	6.49	4010
AQY-BA-481	−7.02	0.47	0.02	22.47	8211	−3.26	0.67	0.53	5.32	4010
	**y: Raw PM_2.5_ Data**					**y: Calibrated PM_2.5_ Data**				
**Device ID**	**b_0_**	**b_1_**	**R^2^**	**RMSE**	**n**	**b_0_**	**b_1_**	**R^2^**	**RMSE**	**n**
AQY1-BA-479A	2.52	0.33	0.25	4.01	12,328	2.88	1.28	0.25	14.13	10,161
AQY1-BA-480A	2.15	0.30	0.28	3.37	12,685	3.36	1.33	0.30	12.88	10,518
AQY1-WilburSpare-07	1.07	0.17	0.41	1.51	9614	3.08	0.64	0.29	7.24	4691
AQY1-WilburSpare-08	1.63	0.24	0.32	2.46	12,642	2.61	1.08	0.30	11.28	7108
AQY1-WilburSpare-09	1.44	0.18	0.22	2.27	12,683	2.81	0.88	0.20	11.30	10,514
AQY1-WilburSpare-10	1.25	0.21	0.39	1.85	12,206	2.47	1.04	0.35	9.14	10,039
AQY-BA-353	1.32	0.23	0.34	2.20	12,663	4.14	1.20	0.36	10.78	7467
AQY-BA-431	2.23	0.35	0.36	3.12	12,131	0.85	0.84	0.38	6.50	10,323
AQY-BA-432	1.84	0.27	0.33	2.66	12,661	0.86	0.73	0.31	6.96	10,853
AQY-BA-464	2.98	0.41	0.31	4.63	6987	1.36	0.73	0.35	6.62	6435
AQY-BA-480	1.65	0.28	0.32	2.95	10,023	0.39	1.30	0.35	10.36	5726
AQY-BA-481	2.66	0.35	0.00	37.59	10,023	−0.44	1.08	0.39	7.86	5726
	**y: Raw PM_10_ Data**					**y: Calibrated PM_10_ Data**				
**Device ID**	**b_0_**	**b_1_**	**R^2^**	**RMSE**	**n**	**b_0_**	**b_1_**	**R^2^**	**RMSE**	**n**
AQY1-BA-479A	4.07	0.35	0.42	7.47	12,269	6.36	1.11	0.40	22.58	10,122
AQY1-BA-480A	2.13	0.25	0.49	4.47	12,596	5.99	1.03	0.40	21.09	10,469
AQY1-WilburSpare-07	0.86	0.20	0.56	3.13	9558	1.23	0.73	0.54	12.45	4655
AQY1-WilburSpare-08	2.23	0.23	0.50	4.18	12,573	1.95	1.08	0.53	18.78	7062
AQY1-WilburSpare-09	2.17	0.20	0.44	4.13	12,614	4.87	0.93	0.36	20.80	10,465
AQY1-WilburSpare-10	1.49	0.26	0.59	3.97	12,141	5.87	0.96	0.52	15.42	9994
AQY-BA-353	1.77	0.21	0.51	3.62	12,594	7.20	1.24	0.49	23.79	7420
AQY-BA-431	4.21	0.19	0.49	3.41	12,063	−2.21	0.87	0.38	18.62	10,274
AQY-BA-432	1.72	0.24	0.55	3.78	12,593	0.08	0.86	0.50	14.49	10,804
AQY-BA-464	0.71	0.25	0.63	3.57	6957	−2.23	0.92	0.54	14.18	6421
AQY-BA-480	NA	NA	NA	NA	NA	NA	NA	NA	NA	NA
AQY-BA-481	0.85	0.35	0.02	47.05	9964	7.93	0.76	0.46	10.53	5702

Notes: N (total number of hours in the study period) = 13,632; n denotes the number of non-missing measurements; measurements from the reference (Hinton) monitor were used as an independent variable (x) and measurements from low-cost monitors were used as a dependent variable (y); b_0_ and b_1_ denote the intercept and slope of the estimated regression line; RMSE represents the root mean square error for the regression line.

## Supplementary Materials

The following supporting information can be downloaded at: https://www.mdpi.com/article/10.3390/ijerph19031647/s1, Table S1 Literature Review Summary; Table S2 AQI Calculation Values; Table S3 Performance Statistics by Device and Pollutants across all AQY1 Units Examined; Underlying Regression Analysis Results for Calibrated Data; Underlying ANCOVA Results; (Tables S4, S5, S6 and S7); Figure S1. AQY1 Inlet Location Diagram; Figure S2. Location of Reference PM Monitors; Figure S3 O_3_ Data from the AQY1 Monitor AQY-BA-464 (Top Panel) vs. the Reference Monitor at Hinton (Lower Panel) for the Time Period 6 April–8 November 2019; Figure S4 NO_2_ Data from the AQY1 Monitor AQY-BA-464 (Top Panel) vs. the Reference Monitor at Hinton (Lower Panel) for the Time Period 6 April–8 November 2019; Figure S5 PM2.5 Data from the AQY1 Monitor AQY-BA-WilburSpare09 (Top Panel) vs. the Reference Monitor at Hinton (Lower Panel) for the Time Period 5–8 November 2019; Figure S6 PM10 Data from the AQY1 Monitor AQY-BA-WilburSpare09 (Top Panel) vs. the Reference Monitor at Hinton (Lower Panel) for the Time Period 5–8 November 2019; Table S8 AQY1 Percentage of Correct Observations by AQI and Pollutant [44,45,46,47,48,49,50,51].

## Abbreviations

AL: Ambient Light; ANCOVA: Analysis of Covariance; ANN: Artificial Neural Networks; API: Application Program Interface; AQI: Air Quality Index; AQ-SPEC: Air Quality Sensor Performance Evaluation Center; AQY: The Aeroqual micro air quality station; AQY1: The Aeroqual micro air quality station version 1; BAM: Beta Attenuation Monitors; CI: Confidence Interval; CO: Carbon Monoxide; CO_2_: Carbon Dioxide; COPD: Chronic Obstructive Pulmonary Disease; DP: Dew Point; EC: Elemental Carbon; EPA: Environmental Protection Agency; GEMM; Global Exposure Mortality Model; GSE: Gas Sensitive Electrochemical; GSS: Gas Sensitive Semiconductor; IHD: Ischemic Heart Disease; LR: Linear Regression; LRI: Lower Respiratory Infections; MAPE: Mean Average Percentage Error; MLR: Multivariate Linear Regression; Multi Linear Regression; NO: Nitrogen Oxide; NO_2_: Nitrogen Dioxide; O_3_: Ozone; P: Pressure (ambient); PM: Particulate Matter; PM_1_: Particulate Matter with a Diameter less than 1 μm; PM_10_: Particulate Matter with a Diameter less than 10 μm; PM_2.5_: Particulate Matter with a Diameter less than 2.5 μm; ppb: Parts Per Billion; QA/QC: Quality Assurance/Quality Control; RH: Relative Humidity; RMSE: Root Mean Squared Error; SMART: Segmented Model and Residual Treatment; SO_2_: Sulfur Dioxide; TCEQ: Texas Commission on Environmental Quality; Temp: Temperature; TTI: Texas A&M Transportation Institute; U.S.: United States; ug/m^3^ Microgram per cubic meter; UK: United Kingdom; USEPA: U.S. Environmental Protection Agency; WD: Wind Direction; WS: Wind Speed.
