# Supplementary material for: Evaluating the Performance of Low-Cost Air Quality Monitors in Dallas, Texas
Source: Int J Environ Res Public Health. 2022 Jan 31;19(3):1647. doi: 10.3390/ijerph19031647 (PMC8835131; doi:10.3390/ijerph19031647)
Supplement: Supplementary file 1 [file ijerph-19-01647-s001.zip › ijerph-1511898-SM revised.pdf]

Evaluating the Performance of Low-Cost Air Quality Monitors in Dallas, Texas: Supplementary Materials

Table S1 Literature Review Summary

Reference	Time Drift Discussion	Location	Low-Cost Air Quality Sensor(s)	Methods of Performance Assessment	Methods of Calibration Assessment	Pollutants and Parameters Studied	Temporal and Spatial Resolution and Coverage	Study and Assessment Duration<br>Dates	Results
[15]	Yes	Cuyama Valley, California, United States	Two separate model types of PM sensors (3 sensors each):<br>-AirBeam Optical Particle Counter ($PM_{2.5}$)<br>-Alphasense Optical Particle Counter (OPC-N2) (PM)	Sensors were co-located with the well-characterized reference instruments:<br>GRIMM 11-R optical particle counter. Used to derive values of PM_1, $PM_{2.5}$ and PM_{10}<br>FEM Met One beta attenuation monitor (BAM-1020) measuring PM_{10}<br>Precision among each sensors and accuracy against reference instruments' readings was evaluated using linear regression (LR) and coefficient of correlation (R^2)	Default conversion algorithm used for Airbeam to convert counts to $PM_{2.5}$ concentrations based on assumptions of density, refractive index and size distribution.<br>The OPC-N2 is calibrated by the manufacturer using polystyrene spherical latex particles with. known diameter, refractive index, and density.<br>No study-specific additional calibration factors were applied to the sensors.	PM_{10}, $PM_{2.5}$, PM_1<br>Relative humidity (RH), Temperature (T)	1-minute temporal resolution<br>All of the sensors were collocated within a few feet of one another. Reference instruments housed nearby in climate-controlled shelter.	12 weeks<br>14th April 2016 to 6th July 2016	This study was specifically interested in wind-blown dust events and regional transport.<br>Both sensor models demonstrated a high degree of co-located precision (intra-sensor variability) among each other (AirBeam R^2 = 0.95 – 0.99; OPC-N2, R^2 = 0.81 – 0.91). OPC-N2 correlations were lower than AirBeam because of variability inherent to wider PM range (1, 2.5, 10 um). Sensors demonstrated a moderate degree of accuracy against the reference instruments (R^2 = 0.6–0.76).<br>**Underestimation of concentrations by a factor of 2-4x was common for both sensors. Authors suggested that the sensors are better suited as a qualitative measure for high PM events.**<br>OPC-N2 displayed highest correlations with PM_{10}, with best performance in the PM_3 to PM_6 range, but was less accurate at sampling particles above $PM_{6.5}$.<br>**Sensor demonstrated a gradual drift towards lower number of PM measurements (~35%) and therefore concentration.** Could be attributed to dust accumulating on fan and reducing flow rate.<br>AirBeam had better performance for $PM_{2.5}$ compared to OPC-N2 and did not experience drift.<br>Sampling orientation, meteorological conditions, particle composition and size, and bin resolution (OPC) were declared to play roles in co-located precision among each sensor and their accuracy against reference measurements.

[49]	No	London Heathrow Airport, London, United Kingdom (UK)	Network of 17 sensors nodes containing the:<br>-Alphasense B4 (CO, NO, NO_2)<br>-Sensair K33 (CO_2)	One low-cost air quality sensor node was co-located with a reference site within the airport for accuracy comparisons. An additional sensor node was placed ~5m from the co-located sensor/reference pair above to explore reproducibility among the network sensors nodes. NO_2 was derived from O_3 measurements. The refence station only measured NO and NO_2. Sensor measurements validation for CO_2 was performed post study. Performance was evaluated using linear regression and correlation coefficients	Calibration of sensors was performed under laboratory conditions prior to deployment. No specific methods mentioned	CO, CO_2, NO, NO_2<br>Wind speed and direction	20 second temporal resolution converted to hourly averages for reference monitor comparison<br>Network of 17 sensors nodes placed in and around the airport covering approximately a 6 km^2 area designed to cover various activity zones	5 weeks<br>4th October 2011 to 11th November 2012	The authors suggested that the critical components of this study were the explicit separation of local and non-local emissions and the direct determination of emission indices using the sensor network coupled to appropriate analysis methods. This is an important demonstration of the potential of emerging low-cost air quality sensor technology and a relatively unique application.<br><u>NO_x</u><br>Co-located sensor with reference monitor had strong performance for NO_x (R^2 = 0.94, m = 0.96).<br>Two nearby sensor nodes had strong performance among them for NO and NO_2 (NO_2: R^2 = 0.94, m = 1.06; NO: R^2 = 0.96, m = 1.09) indicating repeatability among sensor nodes.<br><u>CO</u><br>Two nearby sensor nodes had good performance for CO (R^2 = 0.83, m = 0.93) indicating repeatability among sensor nodes.<br><u>CO_2</u><br>Co-located sensor with reference monitor had strong performance for CO_2 (R^2 = 0.92, m = 0.96).<br>Two nearby sensor nodes had good performance among them (R^2 = 0.71, m = 1.0)
[37]	Yes	European Monitoring and Evaluation Programme, Po valley, Italy	The cluster consisted of 5 NO_2 sensors and 2 CO sensors (electrochemical and metal oxide type), 1 NO and 2 O_3 electrochemical sensors, and 2 infrared CO_2 sensors.<br>-Alphasense: O3B4 (O_3)<br>-Citytech: O3_3E1F (O_3)<br>-Alphasense: NO2B4 (NO_2)<br>- Citytech: NO2_3E50 (NO_2)<br>-SGX Sensotech: MICS-2710 and MICS-4514-NO2 (NO_2)	Reference Measurement comparison:<br>O_3: a UV Photometric Analyzer Thermo Environment 49C<br>Nitrogen Oxides: a chemiluminescence Nitrogen Oxides Analyzer Thermo 42C for NO_2/NO/NOx<br>CO: a non-dispersive Infrared Gas-Filter Correlation Spectroscopy Horiba APMA 370<br>SO_2: a UV Fluorescent Analyzer Thermo 43C TL	Three calibration methods were tested: simple linear regression (LR), multivariate linear regression (MLR) and artificial neural networks (ANN) with raw, standardized (scaled) and calibrated sensor responses. Calibration equations were developed on a subset of the full dataset and then subsequently re-applied to predict pollutant	O_3, NO_2, CO, CO_2<br>T, RH, Absolute Humidity (AH), Pressure (P), Wind speed and direction	100 Hz measurements averaged every minute<br>Hourly averages used for calibration	5 months<br>March 2014 to July 2014	**It was shown that the ANNs result both in a lower bias and lower unbiased RMSE than LR and MLR.**<br>Moreover, LR and MLR symbols fall generally outside the target circle in the target diagram shown in the original study, called efficiency score, evidencing RMSE up to 2-fold higher than the standard deviation of reference measurements.<br>Simple LR and MLR have shown to produce the highest measurement uncertainty. While ANN with MLR inputs needed reference data for calibration of most sensors, ANN

			- CairPol: CairClip NO2 (NO_2)<br>-Citytech: NO_3E100 (NO)<br>-Fiagro: TGS-5042 (CO)<br>-SGX Sensortech: MICS-4514-CO (CO)<br>-Edinburgh Sensors: Gascard NG (CO_2)<br>-ELT Sensors: S-100 (CO_2)	CO_2: a differential Non-dispersive Infrared Gas Analyzer Li-cor 6262<br>The evaluation of sensor performances considered hourly values. It was carried out using only values predicted by each calibration method. For each one, regression and difference-based analysis were conducted to evaluate their performance. These included the calculation of the coefficient of determination (R^2), comparing the slope and intercept of the regression line with the objective values of 1 and 0, respectively. The mean bias error (MBE) and the root mean squared error (RMSE) standardized with the standard deviation of the reference measurements were used to draw a target diagram<br><br>The drift over time was also examined by plotting time series of the daily residuals between reference measurements and sensor predictions	concentrations on the remaining dataset				with raw/scaled data, using only 3 sensors of different types (1 O_3 chemical, 1 NO_2 resistive sensor and 1 CO electrochemical sensor), were able to solve the main interferences of the O_3 sensor.<br><br>**The ANN method increased the strength of association between estimated and reference data (higher R^2 and lower CRMSE). Moreover, it also allowed the decrease of the bias to reference data, with the slope and intercept of orthogonal regression being nearer to 1 and 0, respectively.**<br><br>The authors suggested that it is likely that by combining different type of sensors, like electrochemical O_3 and NO_2 MOx sensors, **the ANN can solve the cross-sensitivity issues from which suffers a major part of sensors.**<br><br>**The highest observed calibration drift during field tests consisted of 2.5% for NO/NO_2 and O_3, 4.5% for CO, 2% for SO_2 and 1.5% for CO_2.**<br>Time series plots showed that there is a positive trend towards high O_3, showing the effect of large slopes of the orthogonal regression and **giving evidence of a slight drift of the calibration methods over time of about 5 nmol/mol over nearly 4 months for ANNs, and about 20 nmol/mol for LR and MLR.** While the ANNs with the raw, scaled and MLR input results in similar drifts and constant noise, MLR showed slightly higher drift and noise than LR.
[36]	Yes	Po valley, Italy	Two types of CO sensors, one electrochemical and one metal oxide, one type of electrochemical NO sensor	Comparison with reference measurements:<br>The measuring campaign was performed at the European Joint Research Center−Ispra	Three calibration methods were tested: simple LR, MLR, ANN with raw, standardized (scaled) and	NO, CO, CO_2<br>T, RH, AH, P, Wind speed	100 Hz measurements averaged every minute<br>Hourly averages used for calibration	2 weeks (calibration period)<br>4 months (evaluation period)	**For NO and CO sensors, neither LR nor MLR performed well enough**.<br>**For CO sensors, the strength of association from calibration to validation decreases when applying**

			and two types of infrared CO_2 sensors were tested:<br>-Citytech: NO_3E100 (NO) → 2 sensors<br>-Figaro: TGS-5042 (CO) → 2 sensors<br>-e2V: MICS-4514 (CO) → 2 sensors<br>-EdinburghSensors: Gascard NG (CO_2) → 1 sensor<br>-ELT Sensors: S-100H (CO_2) → 2 sensors	station. The mobile laboratory was equipped with reference analyzers, meteorological and low-cost sensors<br>O_3: a UV Photometric Analyzer Thermo Environment 49C<br>Nitrogen Oxides: a chemiluminescence Nitrogen Oxides Analyzer Thermo 42C for NO_2/NO/NOx<br>CO: a non-dispersive Infrared Gas-Filter Correlation Spectroscopy Horiba APMA 370<br>CO_2: a differential Non-dispersive Infrared Gas Analyzer Li-cor 6262<br><br>The evaluation of sensor performances only relied on hourly average values. It was carried out using only values predicted by each calibration method. For each one, regression and difference-based analysis were conducted to evaluate their performance. These included the calculation of the coefficient of determination (R^2), comparing the slope and intercept of the regression line with the objective values of 1 and 0, respectively, and using the MBE and RMSE standardized with the standard deviation of the reference measurements to draw a target diagram<br>The drift over time was also examined by plotting time series of the daily residuals between reference measurements and sensor predictions	calibrated sensor responses			March 2014 to July 2014	**either LR or MLR.** The MOx sensors particularly show a radical drop of 90% (calibration R^2 = 0.76 and validation R^2 = 0.035)<br><br>CO and NO levels observed during the field experiment were very low. **This especially affects the extrapolation of the calibration model outside the calibration range**, resulting in a poor correlation between reference and sensors measurements<br>Based on the measurement uncertainty estimated by orthogonal regressions of the sensor outputs versus reference data, the most suitable calibration method appeared to be ANN using raw, MLR or scaled sensor inputs (lowest relative expanded uncertainty of 70% for NO, 30% for CO and 5% for CO2).<br>In all cases, simple LR and MLR have shown to produce the highest measurement uncertainty likely due to the fact that these methods do not take into consideration all interfering factors with their weighted effect.<br>For NO and CO, ANN models decrease the noise. This reduction reaches a factor 10 for CO and a maximum of 100 comparing ANNs to LR and MLR calibration.<br>**ANNs also seem to be able to slightly correct the drift over time of CO** sensors with about 0.05 µmol/mol over four months against 0.25 µ mol/mol for LR and MLR models over the same period. **NO sensors appear to be free from drift over time**, apart from one NO-3E100 sensor with MLR calibration.
[45]	Yes, discussed but not evaluated because campaign was only	Aveiro, Portugal	The different sensor-systems that were installed side-by-side with the reference analyzers (standard equipment) in the field are based on optical particle	A total of 15 participating teams from 12 countries participated in the campaign and installed 130 microsensors on the Air Quality Mobile Laboratory	No additional calibration applied.<br>In the Aristotle university - ISAG-microsensor box, data was collected on an SD card every five minutes	CO, NO_2, O_3, $PM_{2.5}$, PM_{10}, SO_2, Volatile Organic Compounds (VOCs)	Four temporal sampling frequencies were used by research groups participating in the project:	2 weeks<br>13th October 2014 to 27th October 2014	The overall performance of the sensors in terms of their statistical metrics and measurement profile **indicated significant differences in results depending on the platform**

	performed over two weeks		counters (OPC), metal oxide semiconductor sensors (MOS), electro- chemical sensors (EC), nondispersive infrared sensors (NDIR) and photoionization detection sensors (PID).<br><br>For PM measurements the sensor-systems measured particle counts based on light scattering.<br><br>Sensor models:<br><br>-Cambridge university SNAQ (Sensor Networks for Air Quality) box<br><br>-Aristotle university - ISAG-microsensor box<br><br>-ECN: airbox<br><br>-NILU + Envira: NanoEnvi platform<br><br>-IDAEA-CSIC: AQMesh node<br><br>-ENEA/air-sensor box<br><br>-VITO: EveryAware SensorBox<br><br>-UCL/CCMOSS MOS micro-hotplate for relative humidity sensing<br><br>-3S – OdorCheckerOutdoor<br><br>-Siemens AG – Ga_2O_3 based microhotplate sensor	(supplied with standard equipment and reference analyzers for continuous measurement of atmospheric pollutant concentrations and meteorological parameters) to monitor various parameters (atmospheric pollutants and meteorological variables) using different measuring principles.<br><br>Some of the sensors failed during the exercise and the results of others will be used for additional research, thus 27 of the sensors deployed were included in this study.<br><br>The comparison of the sensor data generated by different microsensor-systems installed side-by-side with reference analyzers, contributes to the assessment of the performance and the accuracy of microsensor-systems in a real-world context, and supports their calibration and further development.	and was corrected based on the empirical calibration curves provided by the sensor manufacturers.	T, RH, P, Wind speed and direction, solar radiation, precipitation	1-min<br><br>5-min<br><br>15-min<br><br>1-hr		**and sensors considered and the pollutant studied**.<br><br>**By pollutant**, the following results were observed: O_3 (R^2 = 0.12-0.77), CO (R^2 = 0.53-0.87), and NO_2 (R^2 = 0.02-0.89).<br><br>For PM (R^2 = 0.07-0.36) and SO_2 (R^2 = 0.09-0.20) the results showed a poor performance with low correlation coefficients between the reference and sensor measurements.<br><br>Results by pollutant are summarized below.<br><br><u>PM - particulate matter</u><br><br>PM_{10} → Results show a **poor correlation between the reference and the available measurements, with R^2 = 0.36 being the maximum value achieved** for ECN_Box_38.<br><br>$PM_{2.5}$ → Results were only available for three platforms and were **poor with the best performance detected for the ECN_Box_38 with R^2 = 0.27**.<br><br><u>O_3 – Ozone</u><br><br>**High variability was detected, and the best results came from IDAEA/AQMesh and NanoEnvi platforms, both having the lowest CRMSE and MBE, and the higher R^2 (above 0.7)**…ENEA/AirSensorBox, ISAG and CAM_11 platforms demonstrate poor correlations coefficients, with **R^2 values below 0.2**, while they also present higher CRMSE and MBE, with ISAG being by far the worst (MBE~350)”<br><br><u>SO_2 - Sulphur dioxide</u><br><br>There were only two measurement sets available for SO_2, coming from ENEA/AirSensorBox and CAM_11, both demonstrating **poor performance of (R^2 = 0.09 – 0.20)**.<br><br><u>NO_2 - Nitrogen dioxide</u>

									Six sensor platforms were compared; the higher correlations and lowest bias are obtained for IDAEA/AQMesh, ECN_Box_10 and CAM_11 with $R^2 > 0.8$ and MBE close to zero. The NanoEnvi platform demonstrates a good correlation of 0.6, but the ENEA/AirSensorBox and ISAG platforms exhibit a poor correlation of below 0.1, **indicating high variability amongst sensors.**<br><br><u>CO - Carbon monoxide</u><br><br>Four platforms were compared and presented a **satisfactory correlation** ($R^2 > 0.5$). The CAM_11 and IDAEA/AQMesh had the highest correlations ($R^2 > 0.8$).<br><br><u>NO - Nitrogen monoxide</u><br><br>Two platforms were available, IDAEA/AQMesh and CAM_11. **Good correlation ($R^2 = 0.8$) and small bias observed for IDAEA/AQMesh sensor while results show a weak correlation with $R^2 = 0.3$ for CAM_11**.
[21]	No	Memphis, Tennessee, United States	A total of 17 PM sensor pods were deployed in a joint project conducted in the CitySpace project by the USEPA and the Shelby County Health Department.<br><br>PM was measured by an Alphasense OPC-N2 sensor.<br><br>Wind speed and wind direction were measured by an Airmar 110WX meteorology sensor.<br><br>Temperature and RH were measured by a Vaisala temperature and RH sensor.	Sensor pods were collocated with a federal equivalent method (FEM) tapered element oscillating microbalance (TEOM) monitor, which collected hourly $PM_{2.5}$ mass concentration measurements.	The data from each sensor pod that met the correlation criterion were calibrated to better reflect the TEOM concentrations based on the linear regressions of the hourly average $PM_{2.5}$ concentrations from the final collocation period (i.e., the sensor data were normalized using the slopes and intercepts from the regression analysis).<br><br>This calibration helped remove varying biases when comparing the sensor pods to each other (for example one sensor could exhibit a slope of 2 when compared to the TEOM, while another could exhibit a slope of 0.5). The calibrated sensor data were then compared to each other using the Pearson correlation to	$PM_{2.5}$<br><br>RH, T, Wind speed and direction	1-minute temporal resolution<br><br>Measurements averaged to hourly concentrations	6 months<br><br>October 2016 to March 2017	**Several sensor pods failed** during the long-term deployment, and when collocated with the reference TEOM monitor, only 6 of the 17 deployed sensor pods met the data quality objective of R^2 greater than 0.5…**While these six sensor pods had correlations that met the data quality metric, they also had wide ranges of slopes and intercepts when compared to the reference monitor**.<br><br>**The high failure rate was in part due to the periods of high and sustained humidity.**<br><br>**Sensor failure was evident during high RH events.**<br><br>An attempt to develop an improved linear regression including RH found that it was **not a significant predictor at a 0.05 confidence level.**

					examine how temporal trends agree between sites and coefficient of divergence to examine the difference in concentrations between sites.				
[10]	No	Riverside-Rubidoux, California, United States	The 12 $PM_{2.5}$ sensors evaluated in this paper are categorized as optical sensors:<br>-Shinyei: PM Evaluation Kit<br>-Alphasense: OPC-N2<br>-TSI: AirAssure<br>-Hanvon: N1<br>-Airboclab: Foobot<br>-Kaiterra: LaserEgg<br>-PurpleAir: PA-II<br>-HabitatMap: Air Beam 1<br>-SainSmart: Pure Morning P3<br>-IQAir: AirVisual Pro<br>-Uhoo: Uhoo<br>**-Aeroqual: AQY**	The performance of the selected PM sensors was evaluated based on their hourly averaged air pollution readings against 1-h FEM measurements of $PM_{2.5}$. A Met One Beta Attenuation Monitor (BAM), USEPA designated Class III FEM (EQPM-0308-170) for monitoring $PM_{2.5}$, was used to compare against the low-cost sensor measurements. The Met One BAM provides 1-hr average $PM_{2.5}$ concentrations.<br>Data averaged from the low-cost sensors at the 1-hr level were matched by date and time to the hourly FEM BAM $PM_{2.5}$ data. The one hour averaging of data reduced the noise associated with measurements at shorter time resolutions. Statistical analysis was conducted on the 1-hr time matched data to examine data completeness, intra-model variability, least-squares linear regression statistics, measurement error, and impact of environmental conditions.	No calibration was performed, but authors reflection on the importance of calibration:<br>"For sensors that were highly correlated to the FEM BAM, the slope and intercept offsets of the regression statistics indicate that refinement or calibration of the sensors could be performed to improve sensor performance and reduce measurement error dominated by systematic error which could potentially be accounted for to reduce measurement error. The impacts of environmental conditions (RH and PM concentration) were investigated and indicate that the bias error for many low-cost optical particulate sensors on the market are impacted by changing environmental conditions."	$PM_{2.5}$<br>RH, T	Time resolution of each sensor is shown below although hourly averaged data was used averaged for comparison with FEM:<br>PM Evaluation Kit: 1-min<br>OPC-N2: <1-min<br>AirAssure: 5-min<br>N1: 1-min<br>Foobot: 5-min<br>LaserEgg: <1-min<br>PA-II: <1-min<br>Air Beam 1: 1-min<br>Pure Morning P3: <1-min<br>AirVisual Pro: <1-min<br>Uhoo: 1-min<br>AQY: 1-min	Study period:<br>3 years<br>5th February 2015 to 27th March 2018<br>Typical sensor deployment duration:<br>8 weeks<br>Sensors were evaluated in triplicate	Intra-model variability (within model variability)<br>Four sensors, namely **Aeroqual AQY**, Purple Air PA-II, SainSmart P3, and TSI Air Assure, **indicate low intra-model variability** with the standard deviation (SD) less than 0.75 with regards to the mean of means. Three sensors, namely the Laser Egg, Shinyei PM evaluation kit, and IQAir AirVisual Pro, indicate low to moderate intra-model variability with $0.76 \leq SD \leq 1.5$. Four sensors, namely Alphasense OPC-N2, Air Beam 1, Foobot, and Hanvon N1, indicate moderate to high intra-model variability with $1.51 \leq SD \leq 2.75$. The Uhoo indicates high intra-model variability with SD at±6.23.<br>Accuracy<br>Accuracy was defined as the degree to which the 1-hour average $PM_{2.5}$ concentrations recorded by the low-cost sensors conform to the FEM BAM instrument's $PM_{2.5}$ measurements, and it was evaluated by the slope and intercept values of the linear regression, in addition to the R^2 statistic.<br>Six of the 12 sensors were found to have a triplicate average of $R^2 \geq 0.70$ and will be discussed further with regards to slope/intercept for accuracy. Four sensors, namely **Aeroqual AQY,** Purple Air PA-II, Sainsmart P3, and the Shinyei PM Evaluation kit indicated **high linearity with $R^2 \geq 0.75$.** Two sensors, namely TSI Air Assure and Air Visual pro, indicated linearity with $0.70 \geq R^2 \geq 0.74$. With regards to slope as a measure for accuracy, four of these six sensors, namely Aeroqual AQY, Shinyei PM

									Evaluation kit, TSI Air Assure, and IQAir Air Visual Pro, were found to have slope values within±0.25 of the 1.0 ideal value. The Purple Air PA-II and the SainSmart P3 were found to generally overestimate FEM $PM_{2.5}$ concentrations by roughly 50% with slope values between 1.31 and 1.68. With regards to intercept value as a measure for accuracy, three sensors, namely the Sainsmart P3, Shinyei, and IAQir Air Visual Pro were found to have intercept values $\|b\|<2.5$ from the ideal 0.0 value. The remaining three sensors, namely the **Aeroqual AQY**, Purple Air PA-II, and TSI AirAssure, were found to have **higher intercept values ranging from $2.6<\|b\|<4.0$.**<br><br><u>Measurement error</u><br><br>Four sensors, namely the **Aeroqual AQY**, Kaiterra LaserEgg, Shinyei PM Kit, and IQAir AirVisual Pro, **have a Mean Absolute Error (MAE) near or less than 5 µg/m^3**. Five sensors, namely the Alphasense OPC, Air Beam 1, Purple Air PA-II, Sainsmart P3, and the TSI Air Assure, have MAE in the 5–7.5 µg/m^3 range. Three sensors, namely the Foobot, Hanvon N1, and the Uhoo, have MAE greater than 7.5 µg/m^3. For 8 of the 12 sensors, namely the **Aeroqual**, Foobot, Alphasense OPC, AirBeam 1, Hanvon N1, Purple Air PA-II, SainSmart P3, and TSI Air Assure, **the proportion of MBE to MAE is greater than 0.65 indicating that the predominant error associated with these sensors is systematic in nature rather than random**. Accounting for this systematic bias error could significantly reduce the measurement errors associated with low-cost sensors.<br><br>**The Aeroqual AQY bias error (triplicate average: 3.1 µg/^{3}) is strikingly close to the linear regression intercept values (triplicate average: 2.8 µg/m^3) indicating that the sensor may suffer from a zero offset and that**

									**correcting for this offset may reduce measurement error.**<br>Environmental conditions<br>The impact of relative humidity on sensor performance varied between models with **several models exhibiting increased bias error with increasing humidity**.<br>The bias error by RH plot for the **Aeroqual AQY**, TSI AirAssure, and the Shinyei indicate that these sensors are **not strongly impacted by increasing RH**. The remaining 9 sensors, except for the Uhoo, indicate increasingly **positive bias error as RH increases**.<br>Concentration range<br>No consistent trends are seen across the 12 sensors as $PM_{2.5}$ concentrations increase, but certain trends are observed for individual sensors.<br>Purple Air sensor indicates predominant random error between 0 and 12 $\mu g/m^3$ with scatter almost evenly distributed between positive and negative bias. Between 13 and 50 $\mu g/m^3$ the sensor indicates systematic positive bias error. **Aeroqual AQY indicates systematic negative error that increases as concentrations rise from 0 to 25 $\mu g/m^3$. Above 25 $\mu g/m^3$, the Aeroqual AQY bias is scattered around the y=0 line indicating random error.** The Shinyei PM kit, AirVisual Pro, and Laser Egg indicated measurement error dominated by **random error with scatter evenly distributed between positive and negative bias**.<br>**These observations are limited to the real-world $PM_{2.5}$ concentration range in the study area of 0–50 μg/m3. Sensors may behave differently outside this range**.
[44]	Yes	Los Angeles, California, United States	Purple Air PA-II sensors for measuring particulate matter and **AQY Micro Air Quality**	No specific performance assessment methods mentioned. Unknown number of Aeroqual and Purple Air	No calibration mentioned in this brief report.	$PM_{2.5}$, O_3, NO_2<br>T, RH, dew point	No temporal resolution mentioned.	Sensors installed December 2017<br>Duration unknown	**The AQY beta devices co-located with government monitoring stations exhibited strong**

			**Monitor (beta)** for measuring O_3, NO_2, and $PM_{2.5}$.	sensors co-located with government reference monitoring stations.			Network of 100 sensors placed in Los Angeles.		**correlations (O_3: R^2 = 0.97, NO_2: R^2 = 0.78, $PM_{2.5}$: R^2 = 0.76).**<br>**Minimal drift in AQY sensors of -0.33 ppb/month for O_3, 1.9ppb/month for NO_2, and -1.7 ppb/month for $PM_{2.5}$.**<br>**No mention of affects due to temperature or relative humidity.**
[33]	No	Rubidoux, California, United States	**3 Aeroqual (AQY Version 0.5) multi-sensor units**:<br>O_3 – Gas Sensitive Semiconductor (GSS)<br>NO_2 – Gas Sensitive Electrochemical (GSE)<br>$PM_{2.5}$ – Laser Particle Counter (LPC) (model SDS011 by Nova Fitness)	Aeroqual sensors were run side-by-side to the South Coast Air Quality Sensor Performance Evaluation Sensor (AQ-SPEC) reference instruments (FEM; Federal Reference Method, FRM):<br>O_3 instrument - FEM<br>NO_x instrument – FRM<br>$PM_{2.5}$ instruments – GRIMM FEM and Me tone BAM<br>Meteorological station: T, RH, P, Wind speed and direction	No sensor calibration was performed by AQ-SPEC prior to the field testing.<br>NO_2 data was corrected by Aeroqual using two approaches:<br>Correction for O_3 bias using AQY real-time O_3 data and Aeroqual algorithm<br>Correction for O_3 and RH bias using real-time O_3 data and RH (NO_2 V2)<br>$PM_{2.5}$ was corrected based on AQY real-time RH data. No further information mentioned.	O_3, NO_2 , $PM_{2.5}$<br>RH, T	1-minute temporal resolution<br>Averaged to 5-minute, 1-hour, 8-hour and 24-hour periods for comparison to reference monitors	12 weeks<br>22nd December 2017 to 27th March 2018	<u>O_3 – Ozone</u><br>**O_3 had excellent correlations (R^2 > 0.95) with low measurement variability observed between two units (one sensor left out due to failure). Tracked diurnal variations well.**<br><u>NO_2 – Nitrogen Dioxide</u><br>**NO_2 had moderate correlations (R^2~0.50) with substantial measurement variability between two units. Tracked diurnal variations. NO_2 V2 had good correlations (R^2 > 0.74), high accuracy and low measurement variability between two units. Tracked diurnal variations.**<br><u>$PM_{2.5}$ – Fine Particulate Matter</u><br>**$PM_{2.5}$ had very good correlation (GRIMM: R^2 > 0.84 and BAM: R^2 > 0.83), high accuracy and very low measurement variability between two units. Tacked diurnal variations. Corrected based on AQY RH data in real-time.**<br><u>Relative Humidity and Temperature</u><br>**Temperature and relative humidity <u>sensors</u> showed excellent correlation with the SCAQMD Met Station sensors (T: R^2 > 0.91 and RH: R^2 > 0.94). No speculation of their impact on performance. RH utilized to adjust $PM_{2.5}$ values in real-time.**
[34]	No	Rubidoux, California, United States	**3 Aeroqual (AQY Version 1.0) multi-sensor units:**<br>O_3 – GSS<br>NO_2 – GSE	Aeroqual sensors were placed side-by-side to the AQ-SPEC reference instruments (FEM; FRM):<br>O_3 instrument - FEM	No sensor calibration was performed by AQ-SPEC prior to the field testing.<br>Captured from Aeroqual Website:	O_3, NO_2 , $PM_{2.5}$<br>RH, T	1-minute temporal resolution<br>Averaged to 5-minute, 1-hour, 8-hour and 24-hour periods for comparison to reference monitors	12 weeks<br>20th February 2020 to 22nd April 2020	<u>O_3 – Ozone</u><br>**O_3 had excellent correlations (R^2 ~ 0.96, 5-min mean) with low intra-model variability (~2.9 ppb, 8.7%). Tracked diurnal variations well.**

			$PM_{2.5}$ – LPC (model SDS011 by Nova Fitness)<br><br>Differences from AQY v0.5 (reported in Air Quality Sensor Performance Evaluation (2018) above): Separate USB drive memory, new PCB board with sensor connector, real-time clock added, mounting bracket for O_3, NO_2, and $PM_{2.5}$ sensors	NO_x instrument – FRM<br><br>$PM_{2.5}$ instruments – GRIMM FEM and Teledyne APIT640-FEM<br><br>Meteorological station: T, RH, P, Wind speed and direction	$PM_{2.5}$ was corrected based on AQY RH data in real time. No further information mentioned.				**Slight overestimation of FEM measurements (m_{avg} = 0.97).**<br><br><u>NO_2 – Nitrogen Dioxide</u><br><br>**NO_2 had moderate to strong correlations (0.60 < R^2 < 0.78, 5-min) with low intra-model variability (~0.7 ppb, 6.7%). Tracked diurnal variations well. Overestimation of FRM measurements (m_{avg} = 0.71).**<br><br><u>$PM_{2.5}$ – Fine Particulate Matter</u><br><br>**$PM_{2.5}$ had very good correlation (GRIMM: R^2 ~ 0.78 and BAM: R^2 ~ 0.84 for the hourly means), high accuracy and very low intra-model variability (0.76 µg/m^3, 17.1%). Tacked diurnal variations. Underestimation of reference measurements. Correction based on AQY RH data in real-time.**<br><br><u>Relative Humidity and Temperature</u><br><br>**Temperature and relative humidity <u>sensors</u> showed excellent correlation with the Meteorological Station sensors (T: R^2 ~ 0.94 and RH: R^2 ~ 0.98). No speculation of their impact on performance. RH was utilized to adjust $PM_{2.5}$ values.**
[35]	No	Rubidoux, California, United States	**3 Aeroqual (AQY Version 1.0) multi-sensor units:**<br><br>PM_{10} – LPC (model SDS011 by Nova Fitness)<br><br>Differences from AQY v0.5 (reported in Air Quality Sensor Performance Evaluation (2018) above): Separate USB drive memory, new PCB board with sensor connector, real-time clock added	Aeroqual sensors were placed side-by-side to the AQ-SPEC reference instruments (FEM; FRM):<br><br>PM_{10} instruments – MetOne BAM and Teledyne API T640<br><br>Meteorological station: T, RH, P, Wind speed and direction	No sensor calibration was performed by AQ-SPEC prior to the field testing.	PM_{10}<br><br>RH, T	1-minute temporal resolution<br><br>Averaged to 5-minute, 1-hour and 24-hour periods for comparison to reference monitors	8 weeks<br><br>29th October 2020 to 24th December 2020	<u>PM_{10} – Coarse Particulate Matter</u><br><br>**PM_{10} showed weak to strong correlations with the FEM BAM and T640 (0.39 < R^2 < 0.49 and 0.60 < R^2 < 0.74 for FEM BAM and T640, respectively for the 1-hr mean) and underestimated the corresponding FEM BAM and T640 data.** Results differed for the 5-min and 24-hr means, where R^2 for the 5-min mean ranged between 0.56 and 0.68 while it ranged from 0.59 to 0.83 for the 24-hr mean for both instruments.<br><br>**The AQY sensors underestimated the PM_{10} mass concentration as measured by the reference monitors but seemed to track the diurnal variations well.**

									**Low intra-model variability (1.58 μg/m^3, 9.89%).**
[6]	Yes	Oslo, Norway	24 identical AQMesh v3.5 sensor nodes	The performance evaluation was conducted in both laboratory and field conditions. We will focus on the field conditions analysis.<br><br>The characterization of the AQMesh low-cost platforms included field testing against reference instruments, using the standard method of co-location, for a range of different environmental conditions (e.g., weather, traffic). The field tests were designed to identify additional errors which may be introduced as sensors are exposed to real-world conditions, which could not be tested in the laboratory arm of this study (not further discussed).<br><br>Between April and June 2015, 24 AQMesh nodes were co-located at the reference air quality monitoring station of Kirkeveien, Oslo, Norway. From July to September 2015, the nodes were distributed between four air quality monitoring stations in Oslo: Kirkeveien (10 units – allowing long-term evaluation performance), Manglerud (4 units), Åkebergveien (5 units) and Alnabru (4 units).<br><br>The comparison between the data collected by the AQMesh platforms and the reference instrumentation was based on widely used statistical measures including correlation r, the slope and intercept of the regression line, mean bias (MB), mean gross error (MGE), normalized mean bias (NMB), normalized mean gross error (NMGE), RMSE	Linear regression<br><br>Correlations between node and reference data were also significantly lower in the field than in the laboratory. The highest correlation was obtained for the NO sensor and was comparable to the one found in the laboratory. Linear calibration was applied with the slope and intercept obtained in the laboratory.<br><br>After performing a linear regression, the average RMSE of the 24 pods was reduced from 181 ppb to 87 ppb for CO, from 31 ppb to 10 ppb for NO, from 30 ppb to 9 ppb for NO_2, from 22 ppb to 3 ppb for O_3, from 19 ppb to 13 ppb for PM_{10} and from 6 ppb to 3 ppb for $PM_{2.5}$.	NO, NO_2, O_3, CO, $PM_{2.5}$, PM_{10}<br><br>T, RH, P	Standard AQMesh nodes deliver one-hour averaged data but can be configured to deliver 15 min averaged data	6 months<br><br>April 2015 to September 2015	**Performance varies from unit to unit (even though the units are identical), and also spatially, depending on atmospheric composition and meteorological composition**. Performing field calibration might help reduce measurement bias and error.<br><br>High correlations for all the gaseous pollutants in the laboratory ($r > 0.9$) when the sensors were tested under steady temperature and relative humidity conditions, while in the field the correlations were significantly lower. **Results clearly show that a good performance in the laboratory is not indicative of a good performance in the real-world.**<br><br>Results suggested that it is **necessary to perform a field calibration for each sensor individually. Calibration parameters might change over time depending on meteorological conditions and the location and this may result in under or over-estimating pollutant concentrations.**<br><br>Average r from field evaluations:<br><br>CO: 0.60<br><br>NO:0.86<br><br>NO_2: 0.49<br><br>O_3: 0.54<br><br>PM_{10}: 0.56<br><br>$PM_{2.5}$: 0.51<br><br>**Biases to relative humidity and temperature varied across each sensor node, thereby demonstrating that each sensor response is unique**. Performance for PM sensors also varied depending on locations in calm or heavily traffic areas, with busier areas reporting worse correlations. This may indicate an impact of the

									fresh exhaust composition on the readings (note that PM mass concentrations were estimated from particle counts).<br><br>The results show a clear change in behavior of the sensor platforms during the co-location period. This might be related to the sensors' detection limit and to the varying air composition and meteorological conditions. The performance for CO, NO and NO_2 worsened during the month of July and improved again in August and September….The monthly average values of the slope and intercept show that the month to month variation can be significant. This can lead to increased errors and biases that can pass unnoticed once sensor nodes are in the field.
[50]	Yes	Delhi National Capital Region, India<br><br>Two selected study sites:<br><br>Manav Rachna International Institute of Research and Studies (MRIU)<br><br>Faridabad, and Centre for Atmospheric Sciences, Indian Institute of Technology Delhi (IITD)	Custom-designed low-cost PM monitoring device (Atmos) featuring a Plantower PM sensor (model PMS7003) for measuring PM_1, $PM_{2.5}$, and PM_{10} concentration values by a laser-scattering technique. Included a DHT22 sensor for monitoring temperature and relative humidity.	This study evaluated un-calibrated measurements of the Atmos PM sensors in highly pollutant environmental conditions to identify their efficacy for PM_{10} measurements. The sensors were deployed in ambient field conditions onto the terrace of buildings with the inlets of the sensor and reference instruments in close proximity and under similar environmental conditions.<br><br>The reference instruments used were scanning mobility particle sizer (SMPS) in combination with either an optical particle sizer (OPS) or aerodynamic particle sizer (APS). Instruments used:<br><br>OPS™ Model 3330, TSI Inc. (for particles ranging from 0.3 µm to 10 µm) and APS™ Model 3321, TSI Inc., USA (for particles ranging from 0.5 µm to 20 µm). SMPS™ TSI Inc., consisting of an electrostatic classifier, Model 3082, connected to a condensation particle counter (CPC, model	Before field deployment, the sensors were co-located for a 2 ½ week period at the Indian Institute of Technology Kanpur (IITK) to test for consistency among the sensors (intra-sensor variability) under similar high PM conditions. The evaluation of the sensors was only conducted for PM_{10} concentrations, which were not calibrated due to a potential discrepancy in particle size, composition, and optical properties between field and laboratory conditions. Additional statistical measures were conducted do the presence of log-normally distributed data.	PM_{10}, $PM_{2.5}$<br><br>RH, T (measured, but not considered in analysis of this study)	1-10 second temporal resolution for low-cost PM sensor<br><br>5-minute temporal resolution for reference monitors<br><br>Measurements averaged to hourly concentrations	7 weeks<br><br>21st January 2018 to 16th March 2018	<u>Test for intra-sensor variability:</u><br><br>No significant variation or ambiguity was identified between the two PM sensors boxes. The coefficient of determination (R^2) was found to be 0.97, indicating strong correlations.<br><br><u>Performance</u><br><br>Overall mean hourly PM_{10} concentrations measured by the SMPS-OPS, Atmos and SMPS-APS monitors were 98.2 ± 65.5 µg/m^3, 149.2 ± 86.1 µg/m^3, and 74.4 ± 54.6 µg/m^3, respectively at the MRIU site and for the Atmos and SMPS-OPS monitors were 182.3 ± 84.2 µg/m^3 and 181.0 ± 111.5 µg/m^3, respectively at the IITD site. MAE ranged from 56.63 µg/m^3 to 68.74 µg/m^3. Mean hourly $PM_{2.5}$ concentrations measured by SMPS–OPS and Atmos were 117.31 ± 64.7 µg/m^3 and 161.70 ± 98.0 µg/m^3, respectively. Similarly, at MRIU the mean $PM_{2.5}$ concentrations measured by SMPS–OPS, SMPS–APS, and Atmos were 65.0 ± 51.3 µg/m^3, 72.3 ± 52.2 µg/m^3, and 139.1 ± 74.7 µg/m^3. **The Atmos sensors tracked the concentrations well for both PM_{10} and $PM_{2.5}$; however, they overestimated $PM_{2.5}$ concentrations with a constant**

				3776, TSI Inc., USA (from 14 nm to 760 nm particles).<br><br>Statistical analyses for performance evaluation were conducted in R Studio Packages and included mean, standard deviation, quantile-quantile (QQ-plot) formation, coefficient of determination (R^2), Pearson correlation (R), Spearman's correlation (R_s), mean bias error (MBE) mean absolute error (MAE), and scatterplots.					**offset, and PM_{10} concentrations were generally on the higher side.**<br><br>The agreement of the Atmos (Plantower PMS7003) with the SMPS–OPS and the SMPS–APS was observed to be moderate to high for different weeks from the seven-weeks-long field deployment (R^2 = 0.3–0.9), with most between 0.4 and 0.6.<br><br>**Data was observed to be log-normally distributed and positively skewed**, therefore additional statistical measures were conducted such as applying a natural log function. As a result, PM_{10} observed Pearson correlations (R) between the APS and OPS were 0.67 and 0.93 respectively. For $PM_{2.5}$, observed Pearson correlations (R) ranged from 0.86 to 0.94 across the two sites. Spearman's rank order correlations (R_s) were also conducted on the un-corrected (no natural log application) due to the presence of the non-normally distributed data. This resulted in strong correlations of R_s = 0.64 – 0.83. Intercept values ranged from 42.9 to 53.4 µg/m^3.<br><br><u>Drift, RH, and T</u><br><br>**No specific diminishing concentrations were observed in the pattern of the R^2 values between Atmos PM sensors and reference instruments; however, the authors stated that seven-weeks of data might not be sufficient to conclude the existence of drifts in the sensor measurements**. Temperature, humidity and aerosol refractive index were not included in the analysis of the study. The authors recommended a longer study duration and calibration of the sensors to explore the impacts of relative humidity and temperature.
[12]	No<br><br>(External-drift Kriging	Nantes, France	AtmoTrack Sensors	This study is slightly different as it investigated the potential added value of low-cost sensor data with respect to the dispersion model (ADMS-Urban) calculations for air	Before the mobile sensors were installed on vehicles, AtmoTrack sensors were placed next to the Air Pays de la Loire reference stations to be pre-	PM_{10}	10-second temporal resolution averaged to 15 minutes for reference monitor comparisons and 1 hour for data fusion model and spatial	1 month<br><br>November 2018	**Using density plots of the co-located low-cost sensors' readings and the readings from the reference monitors, distributions show similar mean and dispersion implying a consistency over the**

	data fusion applied)			quality mapping by applying a data fusion technique. This methodology consists in combining low-cost mobile and fixed sensor air quality data and dispersion model calculation to provide an estimation of pollutant concentration fields at the urban scale.<br><br>During the sampling period, AtmoTrack deployed 16 fixed sensors including 3 replicates at Victor Hugo station (traffic station) and 3 other replicates at La Bouteillerie station (urban background station). Most of the fixed sensors are in the city center excepted the sensor with the ID 10, which is in the west part of the city. 19 additional mobile sensors were on-board of driving school cars, ambulances and service vehicles to measure PM_{10} concentrations over numerous routes each day of the sampling period. The vehicle routes ensure a unique spatial coverage over the urban area.	calibrated before. No adjustment was made after the sensors were deployed.<br><br>The raw datasets from the low-cost sensors was preprocessed in two steps: 1) the elimination of unreliable data based on the repeatability criterion; 2) correction of the daily variation of the background concentrations.		interpolation (initial time resolution for the low-cost sensors is 10 seconds)<br><br>16 fixed sensors<br><br>19 mobile sensors<br><br>6-250m spatial resolution		**estimation domain of the pollutant measurements.**<br><br>Data fusion was performed on only one day in November: 29th. Hourly fused maps showed disparate responses to data fusion mainly depending on the variability of the sensor data and the correlation between the sensor observations and the drift. The data fusion performance was investigated by comparing daily average of the estimated concentrations, the reference observations and the hourly model outputs at each station of the Air Pays de la Loire network.<br><br>**Considering the model alone implies 8% bias whereas including the low-cost sensor observations reduces the bias to 2.5%.**<br><br>The concentration distributions related to the data fusion **had a lower dispersion than the reference observations and the model estimation**, where the PM_{10} peaks were smoothened.<br><br>The effect of the measurement uncertainty has been investigated by doubling it or reducing it to the reference station measurement uncertainty. The sensitivity study demonstrated that performance was increasing by reducing uncertainty. This highlights the importance to estimate accurately the measurement uncertainty of the devices to ensure relevant air quality mapping.<br><br>The method efficiency was limited by the low correlation between the sensor observations and the model used as external drift in the kriging. This may be explained by the remaining bias in data from the low-cost sensors. The better the correlation is, the lower the error related to the linear regression with the drift, and the better the spatial interpolation is.

[46]	No	USEPA Research Triangle Park; North Carolina, Happy Camp; California, Price and Dutch John; Utah, Springville; California, Pinehurst; California, Camp Nelson; California, United States	SenSevere Real-Time Affordable Multi-Pollutant monitor (RAMP).<br><br>**Aeroqual micro air quality station (AQY)**<br><br>The Purple Air PA-II-SD (PA).	Sensors were co-located with reference instruments at various locations in regular ambient, prescribed fire and wildfire conditions (100-mile radius).<br><br>Performance assessments were conducted using linear-least squares regressions and metrics utilized included the coefficient of determinations (R^2), MBE, MAE, RMSE, normalized root mean squared error (NRMSE) and average percent difference (PD_{avg}).<br><br>Linear multivariate regression was performed to determine impact of environmental parameters such as T, RH, wind speed and direction.<br><br>Reference instruments used: EDM 180 (GRIMM); E-BAM (Met One); E-SAMPLER; (Met One); BAM 1020 (Met One).	No additional calibration was used in this study besides the pre-installed calibration for each sensor by the manufacturer.<br><br>PA sensors reports two measurements due to a correction factor (CF) for indoor and outdoor applications.	$PM_{2.5,}$<br><br>RH, T, Wind speed and direction	Data was time-aligned with reference measurements and low-cost sensor measurements averaged on an hourly basis.<br><br>Sensors were co-located within 3-11 m of reference instruments and located 1-3 m above ground.	1 week – 8 months, depending on site<br><br>Long term testing of two sensors of each type for 2-8 months at the Research Triangle Park site, 8th August 2018 to 30th June 2019<br><br>1-6 weeks in locations below:<br><br>Happy Camp, 8/11–8/29/2018<br><br>Price and Dutch John, 9/24–10/1/2018<br><br>Springville, 10/19–11/27/2018<br><br>Pinehurst, 10/20–10/27/2018<br><br>Camp Nelson, 10/20–10/27/2018	Overall<br><br>Sensors hourly average r^2 = 0.52 - 0.95 but overpredicted concentrations (NRMSE: 80-167%)<br><br>**AQY (r^2 = 0.52 - 0.86): had the highest variations across sensors**<br><br>PA: highest correlation with the reference for all of the sensors evaluated (r^2 = 0.62 to 1.00, outdoor application setting; $r^{2=}$ 0.62 - 0.99 for indoor application setting)<br><br>RAMP (r^2 = 0.69 - 0.99)<br><br>Meteorological Conditions<br><br>All sensors recorded RH and T values which were strongly correlated with reference values in regular ambient conditions (R^2 = > 0.95), **with the AQY sensor having the highest performance. Correlations improved for AQY sensor when considering RH and T in correction factors, but not for PA and RAMP sensors.**<br><br>Ambient Conditions (AIRS site)<br><br>AQY and RAMP reported moderate to good correlations but with different slope values (0.89 and 0.88), which were significantly different from the reference, and MBEs of -0.01 and 1.64 ug/m^3, respectively.<br><br>**AQY sensor had the highest variation because one sensor reported values at half of others.**<br><br>PA reported good correlations but overreported values with an average slope of 1.62, which was significantly different from the reference, and MBE of 2.91 ug/m^3.<br><br>Smoke Impacted Conditions<br><br>All sensors reported moderate to good correlations with linear responses at concentrations up to 200 ug/m^3 but reported higher concentrations with an average slope of 1.27, indicating overestimation (AQY = 1.35, RAMP = 1.27, PA = 2.03).

									PA reported highest correlation values ($R^2 > 0.95$ for most sites) and during high concentrations. **AQY reported poorest correlations (R^2 = 0.52 – 0.77) but were still statistically significant**. RAMP reported lower concentrations (slope) and R^2 compared to PA (same underlying sensors).<br><br><u>Smoke Specific Correction</u><br><br>Each sensor received linear regression smoke correction factors which reduced NRSME to less than 27%. **AQY experienced the greatest improvement from a correction factor for environmental conditions (RH and T) with a NRMSE decrease from 31% to 25%. Correlation with the reference for the AQY increased when RH was included in the linear regression, but not for the PA and the RAMP.**
[19]	Yes	Official air quality monitoring station in Kirkeveien, Oslo, Norway	Three Inovafit low-cost PM sensor units (SDS011) with a DHT22 digital temperature and relative humidity sensor.	Sensor measurements for $PM_{2.5}$ were compared against data from a co-located TEOM 1405 FDMS reference-equivalent instrument.<br><br>Five performance aspects of the sensors were examined: operational data coverage, linearity of response and accuracy, intra-sensor variability, dependence on RH and T, and potential improvement of sensor accuracy by data calibration using a machine-learning method. Coefficient of determination (R^2), mean error (ME) and root-mean-square error (RMSE) were used to evaluate the linearity and accuracy of the data.	Simple multilinear regression (MLR) and a random forest (RF) model were used for correction of temperature and humidity effects.	$PM_{2.5}$<br><br>RH, T	One sensor: 30-second temporal resolution.<br><br>Two sensors: 2.5-minute temporal resolution.<br><br>Measurements averaged to hourly concentrations	Four months<br><br>11th December 2017 to 31st March 2018	Sensor operation time was stable during the four-month period and no obvious errors were observed. The three sensors also provided very similar results and inter-sensor correlations exhibited R^2 values higher than 0.97 and a variability around 9.64 %. All sensors demonstrated quite high linearity against officially measured concentrations of $PM_{2.5}$, with R^2 values of 0.55, 0.68 and 0.71, and slopes of 0.71, 0.82 and 0.89, indicating a general underestimation, particularly at higher concentrations. ME and RMSE were generally lower than 2 and 6 ug/m^3 respectively. **No significant qualitative drift of the signal was observed for any of the three sensor systems over the four-month study period.**<br><br>All sensors demonstrated similar patterns based on dependence to temperature. Temperatures less than - 5 ° C saw errors slightly negative or close to zero and temperatures around 0 ° C saw slightly positive errors between 0 and 5 ug/m^3. Error decreased again for higher temperatures and the peak error around 0 ° C was

									speculated to be due to higher RH values at that temperature. Errors because of relative humidity were stable between - 5 and 0 ug/m^3 for values less than 80 %, **but a substantial increase in error was observed for RH values greater than 80 %.** Positive average error values of 10 to 15 ug/m^3 were observed for values close to 100 %.<br>Multilinear regression somewhat improved the accuracy with respect to reference data and increases in R^2 values were relatively modest (0.02 to 0.05); however, **the random forest model increased the correlation significantly**, explaining roughly 10% more of the variability for two sensors and 20% more another sensor. **R^2 value increased from 0.71 to 0.80, from 0.68 to 0.79, and from 0.55 to 0.76 as a result of the random forest model calibration.**<br>The results demonstrated the general feasibility of using these low cost SDS011 sensors for indicative $PM_{2.5}$ monitoring under certain environmental conditions, and improvements in sensor accuracy can be achieved when relative humidity and temperature are accounted for as part of the calibration
[18]	Yes	Bologna, Italy	Optical sensors included:<br>Profiler Model 212 (MetOne Instruments, Inc., Grants Pass).<br>OPC-N2 low-cost sensors (Alphasense Ltd).<br>iSCAPE Citizen Kits (SCK, Fab Lab) low-cost sensor.<br>LOAC (light optical aerosol counter; MeteoModem).	This study had three aims: Characterize the performances and reproducibility of different brands of low sensors in comparison to reference instruments; assess instrument variability using batches of the same kind of low-cost sensors from the same producer; Perform a comparative analysis of the various optical particle counters (OPCs) under different meteorological conditions capable of sensibly affecting the PM size distribution, and consequently, the estimated mass concentration data.	No additional calibration was applied. Manufacturer calibration process was mentioned: The OPC-N2 and the SCK sensors estimates for PM_1, $PM_{2.5}$ and PM_{10} mass concentrations from count measurements use embedded proprietary algorithms, not yet disclosed to the public.<br>The LOAC sensor estimates for $PM_{2.5}$ and PM_{10} mass concentrations from count measurements. In general, the algorithms used by the optical sensors assume a default particle density	PM_{10}, $PM_{2.5}$, PM_1	Measurement frequency of the low-cost sensors ranged from 1 to 60 seconds.<br>Averaged to 1,10, 30, 60 minutes or 24 hours for evaluation.<br>Assessed seasonal variability. The study periods included a range of different meteorological conditions, representative of typical weather affecting the city and surrounding region in different seasons.	~6 months in total<br>6th June 2019 to 4th August 2019 and 23rd September 2019 to 12th February 2020.	**High bias at high time resolution and high RH. Performance improves when lowering the time resolution to hourly or daily averages**.<br>Low-cost sensors, and all OPCs, are affected by biases and low correlations when working at elevated time resolution.<br>Other biases that emerged are tightly connected with aerosol complexity, and as such, cannot be ignored, since the PM data might be seriously misleading if not considered and suitably corrected. Deviations were observed using flat density correction factors when converting particle number densities into mass, suggesting a

				To investigate and compare the performances of sensors in measuring particle size distributions and particle mass concentrations, they were co-located on the rooftop of the Department of Physics and Astronomy of the University of Bologna. Their measurements were compared to those obtained from the co-located MetOne instrument.	(1650 kg m^{-3} in the case of OPC-N2; unknown for the SCKs); a volume-weighting factor (default set to 1) to account for errors in sizing due to differences in the refractive index of particles used for calibration and those being measured; and for SCK, an atmospheric correction factor used in field evaluation whose details are not available from the manufacturer.				post field campaign reassessment and post-processing of data. This is especially important in countries/areas affected by mineral dust outbreaks whose properties and size distribution spectrum are significantly different from the urban background.<br>Performance of a sensor is highly impacted by the prevailing weather conditions, **suggesting particular caution in their use for estimating PM concentrations at high RH conditions, such as rain and fog events**. Conversely, their performances under conditions of weak synoptic forcing and prevailing anticyclonic conditions were in general characterized by low biases and elevated correlation coefficients.<br>R^2 with respect to the MetOne reference instrument, for three weeks ranged from 0 to 0.98, and depended on the size fraction investigated (Table 2 in the original paper).
[16]	No	Bay Area Air Quality Management District (AQMD)<br>Oakland, California, United States	Shinyei PPD42NS (PANDA: Portable and Affordable Nephelometric Data Acquisition).<br>Custom-built, potable, battery-operated aerosol monitoring instrument developed using low-cost, off-the-shelf optical aerosol sensors.	**Co-location with 2-m of the inlet of the Federal Equivalent Method (FEM) β-attenuation monitor (BAM-1020, Met One Instruments) that the Bay Area AQMD uses to monitor continuous $PM_{2.5}$ mass concentrations.**<br>The authors also deployed their own commercially available optical instruments at the regulatory monitoring site: a 16-channel particle sizer (GRIMM OPC, Model 1.108, GRIMM); a nephelometer (DustTrak II model 8530, TSI) equipped with a 2.5 µm impactor and programmed with the default correction factor for ISO 12103-1 A1; and a consumer-oriented, laser- based optical particle counter (DC1700, Dylos Corp).	Calibrated against the reference instrument which has an FEM status. The calibration was done using 24 h averages of $PM_{2.5}$ from the reference instrument.	$PM_{2.5}$<br>Ambient light (AL), RH, T	1 hour and 24-hour averages used for comparison.<br>Co-located within 2-m of reference instrument (regulatory monitoring site).	6 weeks (1 hr-concentrations)<br>15th April 2013 to 23rd April 2013<br>3 months (24-hr concentrations)<br>1st August 2013 to 15th November 2013	Using the 24 hours data resulted in better performance indicators than using the 1-hour data: **linear corrections were sufficient to explain 60 % of the variance in 1-hour reference $PM_{2.5}$ data and 72 % of the variance in 24-hour data**.<br>Shinyei PPD42NS with other optical instruments for hourly averages:<br>Individual PANDAs units against each other (R^2 = 0.91 – 0.92) – Low intra-sensor variability<br>PANDAs against BAM-1020 Reference (R^2 = 0.55 – 0.60)<br>PANDAs and the Dylos (R^2 = 0.87 – 0.92)<br>PANDAs and the GRIMM (R^2 = 0.90 – 0.94)<br>PANDAS and DustTrak (R^2 = 0.55 – 0.60)

				To quantify and compare the strengths of correlations, R^2 from ordinary least-squares regression models fit to each pairwise dataset was used. Empirical and simulated R^2 values were calculated for two BAM-1020s to provide perspective on the range of R^2 values expected with 1 h integration times. RMSE were computed to assess the accuracy of linear calibrations. Sensitivity analyses was designed to assess the effects of T, RH, and **ambient light** on instrument performance.					Variations in light, T, and RH had negligible effects.
[48]	No	Beijing, China	3 low-cost PM sensors:<br><br>Plantower PMSA003<br><br>Shinyei PPD42NS<br><br>NOVA SDS011	The sensors tracked $PM_{2.5}$ concentrations, which were compared to the field measurements at the national control monitoring station of the Ministry of Ecology and Environment at the same location.<br><br>Reference instruments:<br><br>GRIMM EDM 180: research grade, high precision instrument<br><br>Dylos DC1700: medium precision instrument<br><br>The measurements from the low-cost sensors and the reference instruments were compared in four ways and presented as such in the results section: (1) The 1-hour $PM_{2.5}$ mass concentration at Ministry of Ecology and Environment sites in Beijing with the results of 5 nearby sensors were compared, (2) 5 field tested PMSA003 sites and one Ministry of Ecology and Environment at a distance of 2 km, (3) The $PM_{2.5}$ hourly concentration data points were distributed, and (4) Impact of air humidity on sensor performance was investigated.	Calibration was not discussed, but results suggested to account for different issues in future tests based on their results.	$PM_{2.5}$, PM_{10}<br><br>RH, T	2-second temporal resolution.<br><br>Hourly averaged.<br><br>The experiment was divided in two stages: The first stage of study (evaluating the low-cost sensors): low-cost sensors were placed at the inlet of reference sensors. Second stage: low cost sensors which performed the best were deployed in the field in Beijing, and the multiple sensors were compared, also against the national control monitoring station of the Ministry of Ecology and Environment at the same location.	7 months<br><br>25th October 2019 to 10th June 2020 (Field Test)<br><br>1 week<br><br>8th February 2018 to 15th February 2018 (Plantower PMSA003, Shinyei PPD42NS, NOVA SDS011) (Evaluation)<br><br>3 weeks<br><br>8th February 2018 to 28th February 2018 (PMSA003)	Evaluation<br><br>Against the standard instruments, Plantower PMSA003 had the best fitting effect, with R^2 = 0.88~0.97 and RMSE = 0.02~0.09 after normalization, compared to Shinyei PPD42NS with R^2 = 0.76~0.86 and RMSE = 0.09~0.14, and NOVA SDS011 with R^2 = 0.85~0.89 and RMSE = 0.05~0.07. based on these results, the Plantower PMSA003 was selected for further evaluation and research.<br><br>Field<br><br>In the field, PMSA003 was very sensitive to most of the extreme concentration peaks. **The performance of the sensor became worse in the high-concentration range; as the difference between the sensor and the reference instrument increased**, and the **consistency among the sensors became worse**.<br><br>Overall, however, there is a good linear relationship between these sensors and reference instrument and also consistency among these sensors. The correlations between different PMSA003 sensors and the reference site equipment correspond to R^2 = 0.83~0.90, which **shows good consistency between these instruments**. The correlations among the five PMSA003 sensors

				Four parameters were used to evaluate the performance of the PM sensors: R^2, slope, intercept, RMSE, and percentage of relative bias.					also exhibit **high inter-sensor correlations** (R^2 = 0.91~0.98).<br><br>The environmental conditions of this study were variable, including variable concentrations and meteorological conditions. Results showed increasing underestimation when $PM_{2.5}$ concentration increased. **When the $PM_{2.5}$ concentration was <35 μg/m³, the relative bias was approximately 0%. The median relative bias reached −24.82% when the $PM_{2.5}$ concentration was >250 μg/m³.**<br><br>A small impact of high RH (>75%) was observed from raw PMSA003 data. PMSA003 significantly overestimated concentrations in high RH. The relative bias between PMSA003 and the reference instrument gradually increased with increasing RH. **When RH was 0%~60%, the median of the relative bias was below 0%. As RH increased, the relative bias gradually exceeded 0%. Median relative bias was 7.9% and 14.7% at 60%–75% and >75% RH, respectively**. This indicates overestimation of measured concentrations and high errors when RH > 60%. **PMSA003 performed poorly during sand and dust events**, especially when measuring ambient PM_{10}.
[38]	Yes	Minneapolis-Saint Paul metro area, Minnesota, United States	The researchers developed a wireless Mobile Autonomous Air Quality Sensor box (MAAQSbox) to measure air pollution. The MAAQSbox contains low-cost mobile air quality monitoring sensors. The device is autonomous and unique and holds 7 gas sensors and 2 particle sensors.<br><br>AlphaSense B4 (NO_2, NO, CO, O_3).<br><br>AlphaSense OPC-N2 (PM_1, $PM_{2.5}$, PM_{10}).<br><br>The MAAQSbox functions under various and extreme weather conditions and works well in an extreme	This study examined the quality of the low-cost, mobile air quality monitoring data by assessing the performance of MAAQSbox relative to Minnesota Pollution Control Agency stationary air monitoring regulatory equipment.	Both laboratory and field calibration were conducted.<br><br>Laboratory calibration: The calibration in the laboratory was conducted for CO, NO, and NO_2. The references were cylinders with certain known concentrations. The concentrations were calculated using the equation provided by the manufacturer.<br><br>When the calibration constants obtained in the laboratory were applied for outside	CO, NO, NO_2, O_3, $PM_{2.5}$, PM_{10}, PM_1.<br><br>Humidity, rain/moisture sensor.	1 Hz.<br><br>Hourly averaged to match the reference monitor.<br><br>MAAQSbox and the reference air monitoring station's inlet were ~ 30 cm apart, and at the same height facing the same direction.	September 2018<br><br>(154 hours)	MLR results for all sensors were improved by including T and RH as independent variables.<br><br>The R^2 of CO, NO, NO_2, and O_3 gas sensors are 0.96, 0.97, 0.81, and 0.95 respectively, while the R^2 of $PM_{2.5}$ particle sensor is 0.6.<br><br>The largest effect in RMSE due to inclusion of the T and humidity was in the NO sensor, where RMSE reduced from 8.1 to 3.4.<br><br>**B4 sensors were sensitive to ambient conditions such as temperature and RH.**<br><br>The results with OPC-N2 differs from the reference air monitoring station indicating further

			temperature range. It has a system to protect the sensors during rain or relatively RH.		measurements, there were reported instances of negative concentrations, as well as significant differences between the low-cost sensors and readings from the air monitoring station references. Based on these unsatisfactory results, an additional field calibration was conducted.<br><br>Field calibration: the calibration was conducted in the field to evaluate the low-cost sensors performance against the reference instrument, as well as the impacts of T, RH, and cross-sensitivities in calculating concentrations. The calibrations of the low-cost sensors were determined by MLR. A field calibration has been performed by making side by side measurements with the MAAQSbox and Minnesota Pollution Agency air monitoring station.<br><br>The sensors at the reference air monitoring station were:<br><br>Teledyne 190 T200 NOx for NO and NO_2<br><br>T300 for CO and T400 for O_3<br><br>The BAM 1020 (Metone) for $PM_{2.5}$				developments are needed to enable more accurate $PM_{2.5}$ measurements.<br><br><u>Calibration</u><br><br>**T and RH increase the R^2 of the all models**, and the authors concluded that they must be included in the calculation of concentrations.<br><br>CO: 0.946 to 0.956<br><br>NO: 0.835 to 0.971<br><br>NO_2: 0.742 to 0.804<br><br>O_3: 0.928 to 0.945<br><br>$PM_{.2.5}$: 0.542 to 0.593 (Temperature only). RH was not included in the model because its p-value was higher than 0.05.<br><br>The slopes are 0.99, 0.98, and 0.95 for CO, NO, and O_3 sensor respectively. The slope of NO_2 is 0.79.<br><br>Slope of OPC-N2 was 0.59, but the removal of the four highest concentrations improved it to 0.71<br><br>**The authors highly recommended calibration in the filed before conducting any measurements with low-cost sensors**, as the results show them to be affected by T and RH. **The calibration must be conducted periodically because the sensitivity of sensor changes over time ~3 months.**
[48]	No	Dongjak-gu, Seoul, South Korea	3 Plantower PMS7003 sensors.	A multi-sensor platform has been developed and co-located with the governmental regulatory BAM PM711 in the government station to evaluate low-cost light scattering $PM_{2.5}$ sensor.<br><br>A novel combined calibration method has been introduced to increase low-cost sensor	In this paper, calibration doesn't mean any correction for the observed data in the training dataset. The calibration means an estimation for the unseen data in the training dataset	$PM_{2.5}$<br><br>RH, T, AL	1-second temporal resolution.<br><br>5-minute sampling intervals were converted into 1-hour and 24-hour averages.<br><br>The three low-cost PM sensors are mounted on a single multi-sensor	15th January 2019 to 4th September 2019.	The comparison of the uncalibrated raw PM signal and **the calibrated PM signal expressed a significant improvement** (e.g., the non-linear MLP calibration reduced the MAE from 9.78 to 3.55 μg/m^3), and **the calibration, including the PM raw signal with humidity signal showed remarkable improvement** (e.g., the non-linear MLP calibration reduced MAE from 3.55 to 2.99 μg/m^3). **The**

				accuracy. The performance was compared to other calibration methods.<br><br>The performance of the low-cost PM sensor was analyzed using the following metrics: MAE, MSE, RMSE, R^2, slope, intercept, mean, standard deviation, and quartiles.	The authors developed a novel combined calibration algorithm. The algorithm selectively applies multiple calibration models and statistically reduces residuals, while using a prebuilt parameter lookup table where each cell records statistical parameters of each calibration model at current input parameters.<br><br>Three different calibration methods were evaluated (multivariate linear regression, non-linear, and segmented model and residual treatment (SMART) calibration).<br><br>The multivariate was based on humidity and temperature corrections, where these parameters were used as explanatory variables. Non-linear calibration was performed based on a multilayer perceptron (MLP) from the neural network and it consists of an input layer, an output layer, and hidden layers. The SMART calibration maps the most probabilistically appropriate models given multiple linear/non-linear calibration models and reduces residuals associated with the full range of explanatory variables.		platform to identify sample variation among three low-cost sensor samples.		**improvement was insignificant by including temperature and ambient light.**<br><br>**The SMART calibration method significantly improves the accuracy of the low-cost PM sensors** (e.g., RMSE: from 23.94 to 4.70 ug/m^3) and increases the correlation (e.g., R^2: from 0.41 to 0.89).<br><br>The means and standard deviations in the raw signal of the low-cost sensor and the governmental BAM outputs were 38.15 ± 31.29 and 23.10 ± 14.84 ug/m^3, with around 65% normalized mean bias error.<br><br>A comparison of calibration methods, such as MLR, MLP, and SMART calibration, was performed. The means and standard deviations in the SMART calibration of the low-cost sensor and the BAM output were 23.09± 13.85 and 23.01 ± 14.74 ug/m^3 with around 0.35%. normalized mean bias error. When the raw signal and calibrated signals of the low-cost sensor were compared to the figures from BAM output by applying correlation index, R^2, increased correlations between the low-cost sensor and the BAM output were observed as 0.41 (raw signal), 0.82 (LR), 0.84 (MLR), 0.83 (MLP), and 0.89 (SMART calibration). This calibration model was verified with the possibility of being applied to future datasets.<br><br>Authors conclude that **calibration is highly required when low-cost sensors are used for high accuracy sensing**.<br><br>Sample-to-sample variability of the low-cost sensors was evaluated among three co-located low-cost sensors. **The sensors were very strongly correlated having high correlation coefficients ranging from 0.985 to 0.997**.
[47]	No	Nablus in Northern West Bank, Palestine	Three low-cost particulate matter monitors (AirU's) utilizing the Plantower Particulate Matter Sensor	To assess the air quality in the city of Nablus during and outside of dust storms, at three different locations, by	The AirUs were calibrated for local PM using a Mini-Vol configured for $PM_{2.5}$ collection. The Mini-Vol is	PM_{10}, $PM_{2.5}$	"Near-real-time" 1-minute average concentrations.	18th March to 5th April and 20th May to 13th June of 2018.	PM concentrations were found to be highly variable and exceeded the WHO guidelines most of the time.

			(PMS) 3003, developed by the University of Utah College of Civil Engineering.	measuring the real-time PM concentrations using low-cost AirU sensors.<br><br>Pre-calibrated before deployment.	a filter-based, low-volume air sampler (Airmetrics Co., Inc.; 5 L/min flow rate). The instruments were co-located and operated for seven periods under differing ambient conditions. As the expected concentration range and particle composition across the chosen sample locations was unknown, the researchers judged it best to calibrate the sensors across differing local PM types. The periods ranged from one day to several days depending on expected PM concentrations, during which $PM_{2.5}$ ranged from 17 to 168 µg/m^3.<br><br>Owing to system and time limitations, PM_{10} was not calibrated directly through the Mini-Vol filters, but rather was calculated by multiplying the apparent PM_{10} from the AirUs with the ratio of calibrated-to-apparent $PM_{2.5}$ concentrations.		Averaged to daily concentrations.<br><br>To examine the spatial and temporal distribution of PM concentrations in the city of Nablus, three sites were selected at different elevations and differing local source areas throughout the city (apartment complex, near downtown area and valley bottom, private residence).		There were certain periods where PM concentrations were high at all three sites. The episodic higher concentration spikes at all locations could possibly be attributed to the occurrence of seasonal dust storms.<br><br>The authors discuss that when the linearity of the AirU systems shifted above 40 µg/m^3, a more reflective power curve fit was applied to the data obtained within this study. The manufacture's literature **suggests that changes in ambient T and RH may have a significant effect of the accuracy and precision of these low-cost, light scattering-based sensors.** However, the authors also highlight that the available literature of field evaluations showed **reported readings to be statistically impacted only when the RH was >75%.** The impacts from changes in temperature alone were negligible.<br><br>Average R^2 for the three sensors after the power curve application for calibration was 0.88.
[51]	Yes (speculated but not evaluated)	2015 Hong Kong Marathon<br><br>Hong Kong, China	Custom made mini air station (MAS).<br><br>The MAS contains two electrochemical sensors (NO_2-B4 and CO-B4, Alphasense), assembled on individual sensor boards supplied by the manufacturer. These sensors were used for NO_2 and CO gas measurements.<br><br>The second sensor was a photometer (ES-642, Metone) with a $PM_{2.5}$ cyclone inlet which was used for monitoring $PM_{2.5}$ concentration.<br><br>In addition to the NO_2, CO and $PM_{2.5}$ pollutants monitored by MAS, ozone was measured using portable ozone monitors	Both laboratory characterization and field evaluation of sensor and system performance were carried out prior to deployment.<br><br><u>Laboratory Performance</u><br><br>The laboratory performance test focused on three main components: (1) establishing the linearity and lower detection limit of the electrochemical sensors; (2) determining impacts of humidity and temperature on performance; and (3) calibration of ozone monitors with standard ozone gas.<br><br>For the first test component, NO_2 and CO sensors were set	The ozone monitor calibrations were conducted in the laboratory using O_3 gas generated from a calibration source (Model T700U, Teledyne) and flushed through the POM over a range of 0 to 300 ppb with steps of 50 ppb, each step lasted 10 minutes. These concentration steps were selected to cover the range of typical urban pollutant concentrations.<br><br>Light scattering-based PM photometers for PM concentrations were subjected to two tiers of corrections, including the	CO, O_3, NO_2, and $PM_{2.5}$<br><br>T, RH	Response time per pollutant:<br><br>NO_2: < 25 seconds<br><br>CO: < 15 seconds<br><br>$PM_{2.5}$: NA<br><br>O_3: 20 seconds<br><br>The sensor systems' raw data were transmitted in real time to the cloud server located at City University at 5 second intervals.<br><br>Averaged to 1-minute, 5-minute, 1-hour.<br><br>3 monitoring sites along the marathon route.	<u>Laboratory</u><br><br>Date and duration unknown<br><br><u>Field Test</u><br><br>52 hours<br><br>16th January 2015 to 18th January 2015<br><br><u>Marathon Deployment:</u><br><br>25th January 2015	<u>Laboratory performance test results</u><br><br>The NO_2 and CO sensors and the POM demonstrated high linearity of sensor response to the pollutants in the concentration range. R^2 of this correlation was > 0.99.<br><br>For temperature and RH tests, CO and NO_2 sensors showed different behaviors and responses with varying conditions. In the temperature and RH range, CO sensor output showed **no discernible variation at the same CO concentration**. For the NO_2 sensor, the change of **temperature showed little impact on sensor response**. But a positive relation was observed between **RH and the gain of the sensor signal**, possibly due to the humidity equilibrium

			(POM, 2B Technologies, Boulder, CO, USA). These monitors are compact UV-based ozone monitors.	up in a Teflon chamber, and two calibrated gas analyzers were used as references, i.e., the NO_2 analyzer (Model T500U, Teledyne, Thousand Oaks, CA, USA) and CO analyzer (Model T300U, Teledyne).<br><br>Laboratory tests were carried out by supplying temperature- and humidity-controlled standard gas to the NO_2 and CO sensors. RH ranged from 40% to 70%, and it was not possible to generate higher RH.<br><br><u>Field Testing</u><br><br>Three identical sets of monitoring systems, mounted on tripods, were co-located with the roadside Air Quality Monitoring Station (AQMS) in Central, Hong Kong operated by the Hong Kong Environmental Protection Department. The station is located at the junction of Charter Road and Des Voeux Road with busy traffic. Its $PM_{2.5}$ inlets were 4.5 m above the ground. The field co-location was carried out for three days. During the test, the sensor systems were placed on the same platform at a distance of 1 to 2 m from the inlets of the AQMS.	k factor to account for the photometer response to the concentration of particles with different characteristics from calibration aerosols and the impact of RH when above 40% due to the alternation of the particle refractive index by wetted particles.				between the ambient air and the sulfuric acid electrode.<br><br>The laboratory-test derived equations showed the inherent relation between the sensor gain in differential voltage and the pollutant concentration with the correction due to the varying T or RH, if any. The parameters in the correction equation were further refined in the field performance tests by multiple regression analysis results between the actual pollutants' concentration and the sensor gain under ambient conditions.<br><br><u>Field Test Results</u><br><br>Ambient T and RH ranged from 15 to 22 °C and 33% to 89%. **As such T was in the range tested in the laboratory experiment, while RH was much higher.**<br><br>The field tests of MAS sensors in comparison with the routine AQMS monitoring data showed **overall very good performance after correction by the developed algorithms in the temperature and humidity ranges encountered.**<br><br>CO concentration data between MAS and AQMS with a high linearity of correction and excellent agreement (R^2 = 0.97; m (slope) = 0.91).<br><br>NO_2 sensor and AQMS data followed very similar trends with good agreement (R^2 = 0.90; m =1.09).<br><br>MAS $PM_{2.5}$ sensor performance compared to the AQMS $PM_{2.5}$ data after k factor and humidity correction. At 5-min resolution, the two datasets showed good agreement in the overall trend (R^2 = 0.92; m = 1.05).<br><br><u>Drift</u><br><br>The authors did not investigate drift, but they discussed it. They discussed that a very important factor they did not account for in their short-term study is the

									possibility of response drift due to irreversible cell changes over time. They suggested that **longer studies are needed to better understand sensors' performance and limitations in the traffic impacted urban sites of Hong Kong and tropical/semitropical Asia.**
[13]	No	Oslo, Norway	AQMesh v3.5 platforms with Alphasense series B.	24 AQMesh platforms were co-located at an air quality reference monitoring station at Kirkeveien street, Oslo, Norway. The Kirkeveien station (10.7245 ° E, 59.9323 ° N) was located in a street with busy traffic and is equipped with CEN approved gas analyzers for CO, O_3 and nitrogen oxides (NOx).<br><br>CO is measured using non-dispersive infrared spectroscopy (EN14626), NO_x is measured using chemiluminescence (EN14211), and O_3 is measured using UV photometry (EN14625).	The co-location results showed that even for the same sensor type and platform version, the performance can be very different from sensor to sensor.<br><br>To reduce the bias and errors, a linear regression employing the calibration data from the co-location was applied. The slope and offset were calculated for each of the 24 AQMesh platforms.<br><br>The calibration was carried out separately for each individual gas sensor within each platform in order to achieve the best possible performance for the various species.	CO, NO, NO_2, O_3<br><br>T, RH, P	1-hour temporal resolution although the AQMesh can deliver 15-minute averaged data.	~2 months<br><br>13th April 2015 to 24th June 2015 (Performance testing)<br><br>January 2016 for demonstrating mapping methodology for NO_2 as its levels are relatively high in January.	From the co-location, **NO had an average r value of 0.86 (0.60-0.98), NO_2 had an average r value of 0.49 (0.21-0.72), and O_3 had an average r value of 0.54 (0.09-0.81). The co-location results show that even for the same sensor type and platform version, the performance can be very different from sensor to sensor.**<br><br>To reduce the bias and errors, the authors applied a linear regression employing the calibration data from the co-location for each of the 24 AQMesh platforms. The calibration was carried out separately for each **individual gas sensor within each platform in order to achieve the best possible performance for the various species**. This process reduced the average RMSE from 30 ppb to 9 ppb for NO_2.<br><br>Validation against official data from air quality monitoring stations equipped with reference instrumentation indicated that the data fusion method was capable of reproducing city-wide averaged values with an R^2 of 0.89 and a RMSE of 14.3 µg/m^3. It was also found capable of reproducing the typical NO_2 daily cycles.<br><br>The (data fusion) methodology for combining observations from a network of low-cost air quality sensors and a high-resolution urban-scale air quality model was demonstrated using data collected by a network of 24 low-cost air quality platforms, which were deployed at the premises of kindergartens throughout urban Oslo. The authors focused on NO_2 as one of the primary traffic-related air

									pollutants. Data fusion maps were created using NO_2 observations from the low-cost sensor network and a time-in-variant modeled air quality map for each hour of January 2016. The results indicated that qualitatively the methodology is able to produce realistic high-resolution maps of urban air quality at high temporal resolution. The fused maps provide realistic daily cycles of NO_2 and it should be noted here that the temporal evolution of the maps and the derived time series are entirely driven by the sensor data, whereas the model only provides information on typical spatial patterns.<br><br>While the mapping methodology was demonstrated solely for NO_2, it can be readily applied to measurements of PM_{10} and $PM_{2.5}$ to produce up-to-date high-resolution maps of urban PM.<br><br>**Results indicated that despite significant uncertainties at the individual sensor level, appropriate processing techniques such as the data fusion method presented were able to exploit the "swarm knowledge" of the entire sensor network and to extract realistic signals, resulting in high-resolution spatial-temporal mapping of urban air quality.**
[20]	Yes	Tušimice Observatory, Czech Republic	Cairclip gas sensor, Plantower PMS7003 and Alphasense OPC-N2 .	This study presents the results of almost one-year of field-testing measurements which were compared with co-located reference monitors or equivalent methods used within the Czech national ambient air quality monitoring network.<br><br>Gaseous sensors were compared to a reference monitor from Teledyne API company (San Diego, CA, USA). Particular onboard analyzers included the T100 (SO_2), T200 (NO_2) and T400 (O_3).	No calibration was applied.	SO_2, NO_2, O_3, and CO; PM_1, $PM_{2.5}$, and PM_{10}<br><br>RH, T	10-minute temporal resolution<br><br>Measurements averaged to hourly concentrations	4 – 11 months<br><br>Cairpol gas sensors measured from November 2017 to September 2018, Plantower particle counters from March 2018 to December 2018, and Alphasense from September 2018 to January 2019	Results demonstrated that data quality depends on the early detection of defective units and changes caused by the effect of meteorological conditions (effect of air temperature and humidity on gas sensors and effect of air humidity with condensation conditions on particle counters), or by the interference of different pollutants (especially in gas sensors). Therefore, comparative measurement is necessary prior to each sensor's field applications.<br><br><u>Cairpol Gas Sensors</u><br><br>All sensor pairs demonstrated high intra-sensor correlations with R_s = 0.99 (SO_2), R_s = 1.00 (NO_2), R_s = 1.00

				Particulate matter sensors were compared with the MP101M (Environment SA, Envea, France) reference monitor and the Fidas200 (Palas, Germany (DE)) equivalent monitor.<br><br>Statistical metrics for performance assessment included non-parametric Spearman's rank correlations (R_s) due to the non-normal distribution of measured values, coefficient of determination (R^2), mean bias error (MBE), mean absolute error (MAE) and root-mean-square error (RMSE).					(O_3) and R_s = 0.81 (CO). Despite this, significant intra-sensor variability, and drift, was present initially among the SO_2 sensor (consistent difference in mean concentrations of 60 ppb for start of testing, presumed erroneous initial manufacture calibration), and a difference in mean concentrations of about 10 ppb for CO sensor arising after 3 months of measurement (potential interference with other pollutants not ruled out as a cause). No such drift was identified in the NO_2 or O_3 sensors. Comparisons with reference monitors saw overall highly unsatisfactory results and weak correlations for SO_2 and NO_2, and the strongest in O_3. CO sensors were not compared with a reference monitor.<br><br>SO_2: R_s = 0.01; MBE = -70.23 (ppb)<br><br>NO_2: R_s = - 0.26; MBE = 27.56 (ppb)<br><br>O_3: R_s = 0.68; MBE = 10.54 (ppb)<br><br>In all the Cairclip sensors, significant correlations of measured gas concentrations with the ambient air temperature ($R_s > 0.79$) and relative humidity ($R_s < -0.50$) were found. The O_3 sensor, which had the best correlations, still experienced variations in measurement quality depending on the months, with the best performance during the warmer months. The maximum lifetime of the gas sensors was identified as 11-months due to significant drift to unreal stable values at this time.<br><br><u>Plantower and Alphasense Particle Counters</u><br><br>Intra-sensors comparison within pairs of the Plantower and Alphasense showed highly significant correlations in the measured PM concentrations (both types $R_s > 0.95$ in all PM fractions. In both sensor types, no significant data drifts were found within the sensor pairs, although the OPC-N2 sensors tended to differ in mean and standard deviation (SD)

									concentration values (especially in the case of $PM_{2.5}$ and PM_{10} fractions.<br><br>The PMS7003 showed good measurement quality and correlations with the equivalent and reference monitors ($R_s > 0.70$ for all PM fractions with Fidas200, and $R_s > 0.62$ for $PM_{2.5}$ and PM_{10} with the MP101 M). MBE ranged from -7.94 to 1.39 μg/m^3, and MAE ranged from 3.24 to 12.05 μg/m^3. No significant outliers were identified.<br><br>The OPC-N2 particle counters experienced considerably weaker measurement quality. Strong positive correlations with both control monitors were still evident (with Fidas200 $R_s > 0.75$ for all fractions, with RM $R_s > 0.63$ for $PM_{2.5}$ and PM_{10}), but mean values and SDs differed significantly in all PM fractions, with MBE ranging from - 174.61 to - 26.27 μg/m^3 and MAE ranging from 27.52 to 178.06 μg/m^3. This high value of the measurement errors showed the presence of extreme outliers in all PM fractions analyzed by OPC-N2 sensors.<br><br>In both tested particle counter types, the concentrations of all measured fractions correlated weakly negatively (yet statistically significantly) with ambient T (Plantower sensors $R_s < -0.24$, Alphasense sensors $R_s < -0.16$) and significantly positively with RH (Plantower sensors $R_s > 0.46$, Alphasense sensors $R_s > 0.57$). **In the case of the PMS7003 particle counters, there were no extreme outliers in the measured concentrations detected in relation to the effect of T and RH. The OPC-N2 experienced large high-concentration outliers during RH > 80%.**

Table S2 AQI Calculation Values, Source: (United States Environmental Protection Agency, 2018)

AQI Level	AQI_lo	AQI_hi	NO_2 (ppb) 1-Hr Average		PM_{10} (ug/m^3) 24-Hr Average		PM_{25} (ug/m^3) 24-Hr Average		O_3 (ppm) 8-Hr Average	
			CONC_lo	CONC_hi	CONC_lo	CONC_hi	CONC_lo	CONC_hi	CONC_lo	CONC_hi
Good	0	50	0	53	0	54	0.0	12.0	0	0.054
Moderate	51	100	54	100	55	154	12.1	35.4	0.055	0.070
Unhealthy for Sensitive Groups	101	150	101	360	155	254	35.5	55.4	0.071	0.085
Unhealthy	151	200	361	649	255	354	55.5	150.4	0.086	0.105
Very Unhealthy	201	300	650	1249	355	424	150.0	250.4	0.106	0.200
Hazardous	301	400	1250	1649	425	504	250.5	350.4	0.405*	0.504*
	401	500	1650	2049	505	604	350.5	500.4	0.505*	0.604*
* 1-hour values										

3 **Table S3 Performance Statistics by Device and Pollutants across all AQY1 Units Examined**

Device ID	O_3 Raw Data	O_3 Calibrated Data
	MAPE (%)	MAPE (%)
AQY-BA-353	20%	5%
AQY-BA-431	63%	28%
AQY-BA-432	84%	30%
AQY-BA-464	28%	44%
AQY-BA-480	56%	45%
AQY-BA-481	46%	44%
AQY1-BA-479A	68%	14%
AQY1-BA-480A	61%	26%
AQY1-WilburSpare-07	34%	18%
AQY1-WilburSpare-08	45%	6%
AQY1-WilburSpare-09	77%	9%
AQY1-WilburSpare-10	52%	16%
Device ID	NO_2 Raw Data	NO_2 Calibrated Data
	MAPE (%)	MAPE (%)
AQY-BA-353	170%	43%
AQY-BA-431	361%	72%
AQY-BA-432	210%	63%
AQY-BA-464	305%	106%
AQY-BA-480	334%	26%
AQY-BA-481	312%	26%
AQY1-BA-479A	170%	75%
AQY1-BA-480A	261%	54%
AQY1-WilburSpare-07	338%	33%
AQY1-WilburSpare-08	179%	42%
AQY1-WilburSpare-09	145%	87%
AQY1-WilburSpare-10	212%	60%
Device ID	$PM_{2.5}$ Raw Data	$PM_{2.5}$ Calibrated Data
	MAPE (%)	MAPE (%)
AQY-BA-353	63%	80%

**AQY-BA-431**	58%	61%
**AQY-BA-432**	62%	65%
**AQY-BA-464**	41%	40%
**AQY-BA-480**	50%	52%
**AQY-BA-481**	64%	39%
**AQY1-BA-479A**	68%	124%
**AQY1-BA-480A**	65%	132%
**AQY1-WilburSpare-07**	49%	29%
**AQY1-WilburSpare-08**	63%	64%
**AQY1-WilburSpare-09**	67%	99%
**AQY1-WilburSpare-10**	61%	88%
**Device ID**	**PM_{10} Raw Data**	**PM_{10} Calibrated Data**
	**MAPE (%)**	**MAPE (%)**
**AQY-BA-353**	63%	62%
**AQY-BA-431**	55%	64%
**AQY-BA-432**	61%	54%
**AQY-BA-464**	36%	31%
**AQY-BA-480**	NA	NA
**AQY-BA-481**	58%	29%
**AQY1-BA-479A**	53%	77%
**AQY1-BA-480A**	60%	77%
**AQY1-WilburSpare-07**	51%	20%
**AQY1-WilburSpare-08**	60%	42%
**AQY1-WilburSpare-09**	63%	73%
**AQY1-WilburSpare-10**	58%	55%

4

Underlying Regression Analysis Results for Calibrated Data

1

Regression plots between the reference monitor readings (x) and low-cost sensors (y) by Device ID

1. Ozone

Bivariate Fit of O3_Cal_Data By O3_HintonData Device ID=AQY1-BA-479A

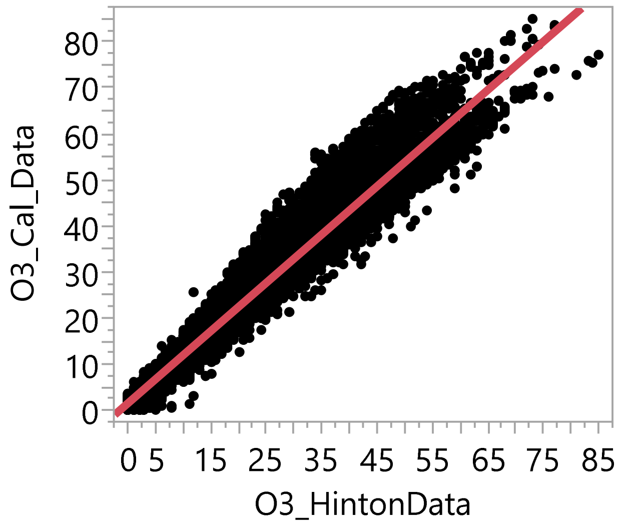

Linear Fit

Linear Fit

O3_Cal_Data = 1.8869909 + 1.047227*O3_HintonData

Bivariate Fit of O3_Cal_Data By O3_HintonData Device ID=AQY1-BA-480A

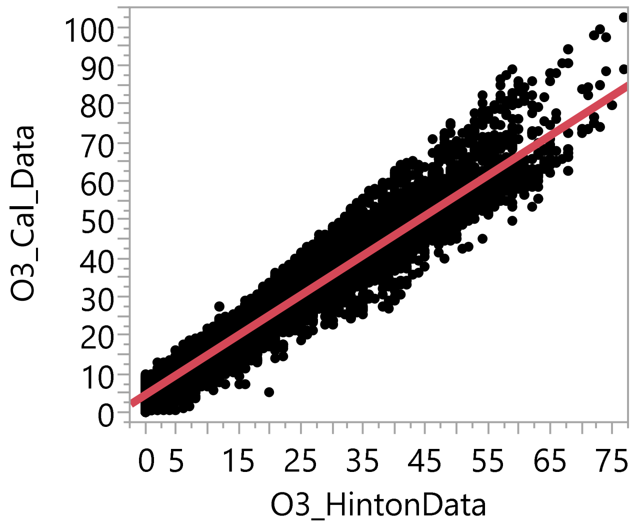

Linear Fit

Linear Fit

O3_Cal_Data = 4.9704812 + 1.033926*O3_HintonData

Bivariate Fit of O3_Cal_Data By O3_HintonData Device ID=AQY1-WilburSpare-07

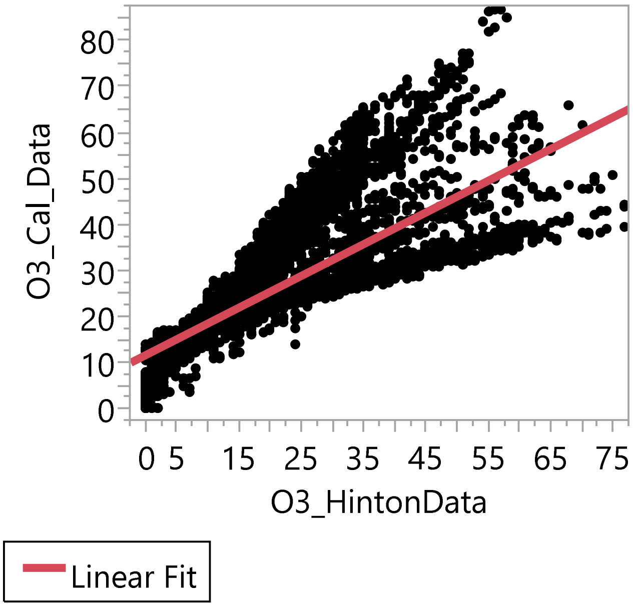

Linear Fit

O3_Cal_Data = 11.864831 + 0.6891471*O3_HintonData

Bivariate Fit of O3_Cal_Data By O3_HintonData Device ID=AQY1-WilburSpare-08

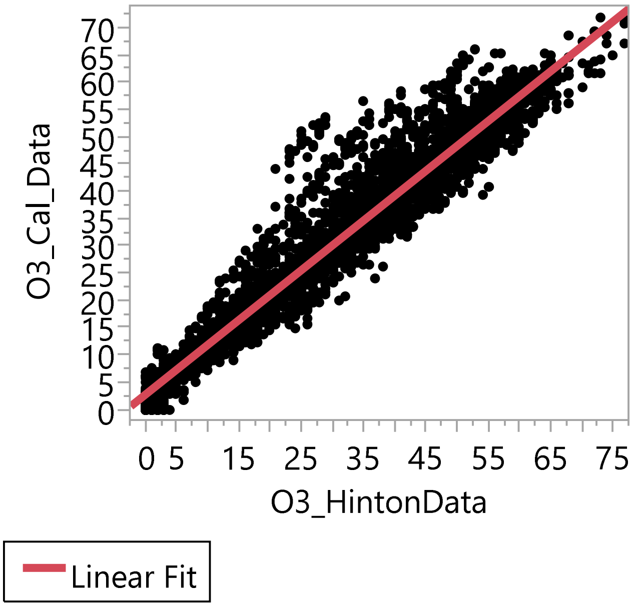

Linear Fit

O3_Cal_Data = 2.9569704 + 0.9123921*O3_HintonData

Bivariate Fit of O3_Cal_Data By O3_HintonData Device ID=AQY1-WilburSpare-09

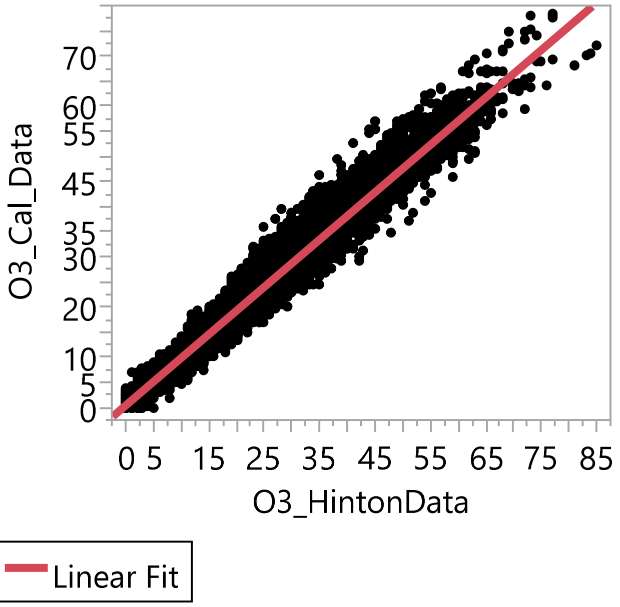

Linear Fit

O3_Cal_Data = 0.7581913 + 0.9431152*O3_HintonData

Bivariate Fit of O3_Cal_Data By O3_HintonData Device ID=AQY1-WilburSpare-10

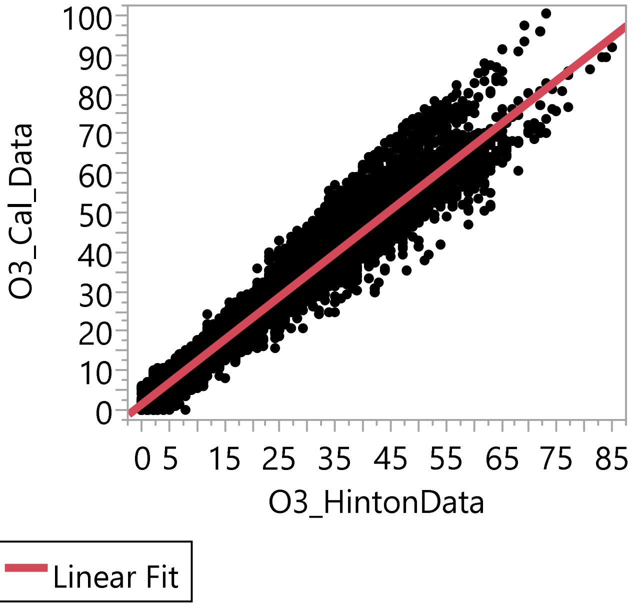

Linear Fit

O3_Cal_Data = 1.8100818 + 1.0948811*O3_HintonData

Bivariate Fit of O3_Cal_Data By O3_HintonData Device ID=AQY-BA-353

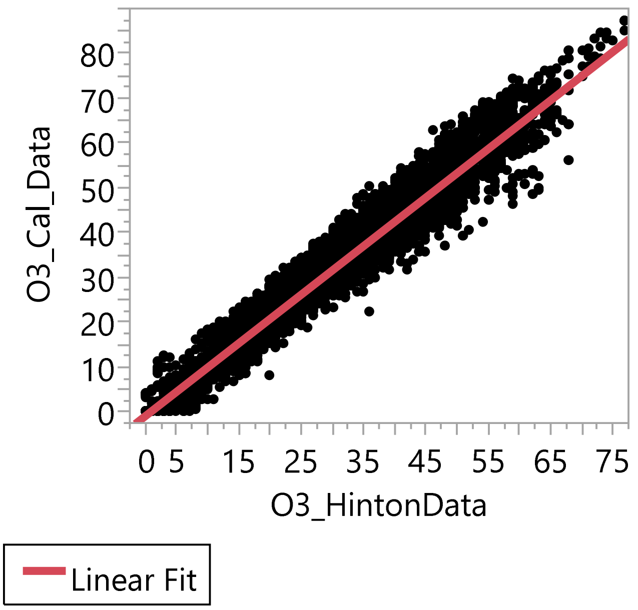

Linear Fit

O3_Cal_Data = -0.575053 + 1.0844071*O3_HintonData

Bivariate Fit of O3_Cal_Data By O3_HintonData Device ID=AQY-BA-431

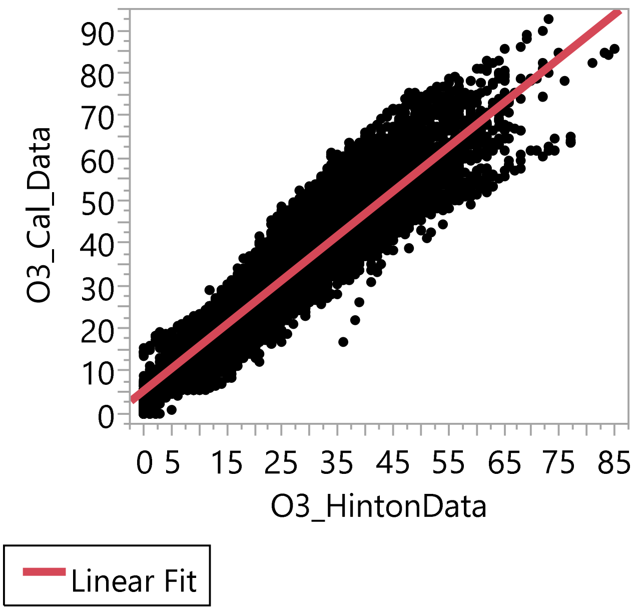

Linear Fit

O3_Cal_Data = 5.6688616 + 1.0419061*O3_HintonData

Bivariate Fit of O3_Cal_Data By O3_HintonData Device ID=AQY-BA-432

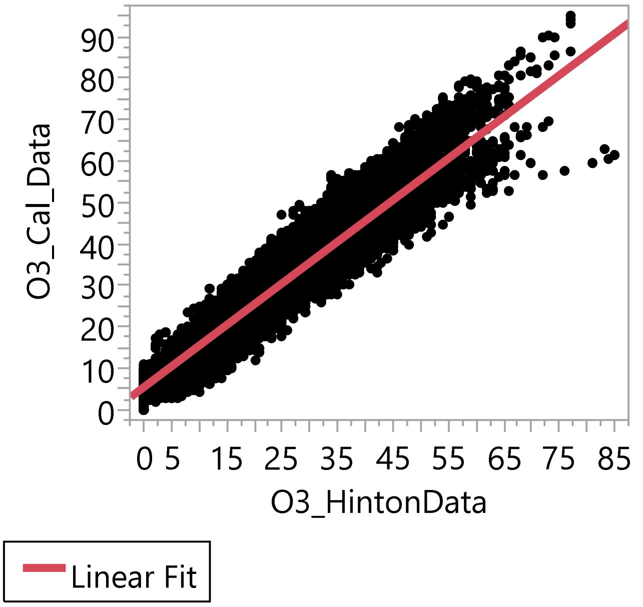

Linear Fit

O3_Cal_Data = 5.7789284 + 1.0041365*O3_HintonData

Bivariate Fit of O3_Cal_Data By O3_HintonData Device ID=AQY-BA-464

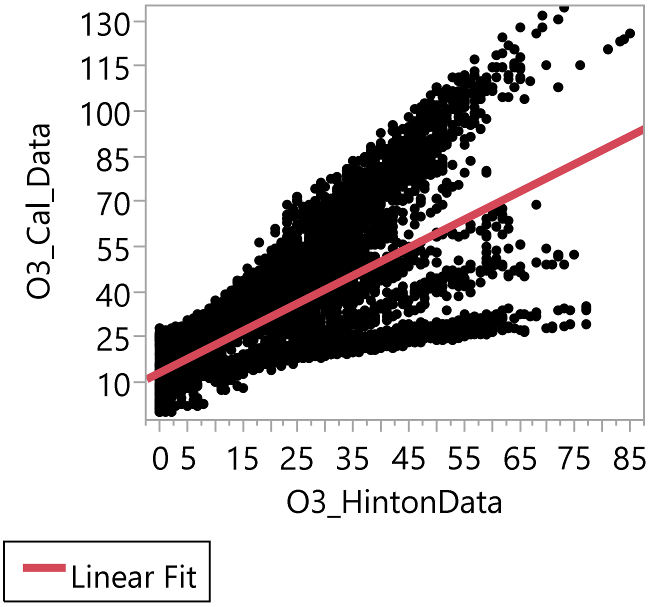

Linear Fit

O3_Cal_Data = 13.617921 + 0.9233601*O3_HintonData

Bivariate Fit of O3_Cal_Data By O3_HintonData Device ID=AQY-BA-480

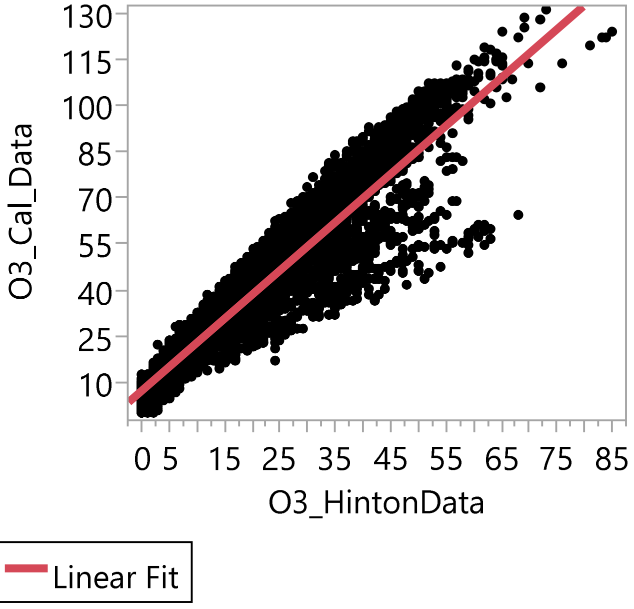

Linear Fit

O3_Cal_Data = 7.7068458 + 1.5685309*O3_HintonData

Bivariate Fit of O3_Cal_Data By O3_HintonData Device ID=AQY-BA-481

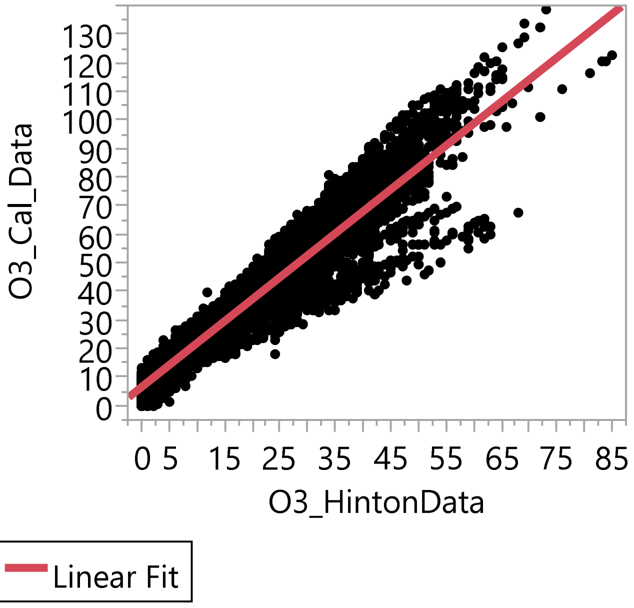

Linear Fit

O3_Cal_Data = 7.1454695 + 1.5357015*O3_HintonData

2. NO_2

Bivariate Fit of NO2_Cal_Data By NO2_HintonData Device ID=AQY1-BA-479A

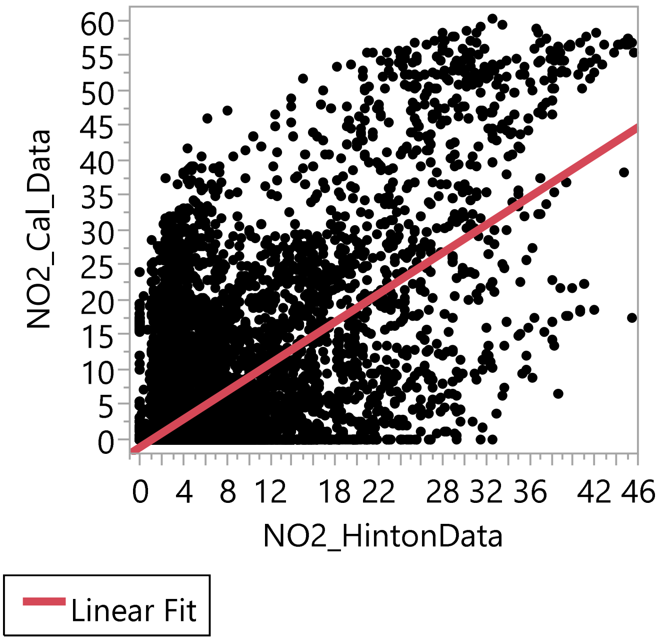

Linear Fit

NO2_Cal_Data = -0.833695 + 0.9957524*NO2_HintonData

Bivariate Fit of NO2_Cal_Data By NO2_HintonData Device ID=AQY1-BA-480A

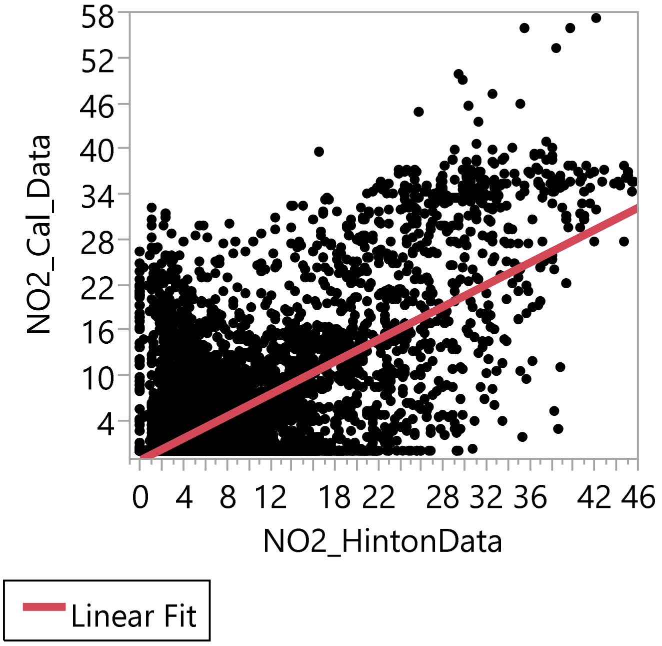

Linear Fit

NO2_Cal_Data = -0.985607 + 0.7242245*NO2_HintonData

Bivariate Fit of NO2_Cal_Data By NO2_HintonData Device ID=AQY1-WilburSpare-07

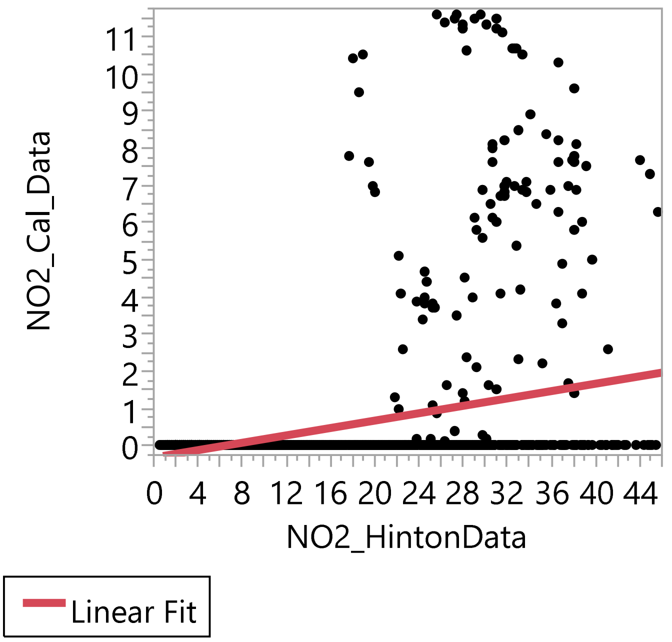

Linear Fit

NO2_Cal_Data = -0.286986 + 0.0495619*NO2_HintonData

Bivariate Fit of NO2_Cal_Data By NO2_HintonData Device ID=AQY1-WilburSpare-08

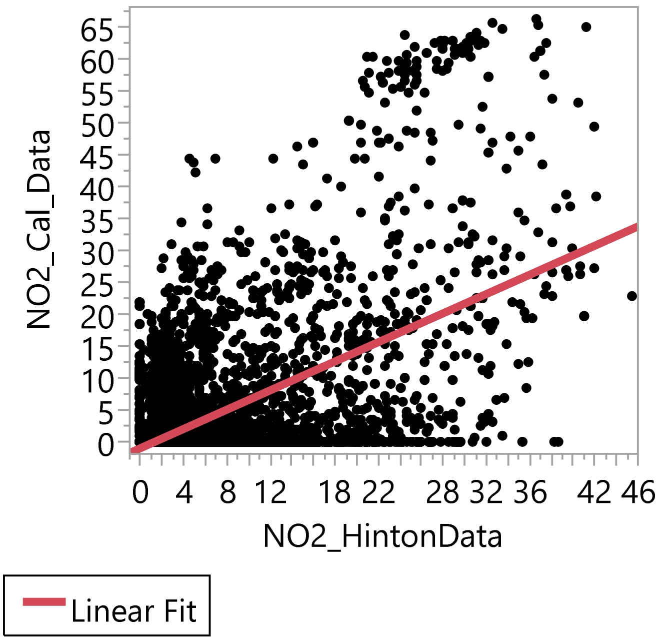

Linear Fit

NO2_Cal_Data = -0.921431 + 0.7556628*NO2_HintonData

Bivariate Fit of NO2_Cal_Data By NO2_HintonData Device ID=AQY1-WilburSpare-09

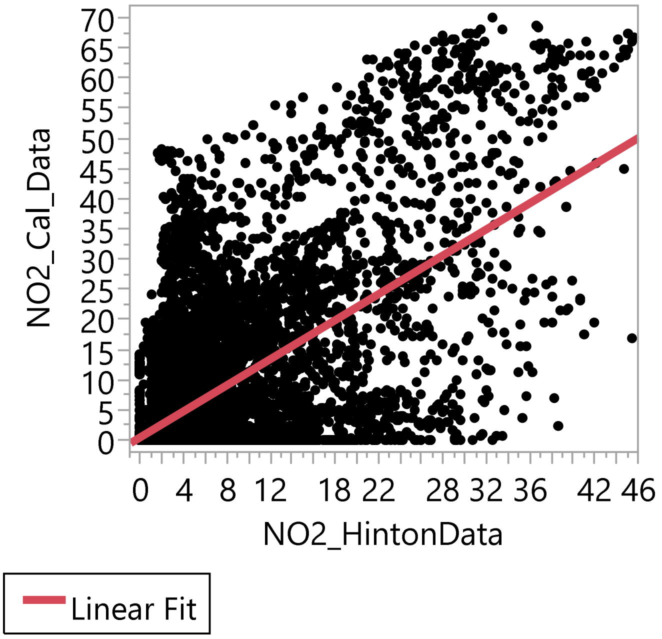

Linear Fit

NO2_Cal_Data = 0.7280688 + 1.0770973*NO2_HintonData

Bivariate Fit of NO2_Cal_Data By NO2_HintonData Device ID=AQY1-WilburSpare-10

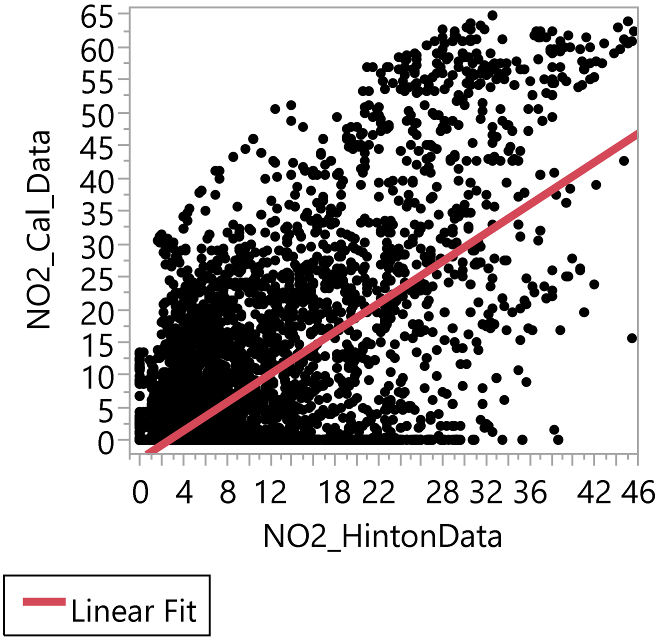

Linear Fit

NO2_Cal_Data = -2.518526 + 1.0763525*NO2_HintonData

Bivariate Fit of NO2_Cal_Data By NO2_HintonData Device ID=AQY-BA-353

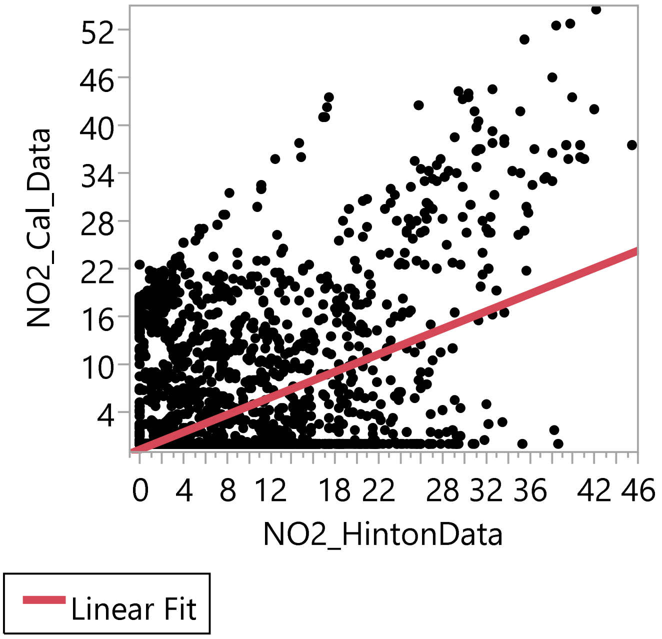

Linear Fit

NO2_Cal_Data = -0.488025 + 0.541493*NO2_HintonData

Bivariate Fit of NO2_Cal_Data By NO2_HintonData Device ID=AQY-BA-431

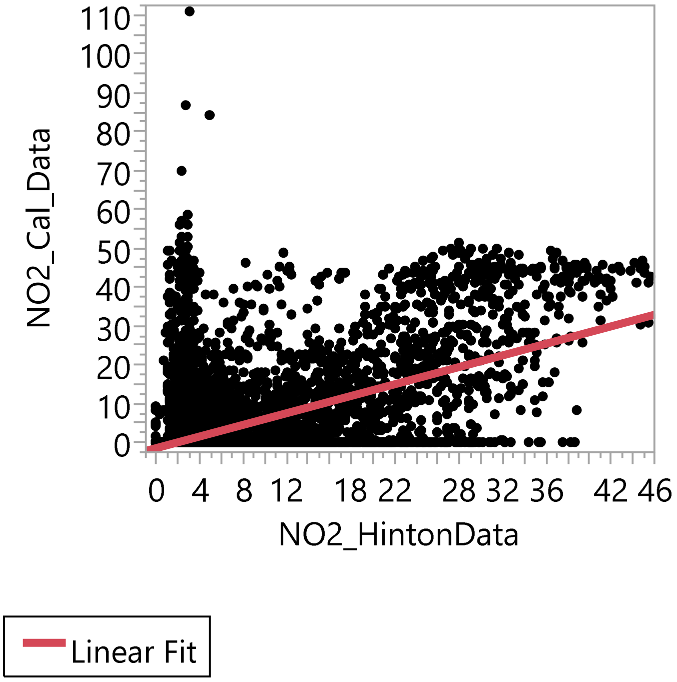

Linear Fit

NO2_Cal_Data = -1.14439 + 0.7454608*NO2_HintonData

Bivariate Fit of NO2_Cal_Data By NO2_HintonData Device ID=AQY-BA-432

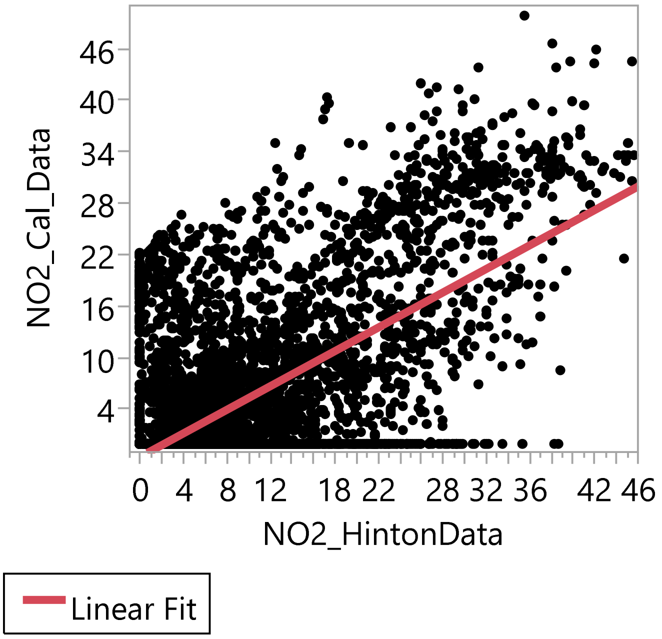

Linear Fit

NO2_Cal_Data = -1.376953 + 0.6843072*NO2_HintonData

Bivariate Fit of NO2_Cal_Data By NO2_HintonData Device ID=AQY-BA-464

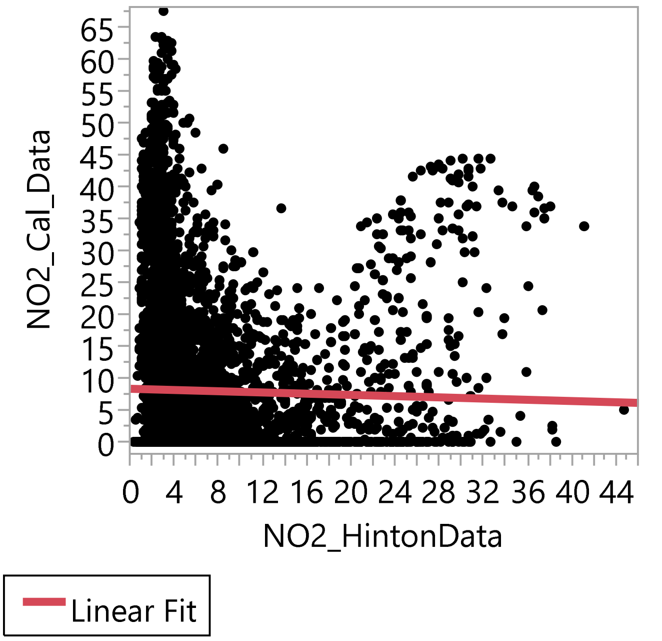

Linear Fit

NO2_Cal_Data = 8.3023017 - 0.0479185*NO2_HintonData

Bivariate Fit of NO2_Cal_Data By NO2_HintonData Device ID=AQY-BA-480

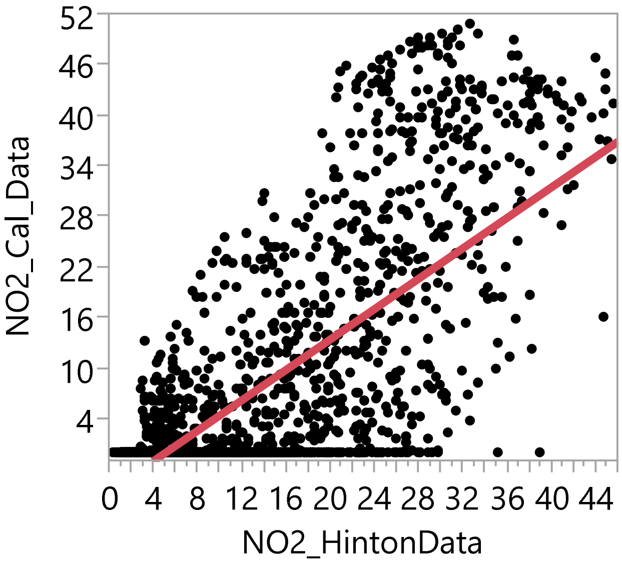

Linear Fit

Linear Fit

NO2_Cal_Data = -4.517013 + 0.9015863*NO2_HintonData

Bivariate Fit of NO2_Cal_Data By NO2_HintonData Device ID=AQY-BA-481

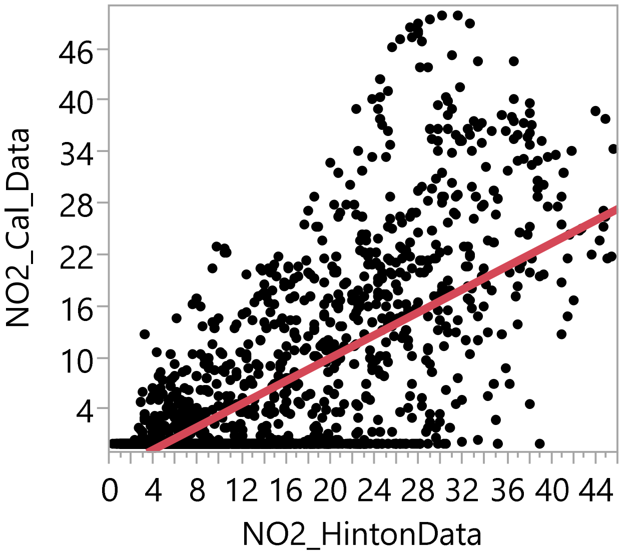

Linear Fit

Linear Fit

NO2_Cal_Data = -3.257288 + 0.6671978*NO2_HintonData

3. PM2.5

Bivariate Fit of PM25_Cal_Data By PM25_HintonData Device ID=AQY1-BA-479A

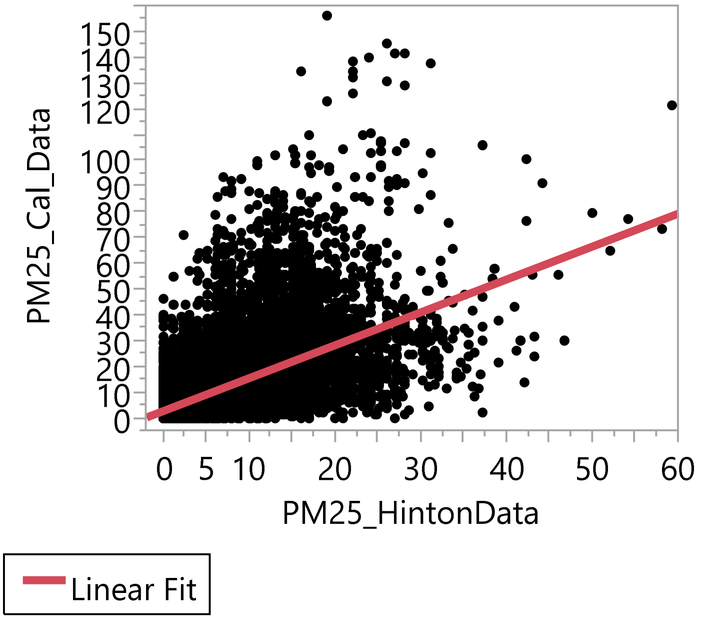

Linear Fit

PM25_Cal_Data = 2.8838534 + 1.2785478*PM25_HintonData

Bivariate Fit of PM25_Cal_Data By PM25_HintonData Device ID=AQY1-BA-480A

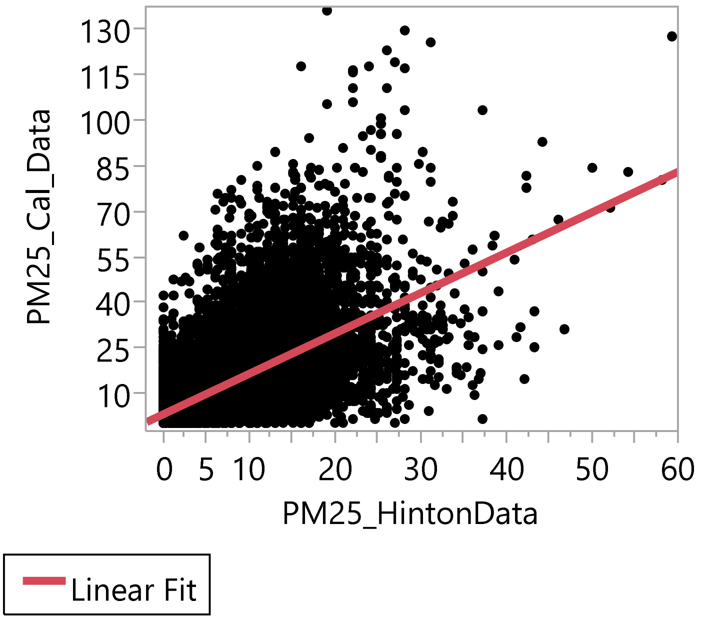

Linear Fit

PM25_Cal_Data = 3.364034 + 1.3340054*PM25_HintonData

Bivariate Fit of PM25_Cal_Data By PM25_HintonData Device ID=AQY1-WilburSpare-07

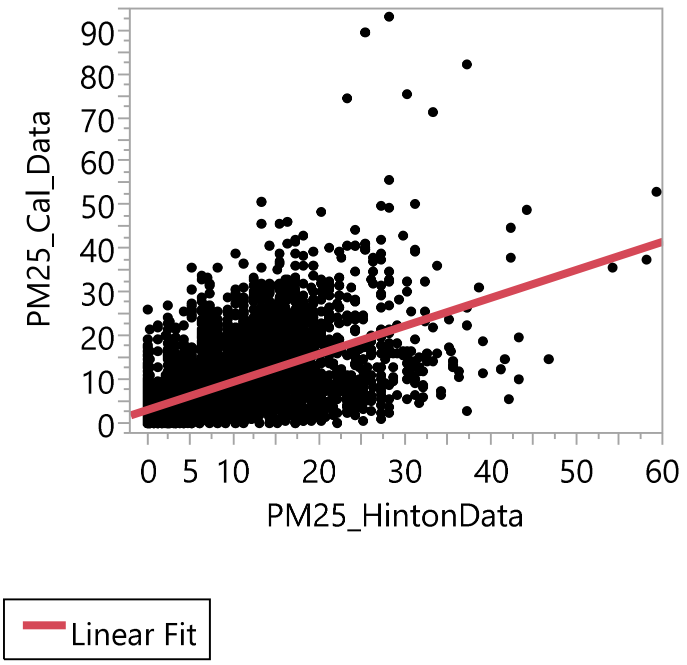

Linear Fit

PM25_Cal_Data = 3.0827156 + 0.6436353*PM25_HintonData

Bivariate Fit of PM25_Cal_Data By PM25_HintonData Device ID=AQY1-WilburSpare-08

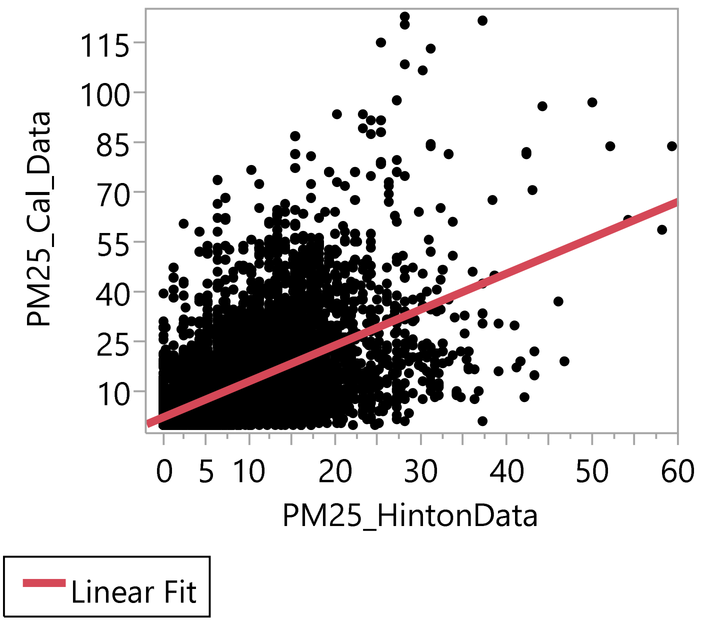

Linear Fit

PM25_Cal_Data = 2.6066944 + 1.079953*PM25_HintonData

Bivariate Fit of PM25_Cal_Data By PM25_HintonData Device ID=AQY1-WilburSpare-09

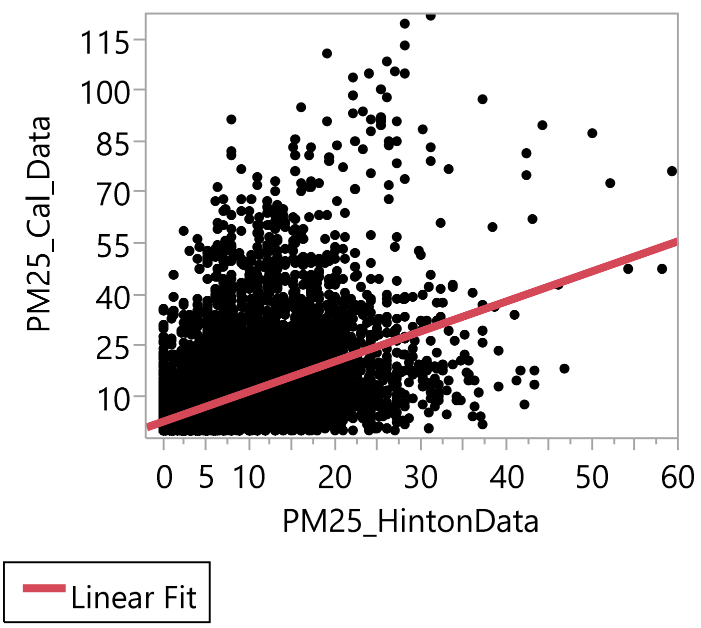

Linear Fit

PM25_Cal_Data = 2.8078453 + 0.883999*PM25_HintonData

Bivariate Fit of PM25_Cal_Data By PM25_HintonData Device ID=AQY1-WilburSpare-10

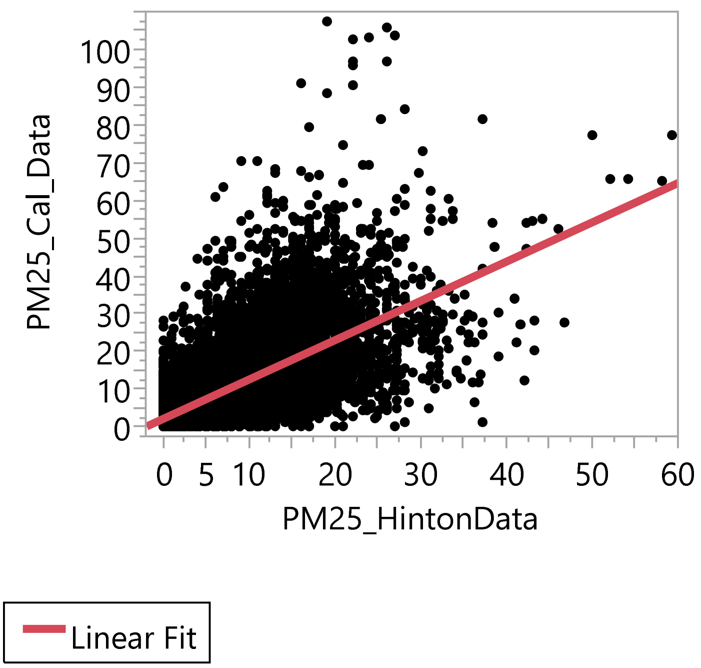

Linear Fit

PM25_Cal_Data = 2.4730917 + 1.0414393*PM25_HintonData

Bivariate Fit of PM25_Cal_Data By PM25_HintonData Device ID=AQY-BA-353

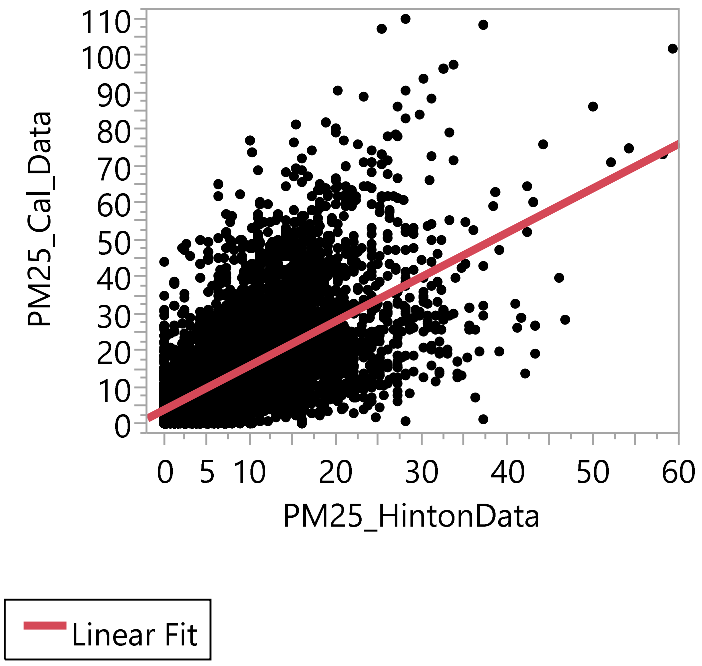

Linear Fit

PM25_Cal_Data = 4.1382138 + 1.201585*PM25_HintonData

Bivariate Fit of PM25_Cal_Data By PM25_HintonData Device ID=AQY-BA-431

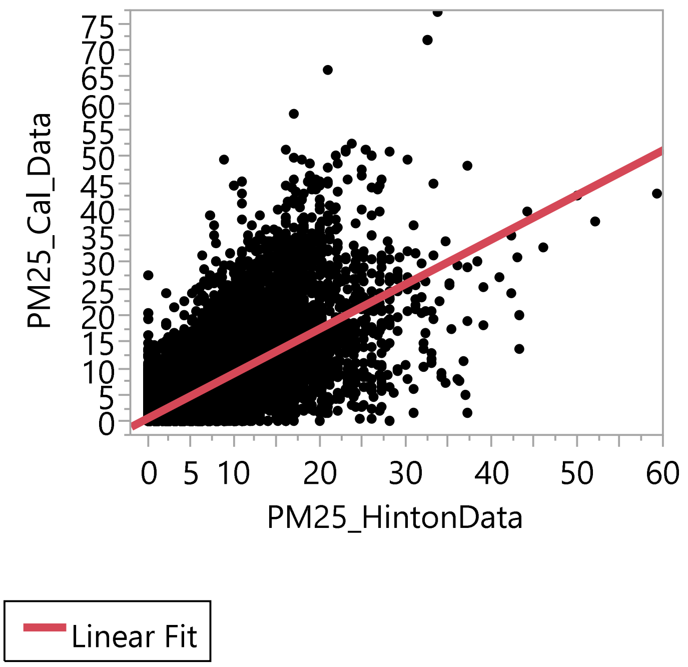

Linear Fit

PM25_Cal_Data = 0.8461895 + 0.8413467*PM25_HintonData

Bivariate Fit of PM25_Cal_Data By PM25_HintonData Device ID=AQY-BA-432

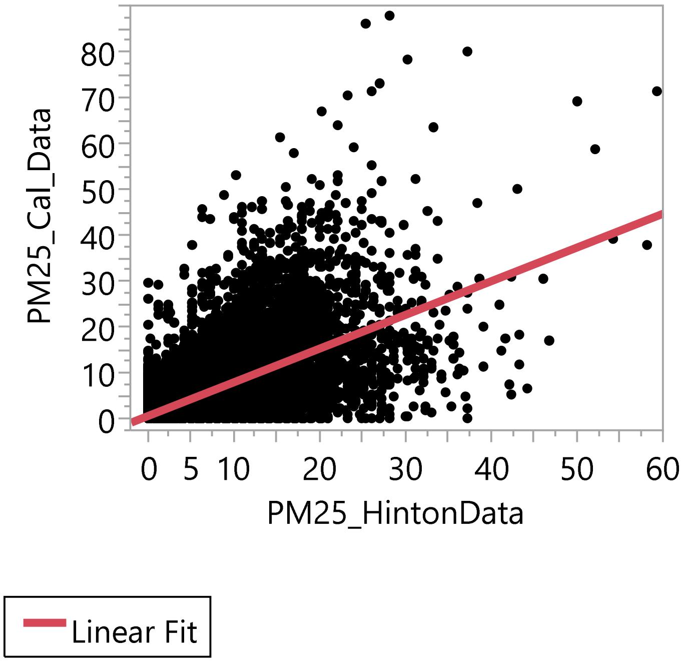

Linear Fit

PM25_Cal_Data = 0.8578091 + 0.7344749*PM25_HintonData

Bivariate Fit of PM25_Cal_Data By PM25_HintonData Device ID=AQY-BA-464

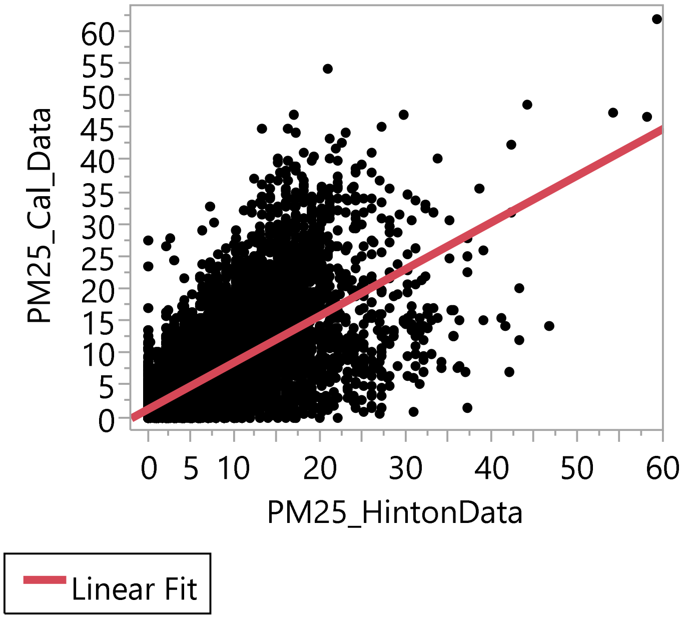

Linear Fit

PM25_Cal_Data = 1.3632937 + 0.7267461*PM25_HintonData

Bivariate Fit of PM25_Cal_Data By PM25_HintonData Device ID=AQY-BA-480

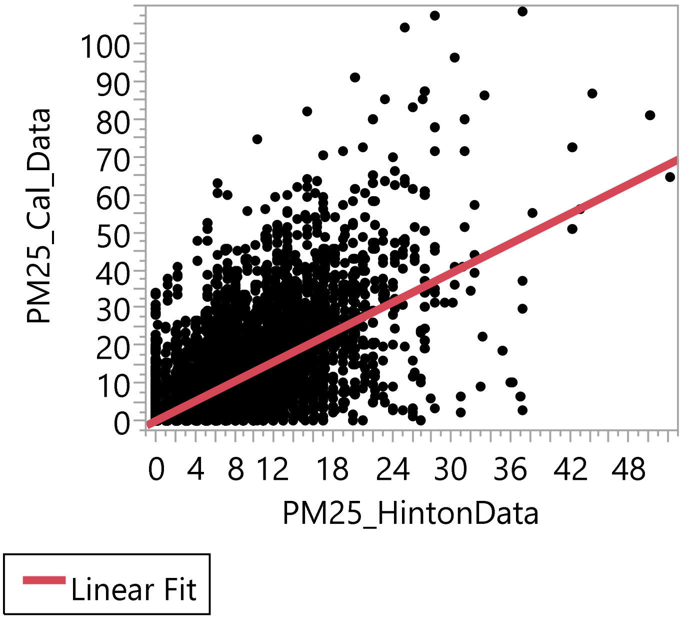

Linear Fit

PM25_Cal_Data = 0.3900841 + 1.3045784*PM25_HintonData

Bivariate Fit of PM25_Cal_Data By PM25_HintonData Device ID=AQY-BA-481

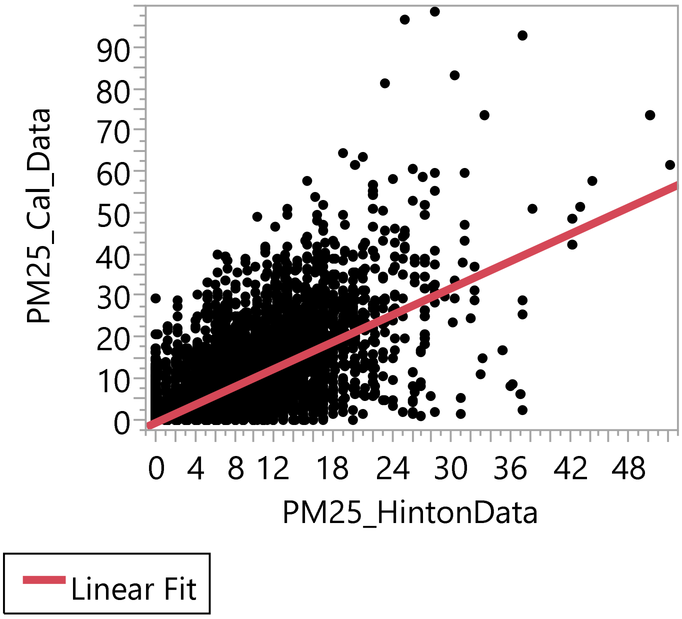

Linear Fit

PM25_Cal_Data = -0.435123 + 1.083844*PM25_HintonData

Regression plots between the reference monitor readings (x) and low-cost sensors (y) by Device ID (outliers are included)

PM10

Bivariate Fit of PM10_Cal_Data By PM10_HintonData Device ID=AQY1-BA-479A

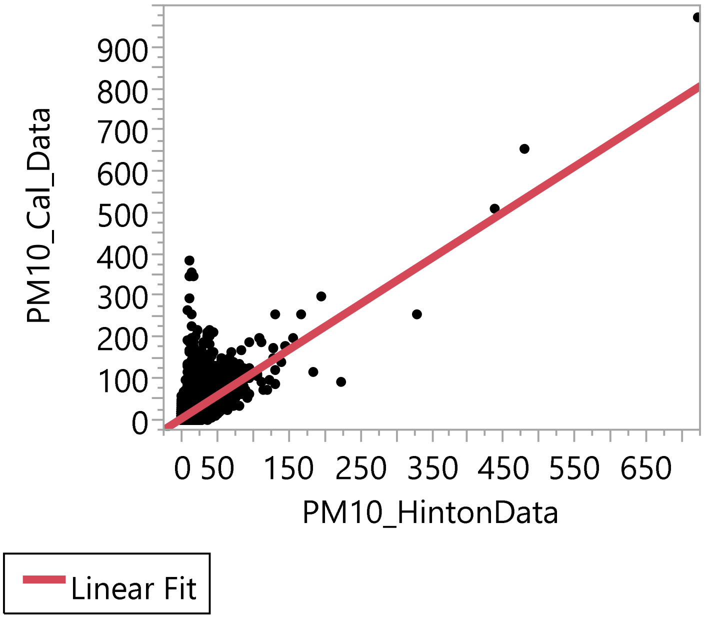

Linear Fit

PM10_Cal_Data = 6.3573051 + 1.1060458*PM10_HintonData

Bivariate Fit of PM10_Cal_Data By PM10_HintonData Device ID=AQY1-BA-480A

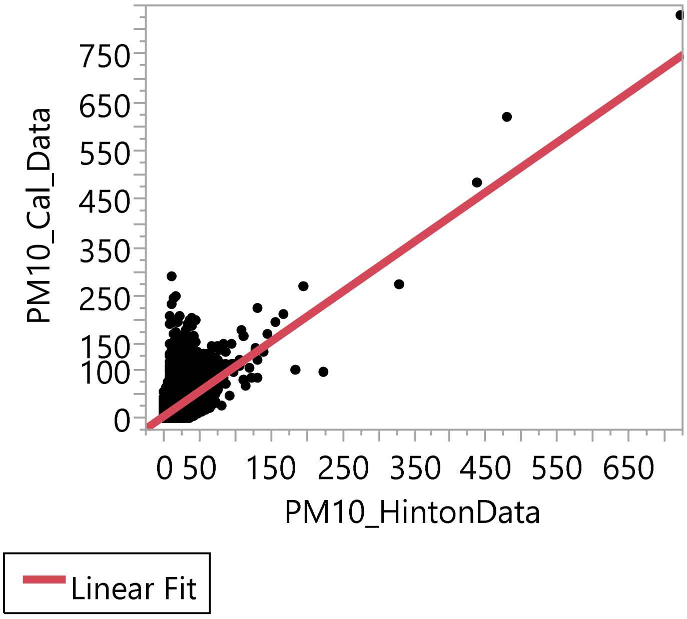

Linear Fit

PM10_Cal_Data = 5.9894411 + 1.0274075*PM10_HintonData

Bivariate Fit of PM10_Cal_Data By PM10_HintonData Device ID=AQY1-WilburSpare-07

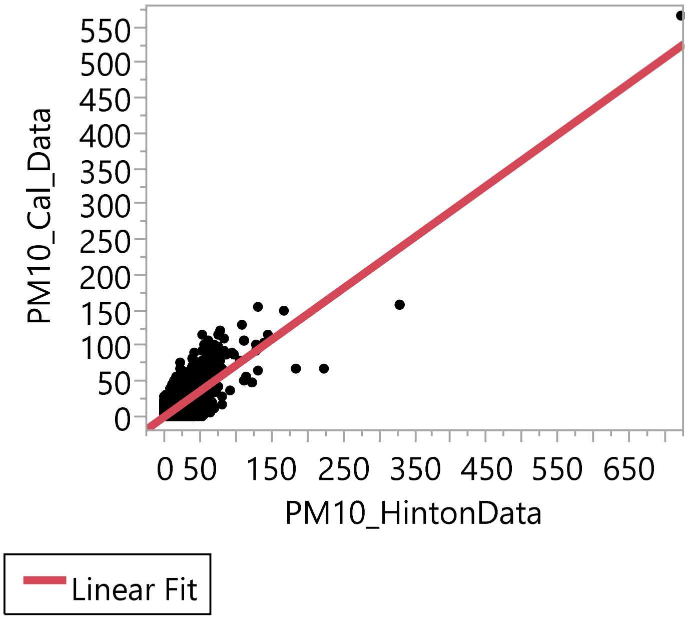

Linear Fit

PM10_Cal_Data = 1.2292176 + 0.725352*PM10_HintonData

Bivariate Fit of PM10_Cal_Data By PM10_HintonData Device ID=AQY1-WilburSpare-08

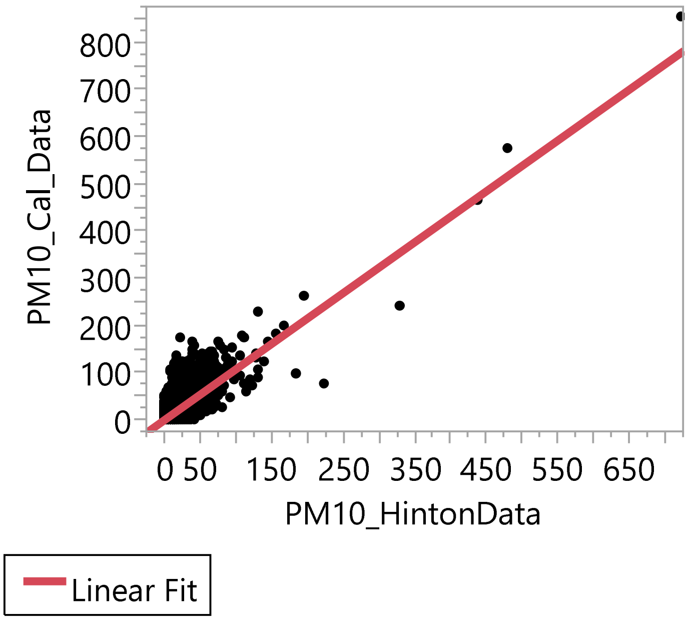

Linear Fit

PM10_Cal_Data = 1.946227 + 1.0774187*PM10_HintonData

Bivariate Fit of PM10_Cal_Data By PM10_HintonData Device ID=AQY1-WilburSpare-09

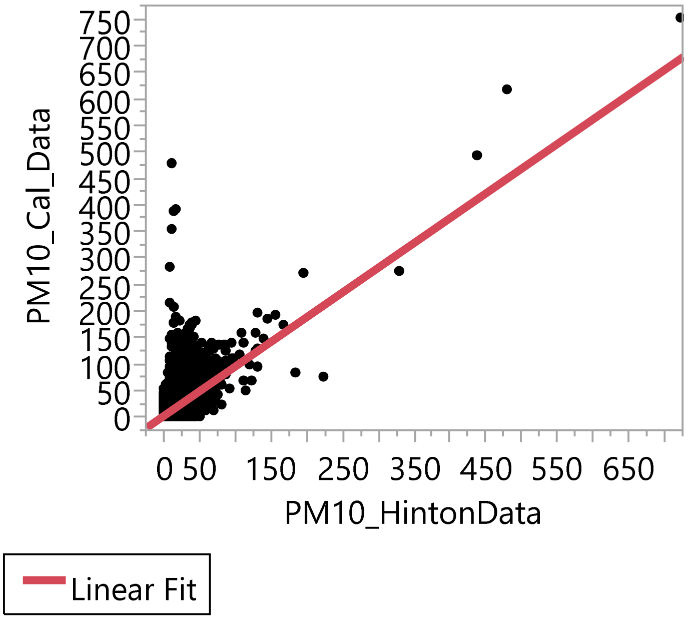

Linear Fit

PM10_Cal_Data = 4.8712306 + 0.9315192*PM10_HintonData

Bivariate Fit of PM10_Cal_Data By PM10_HintonData Device ID=AQY1-WilburSpare-10

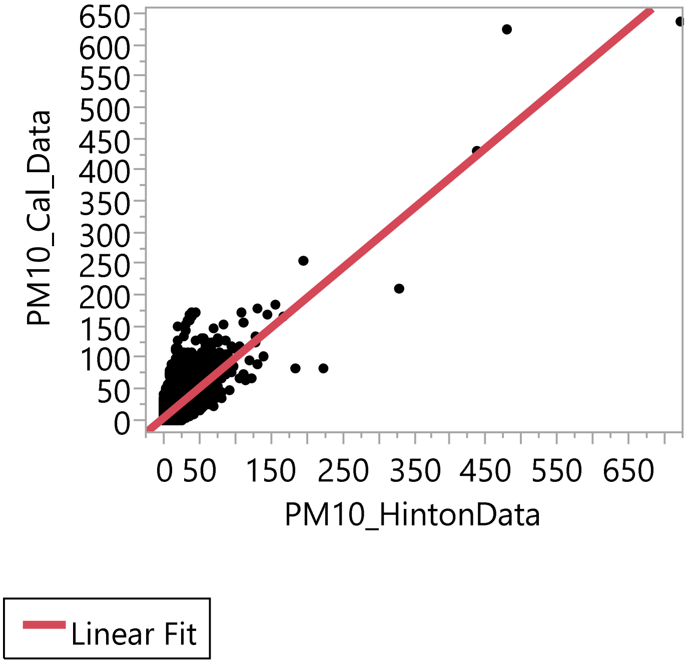

Linear Fit

PM10_Cal_Data = 5.8724448 + 0.9603098*PM10_HintonData

Bivariate Fit of PM10_Cal_Data By PM10_HintonData Device ID=AQY-BA-353

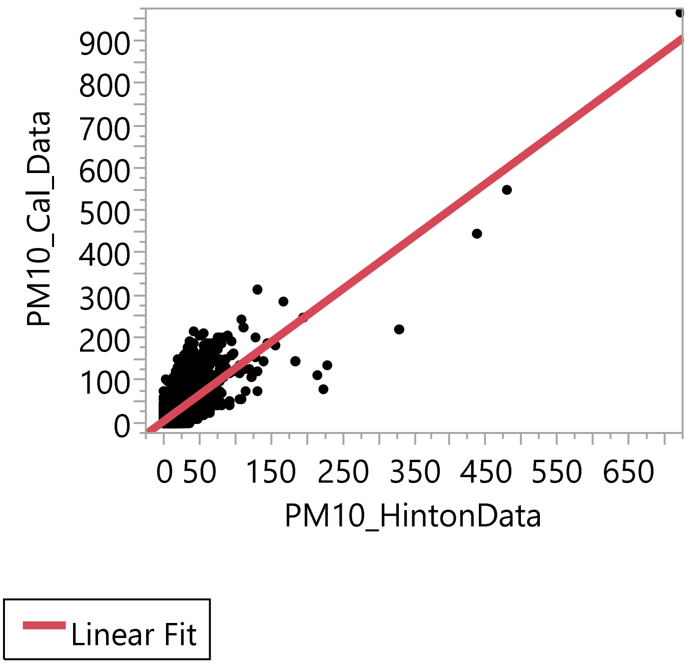

Linear Fit
PM10_Cal_Data = 7.1961824 + 1.2431797*PM10_HintonData

Bivariate Fit of PM10_Cal_Data By PM10_HintonData Device ID=AQY-BA-431

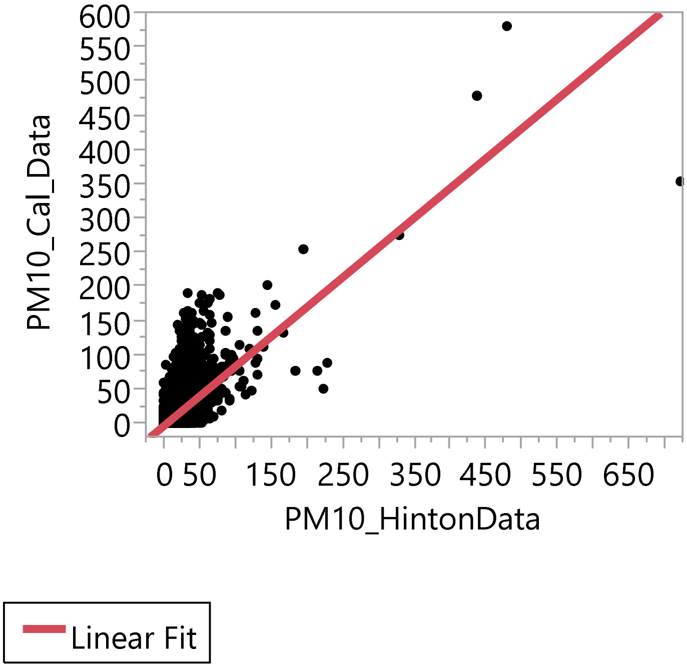

Linear Fit
PM10_Cal_Data = -2.205798 + 0.8688078*PM10_HintonData

Bivariate Fit of PM10_Cal_Data By PM10_HintonData Device ID=AQY-BA-432

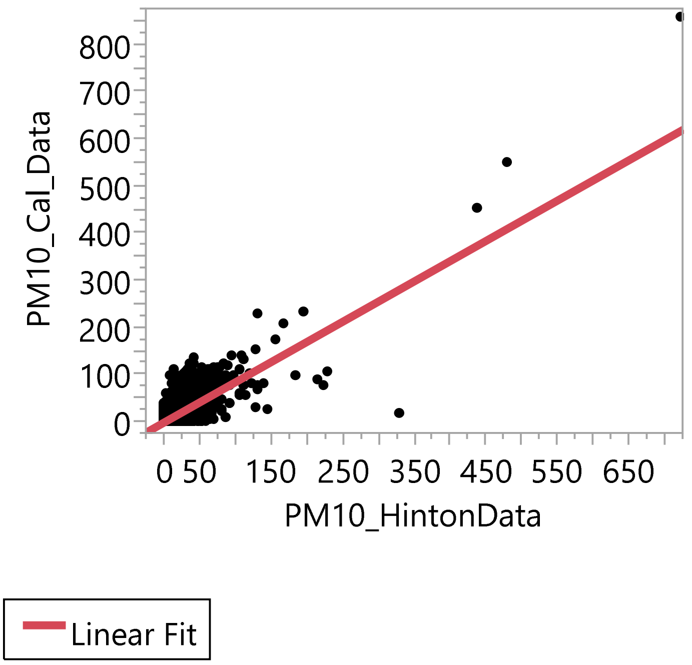

Linear Fit

PM10_Cal_Data = 0.0754955 + 0.8561238*PM10_HintonData

Bivariate Fit of PM10_Cal_Data By PM10_HintonData Device ID=AQY-BA-464

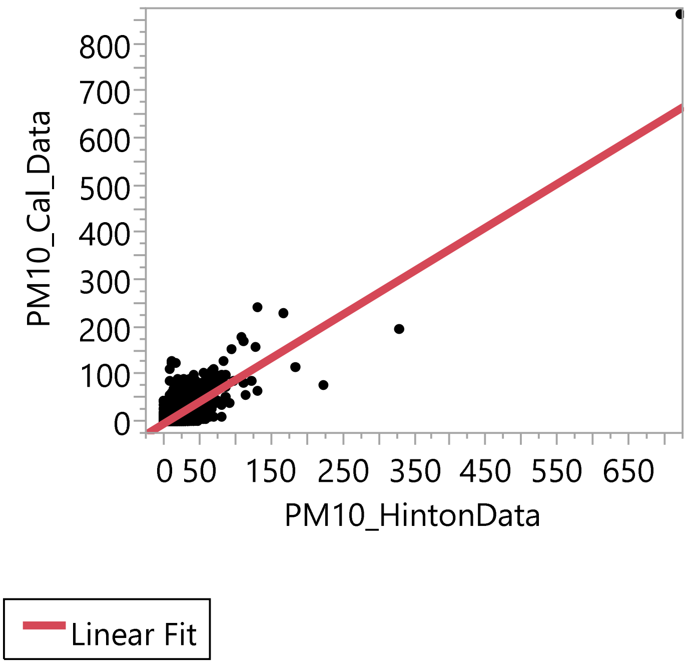

Linear Fit

PM10_Cal_Data = -2.231278 + 0.923752*PM10_HintonData

Bivariate Fit of PM10_Cal_Data By PM10_HintonData Device ID=AQY-BA-480

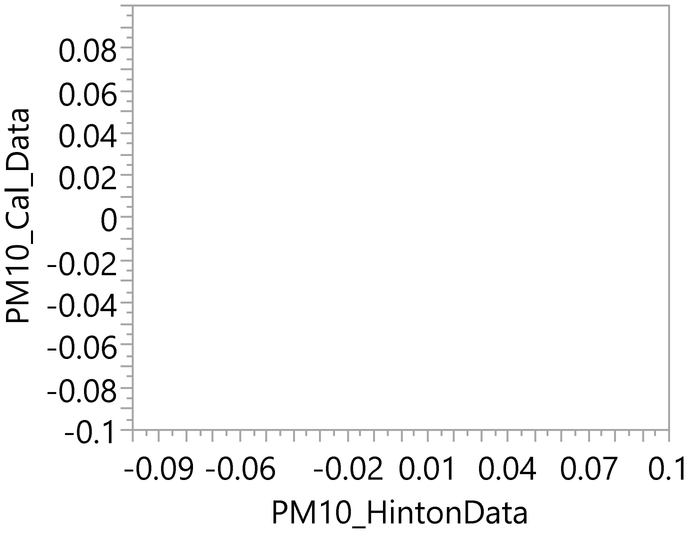

Bivariate Fit of PM10_Cal_Data By PM10_HintonData Device ID=AQY-BA-481

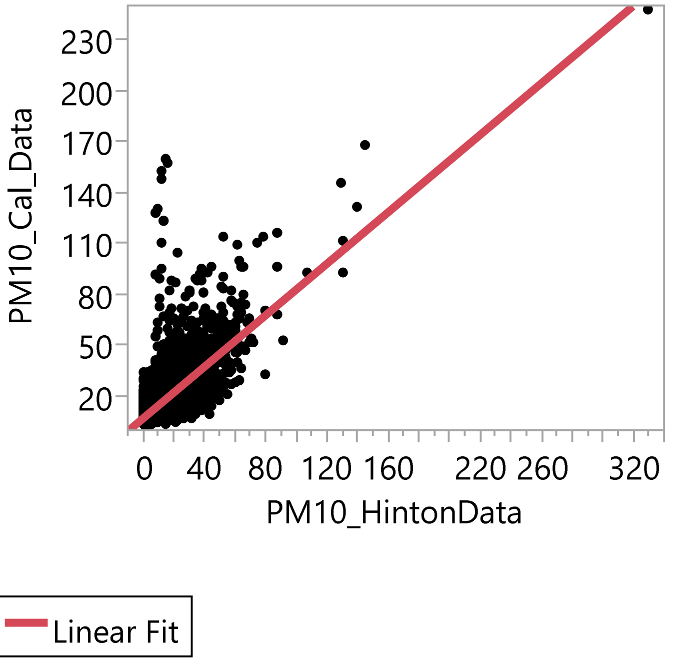

Linear Fit

PM10_Cal_Data = 7.9272309 + 0.7587557*PM10_HintonData

Underlying ANCOVA Results

Table S4. Results of ANCOVA F-tests for O_3 Raw - O_3 Hinton Effect Tests

Source	Nparm	DF	Sum of Squares	F Ratio	Prob > F
Device ID	11	11	2876574.5	5404.695	<.0001*
Temp	1	1	1121291.6	23174.31	<.0001*
RH	1	1	14951.4	309.0077	<.0001*
WD	1	1	3971.4	82.0796	<.0001*
WS	1	1	5107.4	105.5574	<.0001*
Device ID*Temp	11	11	350013.6	657.6283	<.0001*
Device ID*RH	11	11	487546.8	916.0346	<.0001*
Device ID*WD	11	11	4949.5	9.2994	<.0001*
Device ID*WS	11	11	179557.4	337.3641	<.0001*

Supplementary Material: Underlying ANCOVA results for Figure 2 (O3)

1. **Raw data**

1) **Temp**

Response O3 Raw-O3 Hinton

Regression Plot

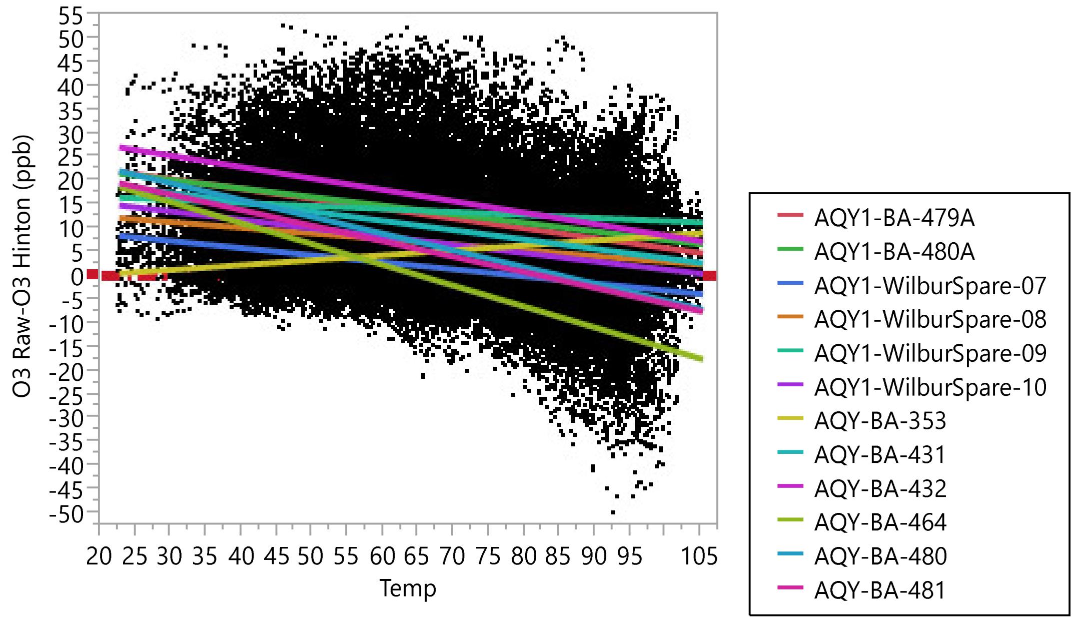

Summary of Fit

RSquare	0.417072
RSquare Adj	0.416963
Root Mean Square Error	7.412667
Mean of Response	8.262987
Observations (or Sum Wgts)	123097

Analysis of Variance

Source	DF	Sum of Squares	Mean Square	F Ratio
Model	23	4838460	210368	3828.516
Error	123073	6762569	55	**Prob > F**
C. Total	123096	11601030		<.0001*

Parameter Estimates

Term	Estimate	Std Error	t Ratio	Prob>|t|
Intercept	21.305079	0.093139	228.74	<.0001*
Device ID[AQY1-BA-479A]	4.4836435	0.066271	67.66	<.0001*
Device ID[AQY1-BA-480A]	5.378161	0.068335	78.70	<.0001*
Device ID[AQY1-WilburSpare-07]	-6.198739	0.073991	-83.78	<.0001*
Device ID[AQY1-WilburSpare-08]	-1.014969	0.06854	-14.81	<.0001*
Device ID[AQY1-WilburSpare-09]	5.8677332	0.067579	86.83	<.0001*
Device ID[AQY1-WilburSpare-10]	-1.021862	0.0665	-15.37	<.0001*
Device ID[AQY-BA-353]	-2.207162	0.071887	-30.70	<.0001*
Device ID[AQY-BA-431]	2.0292931	0.06677	30.39	<.0001*
Device ID[AQY-BA-432]	8.1078984	0.067404	120.29	<.0001*
Device ID[AQY-BA-464]	-9.64501	0.086704	-111.2	<.0001*
Device ID[AQY-BA-480]	-2.212342	0.075179	-29.43	<.0001*

Term	Estimate	Std Error	t Ratio	Prob>|t|
Temp	-0.195537	0.001292	-151.3	<.0001*
Device ID[AQY1-BA-479A]*(Temp-69.8635)	-0.011506	0.003993	-2.88	0.0040*
Device ID[AQY1-BA-480A]*(Temp-69.8635)	0.0148098	0.003994	3.71	0.0002*
Device ID[AQY1-WilburSpare-07]*(Temp-69.8635)	0.0461096	0.004351	10.60	<.0001*
Device ID[AQY1-WilburSpare-08]*(Temp-69.8635)	0.0768068	0.004182	18.37	<.0001*
Device ID[AQY1-WilburSpare-09]*(Temp-69.8635)	0.1319806	0.004099	32.20	<.0001*
Device ID[AQY1-WilburSpare-10]*(Temp-69.8635)	0.0207832	0.004022	5.17	<.0001*
Device ID[AQY-BA-353]*(Temp-69.8635)	0.2991603	0.004417	67.73	<.0001*
Device ID[AQY-BA-431]*(Temp-69.8635)	-0.000681	0.004015	-0.17	0.8653
Device ID[AQY-BA-432]*(Temp-69.8635)	-0.043977	0.004029	-10.92	<.0001*
Device ID[AQY-BA-464]*(Temp-69.8635)	-0.243679	0.005363	-45.44	<.0001*
Device ID[AQY-BA-480]*(Temp-69.8635)	-0.157442	0.00449	-35.06	<.0001*

Effect Tests

Source	Nparm	DF	Sum of Squares	F Ratio	Prob > F
Device ID	11	11	2862977.2	4736.704	<.0001*
Temp	1	1	1257862.7	22892.03	<.0001*
Device ID*Temp	11	11	521062.2	862.0807	<.0001*

2) RH

Response O3 Raw-O3 Hinton
Regression Plot

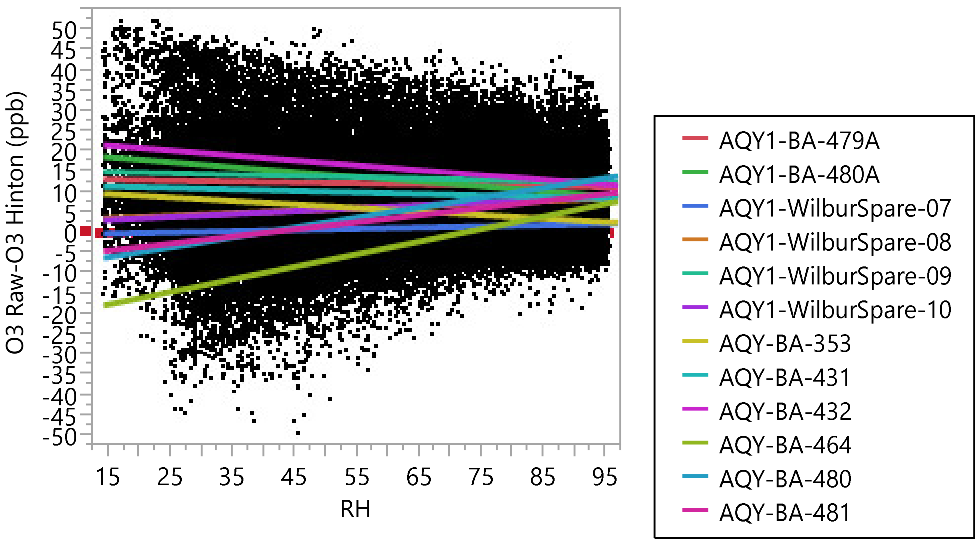

Summary of Fit

RSquare	0.333492
RSquare Adj	0.333367
Root Mean Square Error	7.925904
Mean of Response	8.262255
Observations (or Sum Wgts)	123117

Analysis of Variance

Source	DF	Sum of Squares	Mean Square	F Ratio
Model	23	3869101	168222	2677.840
Error	123093	7732696	63	**Prob > F**
C. Total	123116	11601797		<.0001*

Parameter Estimates

Term	Estimate	Std Error	t Ratio	Prob>|t|
Intercept	5.2391561	0.076112	68.83	<.0001*
Device ID[AQY1-BA-479A]	4.5476954	0.07083	64.21	<.0001*
Device ID[AQY1-BA-480A]	5.5878137	0.072961	76.59	<.0001*
Device ID[AQY1-WilburSpare-07]	-6.285821	0.079008	-79.56	<.0001*
Device ID[AQY1-WilburSpare-08]	-1.092078	0.07313	-14.93	<.0001*
Device ID[AQY1-WilburSpare-09]	6.0354329	0.071895	83.95	<.0001*
Device ID[AQY1-WilburSpare-10]	-0.968633	0.071089	-13.63	<.0001*
Device ID[AQY-BA-353]	-1.957569	0.076148	-25.71	<.0001*
Device ID[AQY-BA-431]	2.2065329	0.0713	30.95	<.0001*
Device ID[AQY-BA-432]	8.4798246	0.071782	118.13	<.0001*
Device ID[AQY-BA-464]	-10.69159	0.092063	-116.1	<.0001*
Device ID[AQY-BA-480]	-2.127572	0.080359	-26.48	<.0001*
RH	0.0386175	0.001181	32.69	<.0001*
Device ID[AQY1-BA-479A]*(RH-61.3915)	-0.061276	0.003636	-16.85	<.0001*
Device ID[AQY1-BA-480A]*(RH-61.3915)	-0.161242	0.00374	-43.12	<.0001*
Device ID[AQY1-WilburSpare-07]*(RH-61.3915)	-0.00855	0.004091	-2.09	0.0366*
Device ID[AQY1-WilburSpare-08]*(RH-61.3915)	0.0216811	0.003759	5.77	<.0001*
Device ID[AQY1-WilburSpare-09]*(RH-61.3915)	-0.072572	0.003696	-19.64	<.0001*
Device ID[AQY1-WilburSpare-10]*(RH-61.3915)	0.0330058	0.003679	8.97	<.0001*
Device ID[AQY-BA-353]*(RH-61.3915)	-0.124846	0.003949	-31.62	<.0001*
Device ID[AQY-BA-431]*(RH-61.3915)	-0.075845	0.003641	-20.83	<.0001*
Device ID[AQY-BA-432]*(RH-61.3915)	-0.161503	0.003645	-44.31	<.0001*
Device ID[AQY-BA-464]*(RH-61.3915)	0.270231	0.004813	56.14	<.0001*
Device ID[AQY-BA-480]*(RH-61.3915)	0.2049024	0.004177	49.05	<.0001*

Effect Tests

Source	Nparm	DF	Sum of Squares	F Ratio	Prob > F
Device ID	11	11	3160532.9	4573.725	<.0001*
RH	1	1	67145.0	1068.849	<.0001*
Device ID*RH	11	11	717654.8	1038.545	<.0001*

3) WD

Response O3 Raw-O3 Hinton
Regression Plot

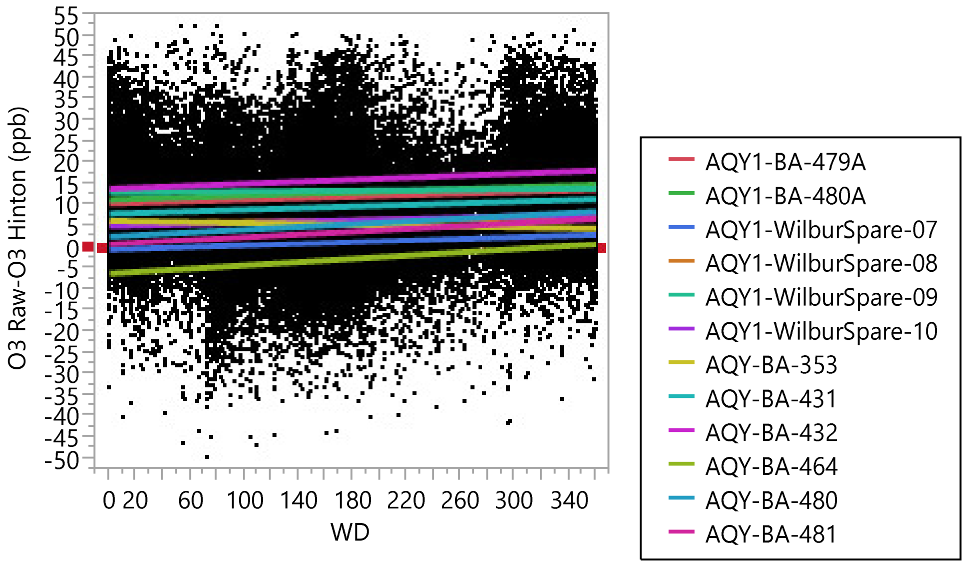

Summary of Fit

RSquare	0.279622
RSquare Adj	0.279487
Root Mean Square Error	8.244511
Mean of Response	8.262636
Observations (or Sum Wgts)	122877

Analysis of Variance

Source	DF	Sum of Squares	Mean Square	F Ratio
Model	23	3241352	140928	2073.330
Error	122853	8350560	68	**Prob > F**
C. Total	122876	11591911		<.0001*

Parameter Estimates

Term	Estimate	Std Error	t Ratio	Prob>|t|
Intercept	6.0162674	0.051755	116.25	<.0001*
Device ID[AQY1-BA-479A]	4.5097959	0.073769	61.13	<.0001*
Device ID[AQY1-BA-480A]	5.5366043	0.075999	72.85	<.0001*
Device ID[AQY1-WilburSpare-07]	-6.311776	0.082188	-76.80	<.0001*
Device ID[AQY1-WilburSpare-08]	-1.099488	0.076197	-14.43	<.0001*
Device ID[AQY1-WilburSpare-09]	5.9704249	0.074877	79.74	<.0001*
Device ID[AQY1-WilburSpare-10]	-1.033116	0.074029	-13.96	<.0001*
Device ID[AQY-BA-353]	-1.995433	0.079423	-25.12	<.0001*
Device ID[AQY-BA-431]	2.1908745	0.074254	29.51	<.0001*
Device ID[AQY-BA-432]	8.4767086	0.074766	113.38	<.0001*
Device ID[AQY-BA-464]	-10.43042	0.095845	-108.8	<.0001*
Device ID[AQY-BA-480]	-2.013055	0.083574	-24.09	<.0001*
WD	0.0093909	0.000267	35.19	<.0001*
Device ID[AQY1-BA-479A]*(WD-172.637)	-0.000334	0.000822	-0.41	0.6842
Device ID[AQY1-BA-480A]*(WD-172.637)	0.0003742	0.000846	0.44	0.6582
Device ID[AQY1-WilburSpare-07]*(WD-172.637)	0.000933	0.00093	1.00	0.3159
Device ID[AQY1-WilburSpare-08]*(WD-172.637)	-0.005038	0.000854	-5.90	<.0001*
Device ID[AQY1-WilburSpare-09]*(WD-172.637)	-0.006839	0.000827	-8.27	<.0001*
Device ID[AQY1-WilburSpare-10]*(WD-172.637)	-0.001686	0.000821	-2.05	0.0401*
Device ID[AQY-BA-353]*(WD-172.637)	-0.01443	0.000908	-15.90	<.0001*
Device ID[AQY-BA-431]*(WD-172.637)	0.0001634	0.000814	0.20	0.8408
Device ID[AQY-BA-432]*(WD-172.637)	0.002498	0.000816	3.06	0.0022*
Device ID[AQY-BA-464]*(WD-172.637)	0.0099389	0.001094	9.09	<.0001*
Device ID[AQY-BA-480]*(WD-172.637)	0.0070768	0.00094	7.53	<.0001*

Effect Tests

Source	Nparm	DF	Sum of Squares	F Ratio	Prob > F
Device ID	11	11	3106432.7	4154.698	<.0001*
WD	1	1	84148.8	1237.992	<.0001*
Device ID*WD	11	11	35437.5	47.3958	<.0001*

4) WS

Response O3 Raw-O3 Hinton

Regression Plot

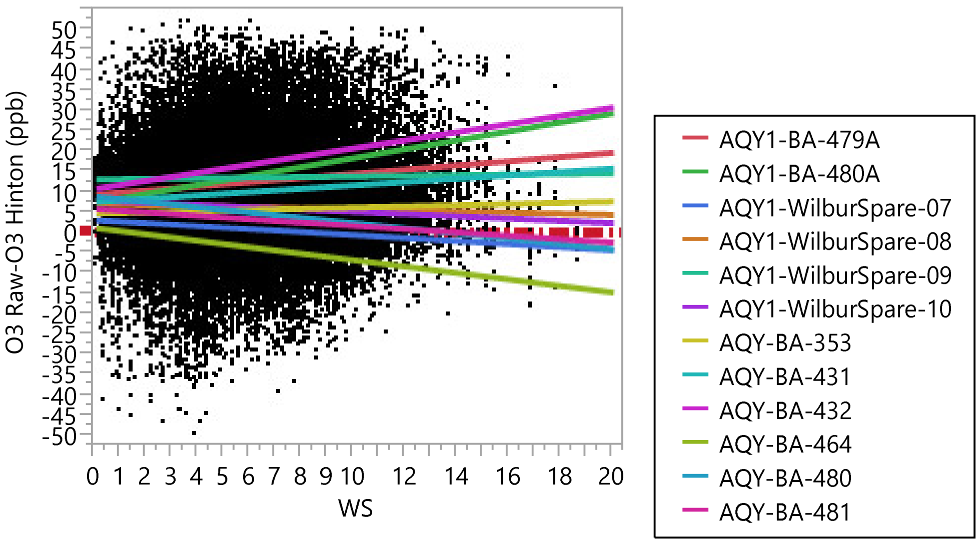

Summary of Fit

RSquare	0.293584
RSquare Adj	0.293452
Root Mean Square Error	8.164222
Mean of Response	8.262636
Observations (or Sum Wgts)	122877

Analysis of Variance

Source	DF	Sum of Squares	Mean Square	F Ratio
Model	23	3403204	147965	2219.885
Error	122853	8188708	67	**Prob > F**
C. Total	122876	11591911		<.0001*

Parameter Estimates

Term	Estimate	Std Error	t Ratio	Prob>|t|
Intercept	7.3676441	0.053636	137.36	<.0001*
Device ID[AQY1-BA-479A]	4.5392827	0.07305	62.14	<.0001*
Device ID[AQY1-BA-480A]	5.611063	0.075258	74.56	<.0001*
Device ID[AQY1-WilburSpare-07]	-6.356275	0.081465	-78.02	<.0001*
Device ID[AQY1-WilburSpare-08]	-1.085682	0.07546	-14.39	<.0001*
Device ID[AQY1-WilburSpare-09]	5.98803	0.074127	80.78	<.0001*
Device ID[AQY1-WilburSpare-10]	-1.007138	0.073304	-13.74	<.0001*
Device ID[AQY-BA-353]	-1.975655	0.078631	-25.13	<.0001*
Device ID[AQY-BA-431]	2.1999474	0.073533	29.92	<.0001*
Device ID[AQY-BA-432]	8.4703991	0.074037	114.41	<.0001*
Device ID[AQY-BA-464]	-10.54492	0.09482	-111.2	<.0001*
Device ID[AQY-BA-480]	-2.008212	0.082765	-24.26	<.0001*
WS	0.0486182	0.009181	5.30	<.0001*
Device ID[AQY1-BA-479A]*(WS-5.25906)	0.463997	0.028421	16.33	<.0001*
Device ID[AQY1-BA-480A]*(WS-5.25906)	1.0455901	0.029039	36.01	<.0001*
Device ID[AQY1-WilburSpare-07]*(WS-5.25906)	-0.422478	0.032297	-13.08	<.0001*
Device ID[AQY1-WilburSpare-08]*(WS-5.25906)	-0.18237	0.029051	-6.28	<.0001*
Device ID[AQY1-WilburSpare-09]*(WS-5.25906)	0.0273136	0.028258	0.97	0.3338
Device ID[AQY1-WilburSpare-10]*(WS-5.25906)	-0.325797	0.02828	-11.52	<.0001*
Device ID[AQY-BA-353]*(WS-5.25906)	0.0958139	0.030521	3.14	0.0017*
Device ID[AQY-BA-431]*(WS-5.25906)	0.3600283	0.028158	12.79	<.0001*
Device ID[AQY-BA-432]*(WS-5.25906)	0.9471076	0.028319	33.44	<.0001*
Device ID[AQY-BA-464]*(WS-5.25906)	-0.840312	0.037686	-22.30	<.0001*
Device ID[AQY-BA-480]*(WS-5.25906)	-0.701362	0.032363	-21.67	<.0001*

Effect Tests

Source	Nparm	DF	Sum of Squares	F Ratio	Prob > F
Device ID	11	11	3141335.7	4284.420	<.0001*
WS	1	1	1869.4	28.0456	<.0001*
Device ID*WS	11	11	265154.0	361.6395	<.0001*

2. Calibrated data

1) Temp

Response O3 Cal- O3 Hinton

Regression Plot

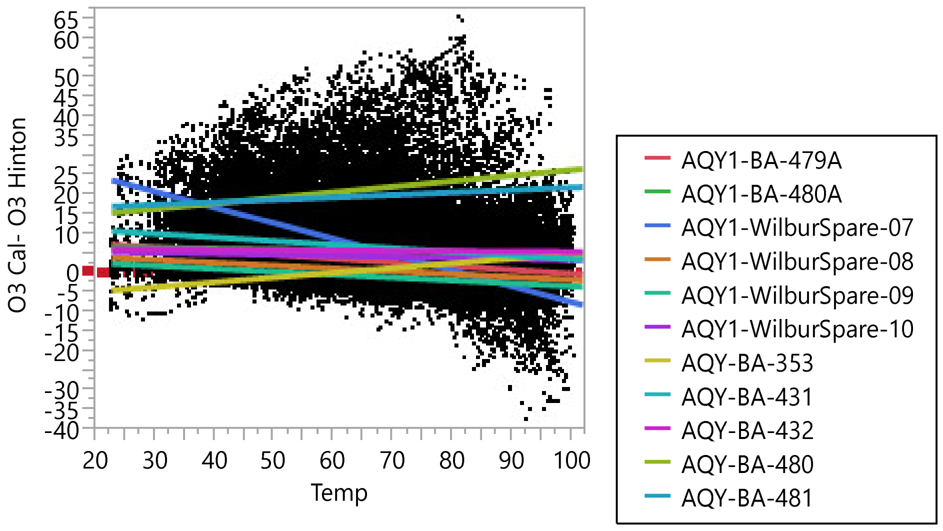

Summary of Fit

RSquare	0.510441
RSquare Adj	0.510317
Root Mean Square Error	6.323088
Mean of Response	5.973425
Observations (or Sum Wgts)	82646

Analysis of Variance

Source	DF	Sum of Squares	Mean Square	F Ratio
Model	21	3444336.2	164016	4102.303
Error	82624	3303426.7	40	**Prob > F**
C. Total	82645	6747763.0		<.0001*

Parameter Estimates

Term	Estimate	Std Error	t Ratio	Prob>|t|
Intercept	9.6942937	0.103403	93.75	<.0001*
Device ID[AQY1-BA-479A]	-3.644404	0.063391	-57.49	<.0001*
Device ID[AQY1-BA-480A]	-1.020294	0.065634	-15.55	<.0001*
Device ID[AQY1-WilburSpare-07]	-0.782828	0.093594	-8.36	<.0001*
Device ID[AQY1-WilburSpare-08]	-5.979466	0.087055	-68.69	<.0001*
Device ID[AQY1-WilburSpare-09]	-7.585273	0.062397	-121.6	<.0001*
Device ID[AQY1-WilburSpare-10]	-2.438096	0.063675	-38.29	<.0001*
Device ID[AQY-BA-353]	-5.731413	0.093946	-61.01	<.0001*
Device ID[AQY-BA-431]	-0.073687	0.06386	-1.15	0.2486
Device ID[AQY-BA-432]	-0.962458	0.063688	-15.11	<.0001*
Device ID[AQY-BA-480]	15.039004	0.088404	170.12	<.0001*
Temp	-0.042686	0.001496	-28.53	<.0001*
Device ID[AQY1-BA-479A]*(Temp-66.9776)	-0.053025	0.003961	-13.39	<.0001*
Device ID[AQY1-BA-480A]*(Temp-66.9776)	0.0212657	0.003991	5.33	<.0001*
Device ID[AQY1-WilburSpare-07]*(Temp-66.9776)	-0.360766	0.005238	-68.88	<.0001*
Device ID[AQY1-WilburSpare-08]*(Temp-66.9776)	-0.029993	0.005207	-5.76	<.0001*
Device ID[AQY1-WilburSpare-09]*(Temp-66.9776)	-0.03159	0.003899	-8.10	<.0001*
Device ID[AQY1-WilburSpare-10]*(Temp-66.9776)	0.0162509	0.00399	4.07	<.0001*
Device ID[AQY-BA-353]*(Temp-66.9776)	0.1636137	0.005505	29.72	<.0001*
Device ID[AQY-BA-431]*(Temp-66.9776)	-0.049534	0.004014	-12.34	<.0001*
Device ID[AQY-BA-432]*(Temp-66.9776)	0.0342781	0.003979	8.61	<.0001*
Device ID[AQY-BA-480]*(Temp-66.9776)	0.1842884	0.005773	31.92	<.0001*

Effect Tests

Source	Nparm	DF	Sum of Squares	F Ratio	Prob > F
Device ID	10	10	2805095.3	7015.993	<.0001*
Temp	1	1	32549.1	814.1058	<.0001*
Device ID*Temp	10	10	267051.8	667.9393	<.0001*

2) **RH**

Response O3 Cal- O3 Hinton

Regression Plot

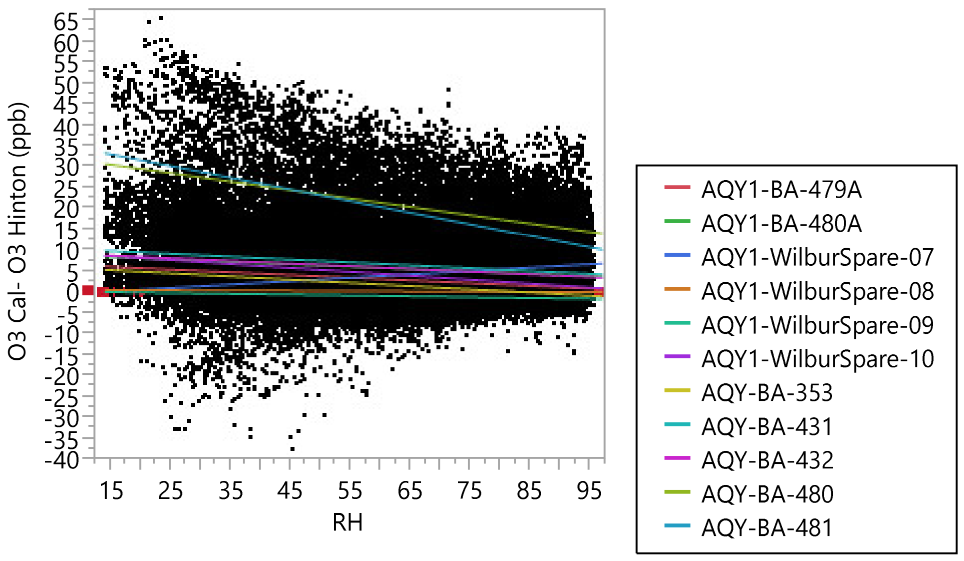

Summary of Fit

RSquare	0.521577
RSquare Adj	0.521455
Root Mean Square Error	6.250592
Mean of Response	5.972863
Observations (or Sum Wgts)	82654

Analysis of Variance

Source	DF	Sum of Squares	Mean Square	F Ratio
Model	21	3519624.6	167601	4289.777
Error	82632	3228424.5	39	**Prob > F**
C. Total	82653	6748049.0		<.0001*

Parameter Estimates

Term	Estimate	Std Error	t Ratio	Prob>|t|
Intercept	11.451992	0.075663	151.35	<.0001*
Device ID[AQY1-BA-479A]	-3.486123	0.062189	-56.06	<.0001*
Device ID[AQY1-BA-480A]	-0.789762	0.064429	-12.26	<.0001*
Device ID[AQY1-WilburSpare-07]	-2.464492	0.089219	-27.62	<.0001*
Device ID[AQY1-WilburSpare-08]	-6.256239	0.080407	-77.81	<.0001*
Device ID[AQY1-WilburSpare-09]	-7.398257	0.06122	-120.8	<.0001*
Device ID[AQY1-WilburSpare-10]	-2.289874	0.062443	-36.67	<.0001*
Device ID[AQY-BA-353]	-4.806339	0.084633	-56.79	<.0001*
Device ID[AQY-BA-431]	0.1376116	0.062664	2.20	0.0281*
Device ID[AQY-BA-432]	-0.751709	0.062138	-12.10	<.0001*
Device ID[AQY-BA-480]	14.641945	0.07894	185.48	<.0001*
RH	-0.077618	0.001172	-66.21	<.0001*
Device ID[AQY1-BA-479A]*(RH-61.8924)	0.0123889	0.003165	3.91	<.0001*
Device ID[AQY1-BA-480A]*(RH-61.8924)	0.0197245	0.003271	6.03	<.0001*
Device ID[AQY1-WilburSpare-07]*(RH-61.8924)	0.1589718	0.004766	33.35	<.0001*
Device ID[AQY1-WilburSpare-08]*(RH-61.8924)	0.0712091	0.004234	16.82	<.0001*
Device ID[AQY1-WilburSpare-09]*(RH-61.8924)	0.0576355	0.003118	18.48	<.0001*
Device ID[AQY1-WilburSpare-10]*(RH-61.8924)	-0.016748	0.003206	-5.22	<.0001*
Device ID[AQY-BA-353]*(RH-61.8924)	0.0008913	0.004504	0.20	0.8431
Device ID[AQY-BA-431]*(RH-61.8924)	0.0090042	0.003168	2.84	0.0045*
Device ID[AQY-BA-432]*(RH-61.8924)	0.0132372	0.003107	4.26	<.0001*
Device ID[AQY-BA-480]*(RH-61.8924)	-0.124126	0.003863	-32.13	<.0001*

Effect Tests

Source	Nparm	DF	Sum of Squares	F Ratio	Prob > F
Device ID	10	10	3250888.2	8320.696	<.0001*
RH	1	1	171272.0	4383.731	<.0001*
Device ID*RH	10	10	197025.8	504.2904	<.0001*

3) WD

Response O3 Cal- O3 Hinton

Regression Plot

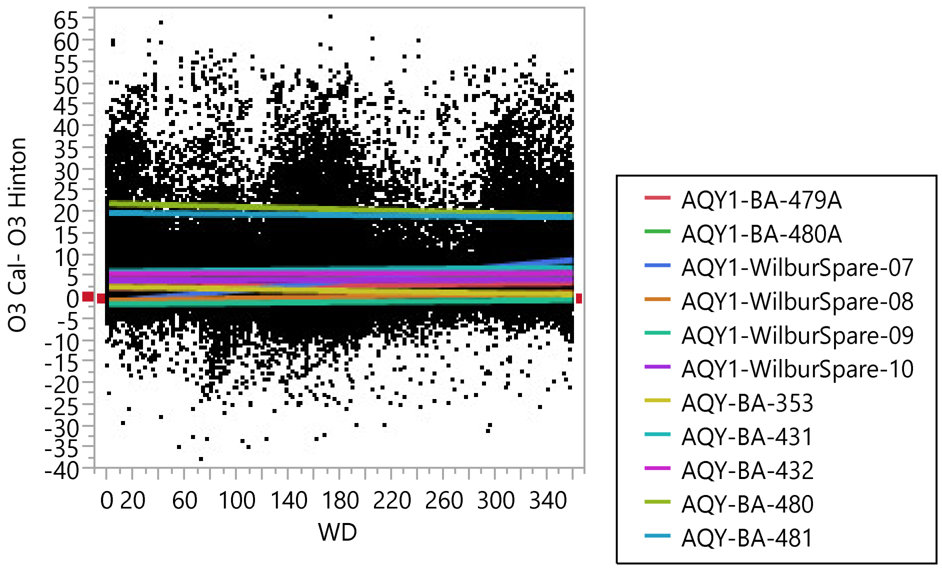

Summary of Fit

RSquare	0.467637
RSquare Adj	0.467501
Root Mean Square Error	6.599067
Mean of Response	5.980496
Observations (or Sum Wgts)	82430

Analysis of Variance

Source	DF	Sum of Squares	Mean Square	F Ratio
Model	21	3152356.1	150112	3447.076
Error	82408	3588677.8	44	**Prob > F**
C. Total	82429	6741033.9		<.0001*

Parameter Estimates

Term	Estimate	Std Error	t Ratio	Prob>|t|
Intercept	6.1647627	0.052267	117.95	<.0001*
Device ID[AQY1-BA-479A]	-3.436656	0.06574	-52.28	<.0001*
Device ID[AQY1-BA-480A]	-0.748963	0.068103	-11.00	<.0001*
Device ID[AQY1-WilburSpare-07]	-2.456249	0.093827	-26.18	<.0001*
Device ID[AQY1-WilburSpare-08]	-6.109193	0.084949	-71.92	<.0001*
Device ID[AQY1-WilburSpare-09]	-7.34055	0.064717	-113.4	<.0001*
Device ID[AQY1-WilburSpare-10]	-2.20814	0.066011	-33.45	<.0001*
Device ID[AQY-BA-353]	-4.650835	0.089868	-51.75	<.0001*
Device ID[AQY-BA-431]	0.2185811	0.066249	3.30	0.0010*
Device ID[AQY-BA-432]	-0.692083	0.06569	-10.54	<.0001*
Device ID[AQY-BA-480]	14.39276	0.083285	172.81	<.0001*
WD	0.0023377	0.000263	8.89	<.0001*
Device ID[AQY1-BA-479A]*(WD-177.784)	0.0005647	0.000711	0.79	0.4269
Device ID[AQY1-BA-480A]*(WD-177.784)	-0.002666	0.000734	-3.63	0.0003*
Device ID[AQY1-WilburSpare-07]*(WD-177.784)	0.0248261	0.001073	23.13	<.0001*
Device ID[AQY1-WilburSpare-08]*(WD-177.784)	0.0026668	0.000963	2.77	0.0056*
Device ID[AQY1-WilburSpare-09]*(WD-177.784)	0.0004082	0.000697	0.59	0.5579
Device ID[AQY1-WilburSpare-10]*(WD-177.784)	-0.001734	0.00071	-2.44	0.0146*
Device ID[AQY-BA-353]*(WD-177.784)	-0.007276	0.001043	-6.97	<.0001*
Device ID[AQY-BA-431]*(WD-177.784)	0.0001258	0.000703	0.18	0.8579
Device ID[AQY-BA-432]*(WD-177.784)	-0.001919	0.000691	-2.78	0.0055*
Device ID[AQY-BA-480]*(WD-177.784)	-0.009849	0.000845	-11.66	<.0001*

Effect Tests

Source	Nparm	DF	Sum of Squares	F Ratio	Prob > F
Device ID	10	10	3115552.6	7154.347	<.0001*
WD	1	1	3444.2	79.0911	<.0001*
Device ID*WD	10	10	29937.1	68.7456	<.0001*

4) WS

Response O3 Cal- O3 Hinton

Regression Plot

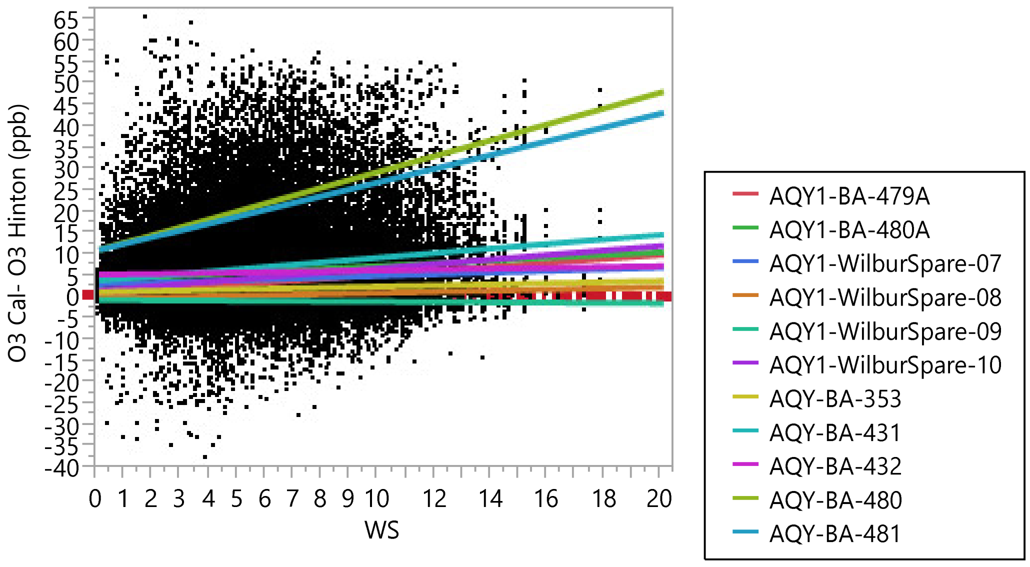

Summary of Fit

RSquare	0.509886
RSquare Adj	0.509761
Root Mean Square Error	6.331799
Mean of Response	5.980496
Observations (or Sum Wgts)	82430

Analysis of Variance

Source	DF	Sum of Squares	Mean Square	F Ratio
Model	21	3437158.9	163674	4082.499
Error	82408	3303875.0	40	**Prob > F**
C. Total	82429	6741033.9		<.0001*

Parameter Estimates

Term	Estimate	Std Error	t Ratio	Prob>|t|
Intercept	3.7503998	0.05182	72.37	<.0001*
Device ID[AQY1-BA-479A]	-3.397357	0.063082	-53.86	<.0001*
Device ID[AQY1-BA-480A]	-0.702489	0.065351	-10.75	<.0001*
Device ID[AQY1-WilburSpare-07]	-2.427532	0.091383	-26.56	<.0001*
Device ID[AQY1-WilburSpare-08]	-6.099527	0.081324	-75.00	<.0001*
Device ID[AQY1-WilburSpare-09]	-7.306463	0.062102	-117.7	<.0001*
Device ID[AQY1-WilburSpare-10]	-2.186582	0.063342	-34.52	<.0001*
Device ID[AQY-BA-353]	-4.56804	0.085547	-53.40	<.0001*
Device ID[AQY-BA-431]	0.2241248	0.063579	3.53	0.0004*
Device ID[AQY-BA-432]	-0.667684	0.063059	-10.59	<.0001*
Device ID[AQY-BA-480]	14.21791	0.079707	178.38	<.0001*
WS	0.5298291	0.008871	59.73	<.0001*
Device ID[AQY1-BA-479A]*(WS-5.27943)	-0.067089	0.02416	-2.78	0.0055*
Device ID[AQY1-BA-480A]*(WS-5.27943)	-0.207033	0.024762	-8.36	<.0001*
Device ID[AQY1-WilburSpare-07]*(WS-5.27943)	-0.332742	0.037208	-8.94	<.0001*
Device ID[AQY1-WilburSpare-08]*(WS-5.27943)	-0.395455	0.031777	-12.44	<.0001*
Device ID[AQY1-WilburSpare-09]*(WS-5.27943)	-0.569036	0.023491	-24.22	<.0001*
Device ID[AQY1-WilburSpare-10]*(WS-5.27943)	-0.006855	0.024027	-0.29	0.7754
Device ID[AQY-BA-353]*(WS-5.27943)	-0.402187	0.033346	-12.06	<.0001*
Device ID[AQY-BA-431]*(WS-5.27943)	0.0092777	0.023911	0.39	0.6980
Device ID[AQY-BA-432]*(WS-5.27943)	-0.429414	0.023494	-18.28	<.0001*
Device ID[AQY-BA-480]*(WS-5.27943)	1.3233335	0.029156	45.39	<.0001*

Effect Tests

Source	Nparm	DF	Sum of Squares	F Ratio	Prob > F
Device ID	10	10	3071221.5	7660.496	<.0001*
WS	1	1	143021.9	3567.370	<.0001*
Device ID*WS	10	10	174765.1	435.9138	<.0001*

Table S5. Results of ANCOVA F-tests for NO_2 Raw – NO_2 Hinton

Source	Nparm	DF	Sum of Squares	F Ratio	Prob > F
Device ID	11	11	3143614.8	1316.078	<.0001*
Temp	1	1	906267.1	4173.507	<.0001*
RH	1	1	159131.9	732.8284	<.0001*
WD	1	1	16195.7	74.5837	<.0001*
WS	1	1	3368.2	15.5111	<.0001*
Device ID*Temp	11	11	2356271.8	986.4557	<.0001*
Device ID*RH	11	11	442532.1	185.2665	<.0001*
Device ID*WD	11	11	24798.2	10.3818	<.0001*
Device ID*WS	11	11	401289.8	168.0004	<.0001*

Supplementary Material: Underlying ANCOVA results for Figure 3 (NO2)

1. **Raw data**

1) **Temp**

Response NO2 Raw-NO2 Hinton
Regression Plot

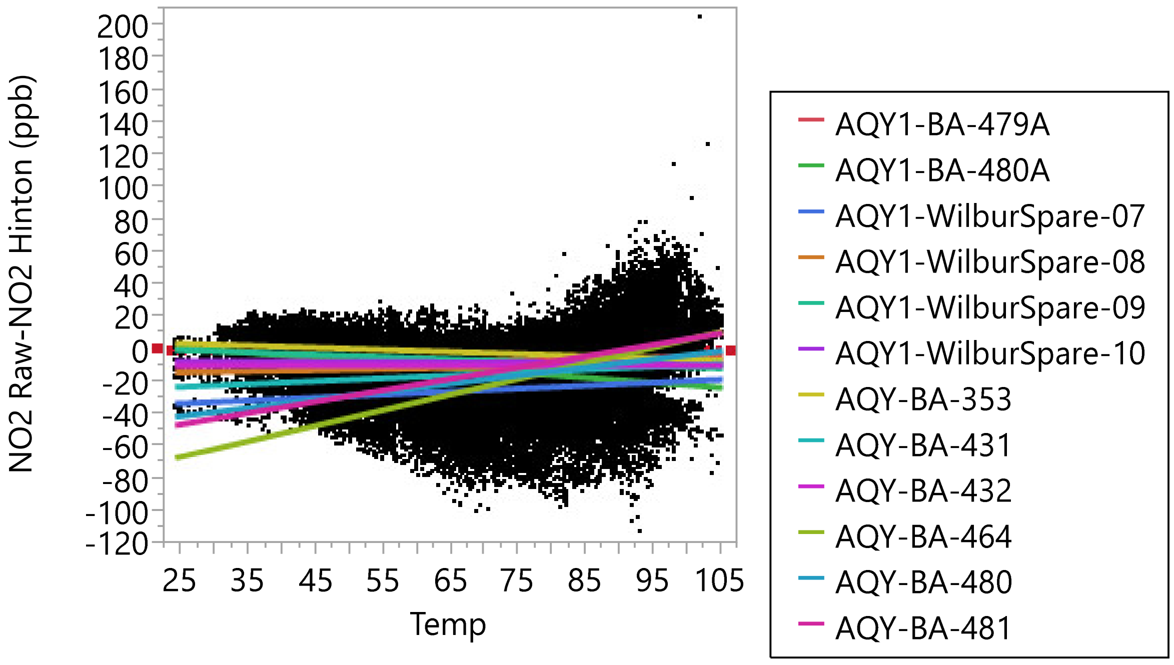

Summary of Fit

RSquare	0.199743
RSquare Adj	0.199564
Root Mean Square Error	15.08773
Mean of Response	-11.2788
Observations (or Sum Wgts)	102571

Analysis of Variance

Source	DF	Sum of Squares	Mean Square	F Ratio
Model	23	5826583	253330	1112.855
Error	102547	23343750	228	**Prob > F**
C. Total	102570	29170334		<.0001*

Parameter Estimates

Term	Estimate	Std Error	t Ratio	Prob>|t|
Intercept	-24.62224	0.233612	-105.4	<.0001*
Device ID[AQY1-BA-479A]	5.5264083	0.146669	37.68	<.0001*
Device ID[AQY1-BA-480A]	-1.662323	0.154889	-10.73	<.0001*
Device ID[AQY1-WilburSpare-07]	-12.16521	0.162688	-74.78	<.0001*
Device ID[AQY1-WilburSpare-08]	1.4418835	0.154535	9.33	<.0001*
Device ID[AQY1-WilburSpare-09]	4.9673465	0.152987	32.47	<.0001*
Device ID[AQY1-WilburSpare-10]	4.1621302	0.146669	28.38	<.0001*
Device ID[AQY-BA-353]	9.6661627	0.165331	58.47	<.0001*
Device ID[AQY-BA-431]	-2.265689	0.149413	-15.16	<.0001*
Device ID[AQY-BA-432]	2.2985465	0.152397	15.08	<.0001*

Term	Estimate	Std Error	t Ratio	Prob>|t|
Device ID[AQY-BA-464]	-7.365876	0.191891	-38.39	<.0001*
Device ID[AQY-BA-480]	-4.61797	0.166516	-27.73	<.0001*
Temp	0.1729736	0.003098	55.84	<.0001*
Device ID[AQY1-BA-479A]*(Temp-73.2653)	-0.099126	0.009208	-10.77	<.0001*
Device ID[AQY1-BA-480A]*(Temp-73.2653)	-0.478517	0.009407	-50.87	<.0001*
Device ID[AQY1-WilburSpare-07]*(Temp-73.2653)	0.0107389	0.010348	1.04	0.2994
Device ID[AQY1-WilburSpare-08]*(Temp-73.2653)	-0.103873	0.010203	-10.18	<.0001*
Device ID[AQY1-WilburSpare-09]*(Temp-73.2653)	-0.313739	0.009592	-32.71	<.0001*
Device ID[AQY1-WilburSpare-10]*(Temp-73.2653)	-0.188806	0.009208	-20.51	<.0001*
Device ID[AQY-BA-353]*(Temp-73.2653)	-0.30059	0.011384	-26.40	<.0001*
Device ID[AQY-BA-431]*(Temp-73.2653)	0.0042433	0.009305	0.46	0.6484
Device ID[AQY-BA-432]*(Temp-73.2653)	-0.172386	0.009439	-18.26	<.0001*
Device ID[AQY-BA-464]*(Temp-73.2653)	0.7880982	0.013453	58.58	<.0001*
Device ID[AQY-BA-480]*(Temp-73.2653)	0.3266757	0.010683	30.58	<.0001*

Effect Tests

Source	Nparm	DF	Sum of Squares	F Ratio	Prob > F
Device ID	11	11	3144044.4	1255.591	<.0001*
Temp	1	1	709761.1	3117.917	<.0001*
Device ID*Temp	11	11	2508332.2	1001.716	<.0001*

2) RH

Response NO2 Raw-NO2 Hinton
Regression Plot

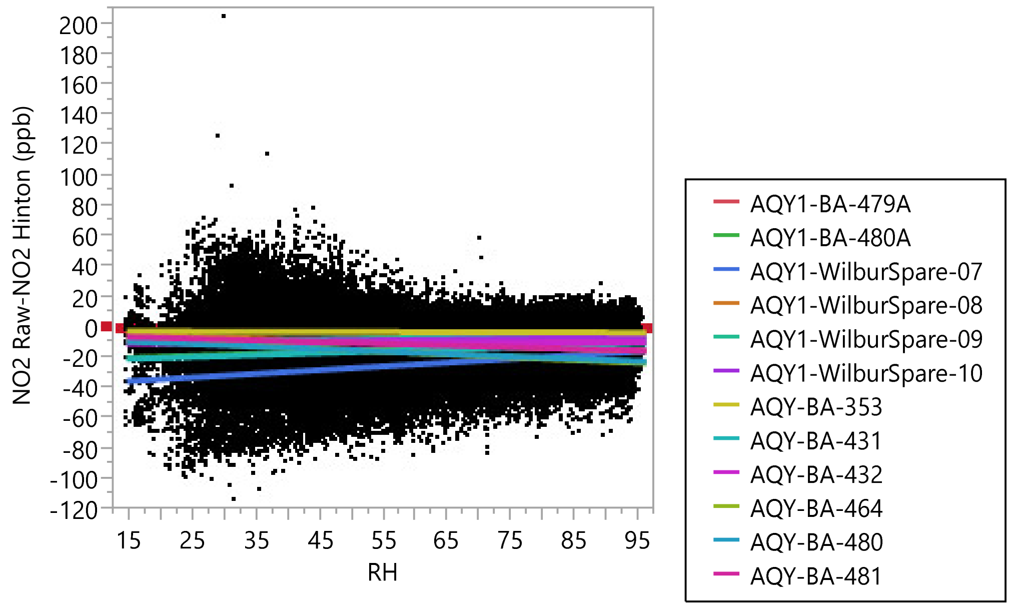

Summary of Fit

RSquare	0.118157
RSquare Adj	0.117959
Root Mean Square Error	15.83763
Mean of Response	-11.2781
Observations (or Sum Wgts)	102583

Analysis of Variance

Source	DF	Sum of Squares	Mean Square	F Ratio
Model	23	3446854	149863	597.4681
Error	102559	25724925	251	**Prob > F**
C. Total	102582	29171780		<.0001*

Parameter Estimates

Term	Estimate	Std Error	t Ratio	Prob>|t|
Intercept	-11.94002	0.169177	-70.58	<.0001*
Device ID[AQY1-BA-479A]	5.1093721	0.153591	33.27	<.0001*
Device ID[AQY1-BA-480A]	-1.776478	0.16229	-10.95	<.0001*
Device ID[AQY1-WilburSpare-07]	-12.14757	0.170056	-71.43	<.0001*
Device ID[AQY1-WilburSpare-08]	1.1299114	0.161646	6.99	<.0001*
Device ID[AQY1-WilburSpare-09]	4.8829708	0.159245	30.66	<.0001*
Device ID[AQY1-WilburSpare-10]	3.830562	0.153591	24.94	<.0001*
Device ID[AQY-BA-353]	8.9137361	0.169731	52.52	<.0001*
Device ID[AQY-BA-431]	-2.822152	0.156222	-18.06	<.0001*
Device ID[AQY-BA-432]	1.950643	0.159055	12.26	<.0001*
Device ID[AQY-BA-464]	-4.898562	0.19753	-24.80	<.0001*
Device ID[AQY-BA-480]	-4.776922	0.174881	-27.32	<.0001*
RH	0.0056074	0.002661	2.11	0.0351*
Device ID[AQY1-BA-479A]*(RH-60.6871)	0.0489051	0.008066	6.06	<.0001*

Term	Estimate	Std Error	t Ratio	Prob>|t|
Device ID[AQY1-BA-480A]*(RH-60.6871)	0.130821	0.008517	15.36	<.0001*
Device ID[AQY1-WilburSpare-07]*(RH-60.6871)	0.2373471	0.009155	25.93	<.0001*
Device ID[AQY1-WilburSpare-08]*(RH-60.6871)	-0.1382	0.008525	-16.21	<.0001*
Device ID[AQY1-WilburSpare-09]*(RH-60.6871)	0.0450909	0.008356	5.40	<.0001*
Device ID[AQY1-WilburSpare-10]*(RH-60.6871)	0.0473292	0.008066	5.87	<.0001*
Device ID[AQY-BA-353]*(RH-60.6871)	-0.015535	0.009083	-1.71	0.0872
Device ID[AQY-BA-431]*(RH-60.6871)	0.1234008	0.008149	15.14	<.0001*
Device ID[AQY-BA-432]*(RH-60.6871)	-0.001093	0.008268	-0.13	0.8949
Device ID[AQY-BA-464]*(RH-60.6871)	-0.191958	0.010863	-17.67	<.0001*
Device ID[AQY-BA-480]*(RH-60.6871)	-0.168483	0.009449	-17.83	<.0001*

Effect Tests

Source	Nparm	DF	Sum of Squares	F Ratio	Prob > F
Device ID	11	11	2869269.3	1039.916	<.0001*
RH	1	1	1113.7	4.4399	0.0351*
Device ID*RH	11	11	523274.7	189.6517	<.0001*

3) WD

Response NO2 Raw-NO2 Hinton

Regression Plot

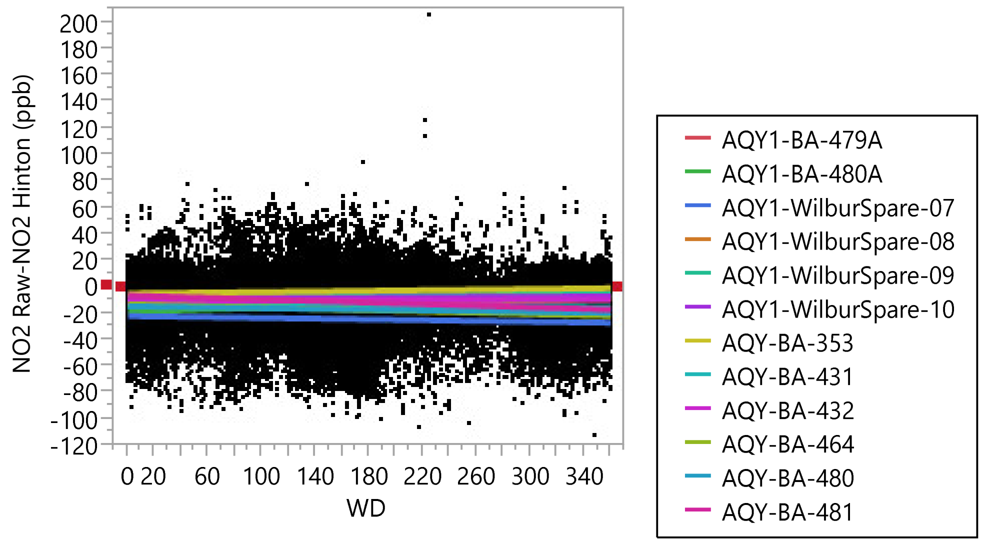

Summary of Fit

RSquare	0.105718
RSquare Adj	0.105518
Root Mean Square Error	15.94947
Mean of Response	-11.2788
Observations (or Sum Wgts)	102571

Analysis of Variance

Source	DF	Sum of Squares	Mean Square	F Ratio
Model	23	3083841	134080	527.0739
Error	102547	26086492	254	**Prob > F**
C. Total	102570	29170334		<.0001*

Parameter Estimates

Term	Estimate	Std Error	t Ratio	Prob>|t|
Intercept	-11.46824	0.11138	-103.0	<.0001*
Device ID[AQY1-BA-479A]	5.131838	0.154714	33.17	<.0001*
Device ID[AQY1-BA-480A]	-1.76802	0.16347	-10.82	<.0001*
Device ID[AQY1-WilburSpare-07]	-12.23331	0.171245	-71.44	<.0001*
Device ID[AQY1-WilburSpare-08]	1.2310474	0.162878	7.56	<.0001*
Device ID[AQY1-WilburSpare-09]	4.9162386	0.160376	30.65	<.0001*
Device ID[AQY1-WilburSpare-10]	3.8521274	0.154714	24.90	<.0001*
Device ID[AQY-BA-353]	9.0200271	0.171367	52.64	<.0001*
Device ID[AQY-BA-431]	-2.821567	0.157347	-17.93	<.0001*
Device ID[AQY-BA-432]	1.9754062	0.160193	12.33	<.0001*
Device ID[AQY-BA-464]	-5.08539	0.199372	-25.51	<.0001*
Device ID[AQY-BA-480]	-4.866523	0.175983	-27.65	<.0001*
WD	-0.000986	0.000599	-1.65	0.0998
Device ID[AQY1-BA-479A]*(WD-166.572)	0.0057027	0.001802	3.16	0.0016*
Device ID[AQY1-BA-480A]*(WD-166.572)	0.029773	0.001926	15.46	<.0001*
Device ID[AQY1-WilburSpare-07]*(WD-166.572)	-0.013253	0.002062	-6.43	<.0001*
Device ID[AQY1-WilburSpare-08]*(WD-166.572)	0.0081078	0.001942	4.18	<.0001*
Device ID[AQY1-WilburSpare-09]*(WD-166.572)	0.0128853	0.001859	6.93	<.0001*
Device ID[AQY1-WilburSpare-10]*(WD-166.572)	0.0062396	0.001802	3.46	0.0005*
Device ID[AQY-BA-353]*(WD-166.572)	0.0120132	0.002121	5.66	<.0001*
Device ID[AQY-BA-431]*(WD-166.572)	-0.001519	0.001809	-0.84	0.4008
Device ID[AQY-BA-432]*(WD-166.572)	0.00474	0.001838	2.58	0.0099*
Device ID[AQY-BA-464]*(WD-166.572)	-0.02343	0.002458	-9.53	<.0001*
Device ID[AQY-BA-480]*(WD-166.572)	-0.012527	0.002101	-5.96	<.0001*

Effect Tests

Source	Nparm	DF	Sum of Squares	F Ratio	Prob > F
Device ID	11	11	2924034.2	1044.954	<.0001*
WD	1	1	688.9	2.7080	0.0998
Device ID*WD	11	11	169720.7	60.6526	<.0001*

4) WS

Response NO2 Raw-NO2 Hinton

Regression Plot

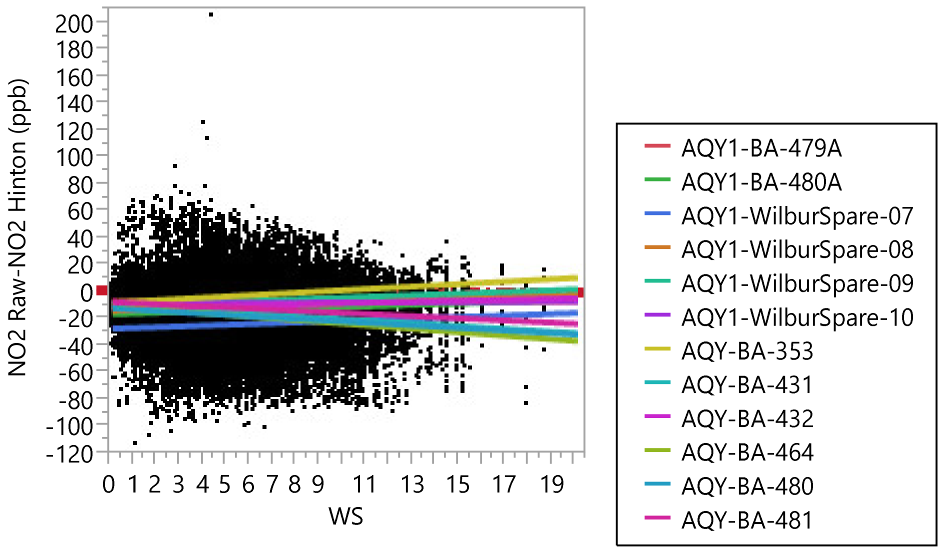

Summary of Fit

RSquare	0.11086
RSquare Adj	0.11066
Root Mean Square Error	15.90356
Mean of Response	-11.2788
Observations (or Sum Wgts)	102571

Analysis of Variance

Source	DF	Sum of Squares	Mean Square	F Ratio
Model	23	3233815	140601	555.9025
Error	102547	25936519	253	**Prob > F**
C. Total	102570	29170334		<.0001*

Parameter Estimates

Term	Estimate	Std Error	t Ratio	Prob>|t|
Intercept	-11.45648	0.114544	-100.0	<.0001*
Device ID[AQY1-BA-479A]	5.148058	0.154246	33.38	<.0001*
Device ID[AQY1-BA-480A]	-1.683471	0.163023	-10.33	<.0001*
Device ID[AQY1-WilburSpare-07]	-12.13519	0.17097	-70.98	<.0001*
Device ID[AQY1-WilburSpare-08]	1.1707637	0.162373	7.21	<.0001*
Device ID[AQY1-WilburSpare-09]	4.9332021	0.159826	30.87	<.0001*
Device ID[AQY1-WilburSpare-10]	3.867097	0.154246	25.07	<.0001*
Device ID[AQY-BA-353]	8.8601907	0.170526	51.96	<.0001*
Device ID[AQY-BA-431]	-2.786634	0.156878	-17.76	<.0001*
Device ID[AQY-BA-432]	1.975777	0.159723	12.37	<.0001*
Device ID[AQY-BA-464]	-4.96962	0.198331	-25.06	<.0001*
Device ID[AQY-BA-480]	-4.957081	0.175488	-28.25	<.0001*
WS	-0.036145	0.020208	-1.79	0.0737
Device ID[AQY1-BA-479A]*(WS-5.10425)	0.0240235	0.061022	0.39	0.6938
Device ID[AQY1-BA-480A]*(WS-5.10425)	0.5673515	0.065065	8.72	<.0001*
Device ID[AQY1-WilburSpare-07]*(WS-5.10425)	0.6156918	0.071184	8.65	<.0001*
Device ID[AQY1-WilburSpare-08]*(WS-5.10425)	0.6446613	0.064424	10.01	<.0001*
Device ID[AQY1-WilburSpare-09]*(WS-5.10425)	0.6431479	0.062543	10.28	<.0001*
Device ID[AQY1-WilburSpare-10]*(WS-5.10425)	0.1136411	0.061022	1.86	0.0626
Device ID[AQY-BA-353]*(WS-5.10425)	0.9567827	0.068514	13.96	<.0001*
Device ID[AQY-BA-431]*(WS-5.10425)	-1.012353	0.061787	-16.38	<.0001*
Device ID[AQY-BA-432]*(WS-5.10425)	0.396955	0.062854	6.32	<.0001*
Device ID[AQY-BA-464]*(WS-5.10425)	-1.251834	0.081448	-15.37	<.0001*
Device ID[AQY-BA-480]*(WS-5.10425)	-0.929429	0.071875	-12.93	<.0001*

Effect Tests

Source	Nparm	DF	Sum of Squares	F Ratio	Prob > F
Device ID	11	11	2880570.3	1035.374	<.0001*
WS	1	1	809.2	3.1993	0.0737
Device ID*WS	11	11	319783.8	114.9410	<.0001*

2. Calibrated data

1) Temp

Response NO2 Cal-NO2 Hinton

Regression Plot

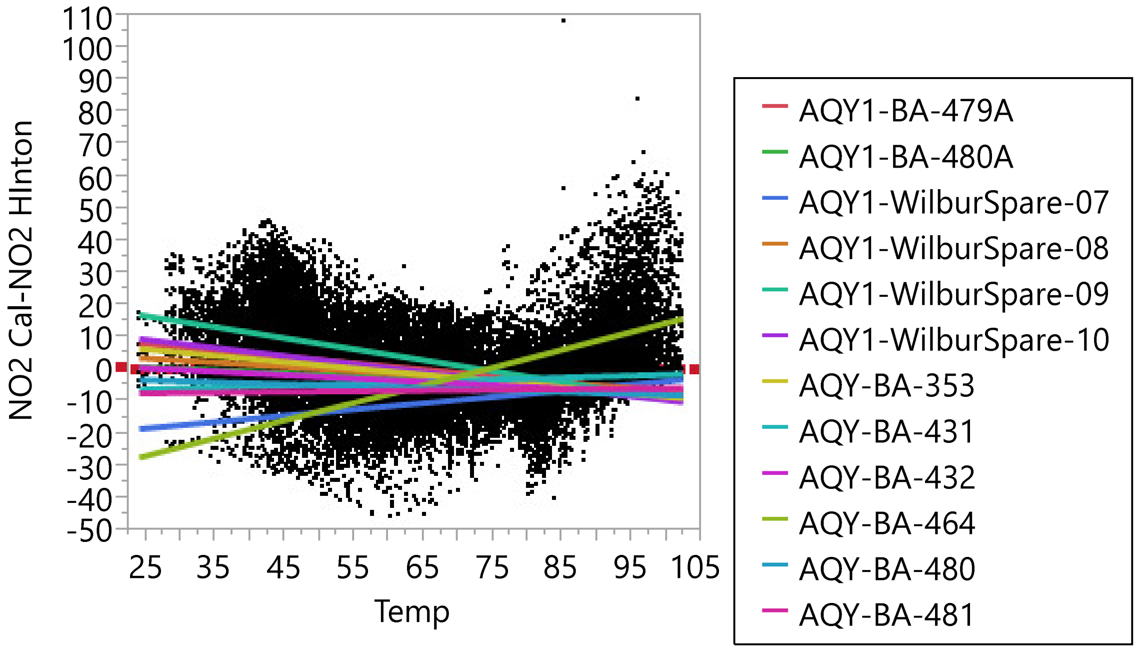

Summary of Fit

RSquare	0.213303
RSquare Adj	0.213051
Root Mean Square Error	8.437835
Mean of Response	-2.71148
Observations (or Sum Wgts)	71907

Analysis of Variance

Source	DF	Sum of Squares	Mean Square	F Ratio
Model	23	1387640.2	60332.2	847.3971
Error	71883	5117858.2	71.2	**Prob > F**
C. Total	71906	6505498.5		<.0001*

Parameter Estimates

Term	Estimate	Std Error	t Ratio	Prob>|t|
Intercept	-0.101977	0.154629	-0.66	0.5096
Device ID[AQY1-BA-479A]	2.4253032	0.093579	25.92	<.0001*
Device ID[AQY1-BA-480A]	0.00094	0.100279	0.01	0.9925
Device ID[AQY1-WilburSpare-07]	-5.928055	0.120719	-49.11	<.0001*
Device ID[AQY1-WilburSpare-08]	1.0960623	0.118609	9.24	<.0001*
Device ID[AQY1-WilburSpare-09]	4.6146267	0.0936	49.30	<.0001*
Device ID[AQY1-WilburSpare-10]	1.3702923	0.093579	14.64	<.0001*
Device ID[AQY-BA-353]	0.9048588	0.129667	6.98	<.0001*
Device ID[AQY-BA-431]	0.1511545	0.095644	1.58	0.1140
Device ID[AQY-BA-432]	-0.839025	0.0966	-8.69	<.0001*
Device ID[AQY-BA-464]	1.5845576	0.118822	13.34	<.0001*
Device ID[AQY-BA-480]	-2.543731	0.141487	-17.98	<.0001*
Temp	-0.045686	0.002167	-21.08	<.0001*
Device ID[AQY1-BA-479A]*(Temp-70.1646)	-0.152262	0.006019	-25.30	<.0001*
Device ID[AQY1-BA-480A]*(Temp-70.1646)	-0.047729	0.006193	-7.71	<.0001*
Device ID[AQY1-WilburSpare-07]*(Temp-70.1646)	0.2425629	0.006975	34.78	<.0001*
Device ID[AQY1-WilburSpare-08]*(Temp-70.1646)	-0.0846	0.007359	-11.50	<.0001*
Device ID[AQY1-WilburSpare-09]*(Temp-70.1646)	-0.295824	0.00602	-49.14	<.0001*
Device ID[AQY1-WilburSpare-10]*(Temp-70.1646)	-0.206046	0.006019	-34.23	<.0001*
Device ID[AQY-BA-353]*(Temp-70.1646)	-0.150157	0.007985	-18.81	<.0001*
Device ID[AQY-BA-431]*(Temp-70.1646)	0.1065808	0.006134	17.37	<.0001*
Device ID[AQY-BA-432]*(Temp-70.1646)	-0.060064	0.006084	-9.87	<.0001*
Device ID[AQY-BA-464]*(Temp-70.1646)	0.5968916	0.007813	76.40	<.0001*
Device ID[AQY-BA-480]*(Temp-70.1646)	-0.015898	0.009236	-1.72	0.0852

Effect Tests

Source	Nparm	DF	Sum of Squares	F Ratio	Prob > F
Device ID	11	11	437587.39	558.7404	<.0001*
Temp	1	1	31644.08	444.4577	<.0001*
Device ID*Temp	11	11	794385.33	1014.323	<.0001*

2) RH

Response NO2 Cal-NO2 Hinton

Regression Plot

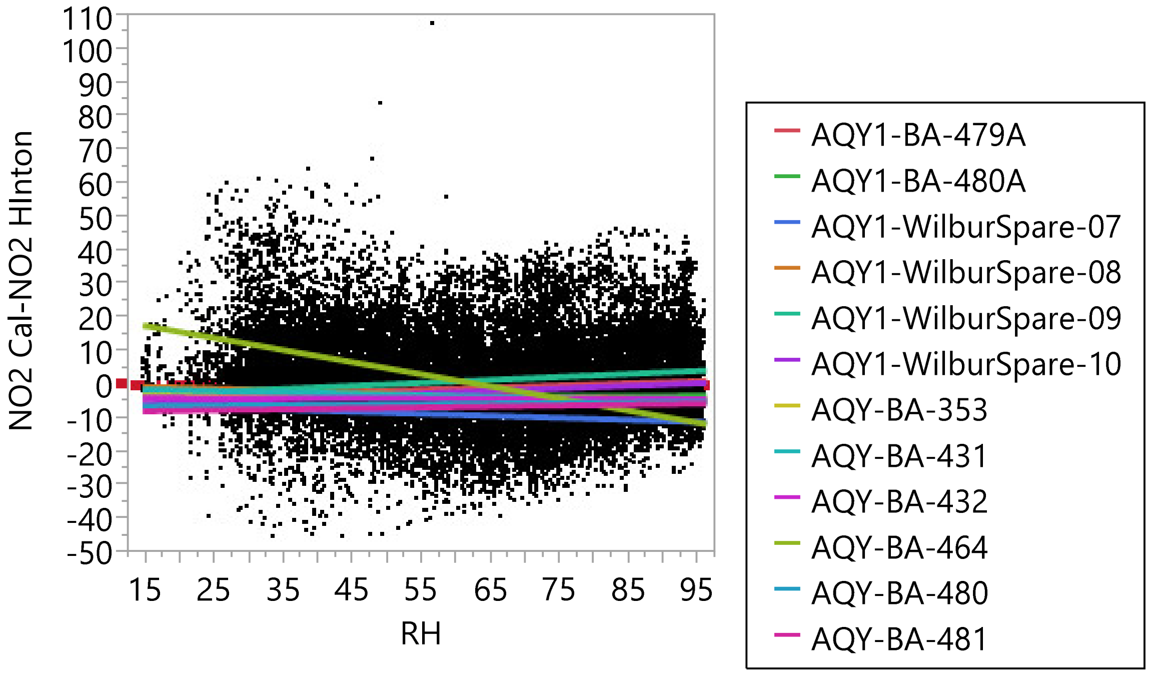

Summary of Fit

RSquare	0.121895
RSquare Adj	0.121614
Root Mean Square Error	8.91457
Mean of Response	-2.71148
Observations (or Sum Wgts)	71907

Analysis of Variance

Source	DF	Sum of Squares	Mean Square	F Ratio
Model	23	792988.5	34477.8	433.8487
Error	71883	5712510.0	79.5	**Prob > F**
C. Total	71906	6505498.5		<.0001*

Parameter Estimates

Term	Estimate	Std Error	t Ratio	Prob>|t|
Intercept	-1.782005	0.115349	-15.45	<.0001*
Device ID[AQY1-BA-479A]	2.2803313	0.09831	23.20	<.0001*
Device ID[AQY1-BA-480A]	-0.126049	0.105436	-1.20	0.2319
Device ID[AQY1-WilburSpare-07]	-5.645075	0.126061	-44.78	<.0001*
Device ID[AQY1-WilburSpare-08]	0.3318089	0.121973	2.72	0.0065*
Device ID[AQY1-WilburSpare-09]	4.4792403	0.098332	45.55	<.0001*
Device ID[AQY1-WilburSpare-10]	1.227961	0.09831	12.49	<.0001*
Device ID[AQY-BA-353]	-0.388097	0.129661	-2.99	0.0028*
Device ID[AQY-BA-431]	-0.050759	0.100408	-0.51	0.6132
Device ID[AQY-BA-432]	-0.768169	0.100723	-7.63	<.0001*
Device ID[AQY-BA-464]	4.164911	0.118155	35.25	<.0001*
Device ID[AQY-BA-480]	-2.298046	0.134093	-17.14	<.0001*
RH	-0.022468	0.001801	-12.48	<.0001*
Device ID[AQY1-BA-479A]*(RH-61.1207)	0.0861611	0.005098	16.90	<.0001*
Device ID[AQY1-BA-480A]*(RH-61.1207)	0.0266417	0.005454	4.88	<.0001*
Device ID[AQY1-WilburSpare-07]*(RH-61.1207)	-0.038378	0.00684	-5.61	<.0001*
Device ID[AQY1-WilburSpare-08]*(RH-61.1207)	-0.02983	0.006444	-4.63	<.0001*
Device ID[AQY1-WilburSpare-09]*(RH-61.1207)	0.1111704	0.005098	21.81	<.0001*
Device ID[AQY1-WilburSpare-10]*(RH-61.1207)	0.098401	0.005098	19.30	<.0001*
Device ID[AQY-BA-353]*(RH-61.1207)	-0.002428	0.00695	-0.35	0.7269
Device ID[AQY-BA-431]*(RH-61.1207)	-0.020585	0.00516	-3.99	<.0001*
Device ID[AQY-BA-432]*(RH-61.1207)	0.0182635	0.005121	3.57	0.0004*
Device ID[AQY-BA-464]*(RH-61.1207)	-0.3373	0.006431	-52.45	<.0001*
Device ID[AQY-BA-480]*(RH-61.1207)	0.0376481	0.006687	5.63	<.0001*

Effect Tests

Source	Nparm	DF	Sum of Squares	F Ratio	Prob > F
Device ID	11	11	497453.21	569.0609	<.0001*
RH	1	1	12373.39	155.6998	<.0001*
Device ID*RH	11	11	292517.68	334.6252	<.0001*

3) WD

Response NO2 Cal-NO2 Hinton

Regression Plot

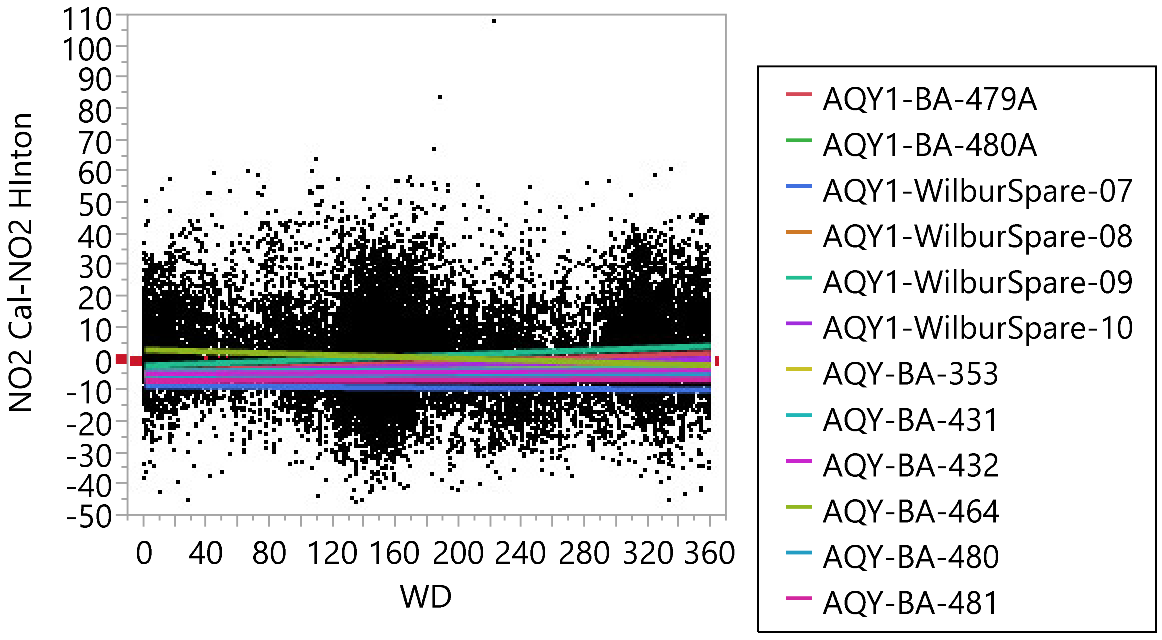

Summary of Fit

RSquare	0.086429
RSquare Adj	0.086137
Root Mean Square Error	9.092813
Mean of Response	-2.71148
Observations (or Sum Wgts)	71907

Analysis of Variance

Source	DF	Sum of Squares	Mean Square	F Ratio
Model	23	562266.2	24446.4	295.6771
Error	71883	5943232.3	82.7	**Prob > F**
C. Total	71906	6505498.5		<.0001*

Parameter Estimates

Term	Estimate	Std Error	t Ratio	Prob>|t|
Intercept	-4.027438	0.07634	-52.76	<.0001*
Device ID[AQY1-BA-479A]	2.2473925	0.100273	22.41	<.0001*
Device ID[AQY1-BA-480A]	-0.166019	0.107523	-1.54	0.1226
Device ID[AQY1-WilburSpare-07]	-5.601261	0.128337	-43.64	<.0001*
Device ID[AQY1-WilburSpare-08]	0.4384154	0.124006	3.54	0.0004*
Device ID[AQY1-WilburSpare-09]	4.4483647	0.100296	44.35	<.0001*
Device ID[AQY1-WilburSpare-10]	1.1991185	0.100273	11.96	<.0001*
Device ID[AQY-BA-353]	-0.301704	0.13244	-2.28	0.0227*
Device ID[AQY-BA-431]	-0.076523	0.102414	-0.75	0.4550
Device ID[AQY-BA-432]	-0.799465	0.102736	-7.78	<.0001*
Device ID[AQY-BA-464]	4.1184182	0.121355	33.94	<.0001*
Device ID[AQY-BA-480]	-2.325079	0.136493	-17.03	<.0001*
WD	0.0052662	0.000396	13.29	<.0001*
Device ID[AQY1-BA-479A]*(WD-171.267)	0.0112178	0.001122	10.00	<.0001*
Device ID[AQY1-BA-480A]*(WD-171.267)	0.0050272	0.001212	4.15	<.0001*
Device ID[AQY1-WilburSpare-07]*(WD-171.267)	-0.009033	0.001485	-6.08	<.0001*
Device ID[AQY1-WilburSpare-08]*(WD-171.267)	0.0023979	0.001439	1.67	0.0957
Device ID[AQY1-WilburSpare-09]*(WD-171.267)	0.012159	0.001122	10.84	<.0001*
Device ID[AQY1-WilburSpare-10]*(WD-171.267)	0.0076191	0.001122	6.79	<.0001*
Device ID[AQY-BA-353]*(WD-171.267)	0.0010709	0.001606	0.67	0.5048
Device ID[AQY-BA-431]*(WD-171.267)	-0.002915	0.001126	-2.59	0.0096*
Device ID[AQY-BA-432]*(WD-171.267)	-0.00268	0.001121	-2.39	0.0168*
Device ID[AQY-BA-464]*(WD-171.267)	-0.019167	0.001445	-13.26	<.0001*
Device ID[AQY-BA-480]*(WD-171.267)	-0.001835	0.001419	-1.29	0.1962

Effect Tests

Source	Nparm	DF	Sum of Squares	F Ratio	Prob > F
Device ID	11	11	490214.26	539.0099	<.0001*
WD	1	1	14594.71	176.5220	<.0001*
Device ID*WD	11	11	38797.48	42.6594	<.0001*

4) WS

Response NO2 Cal-NO2 Hinton

Regression Plot

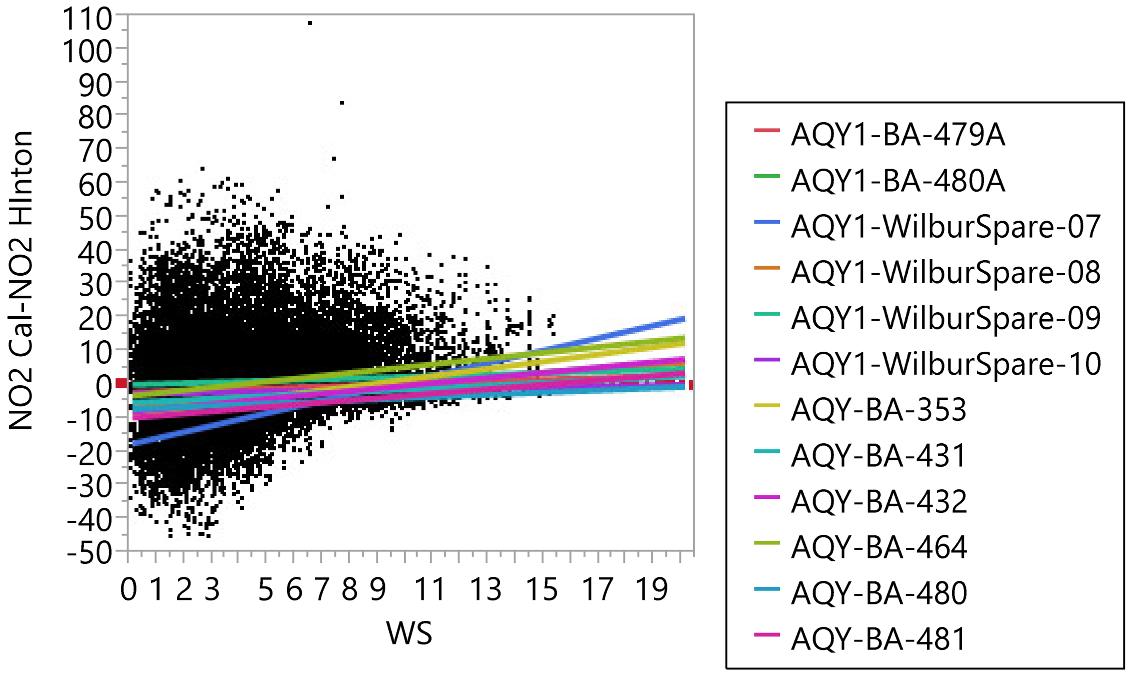

Summary of Fit

RSquare	0.113304
RSquare Adj	0.11302
Root Mean Square Error	8.958072
Mean of Response	-2.71148
Observations (or Sum Wgts)	71907

Analysis of Variance

Source	DF	Sum of Squares	Mean Square	F Ratio
Model	23	737098.9	32047.8	399.3639
Error	71883	5768399.6	80.2	**Prob > F**
C. Total	71906	6505498.5		<.0001*

Parameter Estimates

Term	Estimate	Std Error	t Ratio	Prob>|t|
Intercept	-6.436839	0.0773	-83.27	<.0001*
Device ID[AQY1-BA-479A]	2.1962434	0.098771	22.24	<.0001*
Device ID[AQY1-BA-480A]	-0.149	0.105958	-1.41	0.1597
Device ID[AQY1-WilburSpare-07]	-5.008784	0.127679	-39.23	<.0001*
Device ID[AQY1-WilburSpare-08]	0.4003569	0.122045	3.28	0.0010*
Device ID[AQY1-WilburSpare-09]	4.3989713	0.098793	44.53	<.0001*
Device ID[AQY1-WilburSpare-10]	1.1487937	0.098771	11.63	<.0001*
Device ID[AQY-BA-353]	-0.451922	0.129532	-3.49	0.0005*
Device ID[AQY-BA-431]	-0.144081	0.100883	-1.43	0.1532
Device ID[AQY-BA-432]	-0.926428	0.101249	-9.15	<.0001*
Device ID[AQY-BA-464]	4.2344965	0.118703	35.67	<.0001*
Device ID[AQY-BA-480]	-2.388485	0.133868	-17.84	<.0001*
WS	0.6581927	0.013546	48.59	<.0001*
Device ID[AQY1-BA-479A]*(WS-5.1157)	-0.404362	0.038336	-10.55	<.0001*
Device ID[AQY1-BA-480A]*(WS-5.1157)	0.0417177	0.04147	1.01	0.3144
Device ID[AQY1-WilburSpare-07]*(WS-5.1157)	1.2035813	0.052994	22.71	<.0001*
Device ID[AQY1-WilburSpare-08]*(WS-5.1157)	-0.03277	0.048295	-0.68	0.4974
Device ID[AQY1-WilburSpare-09]*(WS-5.1157)	-0.425094	0.038338	-11.09	<.0001*
Device ID[AQY1-WilburSpare-10]*(WS-5.1157)	-0.527882	0.038336	-13.77	<.0001*
Device ID[AQY-BA-353]*(WS-5.1157)	0.4096705	0.050846	8.06	<.0001*
Device ID[AQY-BA-431]*(WS-5.1157)	-0.26688	0.038917	-6.86	<.0001*
Device ID[AQY-BA-432]*(WS-5.1157)	0.1239008	0.038427	3.22	0.0013*
Device ID[AQY-BA-464]*(WS-5.1157)	0.2005366	0.048138	4.17	<.0001*
Device ID[AQY-BA-480]*(WS-5.1157)	-0.327185	0.050283	-6.51	<.0001*

Effect Tests

Source	Nparm	DF	Sum of Squares	F Ratio	Prob > F
Device ID	11	11	466965.33	529.0087	<.0001*
WS	1	1	189466.22	2361.036	<.0001*
Device ID*WS	11	11	79774.17	90.3734	<.0001*

Table S6. Results of ANCOVA F-tests for $PM_{2.5}$ Raw – $PM_{2.5}$ Hinton

Source	Nparm	DF	Sum of Squares	F Ratio	Prob > F
Device ID	11	11	135305.63	373.1184	<.0001*
Temp	1	1	60963.53	1849.241	<.0001*
RH	1	1	22914.14	695.0675	<.0001*
WD	1	1	429.28	13.0215	0.0003*
WS	1	1	5509.04	167.1089	<.0001*
Device ID*Temp	11	11	21014.54	57.9496	<.0001*
Device ID*RH	11	11	18883.53	52.0732	<.0001*
Device ID*WD	11	11	844.02	2.3275	0.0074*
Device ID*WS	11	11	4390.88	12.1083	<.0001*

Supplementary Material: Underlying ANCOVA results for Figure 4 (PM2.5)

1. **Raw data**
1) **Temp**

Response PM2.5 Raw-PM2.5 Hinton
Regression Plot

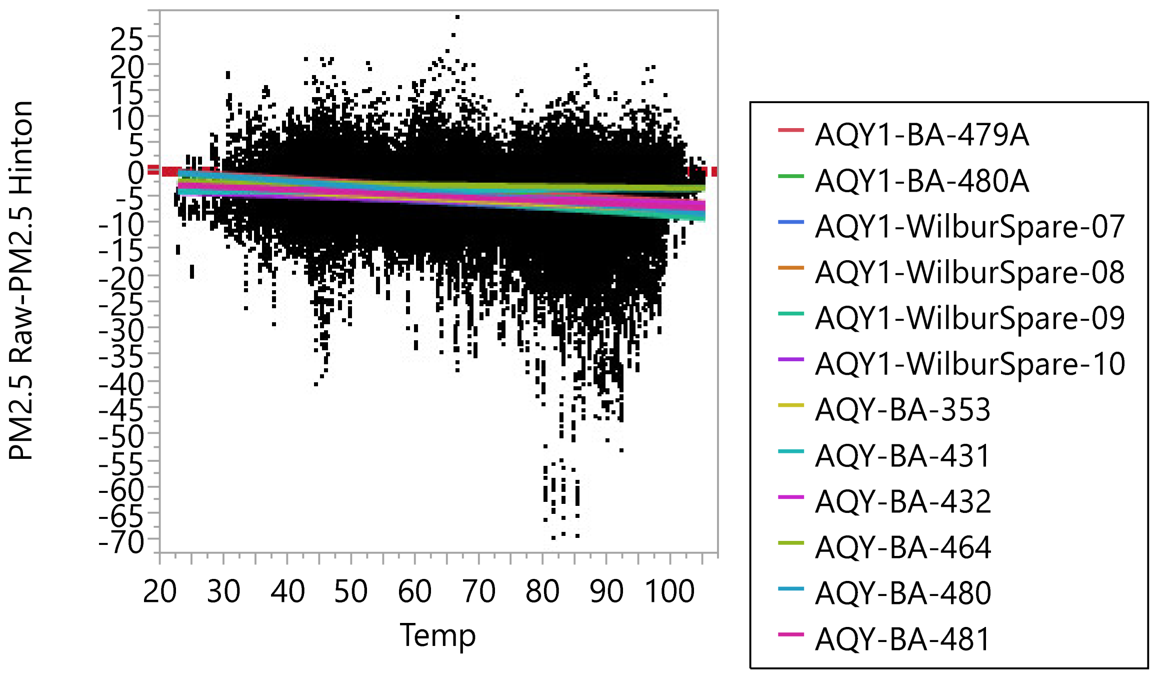

Summary of Fit

RSquare	0.058506
RSquare Adj	0.058347
Root Mean Square Error	5.773907
Mean of Response	-4.8299
Observations (or Sum Wgts)	136268

Analysis of Variance

Source	DF	Sum of Squares	Mean Square	F Ratio
Model	23	282251.7	12271.8	368.1028
Error	136244	4542102.8	33.3	**Prob > F**
C. Total	136267	4824354.4		<.0001*

Parameter Estimates

Term	Estimate	Std Error	t Ratio	Prob>|t|
Intercept	-0.957323	0.069315	-13.81	<.0001*
Device ID[AQY1-BA-479A]	1.2317449	0.050142	24.57	<.0001*
Device ID[AQY1-BA-480A]	0.6386913	0.049525	12.90	<.0001*
Device ID[AQY1-WilburSpare-07]	-1.442751	0.056186	-25.68	<.0001*
Device ID[AQY1-WilburSpare-08]	-0.414544	0.0496	-8.36	<.0001*
Device ID[AQY1-WilburSpare-09]	-1.206914	0.049528	-24.37	<.0001*
Device ID[AQY1-WilburSpare-10]	-1.094342	0.050367	-21.73	<.0001*
Device ID[AQY-BA-353]	-0.860399	0.04956	-17.36	<.0001*
Device ID[AQY-BA-431]	1.271128	0.050547	25.15	<.0001*
Device ID[AQY-BA-432]	0.0720139	0.049564	1.45	0.1462

Term	Estimate	Std Error	t Ratio	Prob>|t|
Device ID[AQY-BA-464]	2.0607784	0.065597	31.42	<.0001*
Device ID[AQY-BA-480]	0.0663639	0.055092	1.20	0.2284
Temp	-0.05431	0.000955	-56.88	<.0001*
Device ID[AQY1-BA-479A]*(Temp-70.3489)	-0.016954	0.003021	-5.61	<.0001*
Device ID[AQY1-BA-480A]*(Temp-70.3489)	-0.020795	0.002975	-6.99	<.0001*
Device ID[AQY1-WilburSpare-07]*(Temp-70.3489)	-0.006686	0.003299	-2.03	0.0427*
Device ID[AQY1-WilburSpare-08]*(Temp-70.3489)	-0.013602	0.002981	-4.56	<.0001*
Device ID[AQY1-WilburSpare-09]*(Temp-70.3489)	-0.030123	0.002975	-10.13	<.0001*
Device ID[AQY1-WilburSpare-10]*(Temp-70.3489)	0.0144563	0.003049	4.74	<.0001*
Device ID[AQY-BA-353]*(Temp-70.3489)	-0.001046	0.002977	-0.35	0.7253
Device ID[AQY-BA-431]*(Temp-70.3489)	0.0620072	0.003039	20.40	<.0001*
Device ID[AQY-BA-432]*(Temp-70.3489)	0.0104367	0.002979	3.50	0.0005*
Device ID[AQY-BA-464]*(Temp-70.3489)	0.039049	0.004036	9.67	<.0001*
Device ID[AQY-BA-480]*(Temp-70.3489)	-0.038247	0.003251	-11.76	<.0001*

Effect Tests

Source	Nparm	DF	Sum of Squares	F Ratio	Prob > F
Device ID	11	11	137549.44	375.0823	<.0001*
Temp	1	1	107848.16	3234.992	<.0001*
Device ID*Temp	11	11	27240.51	74.2819	<.0001*

2) RH

Response PM2.5 Raw-PM2.5 Hinton
Regression Plot

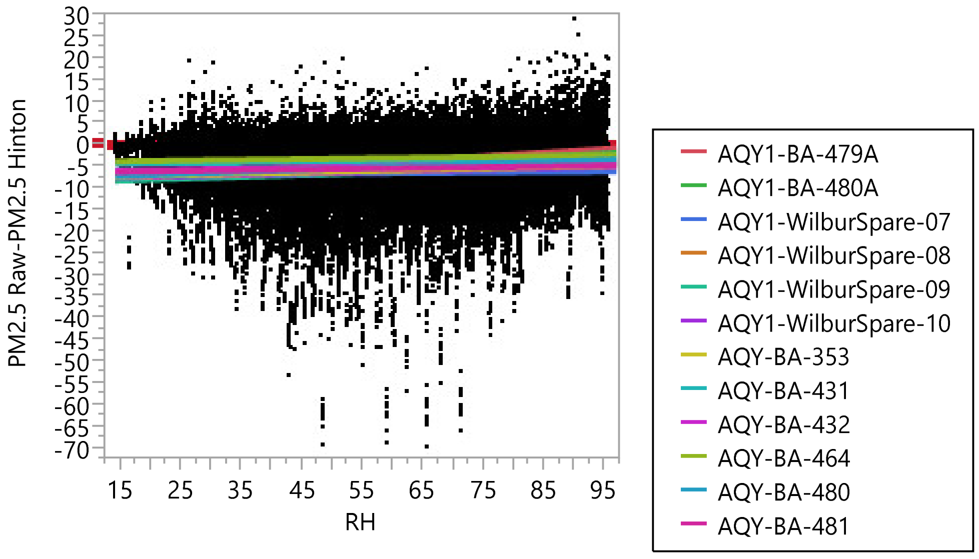

Summary of Fit

RSquare	0.049203
RSquare Adj	0.049042
Root Mean Square Error	5.803137
Mean of Response	-4.83089
Observations (or Sum Wgts)	136288

Analysis of Variance

Source	DF	Sum of Squares	Mean Square	F Ratio
Model	23	237469.0	10324.7	306.5868
Error	136264	4588881.1	33.7	**Prob > F**
C. Total	136287	4826350.2		<.0001*

Parameter Estimates

Term	Estimate	Std Error	t Ratio	Prob>|t|
Intercept	-7.022425	0.053151	-132.1	<.0001*
Device ID[AQY1-BA-479A]	1.2354715	0.050382	24.52	<.0001*
Device ID[AQY1-BA-480A]	0.6798359	0.049738	13.67	<.0001*
Device ID[AQY1-WilburSpare-07]	-1.499503	0.056364	-26.60	<.0001*
Device ID[AQY1-WilburSpare-08]	-0.375974	0.049814	-7.55	<.0001*
Device ID[AQY1-WilburSpare-09]	-1.15841	0.049741	-23.29	<.0001*
Device ID[AQY1-WilburSpare-10]	-1.085334	0.050611	-21.44	<.0001*
Device ID[AQY-BA-353]	-0.828125	0.049777	-16.64	<.0001*
Device ID[AQY-BA-431]	1.2738202	0.050751	25.10	<.0001*
Device ID[AQY-BA-432]	0.0999725	0.04978	2.01	0.0446*
Device ID[AQY-BA-464]	2.0226975	0.065392	30.93	<.0001*
Device ID[AQY-BA-480]	-0.005573	0.055289	-0.10	0.9197
RH	0.0366315	0.000829	44.16	<.0001*
Device ID[AQY1-BA-479A]*(RH-61.0918)	0.0468713	0.002604	18.00	<.0001*
Device ID[AQY1-BA-480A]*(RH-61.0918)	0.0328685	0.002569	12.79	<.0001*
Device ID[AQY1-WilburSpare-07]*(RH-61.0918)	-0.026041	0.002939	-8.86	<.0001*
Device ID[AQY1-WilburSpare-08]*(RH-61.0918)	0.0132644	0.002573	5.15	<.0001*
Device ID[AQY1-WilburSpare-09]*(RH-61.0918)	0.0103345	0.002569	4.02	<.0001*
Device ID[AQY1-WilburSpare-10]*(RH-61.0918)	-0.005332	0.002637	-2.02	0.0432*
Device ID[AQY-BA-353]*(RH-61.0918)	-0.005925	0.002571	-2.30	0.0212*
Device ID[AQY-BA-431]*(RH-61.0918)	-0.022375	0.00261	-8.57	<.0001*
Device ID[AQY-BA-432]*(RH-61.0918)	-0.014996	0.002574	-5.82	<.0001*

Term	Estimate	Std Error	t Ratio	Prob>|t|
Device ID[AQY-BA-464]*(RH-61.0918)	-0.019513	0.003445	-5.66	<.0001*
Device ID[AQY-BA-480]*(RH-61.0918)	0.00963	0.002888	3.33	0.0009*

Effect Tests

Source	Nparm	DF	Sum of Squares	F Ratio	Prob > F
Device ID	11	11	137209.98	370.3969	<.0001*
RH	1	1	65684.58	1950.463	<.0001*
Device ID*RH	11	11	25078.01	67.6978	<.0001*

3) WD

Response PM2.5 Raw-PM2.5 Hinton
Regression Plot

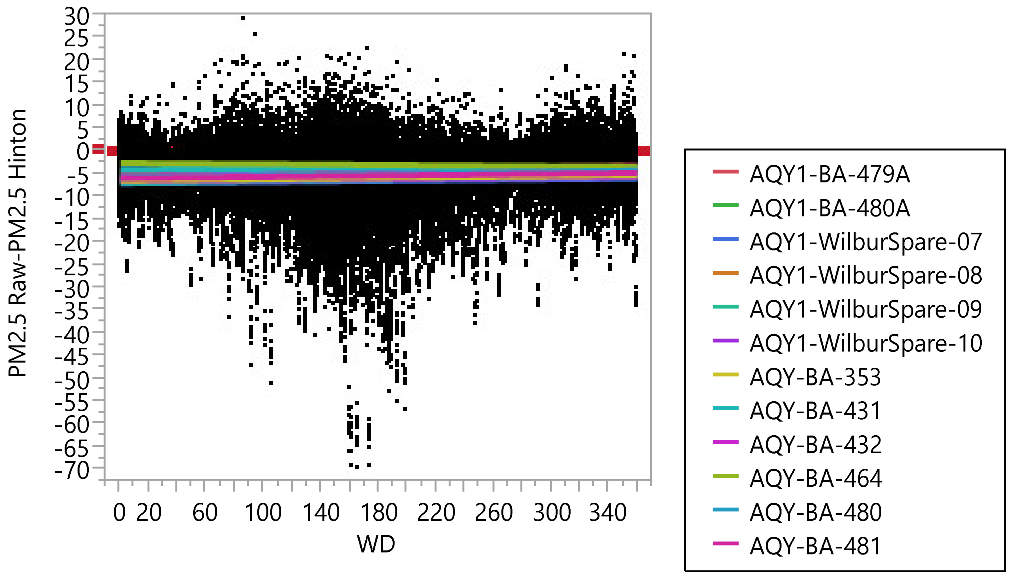

Summary of Fit

RSquare	0.030688
RSquare Adj	0.030524
Root Mean Square Error	5.861536
Mean of Response	-4.84692
Observations (or Sum Wgts)	135744

Analysis of Variance

Source	DF	Sum of Squares	Mean Square	F Ratio
Model	23	147628.1	6418.61	186.8178
Error	135720	4663014.8	34.36	**Prob > F**
C. Total	135743	4810642.9		<.0001*

Parameter Estimates

Term	Estimate	Std Error	t Ratio	Prob>|t|
Intercept	-5.23684	0.035119	-149.1	<.0001*
Device ID[AQY1-BA-479A]	1.2418156	0.051024	24.34	<.0001*
Device ID[AQY1-BA-480A]	0.6801774	0.050369	13.50	<.0001*
Device ID[AQY1-WilburSpare-07]	-1.48162	0.056951	-26.02	<.0001*
Device ID[AQY1-WilburSpare-08]	-0.379627	0.050446	-7.53	<.0001*
Device ID[AQY1-WilburSpare-09]	-1.167924	0.050373	-23.19	<.0001*
Device ID[AQY1-WilburSpare-10]	-1.105198	0.051255	-21.56	<.0001*
Device ID[AQY-BA-353]	-0.83707	0.050408	-16.61	<.0001*
Device ID[AQY-BA-431]	1.2610526	0.051399	24.53	<.0001*
Device ID[AQY-BA-432]	0.0882125	0.050412	1.75	0.0801
Device ID[AQY-BA-464]	2.0370809	0.066134	30.80	<.0001*
Device ID[AQY-BA-480]	0.0062585	0.055859	0.11	0.9108
WD	0.0025549	0.000183	14.00	<.0001*
Device ID[AQY1-BA-479A]*(WD-171.466)	0.0003232	0.000574	0.56	0.5732
Device ID[AQY1-BA-480A]*(WD-171.466)	0.000245	0.000564	0.43	0.6640
Device ID[AQY1-WilburSpare-07]*(WD-171.466)	0.0005384	0.000652	0.83	0.4089
Device ID[AQY1-WilburSpare-08]*(WD-171.466)	0.0003191	0.000565	0.56	0.5723
Device ID[AQY1-WilburSpare-09]*(WD-171.466)	0.0014827	0.000564	2.63	0.0086*
Device ID[AQY1-WilburSpare-10]*(WD-171.466)	-4.182e-5	0.000575	-0.07	0.9420
Device ID[AQY-BA-353]*(WD-171.466)	0.0006273	0.000565	1.11	0.2671
Device ID[AQY-BA-431]*(WD-171.466)	-0.001292	0.000569	-2.27	0.0233*
Device ID[AQY-BA-432]*(WD-171.466)	0.0003857	0.000565	0.68	0.4947
Device ID[AQY-BA-464]*(WD-171.466)	-0.004896	0.000762	-6.42	<.0001*
Device ID[AQY-BA-480]*(WD-171.466)	0.0016078	0.000637	2.52	0.0117*

Effect Tests

Source	Nparm	DF	Sum of Squares	F Ratio	Prob > F
Device ID	11	11	137233.47	363.1152	<.0001*
WD	1	1	6733.23	195.9749	<.0001*
Device ID*WD	11	11	1949.66	5.1587	<.0001*

4) WS

Response PM2.5 Raw-PM2.5 Hinton
Regression Plot

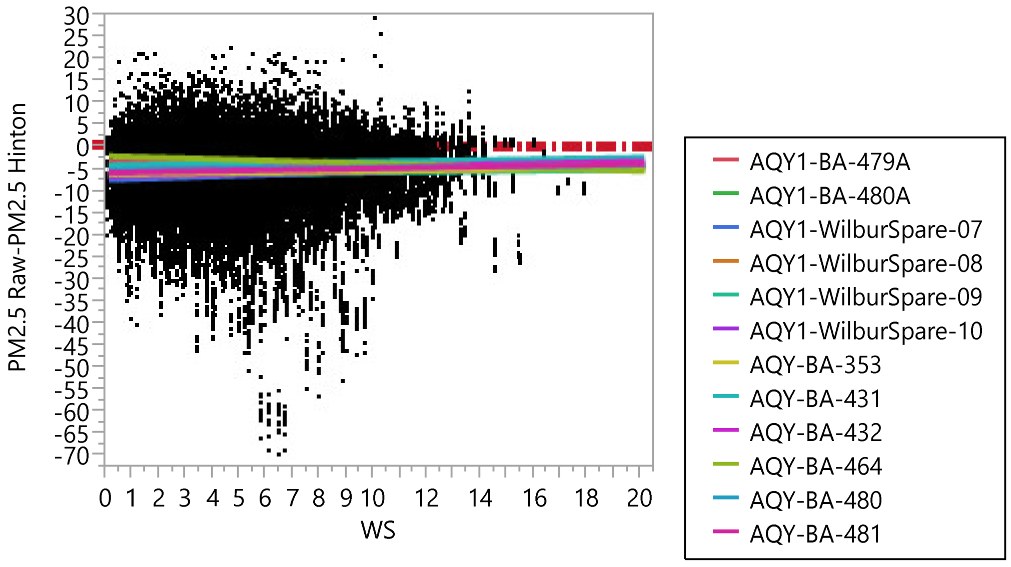

Summary of Fit

RSquare	0.030379
RSquare Adj	0.030215
Root Mean Square Error	5.86247
Mean of Response	-4.84692
Observations (or Sum Wgts)	135744

Analysis of Variance

Source	DF	Sum of Squares	Mean Square	F Ratio
Model	23	146141.9	6354.00	184.8782
Error	135720	4664501.0	34.37	**Prob > F**
C. Total	135743	4810642.9		<.0001*

Parameter Estimates

Term	Estimate	Std Error	t Ratio	Prob>|t|
Intercept	-5.056527	0.036718	-137.7	<.0001*
Device ID[AQY1-BA-479A]	1.2420058	0.051032	24.34	<.0001*
Device ID[AQY1-BA-480A]	0.6801307	0.05038	13.50	<.0001*
Device ID[AQY1-WilburSpare-07]	-1.467454	0.057006	-25.74	<.0001*
Device ID[AQY1-WilburSpare-08]	-0.381346	0.050458	-7.56	<.0001*
Device ID[AQY1-WilburSpare-09]	-1.169463	0.050384	-23.21	<.0001*
Device ID[AQY1-WilburSpare-10]	-1.109346	0.051265	-21.64	<.0001*
Device ID[AQY-BA-353]	-0.840322	0.05042	-16.67	<.0001*
Device ID[AQY-BA-431]	1.2549678	0.051416	24.41	<.0001*
Device ID[AQY-BA-432]	0.0837793	0.050424	1.66	0.0966
Device ID[AQY-BA-464]	2.037309	0.066063	30.84	<.0001*
Device ID[AQY-BA-480]	0.0075875	0.055907	0.14	0.8920
WS	0.0501045	0.006373	7.86	<.0001*
Device ID[AQY1-BA-479A]*(WS-5.19107)	-0.121169	0.020019	-6.05	<.0001*
Device ID[AQY1-BA-480A]*(WS-5.19107)	-0.050422	0.019598	-2.57	0.0101*
Device ID[AQY1-WilburSpare-07]*(WS-5.19107)	0.1427687	0.022983	6.21	<.0001*
Device ID[AQY1-WilburSpare-08]*(WS-5.19107)	-0.023453	0.019617	-1.20	0.2319
Device ID[AQY1-WilburSpare-09]*(WS-5.19107)	0.0186183	0.019598	0.95	0.3421
Device ID[AQY1-WilburSpare-10]*(WS-5.19107)	0.0697081	0.02001	3.48	0.0005*
Device ID[AQY-BA-353]*(WS-5.19107)	0.0217496	0.019612	1.11	0.2674
Device ID[AQY-BA-431]*(WS-5.19107)	0.0371786	0.019926	1.87	0.0621
Device ID[AQY-BA-432]*(WS-5.19107)	0.0598745	0.019619	3.05	0.0023*
Device ID[AQY-BA-464]*(WS-5.19107)	-0.219521	0.02656	-8.27	<.0001*
Device ID[AQY-BA-480]*(WS-5.19107)	0.0153878	0.022441	0.69	0.4929

Effect Tests

Source	Nparm	DF	Sum of Squares	F Ratio	Prob > F
Device ID	11	11	136883.98	362.0750	<.0001*
WS	1	1	2124.06	61.8025	<.0001*
Device ID*WS	11	11	5709.46	15.1022	<.0001*

2. Calibrated data
1) Temp

Response PM2.5 Cal-PM2.5 Hinton
Regression Plot

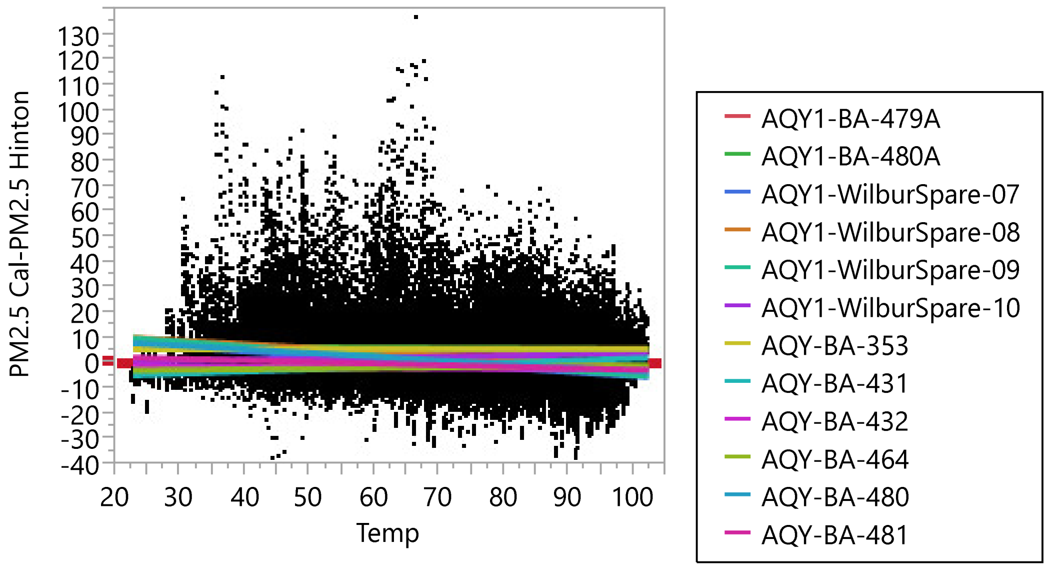

Summary of Fit

RSquare	0.089846
RSquare Adj	0.089635
Root Mean Square Error	10.11525
Mean of Response	2.262342
Observations (or Sum Wgts)	99231

Analysis of Variance

Source	DF	Sum of Squares	Mean Square	F Ratio
Model	23	1002033	43566.6	425.7950
Error	99207	10150695	102.3	**Prob > F**
C. Total	99230	11152728		<.0001*

Parameter Estimates

Term	Estimate	Std Error	t Ratio	Prob>|t|
Intercept	5.517925	0.146808	37.59	<.0001*
Device ID[AQY1-BA-479A]	3.3627945	0.098213	34.24	<.0001*
Device ID[AQY1-BA-480A]	4.2916467	0.096706	44.38	<.0001*
Device ID[AQY1-WilburSpare-07]	-1.513229	0.145685	-10.39	<.0001*
Device ID[AQY1-WilburSpare-08]	1.7077213	0.116886	14.61	<.0001*
Device ID[AQY1-WilburSpare-09]	-0.324326	0.096722	-3.35	0.0008*
Device ID[AQY1-WilburSpare-10]	0.7294623	0.098788	7.38	<.0001*
Device ID[AQY-BA-353]	3.914774	0.113352	34.54	<.0001*
Device ID[AQY-BA-431]	-2.585426	0.097628	-26.48	<.0001*
Device ID[AQY-BA-432]	-3.65121	0.095439	-38.26	<.0001*
Device ID[AQY-BA-464]	-3.433753	0.123454	-27.81	<.0001*
Device ID[AQY-BA-480]	-0.216244	0.142616	-1.52	0.1295
Temp	-0.050523	0.002134	-23.68	<.0001*
Device ID[AQY1-BA-479A]*(Temp-67.6201)	0.0262099	0.006104	4.29	<.0001*
Device ID[AQY1-BA-480A]*(Temp-67.6201)	0.0362107	0.006007	6.03	<.0001*
Device ID[AQY1-WilburSpare-07]*(Temp-67.6201)	-0.110866	0.008108	-13.67	<.0001*
Device ID[AQY1-WilburSpare-08]*(Temp-67.6201)	-0.097313	0.006993	-13.92	<.0001*
Device ID[AQY1-WilburSpare-09]*(Temp-67.6201)	-0.137028	0.006008	-22.81	<.0001*
Device ID[AQY1-WilburSpare-10]*(Temp-67.6201)	0.0905335	0.006163	14.69	<.0001*
Device ID[AQY-BA-353]*(Temp-67.6201)	0.0521089	0.006723	7.75	<.0001*
Device ID[AQY-BA-431]*(Temp-67.6201)	0.1365416	0.006045	22.59	<.0001*
Device ID[AQY-BA-432]*(Temp-67.6201)	0.0348153	0.005885	5.92	<.0001*
Device ID[AQY-BA-464]*(Temp-67.6201)	0.0733319	0.007379	9.94	<.0001*
Device ID[AQY-BA-480]*(Temp-67.6201)	-0.09373	0.009164	-10.23	<.0001*

Effect Tests

Source	Nparm	DF	Sum of Squares	F Ratio	Prob > F
Device ID	11	11	759782.44	675.0611	<.0001*
Temp	1	1	57376.88	560.7683	<.0001*
Device ID*Temp	11	11	187026.03	166.1713	<.0001*

2) RH

Response PM2.5 Cal-PM2.5 Hinton
Regression Plot

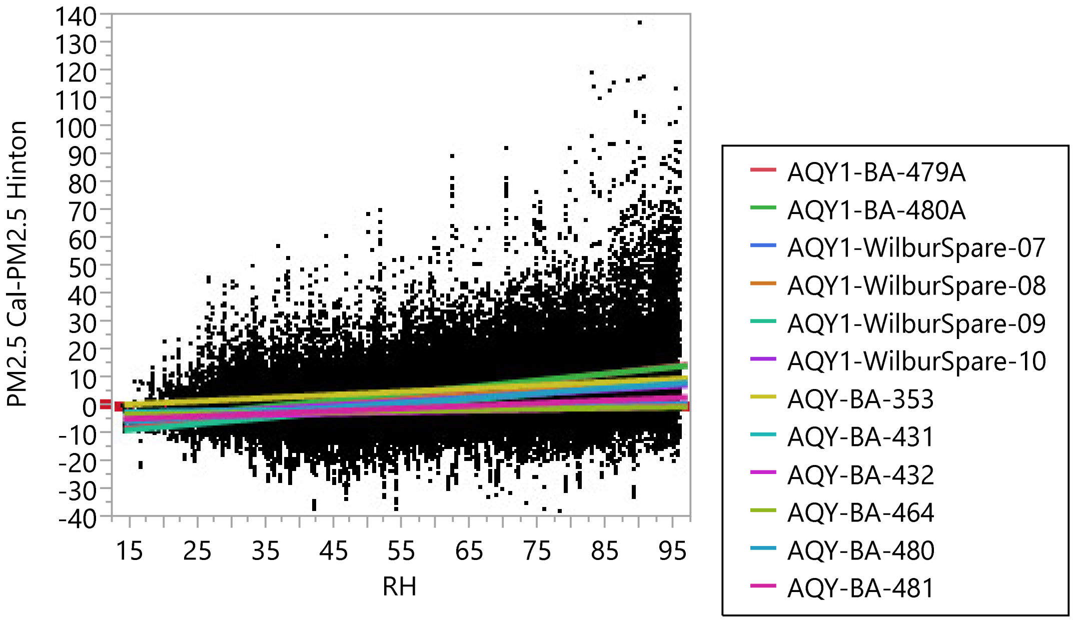

Summary of Fit

RSquare	0.158563
RSquare Adj	0.158368
Root Mean Square Error	9.725548
Mean of Response	2.262132
Observations (or Sum Wgts)	99239

Analysis of Variance

Source	DF	Sum of Squares	Mean Square	F Ratio
Model	23	1768418	76887.8	812.8848
Error	99215	9384378	94.6	**Prob > F**
C. Total	99238	11152797		<.0001*

Parameter Estimates

Term	Estimate	Std Error	t Ratio	Prob>|t|
Intercept	-5.969475	0.106695	-55.95	<.0001*
Device ID[AQY1-BA-479A]	3.2767921	0.093952	34.88	<.0001*
Device ID[AQY1-BA-480A]	4.2437763	0.092526	45.87	<.0001*
Device ID[AQY1-WilburSpare-07]	-2.304587	0.134479	-17.14	<.0001*
Device ID[AQY1-WilburSpare-08]	1.5328581	0.110696	13.85	<.0001*
Device ID[AQY1-WilburSpare-09]	-0.369193	0.092541	-3.99	<.0001*
Device ID[AQY1-WilburSpare-10]	0.7803027	0.094452	8.26	<.0001*
Device ID[AQY-BA-353]	4.0611732	0.10815	37.55	<.0001*
Device ID[AQY-BA-431]	-2.664094	0.093289	-28.56	<.0001*
Device ID[AQY-BA-432]	-3.654947	0.091258	-40.05	<.0001*
Device ID[AQY-BA-464]	-3.341446	0.115294	-28.98	<.0001*
Device ID[AQY-BA-480]	0.4741505	0.122336	3.88	0.0001*
RH	0.1310316	0.001654	79.22	<.0001*
Device ID[AQY1-BA-479A]*(RH-61.6162)	0.1436141	0.004808	29.87	<.0001*
Device ID[AQY1-BA-480A]*(RH-61.6162)	0.1040966	0.004733	21.99	<.0001*
Device ID[AQY1-WilburSpare-07]*(RH-61.6162)	-0.089026	0.007224	-12.32	<.0001*
Device ID[AQY1-WilburSpare-08]*(RH-61.6162)	0.0492776	0.005818	8.47	<.0001*
Device ID[AQY1-WilburSpare-09]*(RH-61.6162)	0.0829033	0.004733	17.52	<.0001*
Device ID[AQY1-WilburSpare-10]*(RH-61.6162)	0.0065623	0.004876	1.35	0.1783
Device ID[AQY-BA-353]*(RH-61.6162)	-0.017955	0.005633	-3.19	0.0014*
Device ID[AQY-BA-431]*(RH-61.6162)	-0.102521	0.004722	-21.71	<.0001*
Device ID[AQY-BA-432]*(RH-61.6162)	-0.080582	0.004652	-17.32	<.0001*
Device ID[AQY-BA-464]*(RH-61.6162)	-0.097623	0.006009	-16.25	<.0001*
Device ID[AQY-BA-480]*(RH-61.6162)	0.0388735	0.005985	6.50	<.0001*

Effect Tests

Source	Nparm	DF	Sum of Squares	F Ratio	Prob > F
Device ID	11	11	779840.88	749.5233	<.0001*
RH	1	1	593584.05	6275.583	<.0001*
Device ID*RH	11	11	267303.40	256.9116	<.0001*

3) WD

Response PM2.5 Cal-PM2.5 Hinton
Regression Plot

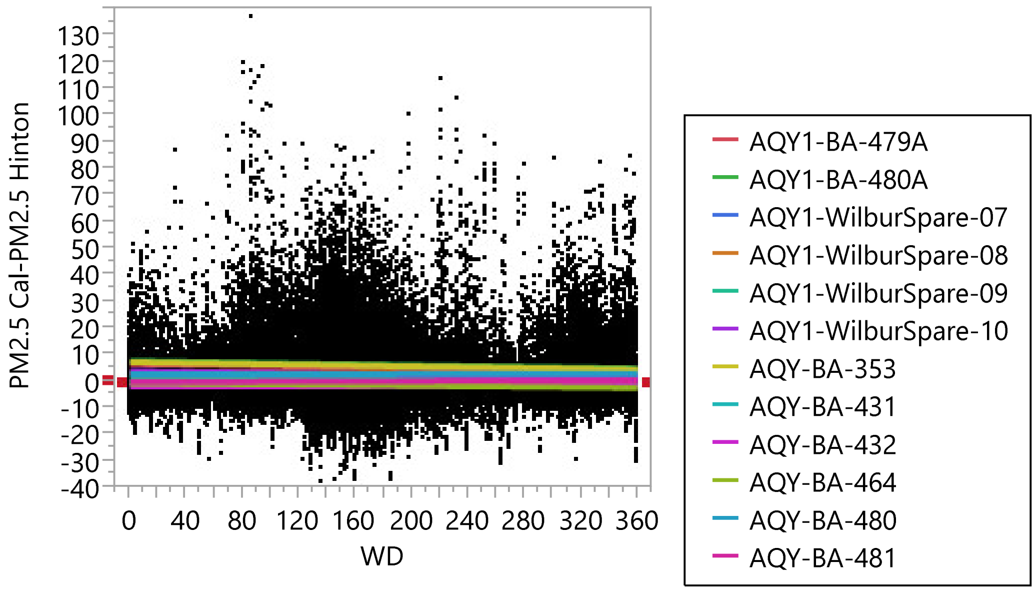

Summary of Fit

RSquare	0.071555
RSquare Adj	0.071339
Root Mean Square Error	10.23069
Mean of Response	2.256144
Observations (or Sum Wgts)	98710

Analysis of Variance

Source	DF	Sum of Squares	Mean Square	F Ratio
Model	23	796071	34611.8	330.6848
Error	98686	10329167	104.7	**Prob > F**
C. Total	98709	11125238		<.0001*

Parameter Estimates

Term	Estimate	Std Error	t Ratio	Prob>|t|
Intercept	2.5026778	0.073315	34.14	<.0001*
Device ID[AQY1-BA-479A]	3.349055	0.099122	33.79	<.0001*
Device ID[AQY1-BA-480A]	4.2894054	0.097611	43.94	<.0001*
Device ID[AQY1-WilburSpare-07]	-2.374106	0.140805	-16.86	<.0001*
Device ID[AQY1-WilburSpare-08]	1.265968	0.116635	10.85	<.0001*
Device ID[AQY1-WilburSpare-09]	-0.343453	0.097627	-3.52	0.0004*
Device ID[AQY1-WilburSpare-10]	0.7367343	0.099658	7.39	<.0001*
Device ID[AQY-BA-353]	3.894116	0.114012	34.16	<.0001*
Device ID[AQY-BA-431]	-2.694952	0.098426	-27.38	<.0001*
Device ID[AQY-BA-432]	-3.675201	0.096265	-38.18	<.0001*
Device ID[AQY-BA-464]	-3.400841	0.121858	-27.91	<.0001*
Device ID[AQY-BA-480]	0.8153975	0.12857	6.34	<.0001*
WD	-0.002203	0.000369	-5.96	<.0001*
Device ID[AQY1-BA-479A]*(WD-176.099)	-0.005366	0.00108	-4.97	<.0001*
Device ID[AQY1-BA-480A]*(WD-176.099)	-0.005605	0.001059	-5.29	<.0001*
Device ID[AQY1-WilburSpare-07]*(WD-176.099)	0.0085281	0.001631	5.23	<.0001*
Device ID[AQY1-WilburSpare-08]*(WD-176.099)	0.0017794	0.001312	1.36	0.1749
Device ID[AQY1-WilburSpare-09]*(WD-176.099)	0.0040723	0.001059	3.84	0.0001*
Device ID[AQY1-WilburSpare-10]*(WD-176.099)	-0.004386	0.001081	-4.06	<.0001*
Device ID[AQY-BA-353]*(WD-176.099)	-0.00559	0.001269	-4.40	<.0001*
Device ID[AQY-BA-431]*(WD-176.099)	-0.000123	0.001048	-0.12	0.9069
Device ID[AQY-BA-432]*(WD-176.099)	0.0027477	0.00104	2.64	0.0082*
Device ID[AQY-BA-464]*(WD-176.099)	-0.00266	0.001364	-1.95	0.0511
Device ID[AQY-BA-480]*(WD-176.099)	0.0029065	0.001305	2.23	0.0259*

Effect Tests

Source	Nparm	DF	Sum of Squares	F Ratio	Prob > F
Device ID	11	11	774761.04	672.9229	<.0001*
WD	1	1	3720.87	35.5496	<.0001*
Device ID*WD	11	11	14922.88	12.9613	<.0001*

4) WS

Response PM2.5 Cal-PM2.5 Hinton

Regression Plot

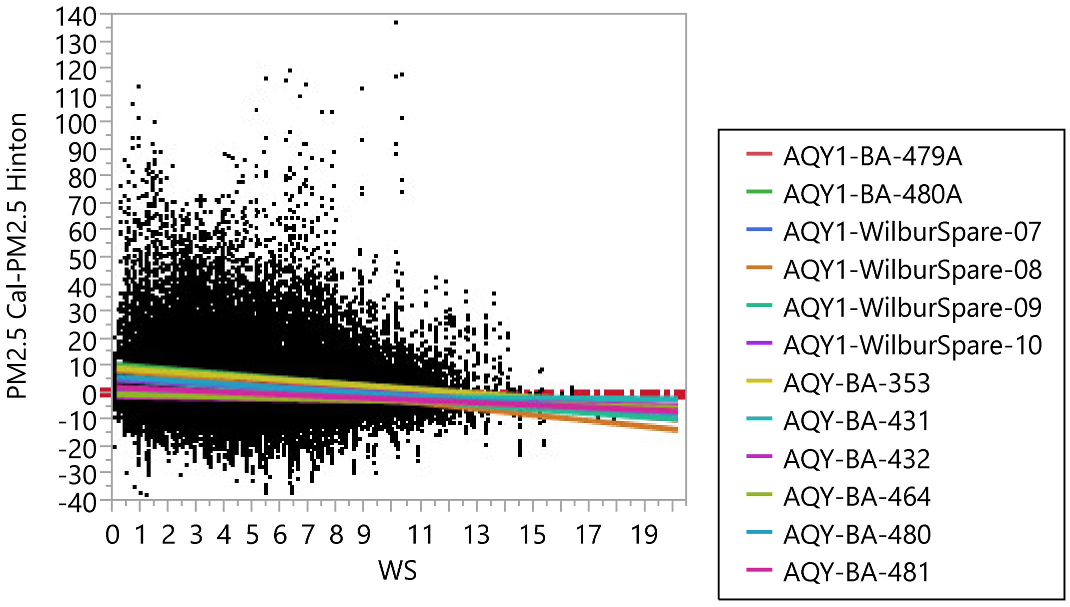

Summary of Fit

RSquare	0.096366
RSquare Adj	0.096155
Root Mean Square Error	10.09307
Mean of Response	2.256144
Observations (or Sum Wgts)	98710

Analysis of Variance

Source	DF	Sum of Squares	Mean Square	F Ratio
Model	23	1072090	46612.6	457.5693
Error	98686	10053148	101.9	**Prob > F**
C. Total	98709	11125238		<.0001*

Parameter Estimates

Term	Estimate	Std Error	t Ratio	Prob>|t|
Intercept	5.1205064	0.074671	68.57	<.0001*
Device ID[AQY1-BA-479A]	3.3707059	0.097802	34.46	<.0001*
Device ID[AQY1-BA-480A]	4.3355945	0.096321	45.01	<.0001*
Device ID[AQY1-WilburSpare-07]	-2.55065	0.140899	-18.10	<.0001*
Device ID[AQY1-WilburSpare-08]	1.1167889	0.11524	9.69	<.0001*
Device ID[AQY1-WilburSpare-09]	-0.302393	0.096337	-3.14	0.0017*
Device ID[AQY1-WilburSpare-10]	0.7644976	0.098337	7.77	<.0001*
Device ID[AQY-BA-353]	3.8525572	0.112535	34.23	<.0001*
Device ID[AQY-BA-431]	-2.67602	0.097168	-27.54	<.0001*
Device ID[AQY-BA-432]	-3.651623	0.095015	-38.43	<.0001*
Device ID[AQY-BA-464]	-3.365093	0.119723	-28.11	<.0001*
Device ID[AQY-BA-480]	0.8999731	0.126443	7.12	<.0001*
WS	-0.578803	0.012955	-44.68	<.0001*
Device ID[AQY1-BA-479A]*(WS-5.21175)	-0.420103	0.037734	-11.13	<.0001*
Device ID[AQY1-BA-480A]*(WS-5.21175)	-0.293701	0.036826	-7.98	<.0001*
Device ID[AQY1-WilburSpare-07]*(WS-5.21175)	0.2164306	0.058218	3.72	0.0002*
Device ID[AQY1-WilburSpare-08]*(WS-5.21175)	-0.529032	0.045097	-11.73	<.0001*
Device ID[AQY1-WilburSpare-09]*(WS-5.21175)	-0.158218	0.036828	-4.30	<.0001*
Device ID[AQY1-WilburSpare-10]*(WS-5.21175)	0.045518	0.037718	1.21	0.2275
Device ID[AQY-BA-353]*(WS-5.21175)	-0.138314	0.043397	-3.19	0.0014*
Device ID[AQY-BA-431]*(WS-5.21175)	0.4999528	0.036646	13.64	<.0001*
Device ID[AQY-BA-432]*(WS-5.21175)	0.4032501	0.03603	11.19	<.0001*
Device ID[AQY-BA-464]*(WS-5.21175)	0.3277782	0.047721	6.87	<.0001*
Device ID[AQY-BA-480]*(WS-5.21175)	-0.096367	0.04674	-2.06	0.0392*

Effect Tests

Source	Nparm	DF	Sum of Squares	F Ratio	Prob > F
Device ID	11	11	773979.80	690.7015	<.0001*
WS	1	1	203351.90	1996.189	<.0001*
Device ID*WS	11	11	70772.57	63.1576	<.0001*

Table S7. Results of ANCOVA F-tests for PM_{10} Raw – PM_{10} Hinton

Source	Nparm	DF	Sum of Squares	F Ratio	Prob > F
Device ID	10	10	326371.72	227.9290	<.0001*
Temp	1	1	494708.85	3454.910	<.0001*
RH	1	1	496814.29	3469.614	<.0001*
WD	1	1	22764.46	158.9807	<.0001*
WS	1	1	130766.60	913.2378	<.0001*
Device ID*Temp	10	10	20411.40	14.2548	<.0001*
Device ID*RH	10	10	51496.10	35.9635	<.0001*
Device ID*WD	10	10	1229.66	0.8588	0.5716
Device ID*WS	10	10	7536.15	5.2630	<.0001*

Supplementary Material: Underlying ANCOVA results for Figure 5 (PM10)

1. **Raw data**

1) **Temp**

Response PM10 Raw-PM10 Hinton
Regression Plot

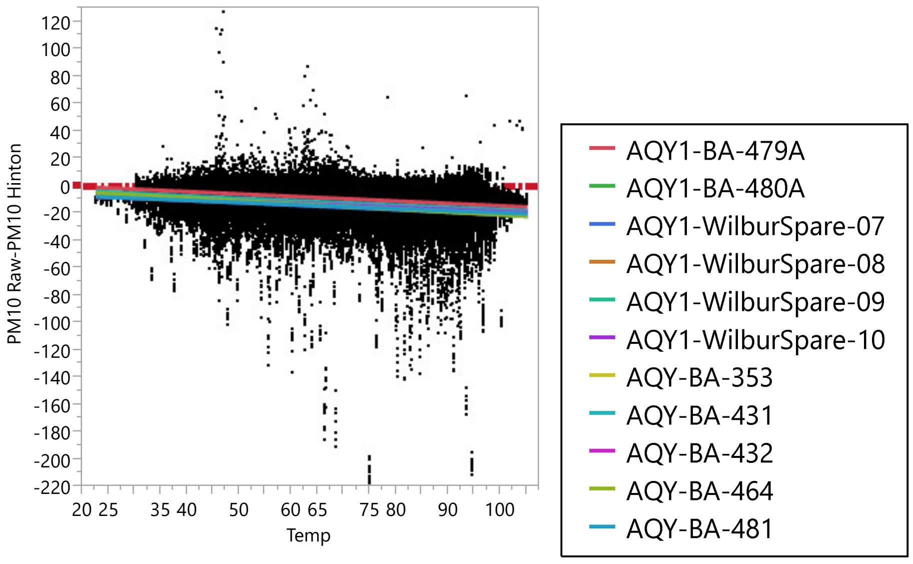

Summary of Fit

RSquare	0.068483
RSquare Adj	0.068327
Root Mean Square Error	12.18824
Mean of Response	-13.6493
Observations (or Sum Wgts)	125514

Analysis of Variance

Source	DF	Sum of Squares	Mean Square	F Ratio
Model	21	1370541	65263.9	439.3300
Error	125492	18642234	148.6	**Prob > F**
C. Total	125513	20012775		<.0001*

Parameter Estimates

Term	Estimate	Std Error	t Ratio	Prob>|t|
Intercept	-1.752309	0.152864	-11.46	<.0001*
Device ID[AQY1-BA-479A]	4.4147975	0.105685	41.77	<.0001*
Device ID[AQY1-BA-480A]	0.2707009	0.104486	2.59	0.0096*
Device ID[AQY1-WilburSpare-07]	-1.797157	0.118386	-15.18	<.0001*
Device ID[AQY1-WilburSpare-08]	0.0764122	0.104575	0.73	0.4650
Device ID[AQY1-WilburSpare-09]	-0.589659	0.104424	-5.65	<.0001*
Device ID[AQY1-WilburSpare-10]	-0.066245	0.106186	-0.62	0.5327
Device ID[AQY-BA-353]	-0.904416	0.104492	-8.66	<.0001*
Device ID[AQY-BA-431]	1.3459559	0.106554	12.63	<.0001*
Device ID[AQY-BA-432]	-0.351202	0.104497	-3.36	0.0008*
Device ID[AQY-BA-464]	-1.149976	0.137966	-8.34	<.0001*
Temp	-0.17071	0.002107	-81.00	<.0001*
Device ID[AQY1-BA-479A]*(Temp-70.2932)	-0.002753	0.006372	-0.43	0.6658
Device ID[AQY1-BA-480A]*(Temp-70.2932)	0.0042348	0.006285	0.67	0.5004
Device ID[AQY1-WilburSpare-07]*(Temp-70.2932)	-0.000812	0.00696	-0.12	0.9071
Device ID[AQY1-WilburSpare-08]*(Temp-70.2932)	-0.005583	0.006291	-0.89	0.3749

Term	Estimate	Std Error	t Ratio	Prob>|t|
Device ID[AQY1-WilburSpare-09]*(Temp-70.2932)	-0.048895	0.006279	-7.79	<.0001*
Device ID[AQY1-WilburSpare-10]*(Temp-70.2932)	0.0450604	0.006434	7.00	<.0001*
Device ID[AQY-BA-353]*(Temp-70.2932)	0.0045239	0.006285	0.72	0.4716
Device ID[AQY-BA-431]*(Temp-70.2932)	0.0041856	0.006415	0.65	0.5141
Device ID[AQY-BA-432]*(Temp-70.2932)	0.0148388	0.006287	2.36	0.0183*
Device ID[AQY-BA-464]*(Temp-70.2932)	-0.033681	0.008493	-3.97	<.0001*

Effect Tests

Source	Nparm	DF	Sum of Squares	F Ratio	Prob > F
Device ID	10	10	330990.08	222.8092	<.0001*
Temp	1	1	974758.85	6561.683	<.0001*
Device ID*Temp	10	10	19084.01	12.8466	<.0001*

2) RH

Response PM10 Raw-PM10 Hinton
Regression Plot

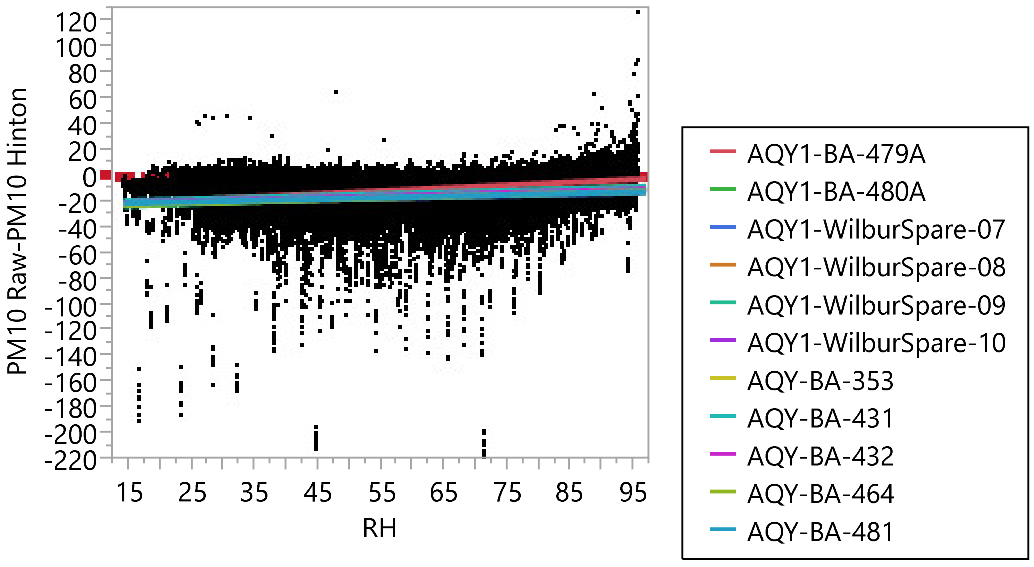

Summary of Fit

RSquare	0.072043
RSquare Adj	0.071887
Root Mean Square Error	12.16666
Mean of Response	-13.652
Observations (or Sum Wgts)	125533

Analysis of Variance

Source	DF	Sum of Squares	Mean Square	F Ratio
Model	21	1442402	68685.8	464.0070
Error	125511	18579086	148.0	**Prob > F**
C. Total	125532	20021488		<.0001*

Parameter Estimates

Term	Estimate	Std Error	t Ratio	Prob>|t|
Intercept	-22.71988	0.116025	-195.8	<.0001*
Device ID[AQY1-BA-479A]	4.4260349	0.105469	41.97	<.0001*
Device ID[AQY1-BA-480A]	0.3702104	0.104237	3.55	0.0004*
Device ID[AQY1-WilburSpare-07]	-1.926274	0.117923	-16.34	<.0001*
Device ID[AQY1-WilburSpare-08]	0.1756066	0.104322	1.68	0.0923
Device ID[AQY1-WilburSpare-09]	-0.468803	0.104171	-4.50	<.0001*
Device ID[AQY1-WilburSpare-10]	-0.026341	0.10597	-0.25	0.8037
Device ID[AQY-BA-353]	-0.810392	0.104244	-7.77	<.0001*
Device ID[AQY-BA-431]	1.500412	0.106272	14.12	<.0001*
Device ID[AQY-BA-432]	-0.255834	0.104248	-2.45	0.0141*
Device ID[AQY-BA-464]	-1.638897	0.13657	-12.00	<.0001*
RH	0.1463816	0.001812	80.80	<.0001*
Device ID[AQY1-BA-479A]*(RH-61.0428)	0.0780487	0.005451	14.32	<.0001*
Device ID[AQY1-BA-480A]*(RH-61.0428)	0.0246472	0.005389	4.57	<.0001*
Device ID[AQY1-WilburSpare-07]*(RH-61.0428)	-0.03791	0.006146	-6.17	<.0001*
Device ID[AQY1-WilburSpare-08]*(RH-61.0428)	0.0207402	0.005388	3.85	0.0001*
Device ID[AQY1-WilburSpare-09]*(RH-61.0428)	0.025184	0.005378	4.68	<.0001*
Device ID[AQY1-WilburSpare-10]*(RH-61.0428)	-0.012476	0.005519	-2.26	0.0238*
Device ID[AQY-BA-353]*(RH-61.0428)	-0.007165	0.005384	-1.33	0.1832
Device ID[AQY-BA-431]*(RH-61.0428)	-0.013991	0.005465	-2.56	0.0105*
Device ID[AQY-BA-432]*(RH-61.0428)	-0.020325	0.00539	-3.77	0.0002*
Device ID[AQY-BA-464]*(RH-61.0428)	-0.018953	0.007185	-2.64	0.0083*

Effect Tests

Source	Nparm	DF	Sum of Squares	F Ratio	Prob > F
Device ID	10	10	350455.82	236.7504	<.0001*
RH	1	1	966388.89	6528.439	<.0001*
Device ID*RH	10	10	50381.42	34.0352	<.0001*

3) WD

Response PM10 Raw-PM10 Hinton
Regression Plot

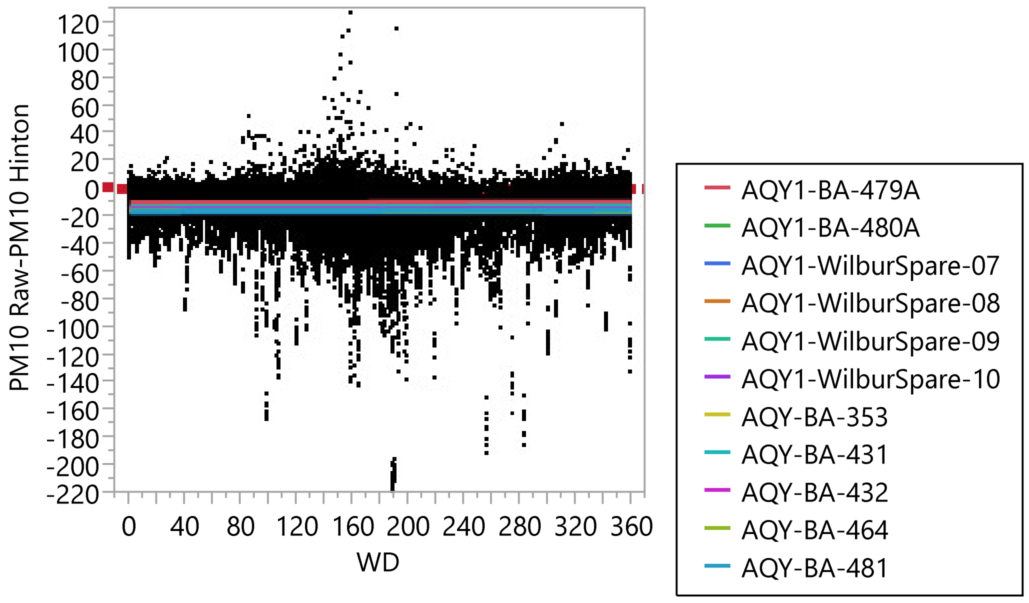

Summary of Fit

RSquare	0.017597
RSquare Adj	0.017432
Root Mean Square Error	12.52935
Mean of Response	-13.6776
Observations (or Sum Wgts)	124991

Analysis of Variance

Source	DF	Sum of Squares	Mean Square	F Ratio
Model	21	351401	16733.4	106.5924
Error	124969	19618217	157.0	**Prob > F**
C. Total	124990	19969618		<.0001*

Parameter Estimates

Term	Estimate	Std Error	t Ratio	Prob>|t|
Intercept	-13.95721	0.078203	-178.5	<.0001*
Device ID[AQY1-BA-479A]	4.4558869	0.108903	40.92	<.0001*
Device ID[AQY1-BA-480A]	0.3685113	0.107626	3.42	0.0006*
Device ID[AQY1-WilburSpare-07]	-1.945152	0.121484	-16.01	<.0001*
Device ID[AQY1-WilburSpare-08]	0.1796989	0.107713	1.67	0.0953
Device ID[AQY1-WilburSpare-09]	-0.470785	0.107558	-4.38	<.0001*
Device ID[AQY1-WilburSpare-10]	-0.072298	0.109418	-0.66	0.5088
Device ID[AQY-BA-353]	-0.815792	0.107632	-7.58	<.0001*
Device ID[AQY-BA-431]	1.4571736	0.109735	13.28	<.0001*
Device ID[AQY-BA-432]	-0.275281	0.107637	-2.56	0.0105*
Device ID[AQY-BA-464]	-1.547275	0.140818	-10.99	<.0001*
WD	0.0009065	0.000407	2.22	0.0261*
Device ID[AQY1-BA-479A]*(WD-171.011)	0.0008862	0.001227	0.72	0.4702
Device ID[AQY1-BA-480A]*(WD-171.011)	-0.000205	0.001208	-0.17	0.8650
Device ID[AQY1-WilburSpare-07]*(WD-171.011)	-0.00159	0.001395	-1.14	0.2543
Device ID[AQY1-WilburSpare-08]*(WD-171.011)	-0.000517	0.00121	-0.43	0.6694
Device ID[AQY1-WilburSpare-09]*(WD-171.011)	0.0014012	0.001208	1.16	0.2459
Device ID[AQY1-WilburSpare-10]*(WD-171.011)	-0.000746	0.00123	-0.61	0.5442
Device ID[AQY-BA-353]*(WD-171.011)	-0.000374	0.00121	-0.31	0.7570
Device ID[AQY-BA-431]*(WD-171.011)	0.0008184	0.001219	0.67	0.5019
Device ID[AQY-BA-432]*(WD-171.011)	5.1075e-5	0.001209	0.04	0.9663
Device ID[AQY-BA-464]*(WD-171.011)	-0.001282	0.001626	-0.79	0.4305

Effect Tests

Source	Nparm	DF	Sum of Squares	F Ratio	Prob > F
Device ID	10	10	349427.83	222.5872	<.0001*
WD	1	1	777.12	4.9503	0.0261*
Device ID*WD	10	10	880.44	0.5608	0.8470

4) WS

Response PM10 Raw-PM10 Hinton
Regression Plot

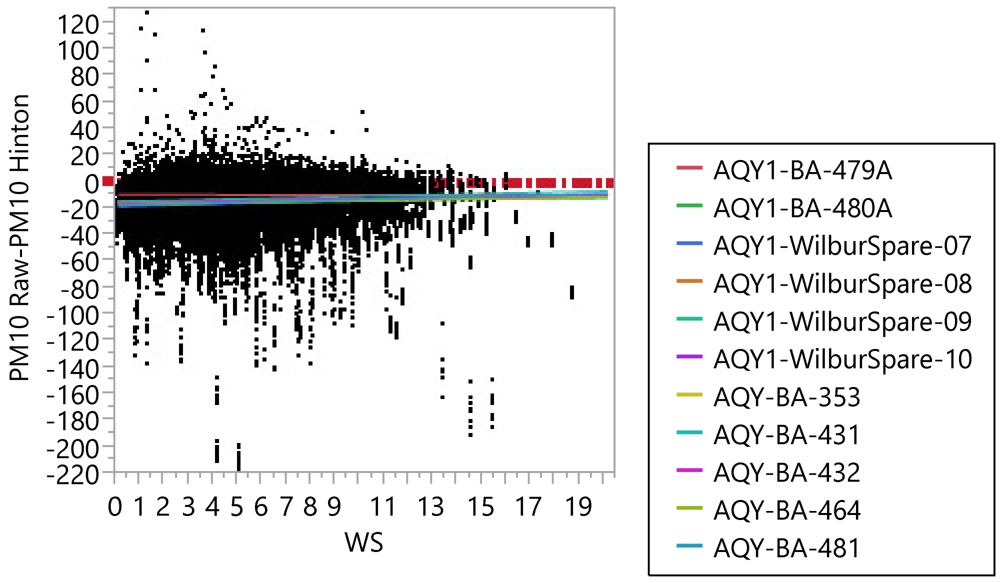

Summary of Fit

RSquare	0.02103
RSquare Adj	0.020866
Root Mean Square Error	12.50744
Mean of Response	-13.6776
Observations (or Sum Wgts)	124991

Analysis of Variance

Source	DF	Sum of Squares	Mean Square	F Ratio
Model	21	419970	19998.6	127.8387
Error	124969	19549648	156.4	**Prob > F**
C. Total	124990	19969618		<.0001*

Parameter Estimates

Term	Estimate	Std Error	t Ratio	Prob>|t|
Intercept	-15.2162	0.081754	-186.1	<.0001*
Device ID[AQY1-BA-479A]	4.45059	0.10871	40.94	<.0001*
Device ID[AQY1-BA-480A]	0.3559748	0.107442	3.31	0.0009*
Device ID[AQY1-WilburSpare-07]	-1.891945	0.121376	-15.59	<.0001*
Device ID[AQY1-WilburSpare-08]	0.1668288	0.10753	1.55	0.1208
Device ID[AQY1-WilburSpare-09]	-0.480158	0.107372	-4.47	<.0001*
Device ID[AQY1-WilburSpare-10]	-0.084868	0.109228	-0.78	0.4372
Device ID[AQY-BA-353]	-0.829441	0.107449	-7.72	<.0001*
Device ID[AQY-BA-431]	1.434334	0.109556	13.09	<.0001*
Device ID[AQY-BA-432]	-0.290459	0.107453	-2.70	0.0069*
Device ID[AQY-BA-464]	-1.534657	0.140422	-10.93	<.0001*
WS	0.2731482	0.014168	19.28	<.0001*
Device ID[AQY1-BA-479A]*(WS-5.20014)	-0.297792	0.042698	-6.97	<.0001*
Device ID[AQY1-BA-480A]*(WS-5.20014)	-0.053793	0.041819	-1.29	0.1983
Device ID[AQY1-WilburSpare-07]*(WS-5.20014)	0.1988765	0.048929	4.06	<.0001*
Device ID[AQY1-WilburSpare-08]*(WS-5.20014)	-0.049855	0.041848	-1.19	0.2335
Device ID[AQY1-WilburSpare-09]*(WS-5.20014)	-0.108517	0.04181	-2.60	0.0094*
Device ID[AQY1-WilburSpare-10]*(WS-5.20014)	0.0044734	0.042667	0.10	0.9165
Device ID[AQY-BA-353]*(WS-5.20014)	-0.01389	0.041837	-0.33	0.7399
Device ID[AQY-BA-431]*(WS-5.20014)	0.1200947	0.042506	2.83	0.0047*
Device ID[AQY-BA-432]*(WS-5.20014)	0.0370039	0.041849	0.88	0.3766
Device ID[AQY-BA-464]*(WS-5.20014)	-0.006749	0.056557	-0.12	0.9050

Effect Tests

Source	Nparm	DF	Sum of Squares	F Ratio	Prob > F
Device ID	10	10	345211.26	220.6725	<.0001*
WS	1	1	58149.38	371.7136	<.0001*
Device ID*WS	10	10	13713.41	8.7661	<.0001*

2. Calibrated data

1) Temp

Response PM10 Cal-PM10 Hinton

Regression Plot

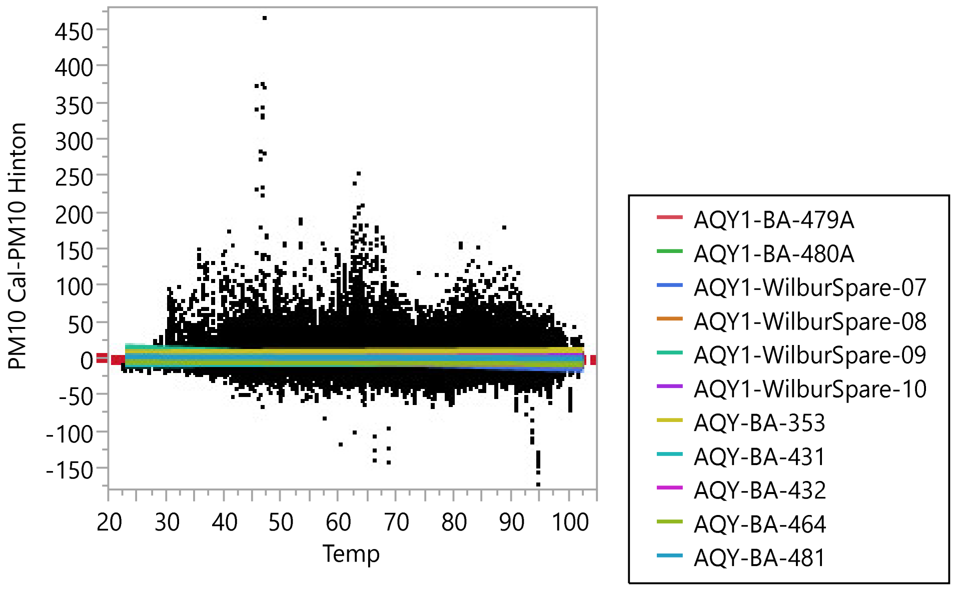

Summary of Fit

RSquare	0.096302
RSquare Adj	0.096098
Root Mean Square Error	18.26955
Mean of Response	2.755378
Observations (or Sum Wgts)	93027

Analysis of Variance

Source	DF	Sum of Squares	Mean Square	F Ratio
Model	21	3308049	157526	471.9512
Error	93005	31042871	334	**Prob > F**
C. Total	93026	34350921		<.0001*

Parameter Estimates

Term	Estimate	Std Error	t Ratio	Prob>|t|
Intercept	9.2171454	0.272628	33.81	<.0001*
Device ID[AQY1-BA-479A]	5.9291312	0.17667	33.56	<.0001*
Device ID[AQY1-BA-480A]	3.8910875	0.174152	22.34	<.0001*
Device ID[AQY1-WilburSpare-07]	-6.18679	0.260428	-23.76	<.0001*
Device ID[AQY1-WilburSpare-08]	1.5409373	0.209562	7.35	<.0001*
Device ID[AQY1-WilburSpare-09]	0.7561408	0.174182	4.34	<.0001*
Device ID[AQY1-WilburSpare-10]	2.4799858	0.177677	13.96	<.0001*
Device ID[AQY-BA-353]	9.8641662	0.203538	48.46	<.0001*
Device ID[AQY-BA-431]	-7.363009	0.176041	-41.83	<.0001*
Device ID[AQY-BA-432]	-5.510465	0.172013	-32.04	<.0001*
Device ID[AQY-BA-464]	-6.269469	0.220424	-28.44	<.0001*
Temp	-0.096854	0.003897	-24.86	<.0001*
Device ID[AQY1-BA-479A]*(Temp-68.0986)	0.0860669	0.010975	7.84	<.0001*
Device ID[AQY1-BA-480A]*(Temp-68.0986)	-0.08827	0.010809	-8.17	<.0001*
Device ID[AQY1-WilburSpare-07]*(Temp-68.0986)	-0.167583	0.014585	-11.49	<.0001*
Device ID[AQY1-WilburSpare-08]*(Temp-68.0986)	-0.085742	0.012575	-6.82	<.0001*
Device ID[AQY1-WilburSpare-09]*(Temp-68.0986)	-0.230168	0.01081	-21.29	<.0001*
Device ID[AQY1-WilburSpare-10]*(Temp-68.0986)	0.116471	0.011088	10.50	<.0001*
Device ID[AQY-BA-353]*(Temp-68.0986)	0.1382256	0.012088	11.44	<.0001*
Device ID[AQY-BA-431]*(Temp-68.0986)	0.1174458	0.010876	10.80	<.0001*
Device ID[AQY-BA-432]*(Temp-68.0986)	-0.000501	0.010588	-0.05	0.9622
Device ID[AQY-BA-464]*(Temp-68.0986)	0.0505831	0.013257	3.82	0.0001*

Effect Tests

Source	Nparm	DF	Sum of Squares	F Ratio	Prob > F
Device ID	10	10	2588302.1	775.4599	<.0001*
Temp	1	1	206202.7	617.7870	<.0001*
Device ID*Temp	10	10	354738.6	106.2803	<.0001*

2) RH

Response PM10 Cal-PM10 Hinton

Regression Plot

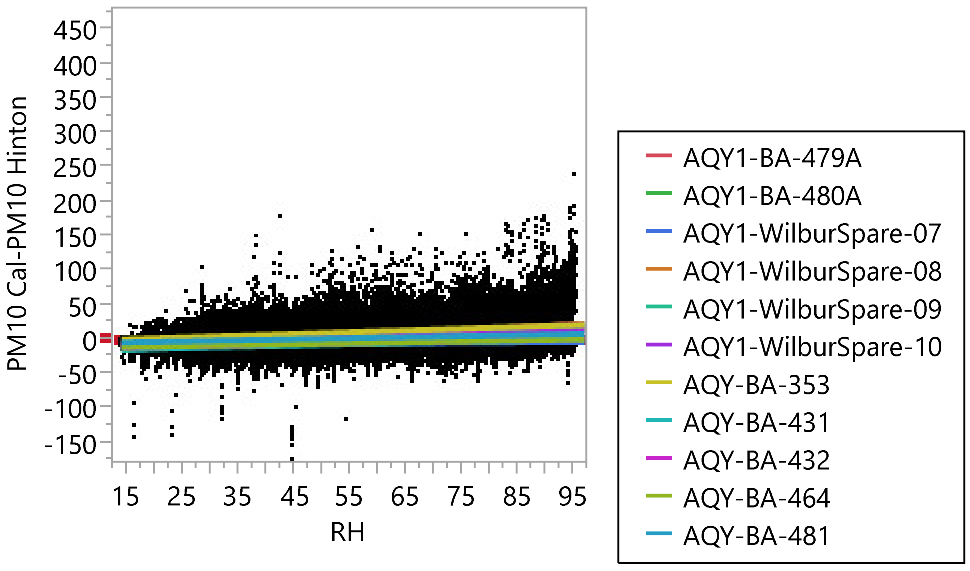

Summary of Fit

RSquare	0.162321
RSquare Adj	0.162131
Root Mean Square Error	17.58991
Mean of Response	2.753398
Observations (or Sum Wgts)	93035

Analysis of Variance

Source	DF	Sum of Squares	Mean Square	F Ratio
Model	21	5576563	265551	858.2623
Error	93013	28778684	309	**Prob > F**
C. Total	93034	34355247		<.0001*

Parameter Estimates

Term	Estimate	Std Error	t Ratio	Prob>|t|
Intercept	-12.38638	0.199054	-62.23	<.0001*
Device ID[AQY1-BA-479A]	5.883984	0.169518	34.71	<.0001*
Device ID[AQY1-BA-480A]	3.9625056	0.167022	23.72	<.0001*
Device ID[AQY1-WilburSpare-07]	-7.26788	0.242315	-29.99	<.0001*
Device ID[AQY1-WilburSpare-08]	1.5752158	0.199609	7.89	<.0001*
Device ID[AQY1-WilburSpare-09]	0.9005932	0.16705	5.39	<.0001*
Device ID[AQY1-WilburSpare-10]	2.620639	0.170438	15.38	<.0001*
Device ID[AQY-BA-353]	10.337856	0.195038	53.00	<.0001*
Device ID[AQY-BA-431]	-7.291341	0.168386	-43.30	<.0001*
Device ID[AQY-BA-432]	-5.343608	0.164745	-32.44	<.0001*
Device ID[AQY-BA-464]	-6.299662	0.207514	-30.36	<.0001*
RH	0.2423488	0.003103	78.11	<.0001*
Device ID[AQY1-BA-479A]*(RH-61.416)	0.178481	0.008679	20.56	<.0001*
Device ID[AQY1-BA-480A]*(RH-61.416)	0.16836	0.008547	19.70	<.0001*
Device ID[AQY1-WilburSpare-07]*(RH-61.416)	-0.183113	0.013028	-14.05	<.0001*
Device ID[AQY1-WilburSpare-08]*(RH-61.416)	0.0390604	0.010505	3.72	0.0002*
Device ID[AQY1-WilburSpare-09]*(RH-61.416)	0.1354415	0.008547	15.85	<.0001*
Device ID[AQY1-WilburSpare-10]*(RH-61.416)	-0.007049	0.008802	-0.80	0.4233
Device ID[AQY-BA-353]*(RH-61.416)	0.0139765	0.010172	1.37	0.1694
Device ID[AQY-BA-431]*(RH-61.416)	-0.05983	0.008528	-7.02	<.0001*
Device ID[AQY-BA-432]*(RH-61.416)	-0.084884	0.008402	-10.10	<.0001*
Device ID[AQY-BA-464]*(RH-61.416)	-0.107556	0.010812	-9.95	<.0001*

Effect Tests

Source	Nparm	DF	Sum of Squares	F Ratio	Prob > F
Device ID	10	10	2740368.6	885.6899	<.0001*
RH	1	1	1887895.8	6101.699	<.0001*
Device ID*RH	10	10	446633.3	144.3523	<.0001*

3) WD

Response PM10 Cal-PM10 Hinton
Regression Plot

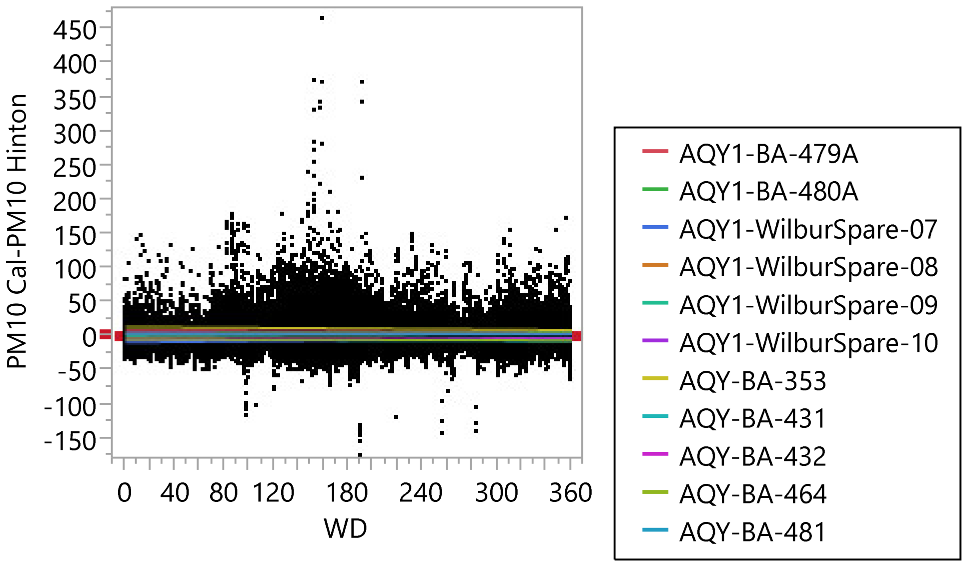

Summary of Fit

RSquare	0.081012
RSquare Adj	0.080804
Root Mean Square Error	18.45921
Mean of Response	2.784733
Observations (or Sum Wgts)	92507

Analysis of Variance

Source	DF	Sum of Squares	Mean Square	F Ratio
Model	21	2778040	132288	388.2335
Error	92485	31513559	341	**Prob > F**
C. Total	92506	34291599		<.0001*

Parameter Estimates

Term	Estimate	Std Error	t Ratio	Prob>|t|
Intercept	2.5949768	0.136665	18.99	<.0001*
Device ID[AQY1-BA-479A]	6.0839969	0.17841	34.10	<.0001*
Device ID[AQY1-BA-480A]	4.127344	0.175778	23.48	<.0001*
Device ID[AQY1-WilburSpare-07]	-7.348133	0.253226	-29.02	<.0001*
Device ID[AQY1-WilburSpare-08]	1.2293679	0.209926	5.86	<.0001*
Device ID[AQY1-WilburSpare-09]	1.0215688	0.175807	5.81	<.0001*
Device ID[AQY1-WilburSpare-10]	2.6134124	0.179418	14.57	<.0001*
Device ID[AQY-BA-353]	10.07598	0.205207	49.10	<.0001*
Device ID[AQY-BA-431]	-7.310933	0.177237	-41.25	<.0001*
Device ID[AQY-BA-432]	-5.318067	0.173361	-30.68	<.0001*
Device ID[AQY-BA-464]	-6.393577	0.218665	-29.24	<.0001*
WD	-0.00033	0.000695	-0.47	0.6356
Device ID[AQY1-BA-479A]*(WD-174.908)	-0.006204	0.00195	-3.18	0.0015*
Device ID[AQY1-BA-480A]*(WD-174.908)	0.0001231	0.001915	0.06	0.9488
Device ID[AQY1-WilburSpare-07]*(WD-174.908)	0.0188416	0.002946	6.40	<.0001*
Device ID[AQY1-WilburSpare-08]*(WD-174.908)	0.0051252	0.002369	2.16	0.0305*
Device ID[AQY1-WilburSpare-09]*(WD-174.908)	0.0066476	0.001915	3.47	0.0005*
Device ID[AQY1-WilburSpare-10]*(WD-174.908)	-0.002565	0.001955	-1.31	0.1895
Device ID[AQY-BA-353]*(WD-174.908)	-0.012466	0.002292	-5.44	<.0001*
Device ID[AQY-BA-431]*(WD-174.908)	-0.005198	0.001896	-2.74	0.0061*
Device ID[AQY-BA-432]*(WD-174.908)	0.0020727	0.00188	1.10	0.2702
Device ID[AQY-BA-464]*(WD-174.908)	-0.005817	0.002455	-2.37	0.0178*

Effect Tests

Source	Nparm	DF	Sum of Squares	F Ratio	Prob > F
Device ID	10	10	2737224.7	803.3121	<.0001*
WD	1	1	76.5	0.2245	0.6356
Device ID*WD	10	10	34388.9	10.0923	<.0001*

4) WS

Response PM10 Cal-PM10 Hinton
Regression Plot

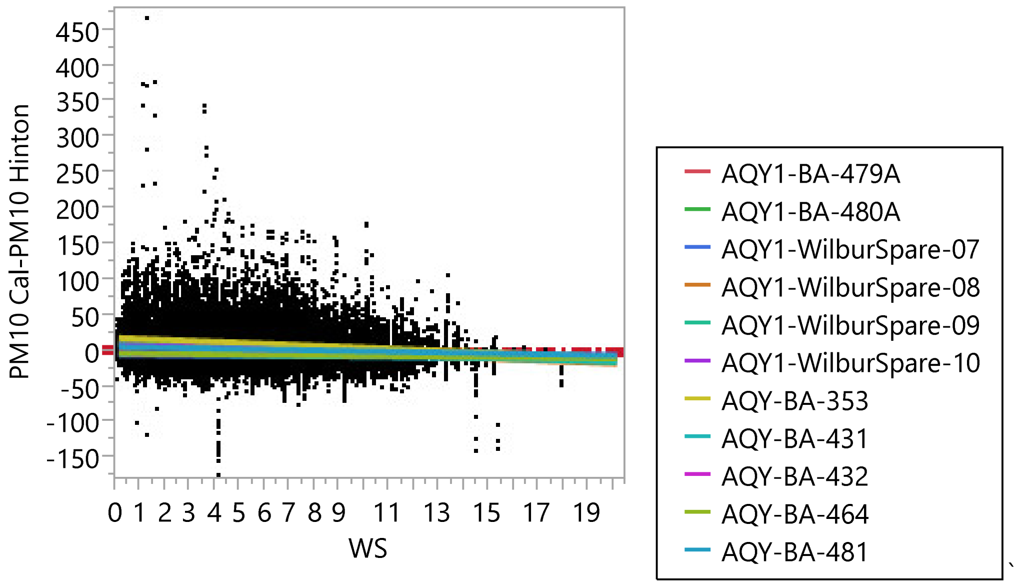

Summary of Fit

RSquare	0.096151
RSquare Adj	0.095946
Root Mean Square Error	18.30654
Mean of Response	2.784733
Observations (or Sum Wgts)	92507

Analysis of Variance

Source	DF	Sum of Squares	Mean Square	F Ratio
Model	21	3297167	157008	468.4997
Error	92485	30994432	335	**Prob > F**
C. Total	92506	34291599		<.0001*

Parameter Estimates

Term	Estimate	Std Error	t Ratio	Prob>|t|
Intercept	6.7134746	0.140356	47.83	<.0001*
Device ID[AQY1-BA-479A]	6.1095276	0.176972	34.52	<.0001*
Device ID[AQY1-BA-480A]	4.193422	0.174384	24.05	<.0001*
Device ID[AQY1-WilburSpare-07]	-7.46517	0.254677	-29.31	<.0001*
Device ID[AQY1-WilburSpare-08]	1.0500244	0.208505	5.04	<.0001*
Device ID[AQY1-WilburSpare-09]	1.0932016	0.174413	6.27	<.0001*
Device ID[AQY1-WilburSpare-10]	2.6515789	0.177987	14.90	<.0001*
Device ID[AQY-BA-353]	9.9838624	0.203631	49.03	<.0001*
Device ID[AQY-BA-431]	-7.258215	0.175918	-41.26	<.0001*
Device ID[AQY-BA-432]	-5.287034	0.172035	-30.73	<.0001*
Device ID[AQY-BA-464]	-6.357558	0.216121	-29.42	<.0001*
WS	-0.804303	0.024466	-32.87	<.0001*
Device ID[AQY1-BA-479A]*(WS-5.20529)	-0.453826	0.068569	-6.62	<.0001*
Device ID[AQY1-BA-480A]*(WS-5.20529)	-0.371468	0.066932	-5.55	<.0001*
Device ID[AQY1-WilburSpare-07]*(WS-5.20529)	0.6850505	0.105722	6.48	<.0001*
Device ID[AQY1-WilburSpare-08]*(WS-5.20529)	-0.543516	0.081946	-6.63	<.0001*
Device ID[AQY1-WilburSpare-09]*(WS-5.20529)	-0.414046	0.066935	-6.19	<.0001*
Device ID[AQY1-WilburSpare-10]*(WS-5.20529)	0.0754578	0.068517	1.10	0.2708
Device ID[AQY-BA-353]*(WS-5.20529)	-0.557325	0.078836	-7.07	<.0001*
Device ID[AQY-BA-431]*(WS-5.20529)	0.368784	0.066603	5.54	<.0001*
Device ID[AQY-BA-432]*(WS-5.20529)	0.5527986	0.065481	8.44	<.0001*
Device ID[AQY-BA-464]*(WS-5.20529)	0.4553143	0.086452	5.27	<.0001*

Effect Tests

Source	Nparm	DF	Sum of Squares	F Ratio	Prob > F
Device ID	10	10	2719688.2	811.5340	<.0001*
WS	1	1	362179.3	1080.715	<.0001*
Device ID*WS	10	10	118305.5	35.3015	<.0001*

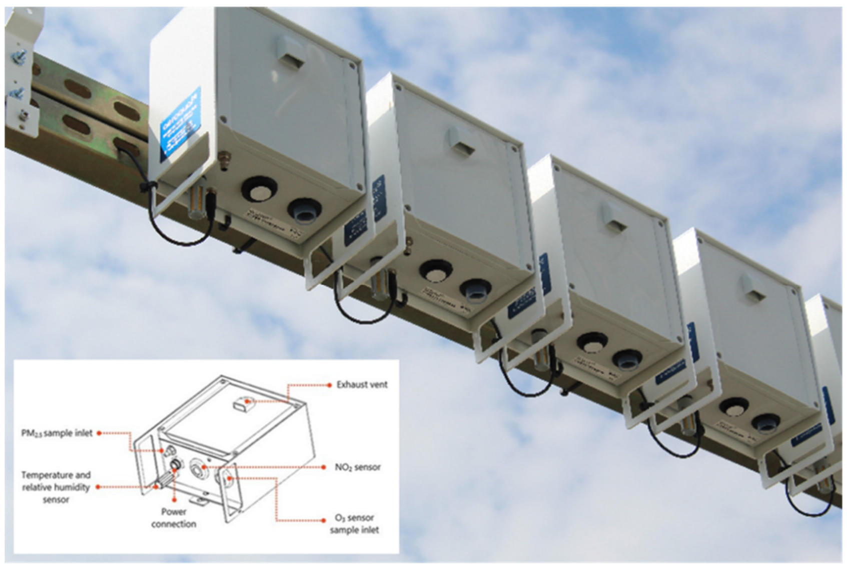

Figure S1. AQY1 Inlet Location Diagram, Sources: Diagram is obtained from Aeroqual Support (https://support.aeroqual.com/Guide/Identify+external+features/92), Source: Own Photo

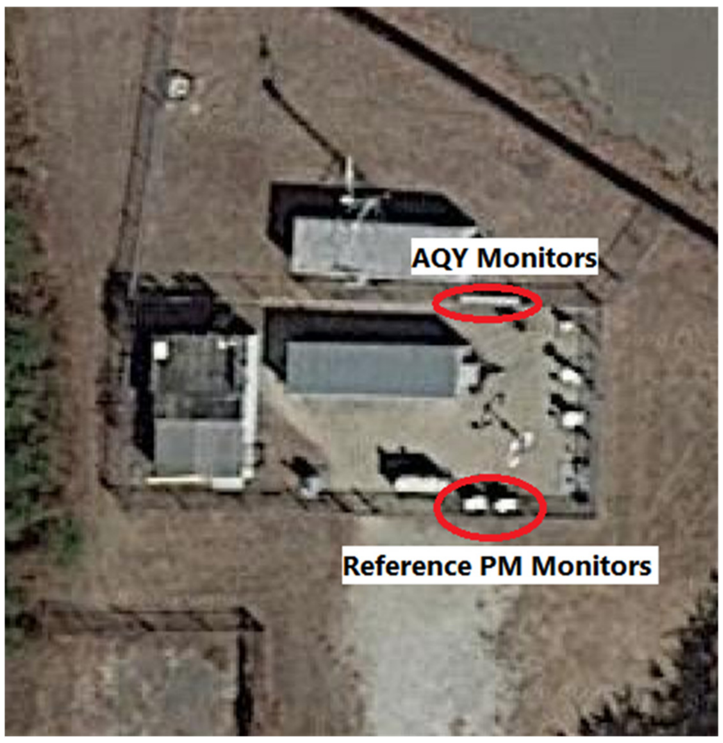

Figure S2. Location of Reference PM Monitors, Source: Google Maps

The figures below show the time series trends for the worst performing AQY1 monitors in terms of the R^2 measure. As shown in these figures, even the worst performing AQY1 monitors seem to trend well with measurements from the reference monitor, where the highs and lows from both the AQY1 and the reference monitors generally coincide over time.

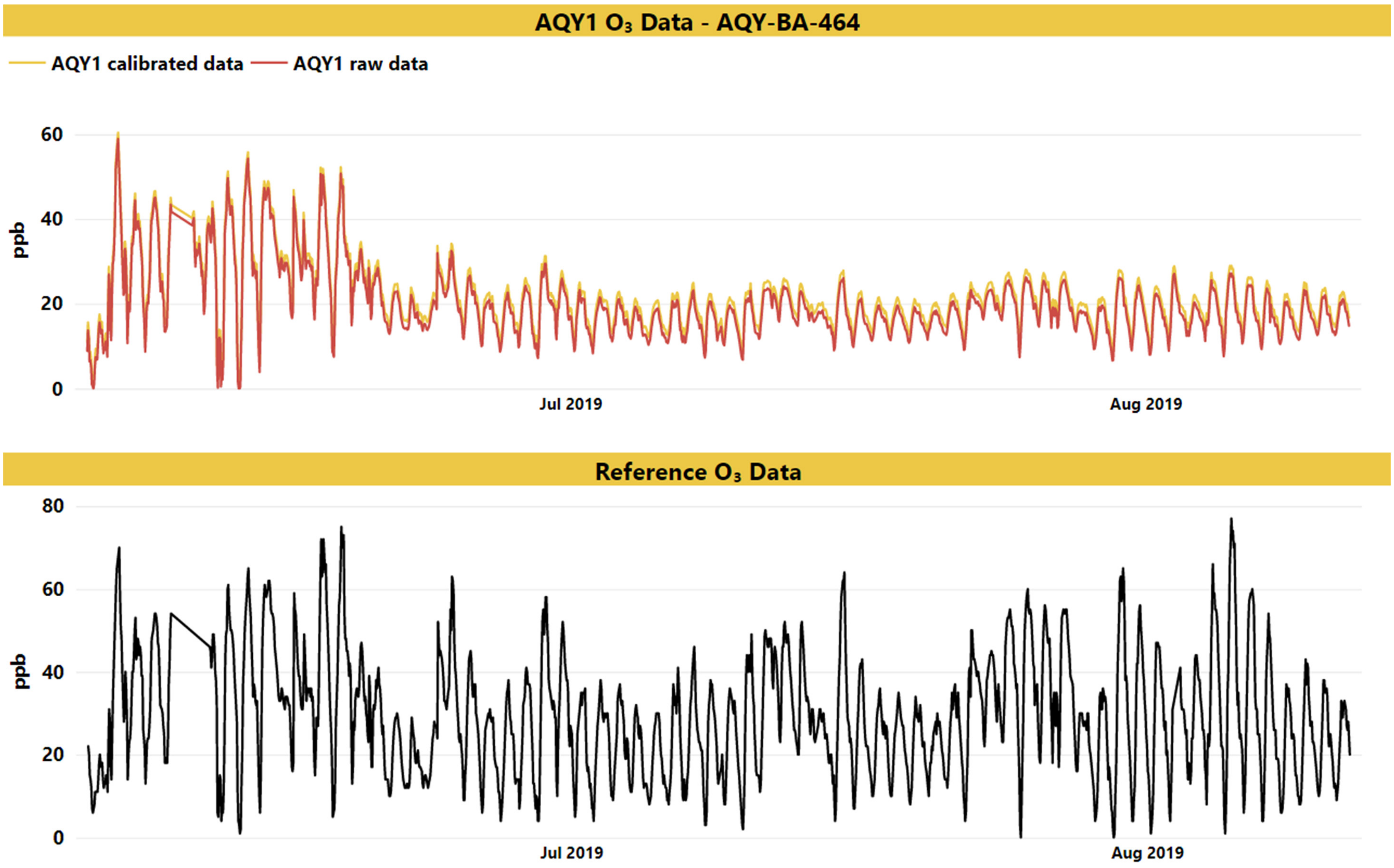

Figure S3 O_3 Data from the AQY1 Monitor AQY-BA-464 (Top Panel) vs the Reference Monitor at Hinton (Lower Panel) for the Time Period 6/4/19 - 8/11/19

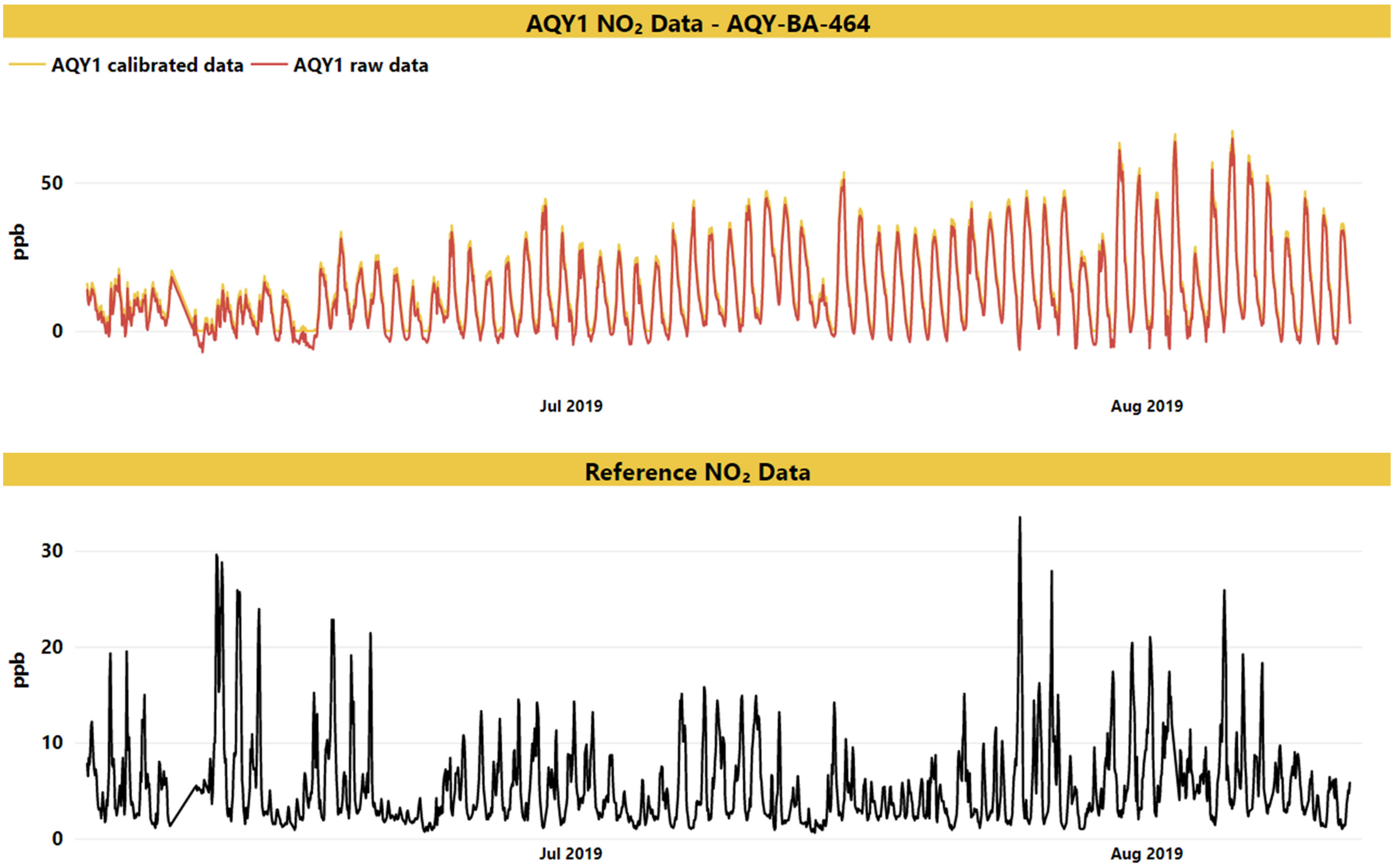

Figure S4 NO_2 Data from the AQY1 Monitor AQY-BA-464 (Top Panel) vs the Reference Monitor at Hinton (Lower Panel) for the Time Period 6/4/19 - 8/11/19

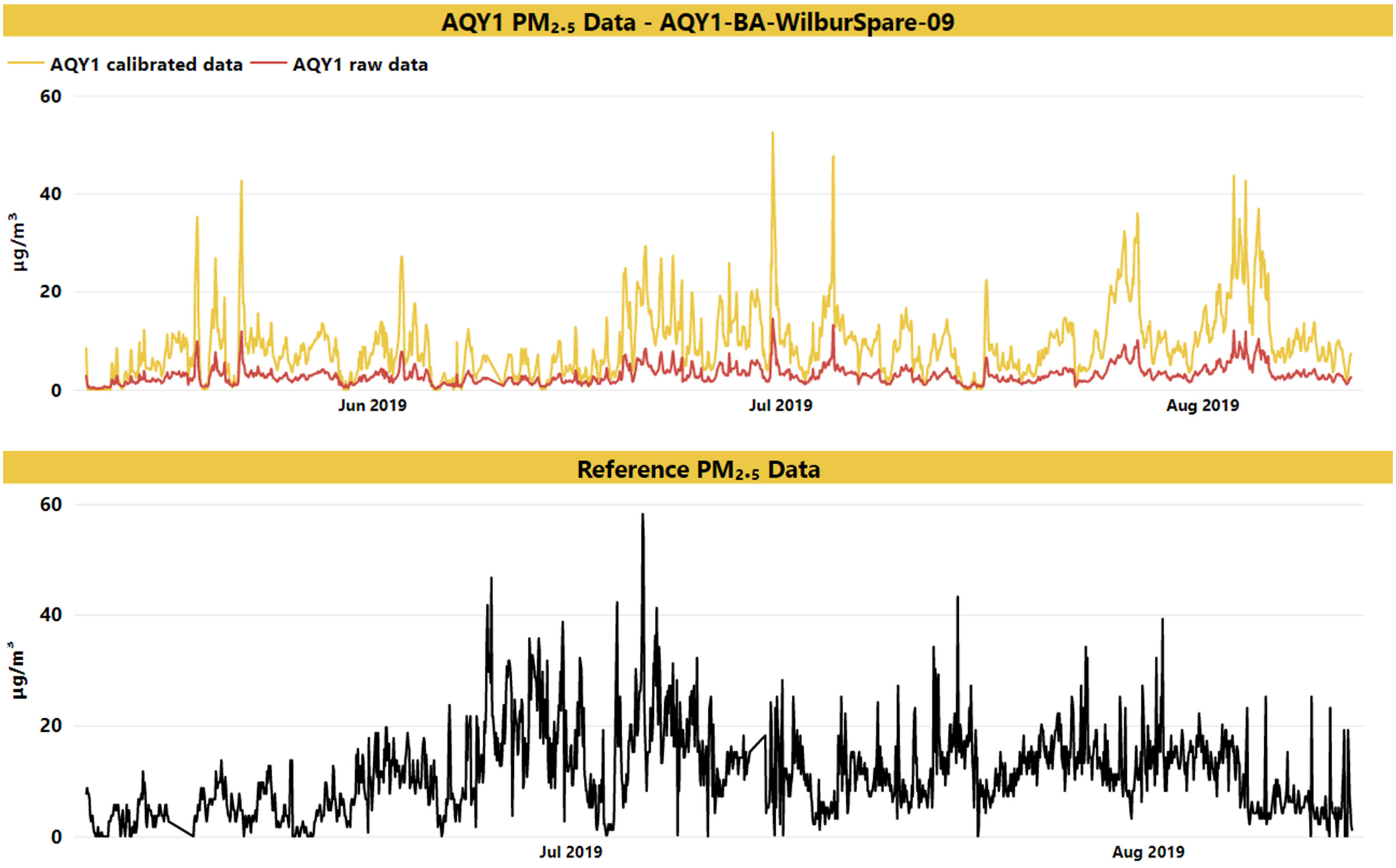

Figure S5 $PM_{2.5}$ Data from the AQY1 Monitor AQY-BA-WilburSpare09 (Top Panel) vs the Reference Monitor at Hinton (Lower Panel) for the Time Period 5/11/19 - 8/11/19

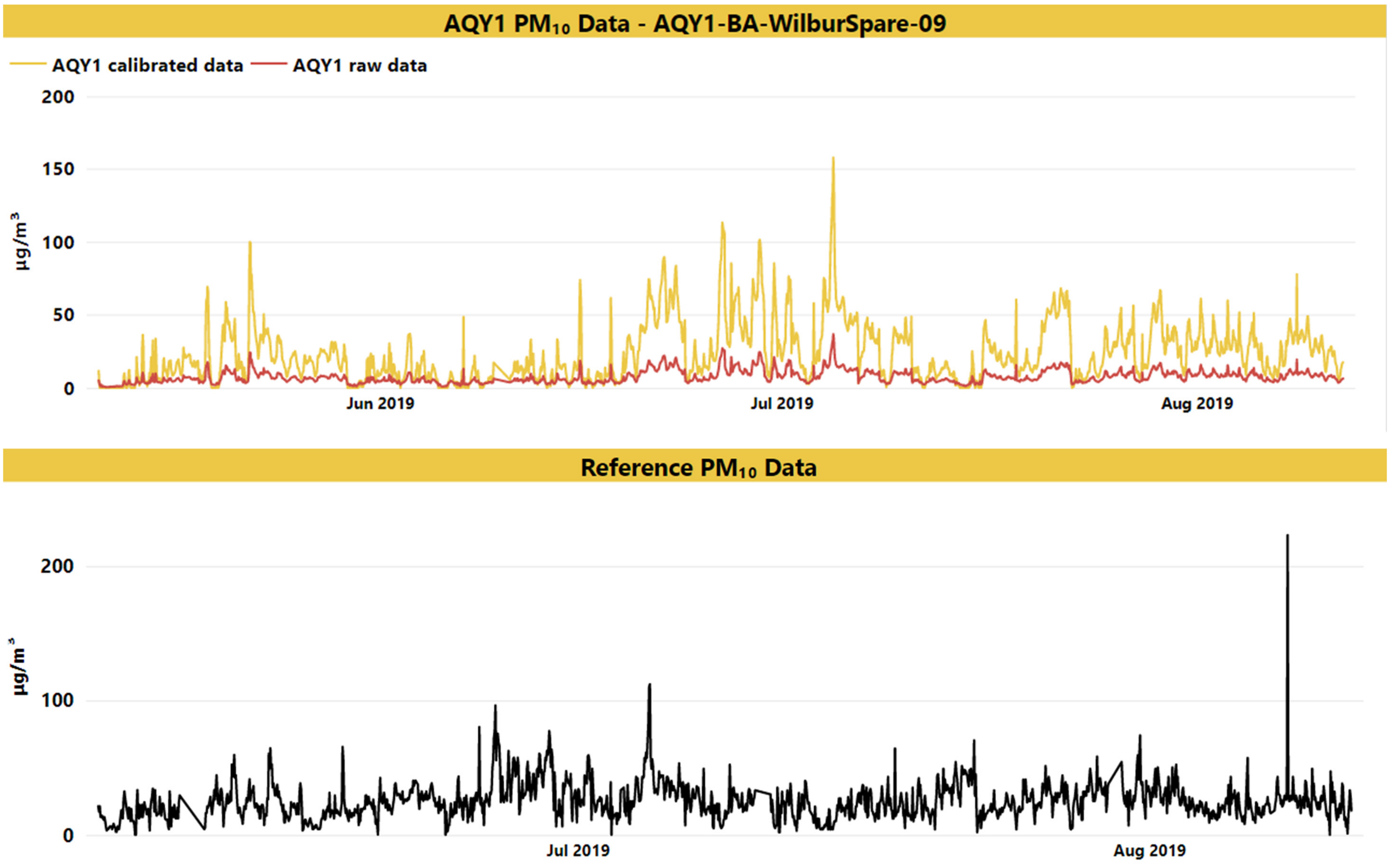

Figure S6 PM_{10} Data from the AQY1 Monitor AQY-BA-WilburSpare09 (Top Panel) vs the Reference Monitor at Hinton (Lower Panel) for the Time Period 5/11/19 - 8/11/19

Comparison with the United States Environmental Protection Agency's Air Quality Index Categories

Table S8 AQY1 Percentage of Correct Observations by AQI and Pollutant

Reference AQI Level	NO_2		PM_{10}		$PM_{2.5}$		O_3	
	Reference Monitor (Hinton) Observations	AQY1 Correct (%)	Reference Monitor (Hinton) Observations	AQY1 Correct (%)	Reference Monitor (Hinton) Observations	AQY1 Correct (%)	Reference Monitor (Hinton) Observations	AQY1 Correct (%)
Good	8,141*	98.9 %	10,723	95.1 %	8,265	75.6 %	8,938	96.2 %
Moderate	None	NA	46	95.2 %	2,546	62.3 %	206	64.7 %
Unhealthy for Sensitive Groups	None	NA	None	NA	None	NA	4	40 %
Total	8,141	98.9 %	10,769	95.1 %	10,811	72.4 %	9,148	95.5 %

NA: not available. *: The NO_2 sensors at the reference monitor's site (Hinton) were offline for a few months during our data collection period.
